# Slow Magnetic Relaxation in Mono‐ and Bimetallic Lanthanide Tetraimido‐Sulfate S(N*t*Bu)_4_^2−^ Complexes

**DOI:** 10.1002/chem.202101076

**Published:** 2021-06-24

**Authors:** Jochen Jung, Florian Benner, Regine Herbst‐Irmer, Selvan Demir, Dietmar Stalke

**Affiliations:** ^1^ Institut für Anorganische Chemie Georg-August Universität Göttingen Tammannstraße 4 37077 Göttingen Germany; ^2^ Department of Chemistry Michigan State University 578 S Shaw Lane East Lansing MI 48824 USA

**Keywords:** slow magnetic relaxation, lanthanide complexes, single-molecule magnets, SN complexes, tetraimido sulfate

## Abstract

Lanthanide ions are particularly well‐suited for the design of single‐molecule magnets owing to their large unquenched orbital angular momentum and strong spin‐orbit coupling that gives rise to high magnetic anisotropy. Such nanoscopic bar magnets can potentially revolutionize high‐density information storage and processing technologies, if blocking temperatures can be increased substantially. Exploring non‐classical ligand scaffolds with the aim to boost the barriers to spin‐relaxation are prerequisite. Here, the synthesis, crystallographic and magnetic characterization of a series of each isomorphous mono‐ and dinuclear lanthanide (Ln=Gd, Tb, Dy, Ho, Er) complexes comprising tetraimido sulfate ligands are presented. The dinuclear Dy complex [{(thf)_2_Li(N*t*Bu)_2_S(*t*BuN)_2_DyCl_2_}_2_ ⋅ ClLi(thf)_2_] (**1c**) shows true signatures of single‐molecule magnet behavior in the absence of a dc field. In addition, the mononuclear Dy and Tb complexes [{(thf)_2_Li(N*t*Bu)_2_S(*t*BuN)_2_LnCl_2_(thf)_2_] (**2b**,**c**) show slow magnetic relaxation under applied dc fields.

## Graphical abstract

**The first series** of isomorphous lanthanide Gd to Er complexes containing the tetraimido sulfate ligand renders these compounds compelling to be investigated for their single‐molecule magnet behaviour. The displayed Dy dimer shows slow magnetic relaxation at zero field. The monomeric Tb and Dy complexes show field‐induced single‐molecule magnet behaviour. The ligand holds great potential for higher blocking systems, that is, removal of chloride ions, lithium transmetalation and promotion of the magnetic communication between the metal ions.

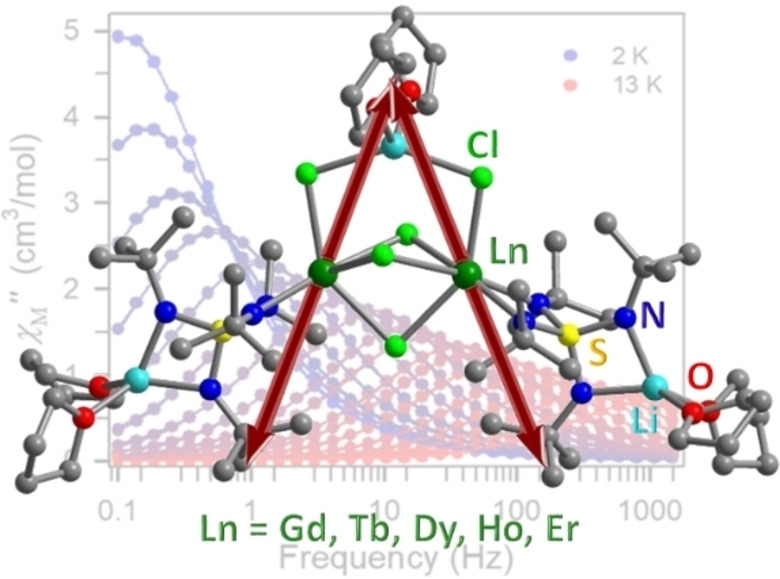

## Introduction

Single‐molecule magnets (SMMs)^[1]^ represent a whole new flourishing area of materials based molecular chemistry.^[2]^ It was realized that not only the nuclearity of an open shell cluster could favorably influence the magnetism features of a molecule. In addition, weak interactions like hydrogen bonds can tune them.^[3]^ To maintain those features even in solution the non‐covalent linkage can be replaced by covalent bonds from polydentate ligands.^[4]^ From the same spirit, the influence of a heavy atom was detected, predominantly from metal bonded heavy halides in HF EPR spectroscopy,^[5]^ and was rationalized by theory.^[6]^ More recently heavy main group elements bonded to the paramagnetic transition metal like germanium and tin^[7]^ are employed in molecular arrays to take advantage of the spin‐orbit coupling. Within the realm of ligand design in heterobimetallic SMMs, we want to take advantage of the polyimido sulfur ligands as the S−N bonds range between the weak non‐covalent interactions as hydrogen bonding and the strong covalent (multiple) C−N and C−O bonds. The S−N bond is perfectly suited for that endeavor as it is covalent with a profound ionic contribution. The sulfur in the middle of coordinating nitrogen atoms can easily adapt to various metals, both geometrically and electronically.[^8^, ^9^] A clear indication is already the fact that in all known metal complexes of the *S*‐alkyltriimidosulfonates [RS(NR)_3_]^−^ (M=Li, Ba, Al, Zn) and in the triimidosulfonic acid MeS(N*t*Bu)_2_NH*t*Bu the sum of all three S−N bond lengths is constant at 4.70(2) Å.[^10^, ^11^] Apart from distance considerations there is vibrational spectroscopic^[12]^ as well as experimental^[13]^ and computational^[14]^ charge density evidence for very polar covalent S−N bonding. First detected in the triimidesulfites [S(NR)_3_]^2−[15,16]^ it was later also found in the tetraimido sulfates [S(NR)_4_]^2−^.^[17]^ We have been already successful employing those ligands to d‐block SMMs with the [MeS(N*t*Bu)]^−^ ligand in [Co{(N*t*Bu)_3_SMe}_2_], (with *U*_eff_=75 cm^−1^, *T*_B_=2.6 K and a hysteresis loop at 2 K),^[18]^ accompanying of a series of complexes with the [S(N*t*Bu)_4_]^2−^ anion.[^19^, ^20^] This fuelled the idea to transfer the ligand concept to f‐block metals. First results are presented in here.

## Results and Discussion

**Scheme 1:**
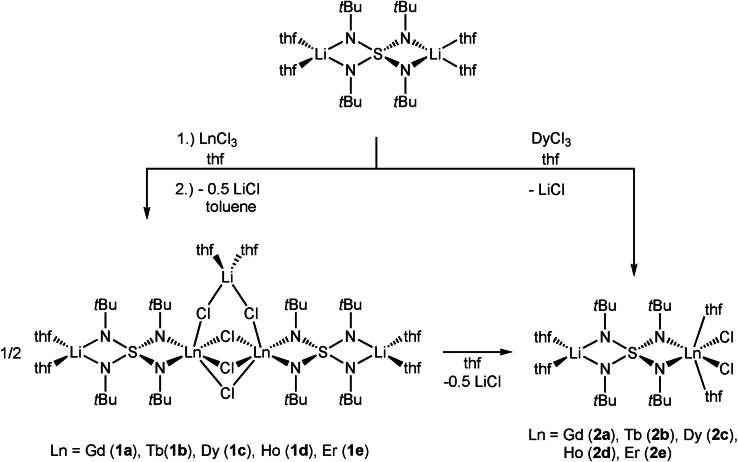
Synthesis of the lanthanide complexes **1a‐e** in the row from gadolinium to erbium [{Cl_2_Ln(N*t*Bu)_2_S(N*t*Bu)_2_Li(thf)_2_} ⋅ {ClLi(thf)_2_}] and the isolation of **2a**–**e** after dissolving **1** in thf as well as direct lithium lanthanide exchange from the starting material.

Single lithium lanthanide metal exchange in [(thf)_4_Li_2_(N*t*Bu)_4_S] with the appropriate lanthanide(III) chloride in thf yields after extraction and crystallization from toluene the binuclear lanthanide(III) compounds [{(thf)_2_Li(N*t*Bu)_2_S(*t*BuN)_2_LnCl_2_}_2_ ⋅ ClLi(thf)_2_] **1 a**–**e** with **a**: Ln=Gd, **b**: Tb, **c**: Dy, **d**: Ho, **e**: Er (Scheme 1). The bimetallic dysprosium complex **1 c** shows true single‐molecule magnet behavior in the absence of dc fields.

**Figure 1:**
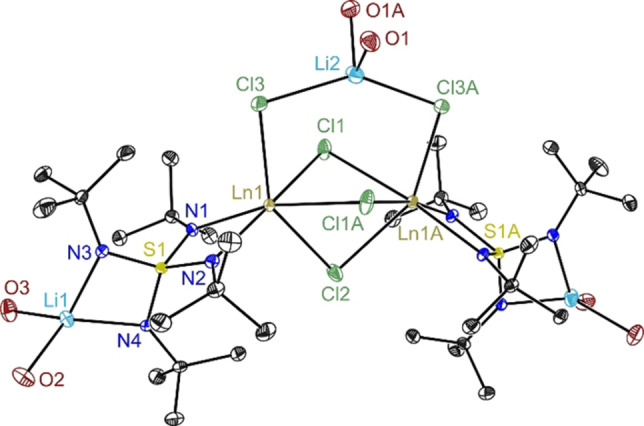
Crystal structure of **1a**–**e** (**a**: Ln=Gd, **b**: Tb, **c**: Dy, **d**: Ho, **e**: Er). Anisotropic displacement parameters are depicted at the 50 % probability level for the dysprosium complex (for the others see Figures S1, S2, S3, S4). The thf molecules are reduced to the coordinating oxygen atoms. Hydrogen atoms and disordered toluene molecule at lattice position are omitted for clarity. Selected bond length [Å] and angles [°] in average for **1a**–**e**. Ln1−N1 2.262, Ln1−N2 2.285, Ln1−Cl1 2.808, Ln1−Cl1 A 2.759, Ln1−Cl2 2.698, Ln1−Cl3 2.644, N1−Ln1−N2 61.61.

We found these dimeric complexes, dissolved in small amounts of thf to recrystallize within hours at room temperature, forming the mononuclear lanthanide(III) compounds [{(thf)_2_Li(N*t*Bu)_2_S(*t*BuN)_2_LnCl_2_(thf)_2_] **2 a**–**e** (**a**: Ln=Gd, **b**: Tb, **c**: Dy, **d**: Ho, **e**: Er) in good yields and purity. Instead, **2 c** was also isolated upon layering the reaction mixture with *n*‐pentane. However, since the crystallization parameters for this reaction are very tedious to control and the final product is insoluble in thf the derivatives rather precipitate than crystallize. Therefore, we concentrated on the optimization of the synthetic route to give the μ‐LiCl‐bridged dimers **1 a**–**e**. They crystallize in the monoclinic space group *C*2/c with half a molecule and half a toluene molecule in the asymmetric unit (Figure 1). The lanthanide ions are coordinated in a distorted octahedral geometry exhibiting pinched axial Cl2 and Cl3 ions with an angle of 144.5° deviating from the ideal axial 180° angle. Two adjacent positions are occupied by the *N*,*N*‐chelating SN‐ligand while the four remaining ligands are chlorine atoms. Three of them bridge both heterobimetallic tetraimido sulfate moieties. The fourth chlorine atom co‐complexes a lithium cation, providing the Cl−Li−Cl link to the second half in **1 a**–**e**. All four Ln−Cl distances differ and range for **1 a** from 2.671 Å to 2.834 Å but shorten monotonously proceeding the period from Gd to Er, following the decreasing ion radii^[21]^ from 2.617 to 2.784 Å in **1 e**. The same trend can be found for the Ln−N distances that decrease from an average value of 2.299 Å in **1 a** to 2.253 Å in **1 e**. As seen before in other complexes containing the tetraimido sulfate ligand S(N*t*Bu)_4_^2−^, the S−N bond distances for all metal complexes are almost identical. Nevertheless, it should be stated that the av. S−N1/N2 distances from the lithium coordinating nitrogen atoms of 1.567 Å are significantly shorter than the S−N3/N4 distances of 1.626 Å to the lanthanide‐coordinated nitrogen atoms. That mirrors the two opposite coordination sites of the tetrahedral ligand which are unsymmetrically coordinated by a lithium ion at the site with shorter S−N bonds and the lanthanide(III) ion at the opposite site. The triply positive charged lanthanides obviously are in much stronger demand of the negatively charged nitrogen atoms, for their part taking advantage of the electropositive central sulfur atom. A stronger demand of the negatively charged nitrogen atoms causes a longer distance between the nitrogen and the positively charged sulfur. The reduced lanthanide radii are also displayed in the reduction of the Ln1⋅⋅⋅Ln1A distances that ranges from 3.835 Å in **1 a** to 3.752 Å in **1 e**. Interestingly, the reduced radii have the opposite effect on the N1−Ln1−N2 angles increasing from 60.91° in **1 a** to 62.24° in **1 e**. Even though all Cl−Ln−Cl angles for one specific metal are different, they remain almost identical when proceeding from one lanthanide cation to the next. The same is valid for the N−S1−N angles. A comparable coordination is found in a series of rare earth metal complexes with β‐diketiminato ligands. Substitution at the nitrogen position with 2,6‐dimethylphenyl,^[22]^ 2,6‐diisopropylphenyl^[23]^ and mesityl^[24]^ gave binuclear complexes with unsymmetrically coordinated metal ions, each attached to one β‐diketiminato ligand, three bridging and a pendent chlorine atoms at one and a thf molecule at the second metal. The averaged Dy−μ‐Cl bond distances of the tetra chloro coordinated dysprosium in the complex with the mesityl substituted ligand^[24]^ is on average 2.767 Å, hence close to those found in **1 c** with 2.726 Å. The same is valid for the Dy−N bond lengths of 2.313 Å and 2.270 Å, respectively.

**Figure 2:**
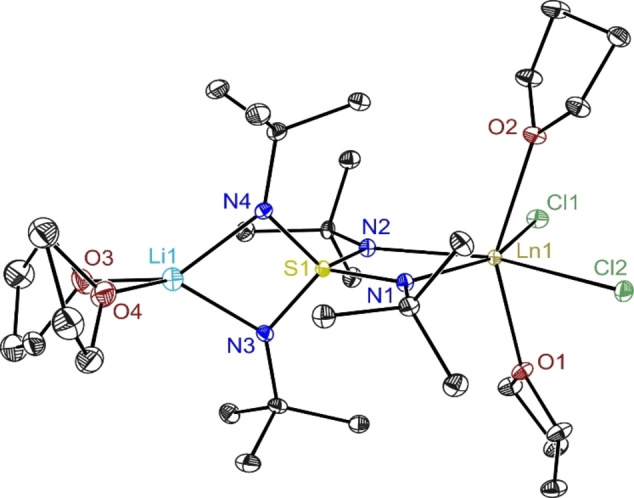
Crystal structure of **2a**–**e** (**a**: Ln=Gd, **b**: Tb, **c**: Dy, **d**: Ho, **e**: Er). Anisotropic displacement parameters are depicted at the 50 % probability level for the dysprosium complex (for the others see Figures S6, S7, S9, S10). Hydrogen atoms are omitted for clarity. Selected bond length [Å] and angles [°] in average for **2a**–**e**. Ln1−N1 2.330, Ln1−N2 2.325, Ln1−Cl1 2.648, Ln1−Cl2 2.639, Ln1−O1 2.418, Ln1−O2 2.418, O1−Ln1−O2 149.88, N1−Ln1−N2 61.00, Cl1−Ln1−Cl2 108.05 (**2a**) 106.59 (**2b**–**e**).

To maximize yields for the non μ‐LiCl‐bridged mononuclear complexes **2 a**–**e**, the reaction mixture can be stored at lower temperature for several hours or days. Instead of isolating the dimer by extraction with toluene first monomeric species **2 c** can as well be obtained directly from the filtered reaction mixture at reduced temperature after addition of *n‐*pentane or by vapor diffusion. **2 a**–**e** crystallize in the orthorhombic space group Pca2_1_ with one molecule in the asymmetric unit (Figure 2). The lanthanide ions are considerably distorted octahedrally coordinated. Two adjacent equatorial positions are occupied by the *N*,*N*‐chelating nitrogen atoms from the S(N*t*Bu)_4_^2−^ ligand and the remaining two by chlorine atoms. Two thf molecules reside in the apical positions, one each. Presumably for steric reasons, they are bent away from the *t*Bu substituents of the ligand. The averaged Ln−N bond distances are slightly longer compared to **1** and range from 2.354 Å (**2 a**) to 2.304 Å (**2 e**). That mirrors the overall trend of stronger interaction for the smaller and hence harder metal ions proceeding from Gd to Er. The Ln1−Cl bond distances in **2** correspond best to the lithium co‐coordinated chlorine atoms in **1**. The Ln1−O distances are in the region normally found for Ln−thf bonds. The O1−Ln1−O2 angle, which only insignificantly increases from **2 a** to **2 e** (av. 149.88°), displays the strong distortion from an idealized octahedron. The changes in S1‐N bond lengths in **2** are almost identical in all compounds **a** to **e** and are not significantly different from those in **1 a**–**e**. Like in **1**, a very acute N1−Ln1−N2 angle from 60.27° to 61.61° is found in **2 a**–**e** facilitated by the acute N1−S1−N2 angle (av. 92.83°), far off the ideal tetrahedral angle anticipated from a *T*_d_ symmetrical S(N*t*Bu)_4_^2−^ dianion.^[20]^ The astonishing flexibility of this ligand that can adapt to a wide variety of metals was noticed earlier.[^8^, ^9^, ^17^]

**Figure 3:**
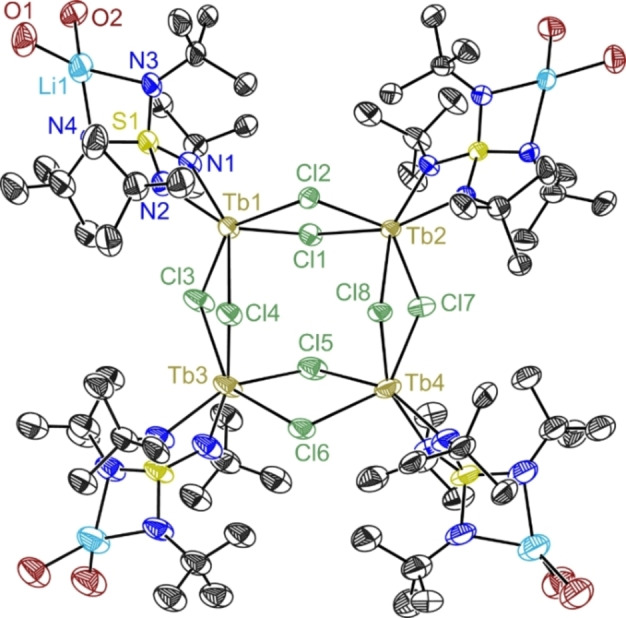
Crystal structure of **3b**. Anisotropic displacement parameters are depicted at the 50 % probability level. Hydrogen atoms and disordered ligands are omitted for clarity. Solvent molecules at lattice positions have not been refined.

In the synthesis for complex **1 b** a minute amount of the tetranuclear side product [{(thf)_2_Li(N*t*Bu)_2_S(*t*BuN)_2_LnCl_2_}_4_] **3 b** could be isolated from toluene (Figure 3). It consists of four units of **2 b** where the thf molecules coordinated to the terbium ions are replaced by two chlorine ions of the next unit, providing the link to a tetrameric tetranuclear cyclic species. Unfortunately, **3 b** is seriously disordered and the resulting geometry is vague. Similar crystals could not be found for dysprosium to erbium, but in the case of gadolinium a very poorly scattering crystal with very poor quality could be recovered which had cell parameters indicating that a similar structural type might exist for Gd too. Nevertheless, we are confident that the tetrameric structural motive **3** is only present below the detection level because the current crystallization strategies do not promote its formation.

Tetranuclear **3 b** contains a similar structural core as the dinuclear **1 b** and the mononuclear **2 b**. The averaged Tb−N distances of 2.309 Å are very close for all compounds (**1 b**: 2.283 Å and **2 b**: 2.335 Å). All Tb−Cl distances are similar, although the terbium atoms in **3 b** are fourfold chlorine coordinated and those in **1 b** and **2 b** only two‐fold. With the rising nuclearity in **3 b** the TbΛTb distance increases to 4.307 Å compared to 3.831 Å in **1 b**. The acute N1−Tb−N2 bite angles of 61.22° (**1 b**), 60.67° (**2 b**), and 61.10° (**3 b**) differ only marginally. The same is valid for the N1−S1−N2 ligand angles at the Tb side of 91.47° (**1 b**), 92.77° (**2 b**) and 92.60° (**3 b**).

### Static magnetic susceptibility measurements

**Figure 4:**
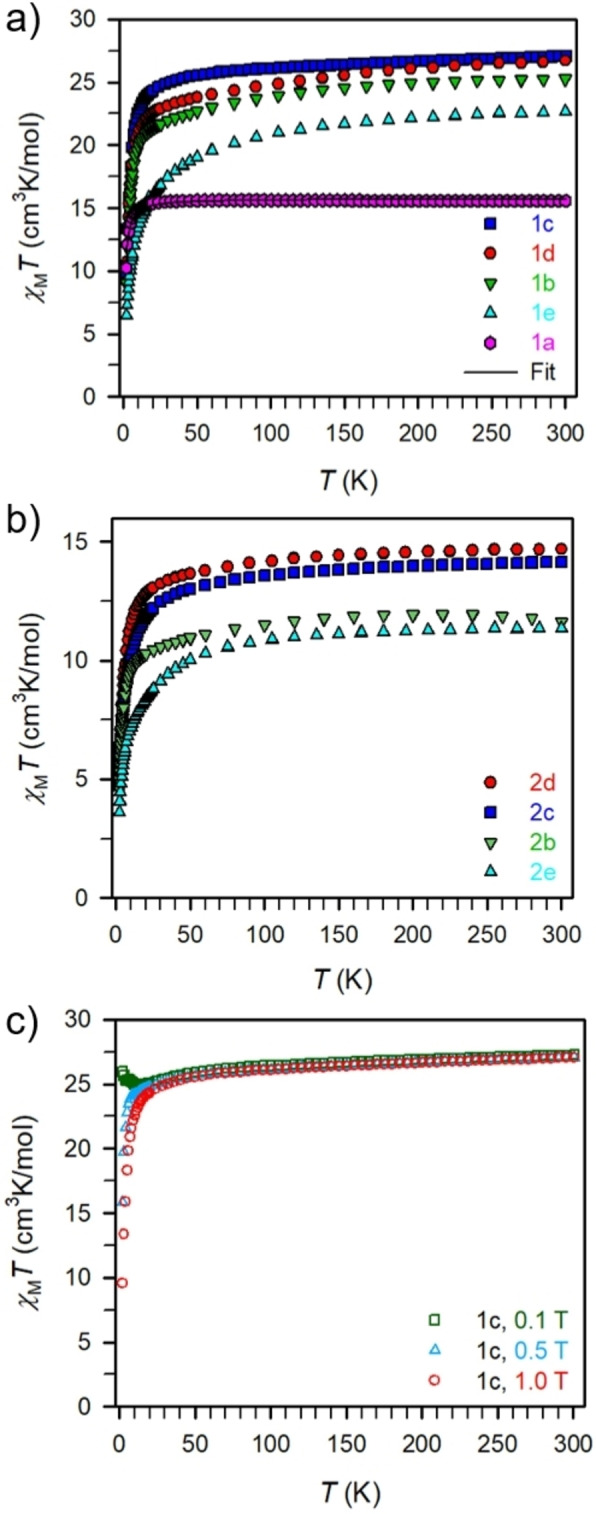
Variable‐temperature dc magnetic susceptibility data for restrained polycrystalline samples of a) **1a** (pink, Gd), **1b** (green, Tb), 1c (blue, Dy), **1d** (red, Ho), and **1e** (cyan, Er), b) **2b** (green, Tb), **2c** (blue, Dy), **2d** (red, Ho), and **2e** (cyan, Er), collected under a 1 T applied dc field. The black line represents a fit to the data for **1a**, as discussed in the main text. c) Variable‐temperature dc magnetic susceptibility data for restrained polycrystalline samples of **1c** collected under a 0.1 T (green squares), 0.5 T (light blue triangles), and 1 T (red circles) applied dc field.

Variable‐temperature dc magnetic susceptibility data were collected for **1 a**–**e** and **2 b**–**c** in the temperature range 2–300 K at 0.1, 0.5 and 1 T fields (Figures 4a) and b), and S12‐S51). The discussion will focus on the data collected at 1 T and bimetallic complexes followed by the monometallic complexes. The room temperature *χ*_M_ *T* values (15.56, 25.27, 27.06, 26.71, and 22.67 cm^3^K/mol for **1 a**–**e**, respectively) are in good agreement with the expected values (15.76, 23.62, 28.34, 28.12, and 22.96 cm^3^K/mol, respectively) for two non‐interacting triply positive lanthanide atoms. As the temperature is decreased to 15 K, *χ*_M_ *T* undergoes a gradual decline and decreases below that temperature rapidly to reach at 2 K a minimum value of 10.24, 8.99, 9.57, 9.14, and 6.46 cm^3^K/mol for **1 a**–**e**, respectively. Such downturn in *χ*_M_ *T* can be generally assigned to the Zeeman effect and/or weak intermolecular interactions. Notably, the decline in *χ*_M_ *T* is much more pronounced for **1 e** and can potentially be ascribed to quite anisotropic trivalent erbium atoms in this ligand environment compared to the lighter lanthanide analogs. By contrast, *χ*_M_ *T* only decreases more noticeably below 10 K for **1 a** hinting at the presence of very weak antiferromagnetic coupling. Due to the half‐filled f‐electron valence shell (4f^7^), trivalent gadolinium ions offer the possibility to determine the magnetic exchange interaction precisely. Fitting the dc data of **1 a** to a spin‐only Hamiltonian *Ĥ*=−2*JŜ*_Gd(1)_+−2*JŜ*_Gd(2)_ where *J* is the intramolecular coupling constant and *Ŝ* the spin operator for each paramagnetic center yielded a coupling constant of *J*=−0.045(1) cm^−1^ which is indicative of weak antiferromagnetic interaction between the Gd^III^ ions. The magnitude of the coupling between the lanthanide ions via superexchange is typically very weak as is the case for the parent compound **1 a** and comparable to various chloride‐bridged complexes or other bridges leading to superexchange pathways in bimetallic gadolinium complexes.[^25^, ^26^, ^27^, ^28^, ^29^] The resembling progression of the temperature dependence of the product of magnetic susceptibility and temperature, *χ*_M_ *T*, suggests that the coupling between the lanthanide ions based on a superexchange mechanism is similarly very weak. This is expected as the lanthanides possess deeply buried 4 f orbitals rendering superexchange as magnetic communication path very weak. One powerful method to raise the coupling between lanthanide ions is through implementation of spin‐carriers such as inorganic or organic radical ligands with diffuse orbitals that allow penetration of the 4 f‐orbitals.[^15^, ^16^, ^25^, ^26^, ^27^, ^28^, ^29^, ^30^] The room temperature *χ*_M_ *T* values of 11.62, 14.15, 14.69, and 11.37 cm^3^K/mol for **2 b**–**e** are slightly lower than the expected values of 11.81, 14.17, 14.06, and 11.48 for the respective free triply positive lanthanide ions (Figure 4b). With decreasing temperature, a gradual decline in *χ*_M_ *T* occurs for **2 b**–**e** and culminates in minima of 4.61, 4.72, 4.95, and 3.60 cm^3^K/mol at 2 K. Noteworthy, there is no plummeting behavior of *χ*_M_ *T* at the lowest temperatures observed such as for mononuclear lanthanide complexes containing phthalocyanine or cyclopentadienyl‐based ligands including the class of dysprosocenium compounds with record high magnetic blocking temperatures.[^31^, ^32^, ^33^] Thus, the tetraimido sulfate ligand scaffold represents neither a strictly axial nor equatorial ligand field to advance the single‐ion anisotropy of the employed lanthanide ions. Akin to the bimetallic complexes, the steeper decrease in *χ*_M_ *T* below 20 K can be ascribed to the Zeeman effect and/or weak intermolecular interactions. To probe this, variable‐field variable temperature magnetic susceptibility measurements performed on **1 a**–**e** and **2 b**–**d** indicate this downturn to be in virtue of the Zeeman effect (Figure 4c).

### Dynamic magnetic susceptibility measurements

**Figure 5:**
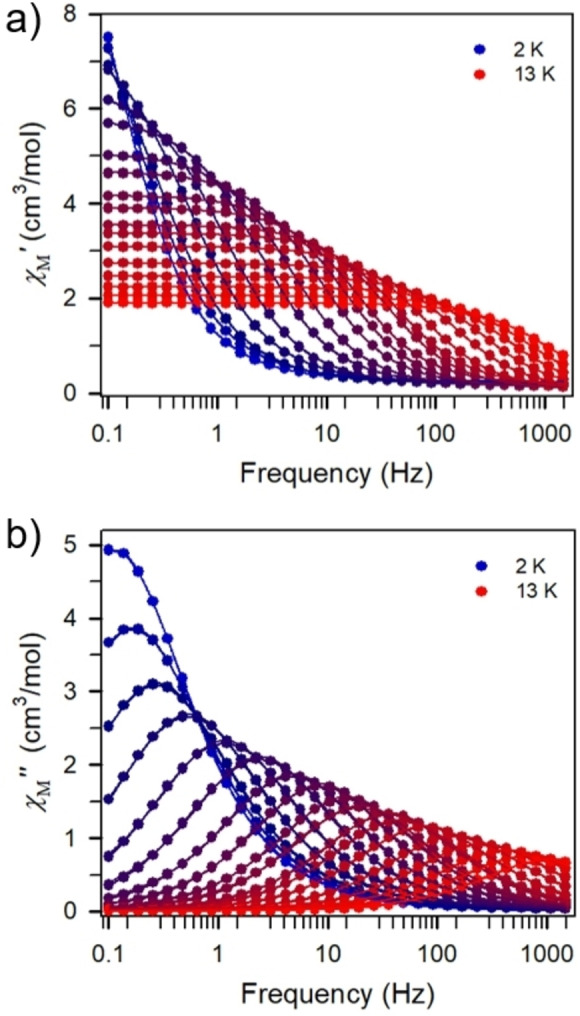
Variable‐temperature, variable‐frequency in‐phase (*χ*_M_′, a) and out‐of‐phase (*χ*_M_′′, b) ac magnetic susceptibility data collected for **1c** under zero applied dc field from 2 K (dark blue circles) to 13 K (red circles). Solid lines represent a fit to the data.

**Figure 6:**
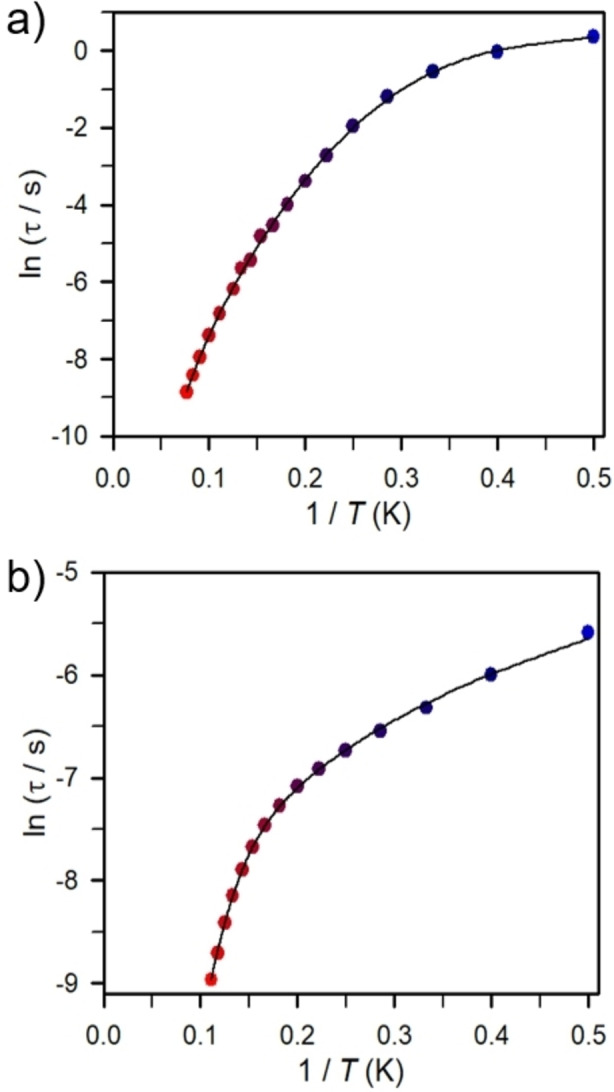
Arrhenius plot of relaxation time data for a) **1c** under zero dc field from 2 K (dark blue circles) to 13 K (red circles) and b) **2b** under 2000 Oe field from 2 K (dark blue circles) to 9 K (red circles). Solid lines represent a fit to the data as described in the main text.

Due to the large magnetic anisotropy inherent to Ln^III^=Tb^III^, Dy^III^, Ho^III^, Er^III^ ions, it was anticipated that single‐molecule magnet behavior could arise. The magnetic relaxation dynamics of **1 b**–**e** and **2 b**–**e**, respectively, were probed through variable‐frequency variable‐temperature ac magnetic susceptibility measurements under zero and applied dc fields up to 3000 Oe. Compounds **1 b**, **1 d**, **1 e**, **2 d**, and **2 e** did not show any out‐of‐phase (*χ*_M_”) signals between 0.1 and 1000 Hz. By contrast, the bimetallic dysprosium complex, **1 c**, showed in‐phase (*χ*_M_ *’*) and out‐of‐phase (*χ*_M_”) signals that suggest long magnetic relaxation times and are indicative of single‐molecule magnet behavior (Figure 5). At 2 K, **1 c** exhibited a peak maximum at 0.1 Hz that shifted to higher frequencies as the temperature was risen until it shifted beyond the frequency limit of 1488 Hz of the magnetometer at 13 K. The ac data collected were used to generate Cole‐Cole plots at each temperature within the range of 2 to 13 K (Figure S52). These plots were fitted to a generalized Debye model to extract the relaxation times, *τ*, which were employed to construct the Arrhenius plot in Figure 6a). Temperature‐dependent relaxation times provide insight into the operative magnetic relaxation processes at certain temperatures. A plot of the natural log of *τ* versus 1/T showing linearity at the highest measured temperatures is suggestive of an Orbach relaxation process that ultimately describes a thermally activated spin‐reversal over a potential barrier.^[34]^ This type of relaxation shows an exponential temperature dependence and is defined with the Arrhenius expression *τ=τ*_0_⋅exp(*U*_eff_/k_B_ *T*). The Arrhenius plot shows a curvature of ln *τ* indicating that other relaxation processes with variable temperature‐dependences are apparent. Hence, the Arrhenius plot was fit to multiple relaxation processes allowing for the relaxation barrier to be determined accurately (see Figure 6a) and Figure S53 in Supporting Information). To satisfactorily model the data for **1 c**, the fit required Orbach, Raman and Quantum tunneling processes [Eq. 1]:

(1) $$\frac{1}{\tau_{obs}}=\frac{1}{\tau_{QTM}}+CT^{n}+\tau_{0}^{-1}exp\left(-U_{eff}/k_{B}T\right)$$

Here, the first term is from the tunneling pathway, the second is for the Raman process, and the third term models the Orbach relaxation pathways. The obtained values of spin‐reversal barrier, *U*_eff_, and attempt time, *τ*_0_, are 28.0(2) cm^−1^ and 5.7(1)×10^−5^ s. For comparison, fitting only the high temperature data to the Arrhenius expression (Figure S61) yields a higher barrier of *U*_eff_=43.9(8) cm^−1^ and *τ*_0_=1.1(1)×10^−6^ s, which is likely closer to the actual barrier height as an Orbach process gives rise to an exponential dependence of *τ* upon temperature. Notably, a satisfactorily fit was also achieved by employing only Quantum tunneling and Raman relaxation processes potentially suggesting that the relaxation barrier obtained is underestimated by considering a QTM, Raman and Orbach Process (Figures S54–55). Since the peaks are moving beyond the frequency limit of a SQUID magnetometer, more insight into the Orbach process remains currently elusive. Application of a 250 Oe dc field leads to out‐of‐phase signals that are observed up to 15 K resulting in a slightly higher spin‐reversal barrier *U*_eff_=54.4(1) cm^−1^ and *τ*_0_=4.7(1)×10^−7^ s (Figures S60, S68–69). Applied dc fields within the range of 200 to 2000 Oe altered the appearance of the χ_M_” peak shape (Figure S65). With increasing dc fields, a slight decline in the intensities of the peaks, concomitant with a shift to lower frequencies occurred. The change of the peak saturates at 500 Oe dc field as above that temperature the peak positions are frequency invariant, and the intensities decrease more rapidly. Thus, variable‐temperature, variable‐frequency in‐phase (χ_M_′, a) and out‐of‐phase (χ_M_”, b) ac magnetic susceptibility data collected for **1 c** under 500 Oe applied dc field indicated a slightly stronger temperature‐dependence contrasted to the zero‐field data (Figures S66–67). The resulting relaxation times were employed to generate the Arrhenius plot. Similarly, to the zero‐field data, the relaxation time were fit to multiple relaxation processes (Figure S56–59). A fit to a an Orbach and Raman relaxation process afforded higher values for the spin‐reversal barrier *U*_eff_=64.9(1) cm^−1^, and attempt time, *τ*_0_=5.2(8)×10^−7^ s (Figures S56–57). The inclusion of a quantum tunneling and direct process, respectively, did not improve the quality of the fit. Akin to the relaxation times extracted from the zero field measurements, the data obtained at 500 Oe may also be described by considering only one Raman relaxation process leading to comparable values of *C*=0.00087(8) (s^−1^K^−n^) and n=6.364(1) (Tables S29 and S30, Figures S58–S59). Barrier heights on the order of 20–60 cm^−1^ are typical for bimetallic superexchange‐coupled dysprosium complexes comprising metal ions with high coordination numbers.^[35]^

**Figure 7:**
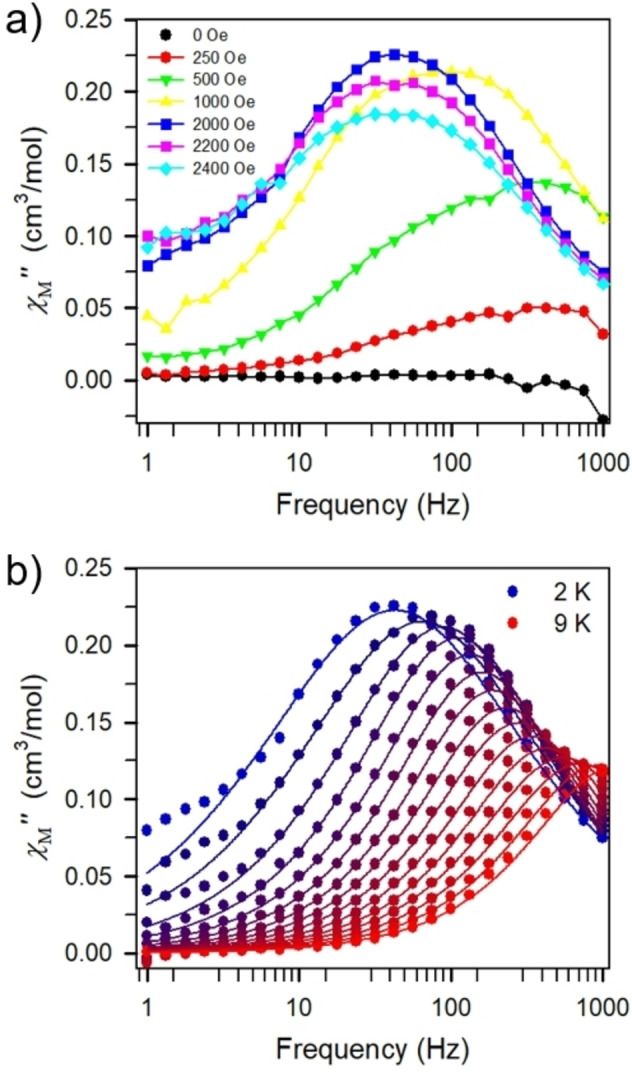
a) Out‐of‐phase ac susceptibility (*χ*_M_”) collected on pure **2b** at 2 K under dc fields ranging from 0 Oe to 2400 Oe. Solid lines are guides for the eye. b) Variable‐frequency out‐of‐phase (*χ*_M_”) ac magnetic susceptibility data collected for **2b** under 2000 Oe applied dc field from 2 K (dark blue circles) to 9 K (red circles). Solid lines represent a fit to the data.

The mononuclear complexes **2 d**–**e** did not show single‐molecule magnet behavior under zero and applied dc fields, respectively. By contrast, in the absence of a dc field, **2 b** and **2 c** lack slow magnetic relaxation, however, when subjected to dc fields out‐of‐phase (χ_M_”) signals were observed. The optimal dc fields were determined to be 2000 Oe and 500 Oe for **2 b** and **2 c**, respectively (Figure 7a) and Figure S78). Under 2000 Oe dc field and within the frequency range 1 and 1000 Hz, a temperature‐dependent out‐of‐phase (χ_M_”) peak was observed for **2 b** between 2 and 9 K (Figures 7b) and S73–74). The extracted relaxation times could be adequately fit to a Direct, Raman and Orbach relaxation process yielding a barrier to spin relaxation of *U*_eff_=42.8(6) cm^−1^ and *τ*_0_=2.2(4)×10^−6^ s (Figure 6b) and Figure S75). In contrast, **2 c** exhibits slow magnetic relaxation within the temperature range 1.8 and 2.4 K (Figures S79‐80). A linear fit to the extracted relaxation times afforded *U*_eff_=11.1(1) cm^−1^ and *τ*_0_=7.0(1)×10^−7^ s, indicative of a faster magnetic relaxation compared to the Dy dimer, **1 c**.

Variable‐field magnetization measurements were performed on all complexes comprising anisotropic metal centers, **1 b**–**d** and **2 b**–**d** to control for magnetic hysteresis. At 1.8 K, and an average sweep rate of 100 Oe/s, the hysteresis loop is closed at zero dc field and slightly open at higher fields for **1 c** (see Figure S70). For **1 b, 1 d, 1 e**, and **2 b–e**, there is neither remnant magnetization at zero dc field nor an open hysteresis loop at higher fields observed (see Figures S77, S82, S84). The low‐temperature magnetization data for the parent compounds **1 c** and **2 b**–**d** are shown in Figures S72, S76, S81, S83. At 2 K, the magnetization mounts quickly until about 2 T, gradually increases between 2 and 7 T, and does not fully saturate at a maximum field of 7 T for **1 c**, **2 c**, and **2 d**, whereas near full saturation is reached at 7 T for **2 b**. For an isolated ±*M_J_* =doublet, the saturation magnetization is calculated to be approximately 4.5 μ_*B*_ for Tb^III^ and *M_J_* =*J*=6. The experimentally determined maximum magnetization at 7 T of 5.07 μ_*B*_ indicates a *M_J_* =±6 doublet for the Tb^III^ ground state.

**Figure 8:**
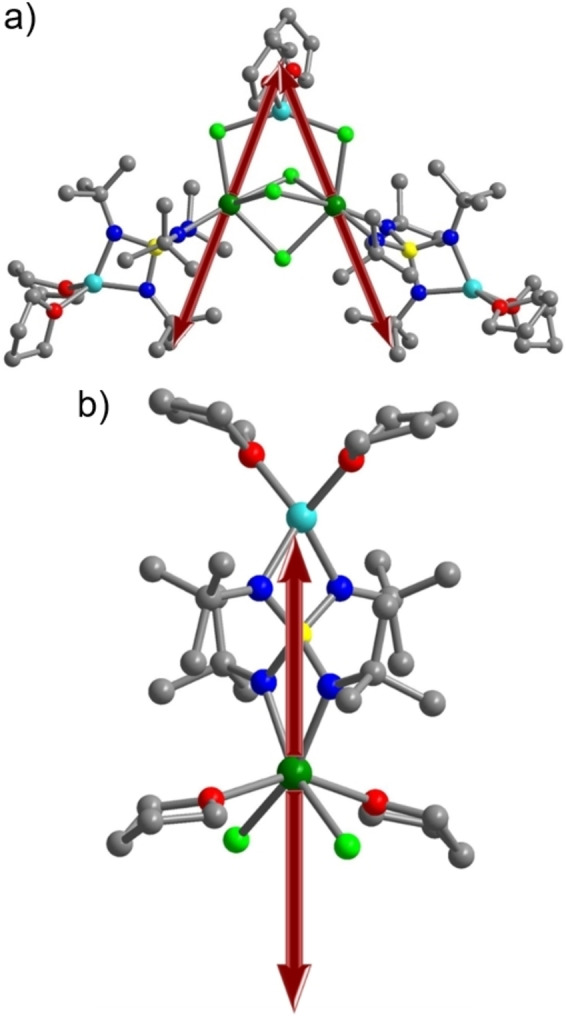
Orientation of the main anisotropy axis in **1 c** a) and **2 c** b). Coordinates are taken from the crystal structure depicted in Figures 1 and 2. Green, pale blue, pale green, blue, red, yellow, and gray spheres represent Dy, Li, N, O, S, and C atoms, respectively; hydrogen atoms are omitted for clarity.

A plausible explanation for the appearance of the much slower magnetic relaxation in **1 c** compared to the much faster magnetic relaxation in **2 c** is provided by the local geometry around each, six‐coordinate, dysprosium ion in **1 c** and **2 c**, respectively. The dysprosium ion features in the first coordination sphere four chlorine atoms and two nitrogen atoms in **1 c**, whereas **2 c** exhibits two chlorine atoms, two oxygen atoms and two nitrogen atoms. Each metal center can be approximated to form a distorted octahedron. In **1 c**, six of the N−Dy‐Cl angles vary between 100.5° and 108.5° with the two opposing donors to reach up to an angle as high as 168°. In **2 c**, the N−Dy−Cl and N−Dy−O angles cover a larger range from 89.61° to 103.9° with the opposite donors featuring the largest angle of 149°. The positioning of the ligands around each metal center suggests a higher magnetic axiality in **1 c** than in **2 c** (Figure 8). In addition, the presence of electronegative oxygen atoms bound to dysprosium introduces transverse magnetic anisotropy in **2 c** which promotes faster magnetic relaxation as has been demonstrated earlier in various dysprosium systems.^[36]^

The program Magellan was employed to both identify the apparent magnetic easy axis in the bimetallic Dy complex **1 c** and to extract information regarding the existence of an easy axis in the Dy monomer **2 c**.^[37]^ The computational result for **1 c** suggests that the axis of preferred orientation on each Dy ion extends between the Li cation bridging two Cl anions bound to the Dy atoms and the most distant *t*Bu group. A similar orientation of the axis toward halides and alkali metal ions has been determined by ab initio calculations for other multimetallic Dy complexes where the dysprosium ions are bridged by chloride ions, giving rise to a *M*_J_=±15/2 ground state.[^26^, ^38^] In a bromide‐bridged bimetallic dysprosium complex the anisotropy axis can, however, favor an orientation towards coordinating tricyclohexylphosphine oxide ligands as opposed to bromide ions.^[25]^

The computational result for **2 c** indicates a preferred alignment of the axis that extends between the two chloride ions and the nitrogen atoms of the S(N*t*Bu)_4_^2−^ ligand where all four exhibit an almost planar arrangement with Cl−Dy−N−S torsion angles of 176.9° and 177.7°. Thus, the preferred alignment of the axis is drastically different compared to **1 c**. The quadrupole approximations of the 4 f shell electron distribution for the tri‐positive Dy^III^ and Tb^III^ ions lead to a similar description of the overall shape of the free‐ion electron density which is oblate.^[39]^ Consequently, the computational results obtained for **2 c** are expected to be transferrable to **2 b**. Oblate‐shaped lanthanide ions such as Tb^III^ and Dy^III^ require axial ligand fields to enhance magnetic anisotropy needed to promote slower magnetic relaxation as has been demonstrated in several mononuclear lanthanide complexes.[^31^, ^33^, ^40^] To boost the single‐ion anisotropy of prolate ions such as Er^III^, an equatorial ligand field is needed as proven in mononuclear Er‐based complexes.^[41]^ Visually non‐axial ligand fields are present in complexes **1 b**, **1 c**, **2 b** and **2 c**. The only complex displaying true slow magnetic relaxation at zero field is the bimetallic Dy complex **1 c** which can be largely ascribed to the Kramers ion nature of the Dy^III^ ion that guarantees a doubly degenerate *m*_J_ ground state which is optimal for single‐molecule magnet design. Furthermore, the Dy^III^ ion merges significant 4 f shell anisotropy with the large‐moment ^6^H_15/2_ ground state. Indeed, the observation of slow magnetic relaxation is due to the single‐ion effect as the magnetic communication between the dysprosium ions is negligible due to both their deeply buried 4 f‐orbitals and a rather large intramolecular Dy⋅⋅⋅Dy distance of 3.8 Å. This interpretation is based off the similar trend in the temperature dependence of the product of magnetic susceptibility and temperature for all bimetallic Ln complexes **1 a**–**e** and the quantified exchange coupling constant of *J*=−0.045(1) cm^−1^ for **1 a** which is indicative of very weak antiferromagnetic coupling between the metal centers. The lack of slow magnetic relaxation in zero dc field for the respective mononuclear Dy complex **2 c** is substantiated by low‐symmetry components of the ligand field that are primary initiators for quantum tunneling of the relaxation or other non‐thermally activated spin‐lattice relaxation processes. The computational result gained from Magellan hints at the two coordinating THF molecules, representing a moderately strong ligand field, interact with the metal center in the hard plane which is generally where quantum tunneling of the magnetization is promoted the most. An akin interpretation justifies the observation of field‐induced single‐molecule magnet behavior for the mononuclear Tb complex, **2 b**. Suppression of the quantum tunneling process to some degree by application of external dc fields invokes field‐induced single‐molecule magnet behavior for **2 b** and **2 c**. Evidently, the ligand field in the remaining isostructural monomeric and dimeric complexes is not the correct one to enhance their single‐ion anisotropy causing those not to be single‐molecule magnets.

## Conclusion

The readily synthesis of the first series of lanthanide complexes containing the tetraimido sulfate ligand renders these compounds compelling to study further for the potential utilization in areas such as high‐density information storage, molecular electronics, and magnetic refrigeration. Three of the investigated systems show single‐molecule magnet behavior where slow magnetic relaxation is observed under zero and applied dc fields, respectively. The respective dinuclear dysprosium congener is the most promising candidate across the series with long relaxation times between 2 and 13 K in the absence of a dc field. The determined barrier to spin‐relaxation is high as *U*_eff_=64.9(1) cm^−1^. Although this barrier height falls short compared to other dinuclear dysprosium single‐molecule magnets, the compound holds great promise to be an intermediate to a higher blocking system. Functionalization of the ligand scaffold and geometry optimizations can be envisioned. Removal of chloride ions with the quest of attaining a lower‐coordinate dysprosium ion to mitigate transverse anisotropy are worth pursuing. Strategies to increase the magnetic communication between metal centers are also underway. By contrast, the mononuclear dysprosium and terbium complexes relax slowly under dc fields. To suppress quantum tunneling of the magnetization that is the primary reason of lack of single‐molecule magnet behavior in the latter compounds, efforts are ongoing to increase a higher axial symmetry with no low‐symmetry elements to meet the demand of oblate trivalent lanthanide ions. In summary, both the dysprosium complexes and the mononuclear terbium complex are promising candidates to serve as the foundation for higher blocking single‐molecule magnets.

## Experimental Section

All experiments were performed under inert gas conditions in N_2_ or Ar using Schlenk techniques or in an Ar glovebox. Solvents were dried over sodium or potassium; distilled prior to use and stored over molecular sieves (3 Å). Starting materials were commercially purchased and used without further purification. [(thf)_4_Li_2_(N*t*Bu)_4_S] was synthesized according to literature known procedure.^[10]^ Elemental analysis (C, H, N, S) were performed at the Analytische Labor, Institut für Anorganische Chemie, University of Göttingen.

**General synthesis for [{(thf)_2_Li(N*t*Bu)_2_S(*t*BuN)_2_LnCl_2_}_2_** **⋅ ClLi(thf)_2_] 1 a**–**e**: A mixture of [(thf)_4_Li_2_(N*t*Bu)_4_S] (500.0 mg, 0.8080 mmol) and LnCl_3_ (0.8080 mmol) is dissolved in thf (20 mL) at ambient temperature. After stirring for 1 d the reaction mixture is concentrated under reduced pressure (7 mL), filtered and subsequently, the solvent is removed under reduced pressure. The residue is dissolved in toluene (5 mL) and the solution is filtered. Crystal growth of **1 a**–**e** starts within several minutes to hours at ambient temperature. For a complete crystallization, the mixture is stored at −34 °C yielding colorless crystals suitable for X‐ray analysis after several days. The solvent is removed, and the crystalline product is washed with *n‐*pentane (2×2 mL). **1 a**: Yield: 266.9 mg (40 %); elemental analysis calcd (%) for C_56_H_120_Cl_5_Gd_2_Li_3_N_8_O_6_S_2_(C_7_H_8_): C 45.30, H 7.72, N 6.71, S 3.84; found: C 42.91, H 6.92, N 6.51, S 4.47. **1 b**: Yield: 304.6 mg (45 %); elemental analysis calcd (%) for C_56_H_120_Cl_5_Tb_2_Li_3_N_8_O_6_ S_2_(C_7_H_8_): C 45.21, H 7.71, N 6.69, S 3.84; found: C 43.64, H 7.87, N 6.88, S 4.80. **1 c**: Yield: 281.5 mg (41 %); elemental analysis calcd (%) for C_56_H_120_Cl_5_Dy_2_Li_3_N_8_O_6_S_2_(C_7_H_8_): C 45.02, H 7.68, N 6.67, S 3.91; found: C 42.63, H 7.62, N 6.61, S 4.14. **1 d**: Yield: 137.1 mg (20 %); elemental analysis calcd (%) for C_56_H_120_Cl_5_Ho_2_Li_3_N_8_O_6_S_2_(C_7_H_8_): C 44.89, H 7.65, N 6.65, S 3.80; found: C 41.27, H 7.06, N 6.71, S 3.92. **1 e**: Yield: 113.8 mg (17 %); elemental analysis calcd (%) for C_56_H_120_Cl_5_Er_2_Li_3_N_8_O_6_S_2_(C_7_H_8_): C 44.76, H 7.60, N 6.63, S 3.79; found: C 42.67, H 7.60, N 7.14, S 5.08.

**General synthesis for [{(thf)_2_Li(N*t*Bu)_2_S(*t*BuN)_2_LnCl_2_(thf)_2_] 2 a**–**e**: [{(thf)_2_Li(N*t*Bu)_2_S(*t*BuN)_2_LnCl_2_}_2_ ⋅ ClLi(thf)_2_] (**1 a**–**e**) (150.0 mg) is dissolved in thf (3 mL) and filtered. Crystallization of **2 a**–**e** starts within hours at ambient temperature where upon the mixture is stored at −34 °C to improve the yield. The target compound is isolated and washed with *n*‐pentane (2×1 mL) yielding crystals suitable for X‐ray analysis. **2 a**: color: colorless; Yield: 98.3 mg (65 %); elemental analysis calcd (%) for C_32_H_68_Cl_2_GdLiN_4_O_4_S: C 45.75, H 8.16, N 6.67, S 3.82; found: C 45.82, H 8.59, N 6.54, S 4.01. **2 b**: color: colorless; Yield: 99.6 mg (66 %); elemental analysis calcd (%) for C_32_H_68_Cl_2_TbLiN_4_O_4_S: C 45.66, H 8.14, N 6.66, S 3.81; found: C 45.98, H 8.66, N 6.50, S 4.34. **2 c**: color: colorless; Yield: 105.2 mg (70 %); elemental analysis calcd (%) for C_32_H_68_Cl_2_DyLiN_4_O_4_S: C 45.47, H 8.11, N 6.63, S 3.79; found: C 46.58, H 8.71, N 6.44, S 4.77. **2 d**: color: pale orange; Yield: 115.5 mg (77 %); elemental analysis calcd (%) for C_32_H_68_Cl_2_HoLiN_4_O_4_S: C 45.34, H 8.09, N 6.61, S 3.78; found: C 45.89, H 8.50, N 6.38, S 3.90. **2 e**: color: light pink; here, only 100.0 mg of **2 e** were used (**1 e**); Yield: 74.6 mg (74 %); elemental analysis calcd (%) for C_32_H_68_Cl_2_ErLiN_4_O_4_S: C 45.21, H 8.06, N 6.59, S 3.77; found: C 45.55, H 8.61, N 6.35, S 4.13.

**Crystallographic data**: Single crystals were selected under cooling using the X‐Temp2 device.^[42]^ The datasets were collected on an Incoatec Ag Microsource^[43]^ with mirror optics and an APEX II detector with a D8 goniometer. The data were integrated with SAINT.^[44]^ A multi‐scan absorption correction was applied using SADABS.^[45]^ The structures were solved by SHELXT^[46]^ and refined on F^2^ using SHELXL^[47]^ in the graphical user interface ShelXle.^[48]^ Full crystallographic data is available in the Supporting Information. Deposition numbers 2069109 (**1 a**); 2069110 (**1 b**), 2069111 (**1 c**), 2069112 (**1 d**), 2069113 (**1 e**), 2069114 (**2 a**); 2069115 (**2 b**), 2069116 (**2 c**), 2069117 (**2 d**), 2069118 (**2 e**), 2069119 (**3 b**) contain the supplementary crystallographic data for this paper. These data are provided free of charge by the joint Cambridge Crystallographic Data Centre and Fachinformationszentrum Karlsruhe Access Structures service.

**Magnetic susceptibility measurements**: The magnetic samples of [{(thf)_2_Li(N*t*Bu)_2_S(*t*BuN)_2_LnCl_2_}_2_ ⋅ ClLi(thf)_2_] **1 a**–**e**, with **a**: Ln=Gd, **b**: Tb, **c**: Dy, **d**: Ho, **e**: Er and species [{(thf)_2_Li(N*t*Bu)_2_S (*t*BuN)_2_LnCl_2_(thf)_2_] **2 a**–**e** (**a**: Ln=Gd, **b**: Tb, **c**: Dy, **d**: Ho, **e**: Er) were prepared by loading crushed crystalline samples into tubes in an argon glove‐box. Sufficient liquid eicosane (at 60 °C) was added to saturate and cover the samples to prevent crystallite torquing and provide good thermal contact between the sample and the bath. Tubes were sealed air‐tight before transferred to the magnetometer. Magnetic susceptibility measurements were collected using a Quantum Design MPMSXL SQUID magnetometer and Quantum Design MPMS3 SQUID magnetometer, respectively. DC susceptibility data measurements were performed at temperatures ranging from 2 to 300 K for **1 a**–**e** and **2 b**–**e**, using applied fields of 0.1, 0.5 and 1 T. Ac magnetic susceptibility data measurements were performed using a 3.6 (MPMSXL) and 4 Oe (MPMS3) switching field, respectively. All data were corrected for diamagnetic contributions from the eicosane and core diamagnetism estimated using Pascal's constants.^[49]^ Cole‐Cole plots were fitted using formulae describing *χ*′ and *χ*′′ in terms of frequency, constant temperature susceptibility (*χ*_T_), adiabatic susceptibility (*χ*_S_), relaxation time (*τ*), and a variable representing the distribution of relaxation times (*α*).^[50]^

## Conflict of interest

The authors declare no conflict of interest.

## Supporting information

As a service to our authors and readers, this journal provides supporting information supplied by the authors. Such materials are peer reviewed and may be re‐organized for online delivery, but are not copy‐edited or typeset. Technical support issues arising from supporting information (other than missing files) should be addressed to the authors.

Supporting Information
