# Supplementary material for: Slow Magnetic Relaxation in Mono‐ and Bimetallic Lanthanide Tetraimido‐Sulfate S(NtBu)4 2− Complexes
Source: Chemistry. 2021 Jun 24;27(48):12310–9. doi: 10.1002/chem.202101076 (PMC8453918; doi:10.1002/chem.202101076)
Supplement: Supplementary file 1 — Supporting Information [file CHEM-27-12310-s001.pdf]

Chemistry–A European Journal

Supporting Information

Slow Magnetic Relaxation in Mono- and Bimetallic Lanthanide Tetraimido-Sulfate $S(NtBu)_4^{2-}$ Complexes

Jochen Jung, Florian Benner, Regine Herbst-Irmer, Selvan Demir,* and Dietmar Stalke*

Table of Contents

Crystallography S7

XRD-Analysis of 1a **S9**

XRD-Analysis of 1b **S12**

XRD-Analysis of 1c **S15**

XRD-Analysis of 1d **S18**

XRD-Analysis of 1e **S21**

XRD-Analysis of 2a **S24**

XRD-Analysis of 2b **S27**

XRD-Analysis of 2c **S30**

XRD-Analysis of 2d **S33**

XRD-Analysis of 2e **S36**

XRD-Analysis of 3b **S39**

Magnetic Measurement Details. S45

Arrhenius Plot Fitting Details. **S45**

Table S26. Best-fit parameters for the Arrhenius plots of **1c** from 2 to 13 K. Equation 1 was used. **S47**

Table S27. Best-fit parameters for the Arrhenius plots of **1c** from 2 to 13 K. Equation 2 was used. **S47**

Table S28. Best-fit parameters for the Arrhenius plot of **1c** from 7 to 13 and 15 K. **S47**

Table S29. Best-fit parameters for the Arrhenius plot of **1c** from 2 to 12 K. Equation 3 was used. **S47**

Table S30. Best-fit parameters for the Arrhenius plot of **1c** from 2 to 12 K. **S47**

Table S31. Best-fit parameters for the Arrhenius plots of **2b** from 2 to 9 K. Equation 4 was used. **S47**

Figure S12. Variable-temperature dc magnetic susceptibility data for **1a** under a 0.1 T, 0.5 T, and 1 T applied dc field. **S48**

Figure S13. Variable-temperature dc magnetic susceptibility data for **1a** under a 0.1 T applied dc field. **S48**

Figure S14. Variable-temperature dc magnetic susceptibility data for **1a** under a 0.5 T applied dc field. **S49**

Figure S15. Variable-temperature dc magnetic susceptibility data for **1a** under a 1 T applied dc field. **S49**

Figure S16. Variable-temperature dc magnetic susceptibility data for **1b** under a 0.1 T, 0.5 T, and 1 T applied dc field. **S50**

Figure S17. Variable-temperature dc magnetic susceptibility data for **1b** under a 750 Oe applied dc field. **S50**

Figure S18. Variable-temperature dc magnetic susceptibility data for **1b** under a 0.1 T applied dc field. **S51**

Figure S19. Variable-temperature dc magnetic susceptibility data for **1b** under a 0.5 T applied dc field. **S51**

Figure S20. Variable-temperature dc magnetic susceptibility data for **1b** under a 1 T applied dc field. **S52**

Figure S21. Variable-temperature dc magnetic susceptibility data for **1c** under a 0.1 T applied dc field. **S52**

Figure S22. Variable-temperature dc magnetic susceptibility data for **1c** under a 0.5 T applied dc field. **S53**

Figure S23. Variable-temperature dc magnetic susceptibility data for **1c** under a 1 T applied dc field. **S53**

Figure S24. Variable-temperature dc magnetic susceptibility data for **1d** under a 0.1 T, 0.5 T, and 1 T applied dc field. **S54**

Figure S25. Variable-temperature dc magnetic susceptibility data for **1d** under a 0.1 T applied dc field. **S54**

Figure S26. Variable-temperature dc magnetic susceptibility data for **1d** under a 0.5 T applied dc field. **S55**

Figure S27. Variable-temperature dc magnetic susceptibility data for **1d** under a 1 T applied dc field. **S55**

Figure S28. Variable-temperature dc magnetic susceptibility data for **1e** under a 0.1 T, 0.5 T, and 1 T applied dc field. **S56**

Figure S29. Variable-temperature dc magnetic susceptibility data for **1e** under a 0.1 T applied dc field. **S56**

Figure S30. Variable-temperature dc magnetic susceptibility data for **1e** under a 0.5 T applied dc field. **S57**

Figure S31. Variable-temperature dc magnetic susceptibility data for **1e** under a 1 T applied dc field. **S57**

Figure S32. Variable-temperature dc magnetic susceptibility data for **1a**, **1b**, **1c**, **1d**, and **1e** under a 0.1 T applied dc field. **S58**

Figure S33. Variable-temperature dc magnetic susceptibility data for **1a**, **1b**, **1c**, **1d**, and **1e** under a 0.5 T applied dc field. **S59**

Figure S34. Variable-temperature dc magnetic susceptibility data for **1a**, **1b**, **1c**, **1d**, and **1e** under a 1 T applied dc field. **S60**

Figure S35. Variable-temperature dc magnetic susceptibility data for **2b** under a 0.1 T, 0.5 T, and 1 T applied dc field. **S61**

Figure S36. Variable-temperature dc magnetic susceptibility data for **2b** under a 0.1 T applied dc field. **S61**

Figure S37. Variable-temperature dc magnetic susceptibility data for **2b** under a 0.5 T applied dc field. **S62**

Figure S38. Variable-temperature dc magnetic susceptibility data for **2b** under a 1 T applied dc field. **S62**

Figure S39. Variable-temperature dc magnetic susceptibility data for **2c** under a 0.1 T applied dc field. **S63**

Figure S40. Variable-temperature dc magnetic susceptibility data for **2c** under a 0.5 T applied dc field. **S63**

Figure S41. Variable-temperature dc magnetic susceptibility data for **2c** under a 1 T applied dc field. **S64**

Figure S42. Variable-temperature dc magnetic susceptibility data for **2d** under a 0.1 T, 0.5 T, and 1 T applied dc field. **S64**

Figure S43. Variable-temperature dc magnetic susceptibility data for **2d** under a 0.1 T applied dc field. **S65**

Figure S44. Variable-temperature dc magnetic susceptibility data for **2d** under a 0.5 T applied dc field. **S65**

Figure S45. Variable-temperature dc magnetic susceptibility data for **2d** under a 1 T applied dc field. **S66**

Figure S46. Variable-temperature dc magnetic susceptibility data for **2e** under a 0.1 T, 0.5 T, and 1 T applied dc field. **S66**

Figure S47. Variable-temperature dc magnetic susceptibility data for **2e** under a 0.1 T applied dc field. **S67**

Figure S48. Variable-temperature dc magnetic susceptibility data for **2e** under a 0.5 T applied dc field. **S67**

Figure S49. Variable-temperature dc magnetic susceptibility data for **2e** under a 1 T applied dc field. **S68**

Figure S50. Variable-temperature dc magnetic susceptibility data for **2b**, **2c**, **2d**, and **2e** under a 0.1 T applied dc field. **S68**

Figure S51. Variable-temperature dc magnetic susceptibility data for **2b**, **2d**, and **2e** under a 0.5 T applied dc field. **S69**

Figure S52. Cole-Cole (Argand) plots for **1c** at H_{dc} = 0 Oe from 2 to 13 K. **S69**

Figure S53. Individual contributions of the multiple magnetic relaxation pathways to the Arrhenius plot of **1c** at 0 Oe from 2 K to 13 K. **S70**

Figure S54. Arrhenius plot of relaxation time data for **1c** at zero dc field from 2 K to 13 K (red circles). (see Figure 44). **S70**

Figure S55. Individual contributions of the multiple magnetic relaxation pathways to the Arrhenius plots of **1c** at 0 Oe (see Figure 43). **S71**

Figure S56. Arrhenius plot of relaxation time data for **1c** at 500 Oe dc field from 3 K to 12 K (red circles). (see Figure S46 and Table S4). **S71**

Figure S57. Individual contributions of the multiple magnetic relaxation pathways to the Arrhenius plots of **1c** at 500 Oe. (see Figure 45 and Table S4). **S72**

Figure S58. Arrhenius plot of relaxation time data for **1c** at 500 Oe dc field from 3 to 12 K. (see Figure S48 and Table S5). **S72**

Figure S59. Individual contributions of the multiple magnetic relaxation pathways to the Arrhenius plots of **1c** at 500 Oe (see Figure 47). **S73**

Figure S60. Arrhenius plot of relaxation time data for **1c** at 0 Oe and 500 Oe dc field. **S73**

Figure S61. Arrhenius plot of relaxation time data for **1c** at 0 Oe dc field from 2 K to 13 K. **S74**

Figure S62. Arrhenius plot of relaxation time data for **1c** at 500 Oe dc field from 3 K to 12 K. **S74**

Figure S63. Arrhenius plot of relaxation time data for **1c** at zero dc field from 7 K to 13 K. **S75**

Figure S64. Individual contributions of the multiple magnetic relaxation pathways to the Arrhenius plots of **1c** at 0 Oe from 7 K to 13 K. **S75**

Figure S65. Out-of-phase ac susceptibility (χ_M'') collected on pure **1c** at 3 K under dc fields ranging from 0 Oe to 2000 Oe. **S76**

Figure S66. Variable-temperature, variable-frequency in-phase (χ_M', top) and out-of-phase (χ_M'', bottom) ac magnetic susceptibility data collected for **1c** under 500 Oe applied dc field from 2 K to 12 K. **S77**

Figure S67. Cole-Cole (Argand) plots for ac susceptibility collected from 3 to 12 K under 500 Oe applied dc field for **1c**. **S78**

Figure S68. Arrhenius plot of relaxation time data for **1c** at 250 Oe dc field from 7 K to 15 K. **S78**

Figure S69 Individual contributions of the multiple magnetic relaxation pathways to the Arrhenius plotc of **1c** at 250 Oe from 7 K to 13 K. **S79**

Figure S70. Variable field magnetization (*M*) data for **1c** collected at 1.8 K. **S79**

Figure S71. Variable field magnetization (*M*) data for **1c** collected at 2 K. **S80**

Figure S72. Variable temperature *M*(*H*) curves for **1c** collected from 0 to 7 T. **S80**

Figure S73. Variable-temperature ac susceptibility for **2b** at H_{dc} = 2000 Oe. **S81**

Figure S74. Cole-Cole (Argand) plots for **2b** at H_{dc} = 2000 Oe from 2 to 9 K. **S82**

Figure S75. Individual contributions of the multiple magnetic relaxation pathways to the Arrhenius plot of **2b** at 2000 Oe from 2 K to 9 K. **S82**

Figure S76. Variable temperature *M*(*H*) curves for **2b** collected from 0 to 7 T. **S83**

Figure S77. Variable field magnetization (*M*) data for **2b** collected at 2 K. **S83**

Figure S78. Out-of-phase ac susceptibility (χ_M'') collected on pure **2c** at 1.8 K under dc fields ranging from 0 Oe to 1250 Oe. **S84**

Figure S79. Variable-temperature, variable-frequency in-phase (χ_M', top) and out-of-phase (χ_M'', bottom) ac magnetic susceptibility data collected for **2c** under 500 Oe applied dc field from 1.8 K to 2.4 K. **S85**

Figure S80. Cole-Cole (Argand) plots for **2c** at H_{dc} = 500 Oe from 1.8 to 2.4 K. **S86**

Figure S81. Variable temperature *M*(*H*) curves for **2c** collected from 0 to 5 T. **S86**

Figure S82. Variable field magnetization (*M*) data for **2c** collected at 1.8 K. **S87**

Figure S83. Variable temperature *M*(*H*) curves for **2d** collected from 0 to 5 T. **S87**

Figure S84. Variable field magnetization (*M*) data for **2d** collected at 2 K. **S88**

References.
S88Crystallography

Table S1 Crystallographic details for compounds **1a-e**.

compound	**1a**	**1b**	**1c**	**1d**	**1e**
CCDC no.					
Empirical formula	$C_{63}H_{128}Cl_5Gd_2Li_3N_8O_6S_2$	$C_{63}H_{128}Cl_5Tb_2Li_3N_8O_6S_2$	$C_{63}H_{128}Cl_5Dy_2Li_3N_8O_6S_2$	$C_{63}H_{128}Cl_5Ho_2Li_3N_8O_6S_2$	$C_{63}H_{128}Cl_5Er_2Li_3N_8O_6S_2$
Formula weight [g/mol]	1670.42	1673.76	1680.92	1685.78	1690.44
Temperature [K]	100(2)	100(2)	100(2)	100(2)	100(2)
Wavelength [Å]	0.56086	0.56086	0.56086	0.56086	0.56086
Crystal system	Monoclinic	Monoclinic	Monoclinic	Monoclinic	Monoclinic
Space group	C2/c	C2/c	C2/c	C2/c	C2/c
a [Å]	27.193(3)	27.151(3)	27.079(3)	26.992(3)	26.968(3)
b [Å]	18.003(2)	18.004(2)	17.966(2)	17.963(2)	17.954(2)
c [Å]	19.262(2)	19.231(2)	19.243(2)	19.258(2)	19.267(2)
β [°]	122.08(2)	122.08(2)	121.90(2)	121.82(2)	121.76(2)
Volume [$Å^3$]	7990(2)	7965(2)	7948(2)	7934(2)	7932(2)
Z	4	4	4	4	4
ρ_{calc} [g/cm^3]	1.389	1.396	1.405	1.411	1.416
μ [mm^{-1}]	1.023	1.081	1.142	1.200	1.271
F(000)	3456	3464	3472	3480	3488
θ range [°]	1.705 to 22.031	1.729 to 21.397	1.727 to 22.016	1.709 to 21.419	1.708 to 21.406
Reflections collected	121073	70797	107564	116183	125512
Independent reflections	9970	9172	9922	9147	9134
R(int)	0.0299	0.0621	0.0722	0.0464	0.0486
Crystal dimensions [mm]	0.230 x 0.211 x 0.154	0.196 x 0.148 x 0.146	0.144 x 0.134 x 0.084	0.195 x 0.126 x 0.111	0.177 x 0.146 x 0.112
Data / Restraints / parameter	9970 / 1067 / 581	9172 / 656 / 481	9922 / 1079 / 581	9147 / 1079 / 581	9134 / 1079 / 581
GoF	1.085	1.017	1.030	1.067	1.079
R1 / *w*R2 (I > 2σ(I))	0.0169 / 0.0370	0.0265 / 0.0553	0.0256 / 0.0511	0.0210 / 0.0452	0.0248 / 0.0594
R1 / *w*R2 (all data)	0.0230 / 0.0407	0.0416 / 0.0609	0.0416 / 0.0571	0.0334 / 0.0505	0.0369 / 0.0662
max. diff peak / hole [$eÅ^{-3}$]	0.941 / -0.489	0.721 / -0.609	0.769 / -0.746	0.971 / -0.781	1.358 / -0.883

Table S2 Crystallographic details for compounds **2a-e**.

compound	**2a**	**2b**	**2c**	**2d**	**2e**
CCDC no.					
Empirical formula	$C_{32}H_{68}Cl_2GdLiN_4O_4S$	$C_{32}H_{68}Cl_2TbLiN_4O_4S$	$C_{32}H_{68}Cl_2DyLiN_4O_4S$	$C_{32}H_{68}Cl_2HoLiN_4O_4S$	$C_{32}H_{68}Cl_2ErLiN_4O_4S$
Formula weight [g/mol]	840.05	841.72	845.30	847.73	850.06
Temperature [K]	100(2)	100(2)	100(2)	100(2)	100(2)
Wavelength [Å]	0.56086	0.56086	0.56086	0.56086	0.56086
Crystal system	Orthorhombic	Orthorhombic	Orthorhombic	Orthorhombic	Orthorhombic
Space group	$Pca2_1$	$Pca2_1$	$Pca2_1$	$Pca2_1$	$Pca2_1$
a [Å]	16.939(2)	16.900(2)	16.967(7)	16.887(2)	16.883(2)
b [Å]	12.911(2)	12.910(2)	12.925(6)	12.873(2)	12.859(2)
c [Å]	18.093(3)	18.088(3)	18.161(8)	18.069(3)	18.070(3)
Volume [$Å^3$]	3956.9(10)	3946.4(10)	3983(3)	3928.0(10)	3923.0(10)
Z	4	4	4	4	4
ρ_{calc} [g/cm^3]	1.410	1.417	1.410	1.434	1.439
μ [mm^{-1}]	1.018	1.077	1.125	1.197	1.270
F(000)	1748	1752	1756	1760	1764
θ range [°]	1.565 to 22.657	1.902 to 27.890	1.563 to 23.612	1.570 to 27.864	1.779 to 27.923
Reflections collected	172173	99654	289767	133552	198994
Independent reflections	10704	19090	12081	19027	19082
R(int)	0.0956	0.0630	0.0637	0.0587	0.0507
Crystal dimensions [mm]	0.190 x 0.097 x 0.097	0.132 x 0.120 x 0.102	0.204 x 0.125 x 0.079	0.229 x 0.185 x 0.170	0.228 x 0.176 x 0.157
Data / Restraints / parameter	10704 / 3946 / 600	19090 / 3948 / 600	12081 / 3922 / 600	19027 / 3927 / 606	19082 / 3929 / 599
GoF	1.029	1.011	1.072	1.045	1.018
R1 / *w*R2 (I > 2σ(I))	0.0242 / 0.0472	0.0282 / 0.0488	0.0191 / 0.0400	0.0251 / 0.0559	0.0185 / 0.0369
R1 / *w*R2 (all data)	0.0365 / 0.0509	0.0473 / 0.0529	0.0275 / 0.0426	0.0341 / 0.0598	0.0266 / 0.0392
Absolute structure parameter	0.505(16)	0.464(11)	0.267(11)	0.430(10)	0.157(7)
Extinction coefficient	0.00031(8)	0.00046(10)	0.00025(6)	0.00146(15)	-
max. diff peak / hole [e Å^{-3}]	0.600 / -0.673	0.510 / -0.659	0.626 / -0.525	1.466 / -1.079	0.556 / -0.690

Table S3 Crystallographic details for compound **3c**.

CCDC no.		ρ_{calc} [g/cm^3]	1.160
Empirical formula	$C_{96}H_{208}Cl_8Li_4N_{16}O_8S_4Tb_4$	μ [mm^{-1}]	1.053
Formula weight [g/mol]	2790.05	F(000)	11456
Temperature [K]	100(2)	θ range [°]	1.518 to 18.206°.
Wavelength [Å]	0.56086	Reflections collected	285015
Crystal system	Monoclinic	Independent reflections	22853
Space group	C2/c	R(int)	0.1400
a [Å]	67.205(4)	Crystal dimensions [mm]	0.169 x 0.152 x 0.100
b [Å]	22.803(2)	Data / Restraints / parameter	22853 / 10529 / 1863
c [Å]	21.301(2)	GoF	1.133
β [°]	101.92(2)	R1 / *w*R2 (I > 2σ(I))	0.0760 / 0.1492
Volume [$Å^3$]	31939(5)	R1 / *w*R2 (all data)	0.1216 / 0.1760
Z	8	max. diff peak / hole [eÅ^{-3}]	2.743 and -1.501

XRD-Analysis of 1a

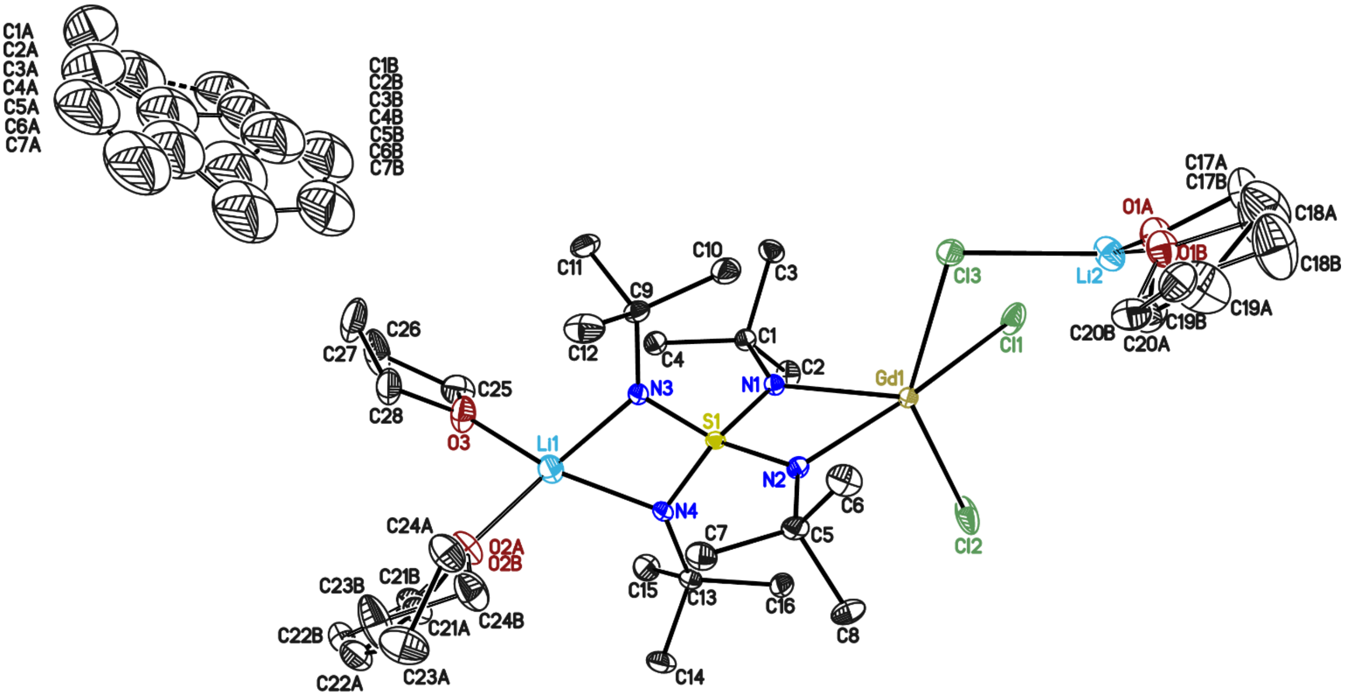

Figure S1 Asymmetric unit of **1a**. Anisotropic displacement parameters are depicted on a probability level of 50%. Hydrogen atoms are omitted for clarity. The disordered thf molecules were refined on two positions. The occupancy of the main positions for the thf molecule with O1B was refined to 0.543(14) and for the thf molecule with O2A was refined to 0.623(13). The disordered toluene was refined on four positions, of which two each are related by a 2-fold axis. The occupancy of the main positions was refined to 0.309(5). For the refinement, distance restraints and restraints for the anisotropic displacement parameters were used.

Table S4 Bond length of **1a** in Å.

**bond**	**length [Å]**	**bond**	**length [Å]**
Li(1)-N(4)	1.981(3)	O(1A)-C(20A)	1.430(10)
Li(1)-N(3)	1.995(3)	O(1A)-C(17A)	1.441(10)
Li(1)-O(2B)	2.006(3)	C(17A)-C(18A)	1.506(8)
Li(1)-O(2A)	2.006(3)	C(18A)-C(19A)	1.480(9)
Li(1)-O(3)	2.021(3)	C(19A)-C(20A)	1.502(9)
Li(1)-S(1)	2.646(3)	O(1B)-C(17B)	1.428(9)
N(1)-C(1)	1.4861(18)	O(1B)-C(20B)	1.451(8)
N(1)-S(1)	1.6222(13)	C(17B)-C(18B)	1.514(7)
N(1)-Gd(1)	2.2885(12)	C(18B)-C(19B)	1.495(7)
S(1)-N(4)	1.5650(13)	C(19B)-C(20B)	1.500(8)
S(1)-N(3)	1.5688(13)	O(2A)-C(24A)	1.440(4)
S(1)-N(2)	1.6292(12)	O(2A)-C(21A)	1.442(7)
S(1)-Gd(1)	3.1152(5)	C(21A)-C(22A)	1.511(7)
Gd(1)-N(2)	2.3098(13)	C(22A)-C(23A)	1.509(7)
Gd(1)-Cl(3)	2.6707(5)	C(23A)-C(24A)	1.514(7)
Gd(1)-Cl(2)	2.7248(5)	O(2B)-C(24B)	1.438(6)
Gd(1)-Cl(1)#1	2.7885(5)	O(2B)-C(21B)	1.452(11)
Gd(1)-Cl(1)	2.8343(9)	C(21B)-C(22B)	1.512(12)
Gd(1)-Gd(1)#1	3.8347(5)	C(22B)-C(23B)	1.505(11)
C(1)-C(4)	1.527(2)	C(23B)-C(24B)	1.509(10)
C(1)-C(3)	1.532(2)	O(3)-C(28)	1.440(2)
C(1)-C(2)	1.535(2)	O(3)-C(25)	1.446(2)
N(3)-C(9)	1.4784(19)	C(25)-C(26)	1.504(3)
Cl(3)-Li(2)	2.4610(14)	C(26)-C(27)	1.517(3)
N(2)-C(5)	1.4900(18)	C(27)-C(28)	1.514(3)
Li(2)-O(1A)#1	1.929(14)	C(1A)-C(2A)	1.36(2)
Li(2)-O(1A)	1.929(14)	C(2A)-C(7A)	1.377(13)
Li(2)-O(1B)#1	2.049(11)	C(2A)-C(3A)	1.385(13)
Li(2)-O(1B)	2.049(11)	C(3A)-C(4A)	1.396(13)
N(4)-C(13)	1.4754(19)	C(4A)-C(5A)	1.413(14)
C(6)-C(5)	1.535(2)	C(5A)-C(6A)	1.406(14)
C(5)-C(7)	1.526(2)	C(6A)-C(7A)	1.406(14)
C(5)-C(8)	1.532(2)	C(1B)-C(2B)	1.34(2)
C(9)-C(10)	1.525(2)	C(2B)-C(7B)	1.392(12)
C(9)-C(12)	1.534(2)	C(2B)-C(3B)	1.413(12)
C(9)-C(11)	1.535(2)	C(3B)-C(4B)	1.394(13)
C(13)-C(16)	1.526(2)	C(4B)-C(5B)	1.414(12)
C(13)-C(14)	1.533(2)	C(5B)-C(6B)	1.394(12)
C(13)-C(15)	1.537(2)	C(6B)-C(7B)	1.367(12)

Symmetry transformations used to generate equivalent atoms:
#1 -x+1,y,-z+3/2

Table S5 Bond angles of **1a**.

N(4)-Li(1)-N(3)	72.11(11)	N(1)-Gd(1)-Gd(1)#1	137.83(3)	C(14)-C(13)-C(15)	107.75(13)
N(4)-Li(1)-O(2B)	119.38(15)	N(2)-Gd(1)-Gd(1)#1	137.81(3)	C(20A)-O(1A)-C(17A)	106.5(8)
N(3)-Li(1)-O(2B)	125.62(15)	Cl(3)-Gd(1)-Gd(1)#1	98.930(9)	C(20A)-O(1A)-Li(2)	115.6(10)
N(4)-Li(1)-O(2A)	119.38(15)	Cl(2)-Gd(1)-Gd(1)#1	45.279(10)	C(17A)-O(1A)-Li(2)	124.7(10)
N(3)-Li(1)-O(2A)	125.62(15)	Cl(1)#1-Gd(1)-Gd(1)#1	47.503(17)	O(1A)-C(17A)-C(18A)	105.7(7)
N(4)-Li(1)-O(3)	130.48(16)	Cl(1)-Gd(1)-Gd(1)#1	46.502(13)	C(19A)-C(18A)-C(17A)	106.1(6)
N(3)-Li(1)-O(3)	118.04(15)	S(1)-Gd(1)-Gd(1)#1	149.410(8)	C(18A)-C(19A)-C(20A)	105.5(6)
O(2B)-Li(1)-O(3)	94.44(13)	N(1)-C(1)-C(4)	115.82(12)	O(1A)-C(20A)-C(19A)	106.0(8)
O(2A)-Li(1)-O(3)	94.44(13)	N(1)-C(1)-C(3)	107.00(12)	C(17B)-O(1B)-C(20B)	107.2(6)
N(4)-Li(1)-S(1)	36.04(6)	C(4)-C(1)-C(3)	109.17(12)	C(17B)-O(1B)-Li(2)	127.0(8)
N(3)-Li(1)-S(1)	36.19(6)	N(1)-C(1)-C(2)	108.41(12)	C(20B)-O(1B)-Li(2)	120.1(7)
O(2B)-Li(1)-S(1)	133.77(14)	C(4)-C(1)-C(2)	108.05(12)	O(1B)-C(17B)-C(18B)	107.2(6)
O(2A)-Li(1)-S(1)	133.77(14)	C(3)-C(1)-C(2)	108.16(12)	C(19B)-C(18B)-C(17B)	105.2(5)
O(3)-Li(1)-S(1)	131.65(13)	Gd(1)#1-Cl(1)-Gd(1)	85.994(15)	C(18B)-C(19B)-C(20B)	102.4(5)
C(1)-N(1)-S(1)	125.80(10)	C(9)-N(3)-S(1)	129.11(10)	O(1B)-C(20B)-C(19B)	104.7(6)
C(1)-N(1)-Gd(1)	129.88(9)	C(9)-N(3)-Li(1)	135.11(13)	C(24A)-O(2A)-C(21A)	109.9(4)
S(1)-N(1)-Gd(1)	104.31(6)	S(1)-N(3)-Li(1)	95.16(10)	C(24A)-O(2A)-Li(1)	126.36(19)
N(4)-S(1)-N(3)	96.61(7)	Li(2)-Cl(3)-Gd(1)	99.17(10)	C(21A)-O(2A)-Li(1)	123.7(4)
N(4)-S(1)-N(1)	117.38(7)	C(5)-N(2)-S(1)	124.88(10)	O(2A)-C(21A)-C(22A)	106.6(6)
N(3)-S(1)-N(1)	117.79(7)	C(5)-N(2)-Gd(1)	131.94(9)	C(23A)-C(22A)-C(21A)	102.3(6)
N(4)-S(1)-N(2)	118.30(7)	S(1)-N(2)-Gd(1)	103.17(6)	C(22A)-C(23A)-C(24A)	103.1(5)
N(3)-S(1)-N(2)	117.06(7)	O(1A)#1-Li(2)-O(1A)	98.6(8)	O(2A)-C(24A)-C(23A)	104.2(4)
N(1)-S(1)-N(2)	91.60(6)	O(1A)#1-Li(2)-O(1B)#1	6.0(10)	C(24B)-O(2B)-C(21B)	105.2(9)
N(4)-S(1)-Li(1)	48.13(8)	O(1A)-Li(2)-O(1B)#1	103.6(3)	C(24B)-O(2B)-Li(1)	129.3(3)
N(3)-S(1)-Li(1)	48.66(8)	O(1B)#1-Li(2)-O(1B)	108.9(7)	C(21B)-O(2B)-Li(1)	120.9(7)
N(1)-S(1)-Li(1)	130.69(8)	O(1A)#1-Li(2)-Cl(3)	105.9(7)	O(2B)-C(21B)-C(22B)	105.3(10)
N(2)-S(1)-Li(1)	137.71(8)	O(1A)-Li(2)-Cl(3)	97.8(6)	C(23B)-C(22B)-C(21B)	105.2(9)
N(4)-S(1)-Gd(1)	132.27(5)	O(1B)#1-Li(2)-Cl(3)	101.8(5)	C(22B)-C(23B)-C(24B)	104.7(8)
N(3)-S(1)-Gd(1)	131.12(5)	O(1B)-Li(2)-Cl(3)	99.3(5)	O(2B)-C(24B)-C(23B)	104.0(7)
N(1)-S(1)-Gd(1)	45.38(4)	O(1A)#1-Li(2)-Cl(3)#1	97.8(6)	C(28)-O(3)-C(25)	108.87(14)
N(2)-S(1)-Gd(1)	46.22(5)	O(1A)-Li(2)-Cl(3)#1	105.9(7)	C(28)-O(3)-Li(1)	114.35(13)
Li(1)-S(1)-Gd(1)	176.06(6)	Cl(3)-Li(2)-Cl(3)#1	143.40(19)	C(25)-O(3)-Li(1)	136.55(14)
N(1)-Gd(1)-N(2)	60.91(4)	Gd(1)#1-Cl(2)-Gd(1)	89.44(2)	O(3)-C(25)-C(26)	106.98(16)
N(1)-Gd(1)-Cl(3)	108.69(3)	C(13)-N(4)-S(1)	129.96(10)	C(25)-C(26)-C(27)	102.67(16)
N(2)-Gd(1)-Cl(3)	108.64(3)	C(13)-N(4)-Li(1)	134.07(13)	C(28)-C(27)-C(26)	101.94(18)
N(1)-Gd(1)-Cl(2)	100.89(3)	S(1)-N(4)-Li(1)	95.83(10)	O(3)-C(28)-C(27)	105.45(15)
N(2)-Gd(1)-Cl(2)	103.25(3)	N(2)-C(5)-C(7)	115.57(13)	C(1A)-C(2A)-C(7A)	122.5(17)
Cl(3)-Gd(1)-Cl(2)	144.104(14)	N(2)-C(5)-C(8)	108.59(12)	C(1A)-C(2A)-C(3A)	120.2(18)
N(1)-Gd(1)-Cl(1)#1	163.47(3)	C(7)-C(5)-C(8)	108.68(13)	C(7A)-C(2A)-C(3A)	116.6(14)
N(2)-Gd(1)-Cl(1)#1	104.51(3)	N(2)-C(5)-C(6)	107.26(12)	C(2A)-C(3A)-C(4A)	124.2(15)
Cl(3)-Gd(1)-Cl(1)#1	82.573(14)	C(7)-C(5)-C(6)	108.66(13)	C(3A)-C(4A)-C(5A)	117.1(15)
Cl(2)-Gd(1)-Cl(1)#1	73.748(15)	C(8)-C(5)-C(6)	107.83(13)	C(6A)-C(5A)-C(4A)	118.8(15)
N(1)-Gd(1)-Cl(1)	108.24(3)	N(3)-C(9)-C(10)	116.69(12)	C(5A)-C(6A)-C(7A)	119.5(16)
N(2)-Gd(1)-Cl(1)	168.14(3)	N(3)-C(9)-C(12)	106.91(12)	C(2A)-C(7A)-C(6A)	119.5(15)
Cl(3)-Gd(1)-Cl(1)	78.648(15)	C(10)-C(9)-C(12)	108.70(14)	C(1B)-C(2B)-C(7B)	116.6(12)
Cl(2)-Gd(1)-Cl(1)	73.022(16)	N(3)-C(9)-C(11)	108.64(12)	C(1B)-C(2B)-C(3B)	118.7(14)
Cl(1)#1-Gd(1)-Cl(1)	85.461(18)	C(10)-C(9)-C(11)	108.23(13)	C(7B)-C(2B)-C(3B)	124.5(13)
N(1)-Gd(1)-S(1)	30.30(3)	C(12)-C(9)-C(11)	107.31(13)	C(4B)-C(3B)-C(2B)	116.5(13)
N(2)-Gd(1)-S(1)	30.61(3)	N(4)-C(13)-C(16)	117.23(12)	C(3B)-C(4B)-C(5B)	119.4(13)
Cl(3)-Gd(1)-S(1)	111.660(12)	N(4)-C(13)-C(14)	107.40(12)	C(6B)-C(5B)-C(4B)	119.9(13)
Cl(2)-Gd(1)-S(1)	104.159(14)	C(16)-C(13)-C(14)	108.83(13)	C(7B)-C(6B)-C(5B)	121.9(13)
Cl(1)#1-Gd(1)-S(1)	134.714(17)	N(4)-C(13)-C(15)	107.23(12)	C(6B)-C(7B)-C(2B)	116.3(13)
Cl(1)-Gd(1)-S(1)	138.386(13)	C(16)-C(13)-C(15)	108.05(13)		

Symmetry transformations used to generate equivalent atoms:
#1 -x+1,y,-z+3/2

XRD-Analysis of 1b

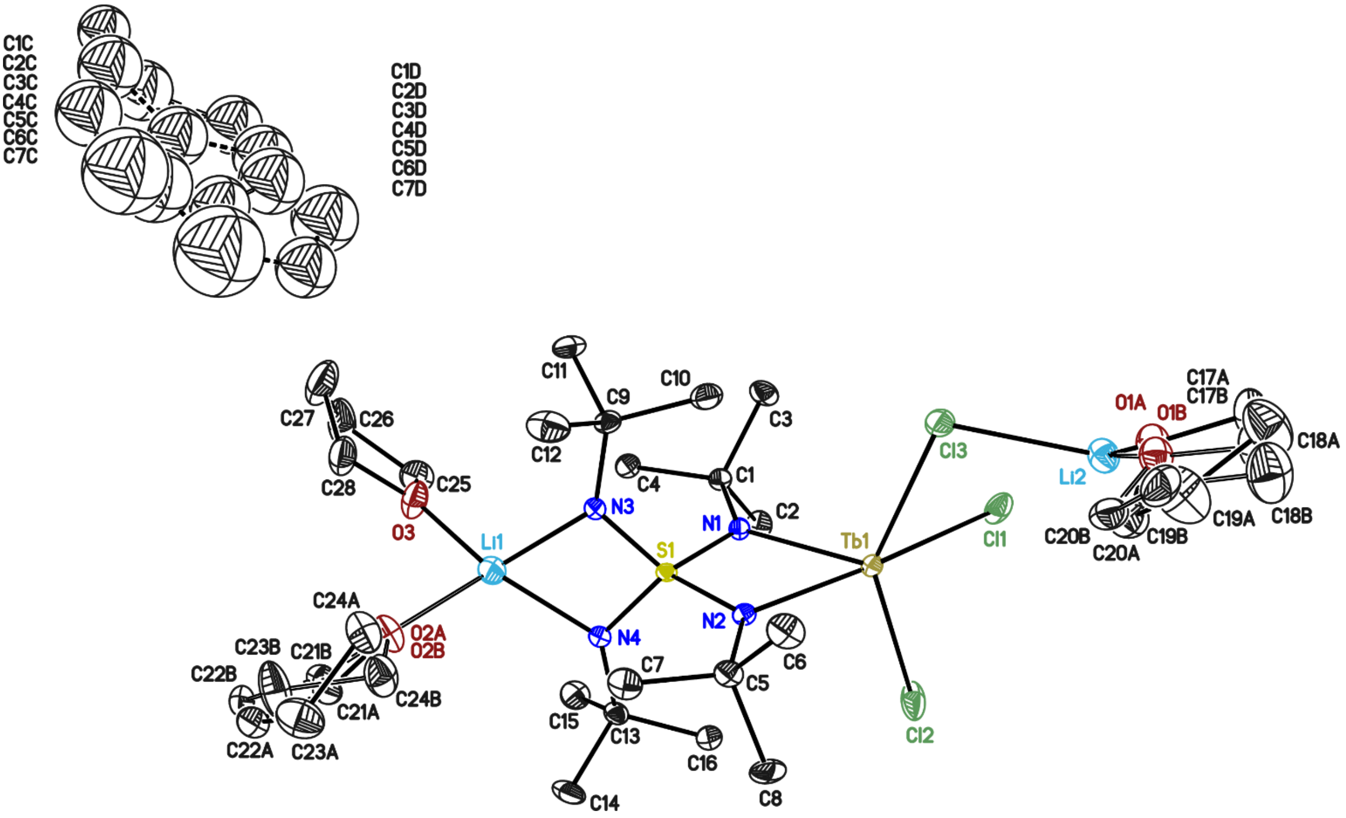

Figure S2 Asymmetric unit of **1b**. Anisotropic displacement parameters are depicted on a probability level of 50%. Hydrogen atoms are omitted for clarity. The disordered thf molecules were refined on two positions. The occupancy of the main positions for the thf molecule with O1A was refined to 0.51(2) and for the thf molecule with O2A to 0.603(19). The disordered toluene was refined on four positions, of which two each are related by a 2-fold axis. The occupancy of the main positions was refined to 0.250(7). All carbon atoms of the toluene were refined isotropically. For the refinement, distance restraints and restraints for the anisotropic displacement parameters were used.

Table S6 Bond length of **1b** in Å.

**bond**	**length [Å]**	**bond**	**length [Å]**
Li(1)-N(4)	1.981(5)	O(1A)-C(17A)	1.433(10)
Li(1)-N(3)	1.992(5)	O(1A)-C(20A)	1.437(11)
Li(1)-O(2B)	2.003(5)	C(17A)-C(18A)	1.497(9)
Li(1)-O(2A)	2.003(5)	C(18A)-C(19A)	1.503(11)
Li(1)-O(3)	2.017(5)	C(19A)-C(20A)	1.496(10)
Li(1)-S(1)	2.646(5)	O(1B)-C(17B)	1.429(11)
N(1)-C(1)	1.484(3)	O(1B)-C(20B)	1.452(10)
N(1)-S(1)	1.622(2)	C(17B)-C(18B)	1.502(10)
N(1)-Tb(1)	2.269(2)	C(18B)-C(19B)	1.512(10)
S(1)-N(4)	1.564(2)	C(19B)-C(20B)	1.502(10)
S(1)-N(3)	1.566(2)	O(2A)-C(24A)	1.434(6)
S(1)-N(2)	1.624(2)	O(2A)-C(21A)	1.445(8)
S(1)-Tb(1)	3.0976(7)	C(21A)-C(22A)	1.501(9)
Tb(1)-N(2)	2.297(2)	C(22A)-C(23A)	1.497(9)
Tb(1)-Cl(3)	2.6540(7)	C(23A)-C(24A)	1.509(9)
Tb(1)-Cl(2)	2.7118(7)	O(2B)-C(24B)	1.444(9)
Tb(1)-Cl(1)#1	2.7707(8)	O(2B)-C(21B)	1.448(11)
Tb(1)-Cl(1)	2.8172(11)	C(21B)-C(22B)	1.497(12)
Tb(1)-Tb(1)#1	3.8307(5)	C(22B)-C(23B)	1.493(12)
C(1)-C(4)	1.523(3)	C(23B)-C(24B)	1.514(12)
C(1)-C(2)	1.534(4)	O(3)-C(28)	1.437(4)
C(1)-C(3)	1.535(4)	O(3)-C(25)	1.446(4)
N(3)-C(9)	1.481(3)	C(25)-C(26)	1.501(4)
Cl(3)-Li(2)	2.460(2)	C(26)-C(27)	1.514(5)
N(2)-C(5)	1.482(3)	C(27)-C(28)	1.513(4)
Li(2)-O(1A)#1	1.950(16)	C(1C)-C(2C)	1.5059
Li(2)-O(1A)	1.950(17)	C(2C)-C(3C)	1.3921
Li(2)-O(1B)	2.042(17)	C(2C)-C(7C)	1.3946
Li(2)-O(1B)#1	2.042(17)	C(3C)-C(4C)	1.3774
N(4)-C(13)	1.473(3)	C(4C)-C(5C)	1.3897
C(6)-C(5)	1.532(4)	C(5C)-C(6C)	1.3814
C(5)-C(8)	1.532(4)	C(6C)-C(7C)	1.3802
C(5)-C(7)	1.532(4)	C(1D)-C(2D)	1.5058
C(9)-C(10)	1.524(4)	C(2D)-C(3D)	1.3921
C(9)-C(11)	1.529(4)	C(2D)-C(7D)	1.3947
C(9)-C(12)	1.536(4)	C(3D)-C(4D)	1.3775
C(13)-C(16)	1.522(4)	C(4D)-C(5D)	1.3897
C(13)-C(14)	1.533(4)	C(5D)-C(6D)	1.3814
C(13)-C(15)	1.537(4)	C(6D)-C(7D)	1.3802

Symmetry transformations used to generate equivalent atoms:
#1 -x+1,y,-z+3/2

Table S7 Bond angles of **1b**.

N(4)-Li(1)-N(3)	72.04(18)	N(1)-Tb(1)-Tb(1)#1	137.63(6)	C(14)-C(13)-C(15)	107.9(2)
N(4)-Li(1)-O(2B)	119.3(3)	N(2)-Tb(1)-Tb(1)#1	137.76(5)	C(17A)-O(1A)-C(20A)	106.8(9)
N(3)-Li(1)-O(2B)	125.8(3)	Cl(3)-Tb(1)-Tb(1)#1	99.121(16)	C(17A)-O(1A)-Li(2)	124.3(11)
N(4)-Li(1)-O(2A)	119.3(3)	Cl(2)-Tb(1)-Tb(1)#1	45.068(15)	C(20A)-O(1A)-Li(2)	117.1(11)
N(3)-Li(1)-O(2A)	125.8(3)	Cl(1)#1-Tb(1)-Tb(1)#1	47.23(2)	O(1A)-C(17A)-C(18A)	105.8(8)
N(4)-Li(1)-O(3)	130.4(3)	Cl(1)-Tb(1)-Tb(1)#1	46.221(17)	C(17A)-C(18A)-C(19A)	106.3(7)
N(3)-Li(1)-O(3)	118.3(2)	S(1)-Tb(1)-Tb(1)#1	149.403(12)	C(20A)-C(19A)-C(18A)	104.7(8)
O(2B)-Li(1)-O(3)	94.3(2)	N(1)-C(1)-C(4)	115.9(2)	O(1A)-C(20A)-C(19A)	106.0(10)
O(2A)-Li(1)-O(3)	94.3(2)	N(1)-C(1)-C(2)	108.5(2)	C(17B)-O(1B)-C(20B)	106.7(9)
N(4)-Li(1)-S(1)	36.03(10)	C(4)-C(1)-C(2)	107.9(2)	C(17B)-O(1B)-Li(2)	126.8(13)
N(3)-Li(1)-S(1)	36.12(10)	N(1)-C(1)-C(3)	107.0(2)	C(20B)-O(1B)-Li(2)	120.3(10)
O(2B)-Li(1)-S(1)	133.8(2)	C(4)-C(1)-C(3)	109.4(2)	O(1B)-C(17B)-C(18B)	107.4(8)
O(2A)-Li(1)-S(1)	133.8(2)	C(2)-C(1)-C(3)	107.9(2)	C(17B)-C(18B)-C(19B)	105.6(8)
O(3)-Li(1)-S(1)	131.8(2)	Tb(1)#1-Cl(1)-Tb(1)	86.55(2)	C(20B)-C(19B)-C(18B)	101.2(7)
C(1)-N(1)-S(1)	125.64(17)	C(9)-N(3)-S(1)	129.31(17)	O(1B)-C(20B)-C(19B)	105.1(9)
C(1)-N(1)-Tb(1)	130.09(16)	C(9)-N(3)-Li(1)	134.8(2)	C(24A)-O(2A)-C(21A)	109.5(6)
S(1)-N(1)-Tb(1)	104.27(10)	S(1)-N(3)-Li(1)	95.30(17)	C(24A)-O(2A)-Li(1)	126.7(3)
N(4)-S(1)-N(3)	96.54(11)	Li(2)-Cl(3)-Tb(1)	98.65(15)	C(21A)-O(2A)-Li(1)	123.8(6)
N(4)-S(1)-N(1)	117.35(12)	C(5)-N(2)-S(1)	125.27(17)	O(2A)-C(21A)-C(22A)	106.6(7)
N(3)-S(1)-N(1)	117.97(11)	C(5)-N(2)-Tb(1)	131.70(16)	C(23A)-C(22A)-C(21A)	102.3(7)
N(4)-S(1)-N(2)	118.36(11)	S(1)-N(2)-Tb(1)	103.03(11)	C(22A)-C(23A)-C(24A)	103.4(7)
N(3)-S(1)-N(2)	117.06(12)	O(1A)#1-Li(2)-O(1A)	99.4(11)	O(2A)-C(24A)-C(23A)	104.4(6)
N(1)-S(1)-N(2)	91.47(11)	O(1A)#1-Li(2)-O(1B)#1	5.4(14)	C(24B)-O(2B)-C(21B)	105.4(10)
N(4)-S(1)-Li(1)	48.14(13)	O(1A)-Li(2)-O(1B)#1	104.0(4)	C(24B)-O(2B)-Li(1)	129.7(4)
N(3)-S(1)-Li(1)	48.58(13)	O(1B)-Li(2)-O(1B)#1	108.7(12)	C(21B)-O(2B)-Li(1)	121.1(8)
N(1)-S(1)-Li(1)	130.81(13)	O(1A)#1-Li(2)-Cl(3)	105.4(8)	O(2B)-C(21B)-C(22B)	106.0(10)
N(2)-S(1)-Li(1)	137.72(14)	O(1A)-Li(2)-Cl(3)	97.7(7)	C(23B)-C(22B)-C(21B)	106.1(10)
N(4)-S(1)-Tb(1)	132.28(8)	O(1B)-Li(2)-Cl(3)	98.9(7)	C(22B)-C(23B)-C(24B)	104.6(10)
N(3)-S(1)-Tb(1)	131.17(8)	O(1B)#1-Li(2)-Cl(3)	101.8(8)	O(2B)-C(24B)-C(23B)	104.3(9)
N(1)-S(1)-Tb(1)	45.23(7)	O(1A)#1-Li(2)-Cl(3)#1	97.7(7)	C(28)-O(3)-C(25)	109.0(2)
N(2)-S(1)-Tb(1)	46.25(8)	O(1A)-Li(2)-Cl(3)#1	105.4(8)	C(28)-O(3)-Li(1)	114.4(2)
Li(1)-S(1)-Tb(1)	176.01(11)	Cl(3)-Li(2)-Cl(3)#1	144.1(3)	C(25)-O(3)-Li(1)	136.4(2)
N(1)-Tb(1)-N(2)	61.22(8)	Tb(1)-Cl(2)-Tb(1)#1	89.87(3)	O(3)-C(25)-C(26)	107.0(3)
N(1)-Tb(1)-Cl(3)	108.63(6)	C(13)-N(4)-S(1)	130.06(17)	C(25)-C(26)-C(27)	102.7(3)
N(2)-Tb(1)-Cl(3)	108.37(6)	C(13)-N(4)-Li(1)	134.0(2)	C(28)-C(27)-C(26)	102.1(3)
N(1)-Tb(1)-Cl(2)	100.93(6)	S(1)-N(4)-Li(1)	95.83(17)	O(3)-C(28)-C(27)	105.3(3)
N(2)-Tb(1)-Cl(2)	103.47(6)	N(2)-C(5)-C(8)	108.7(2)	C(3C)-C(2C)-C(7C)	117.9
Cl(3)-Tb(1)-Cl(2)	144.09(2)	N(2)-C(5)-C(7)	115.3(2)	C(3C)-C(2C)-C(1C)	121.1
N(1)-Tb(1)-Cl(1)#1	164.14(6)	C(8)-C(5)-C(7)	108.5(2)	C(7C)-C(2C)-C(1C)	120.9
N(2)-Tb(1)-Cl(1)#1	104.91(6)	N(2)-C(5)-C(6)	107.5(2)	C(4C)-C(3C)-C(2C)	121.7
Cl(3)-Tb(1)-Cl(1)#1	82.28(2)	C(8)-C(5)-C(6)	108.1(2)	C(3C)-C(4C)-C(5C)	119.5
Cl(2)-Tb(1)-Cl(1)#1	73.76(2)	C(7)-C(5)-C(6)	108.5(2)	C(6C)-C(5C)-C(4C)	119.7
N(1)-Tb(1)-Cl(1)	108.61(6)	N(3)-C(9)-C(10)	116.4(2)	C(7C)-C(6C)-C(5C)	120.4
N(2)-Tb(1)-Cl(1)	168.88(5)	N(3)-C(9)-C(11)	108.6(2)	C(6C)-C(7C)-C(2C)	120.8
Cl(3)-Tb(1)-Cl(1)	78.46(2)	C(10)-C(9)-C(11)	108.3(2)	C(3D)-C(2D)-C(7D)	117.9
Cl(2)-Tb(1)-Cl(1)	73.02(2)	N(3)-C(9)-C(12)	106.9(2)	C(3D)-C(2D)-C(1D)	121.1
Cl(1)#1-Tb(1)-Cl(1)	84.45(3)	C(10)-C(9)-C(12)	108.8(2)	C(7D)-C(2D)-C(1D)	120.9
N(1)-Tb(1)-S(1)	30.50(6)	C(11)-C(9)-C(12)	107.6(2)	C(4D)-C(3D)-C(2D)	121.7
N(2)-Tb(1)-S(1)	30.72(5)	N(4)-C(13)-C(16)	117.1(2)	C(3D)-C(4D)-C(5D)	119.5
Cl(3)-Tb(1)-S(1)	111.48(2)	N(4)-C(13)-C(14)	107.2(2)	C(6D)-C(5D)-C(4D)	119.7
Cl(2)-Tb(1)-S(1)	104.36(2)	C(16)-C(13)-C(14)	108.7(2)	C(7D)-C(6D)-C(5D)	120.4
Cl(1)#1-Tb(1)-S(1)	135.24(2)	N(4)-C(13)-C(15)	107.4(2)	C(6D)-C(7D)-C(2D)	120.8
Cl(1)-Tb(1)-S(1)	138.98(2)	C(16)-C(13)-C(15)	108.2(2)		

Symmetry transformations used to generate equivalent atoms:
#1 -x+1,y,-z+3/2

XRD-Analysis of 1c

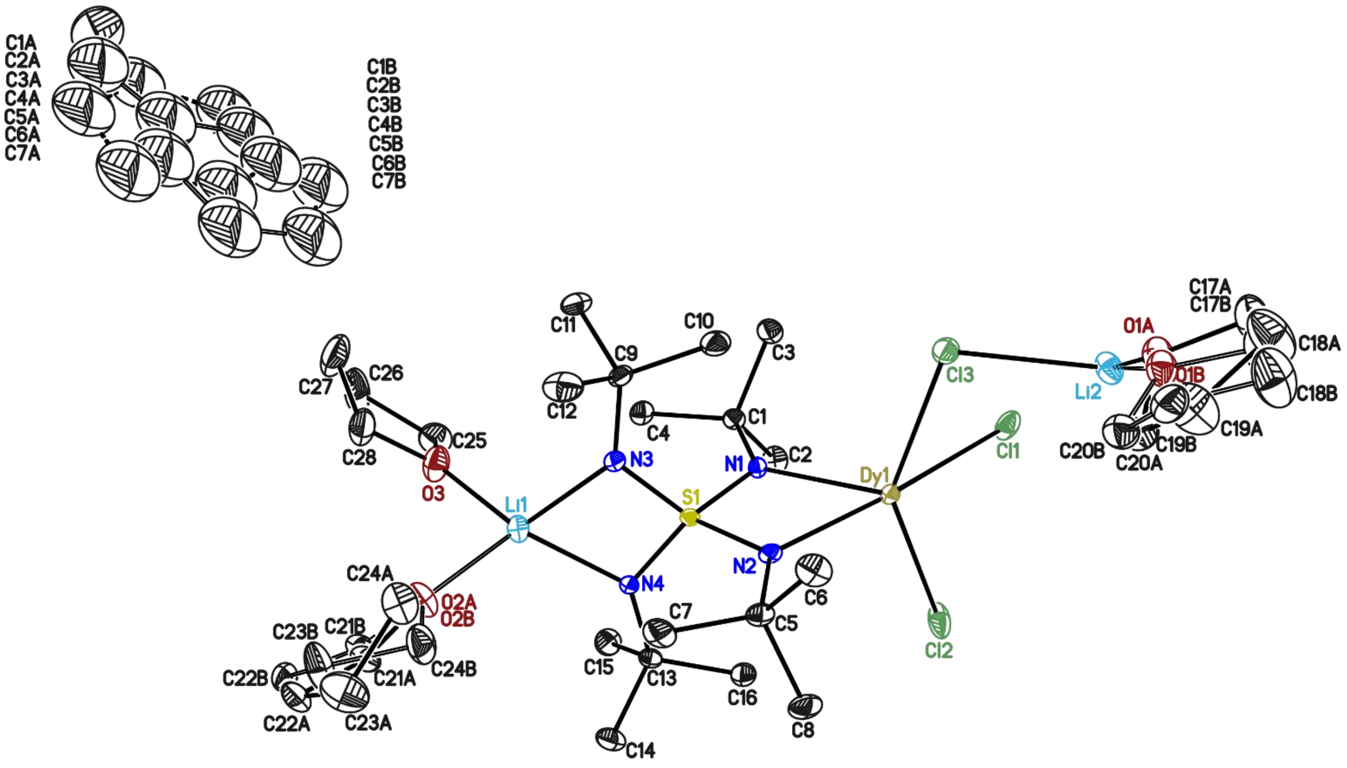

Figure S3 Asymmetric unit of **1c**. Anisotropic displacement parameters are depicted on a probability level of 50%. Hydrogen atoms are omitted for clarity. The disordered thf molecules were refined on two positions. The occupancy of the main positions for the thf molecule with O1A was refined to 0.51(2) and for the thf molecule with O2A to 0.636(17). The disordered toluene was refined on four positions, of which two each are related by a 2-fold axis. The occupancy of the main positions was refined to 0.322(6). For the refinement distance restraints and restraints for the anisotropic displacement parameters were used.

Table S8 Bond length of **1c** in Å.

bond	length [Å]	bond	length [Å]
Li(1)-N(4)	1.979(5)	O(1A)-C(17A)	1.437(10)
Li(1)-N(3)	1.995(5)	O(1A)-C(20A)	1.442(11)
Li(1)-O(2B)	2.003(5)	C(17A)-C(18A)	1.515(9)
Li(1)-O(2A)	2.003(5)	C(18A)-C(19A)	1.490(10)
Li(1)-O(3)	2.021(5)	C(19A)-C(20A)	1.497(10)
Li(1)-S(1)	2.648(4)	O(1B)-C(17B)	1.431(10)
N(1)-C(1)	1.490(3)	O(1B)-C(20B)	1.445(10)
N(1)-S(1)	1.623(2)	C(17B)-C(18B)	1.506(9)
N(1)-Dy(1)	2.2575(19)	C(18B)-C(19B)	1.497(10)
S(1)-N(4)	1.566(2)	C(19B)-C(20B)	1.500(10)
S(1)-N(3)	1.570(2)	O(2A)-C(24A)	1.440(6)
S(1)-N(2)	1.6299(19)	O(2A)-C(21A)	1.443(8)
S(1)-Dy(1)	3.0874(7)	C(21A)-C(22A)	1.510(8)
Dy(1)-N(2)	2.283(2)	C(22A)-C(23A)	1.508(8)
Dy(1)-Cl(3)	2.6447(7)	C(23A)-C(24A)	1.503(9)
Dy(1)-Cl(2)	2.6981(7)	O(2B)-C(24B)	1.439(9)
Dy(1)-Cl(1)#1	2.7542(7)	O(2B)-C(21B)	1.449(12)
Dy(1)-Cl(1)	2.8083(10)	C(21B)-C(22B)	1.509(12)
Dy(1)-Dy(1)#1	3.7969(5)	C(22B)-C(23B)	1.496(12)
C(1)-C(4)	1.526(3)	C(23B)-C(24B)	1.506(11)
C(1)-C(3)	1.528(3)	O(3)-C(28)	1.442(3)
C(1)-C(2)	1.533(3)	O(3)-C(25)	1.448(3)
N(3)-C(9)	1.477(3)	C(25)-C(26)	1.507(4)
Cl(3)-Li(2)	2.459(2)	C(26)-C(27)	1.515(5)
N(2)-C(5)	1.490(3)	C(27)-C(28)	1.513(4)
Li(2)-O(1A)#1	1.953(16)	C(1A)-C(2A)	1.34(3)
Li(2)-O(1A)	1.953(16)	C(2A)-C(7A)	1.382(14)
Li(2)-O(1B)	2.036(16)	C(2A)-C(3A)	1.390(14)
Li(2)-O(1B)#1	2.036(16)	C(3A)-C(4A)	1.392(14)
N(4)-C(13)	1.477(3)	C(4A)-C(5A)	1.400(15)
C(6)-C(5)	1.531(4)	C(5A)-C(6A)	1.403(14)
C(5)-C(8)	1.526(3)	C(6A)-C(7A)	1.401(14)
C(5)-C(7)	1.532(3)	C(1B)-C(2B)	1.33(3)
C(9)-C(10)	1.523(3)	C(2B)-C(7B)	1.391(13)
C(9)-C(11)	1.534(3)	C(2B)-C(3B)	1.402(13)
C(9)-C(12)	1.535(3)	C(3B)-C(4B)	1.388(13)
C(13)-C(16)	1.523(3)	C(4B)-C(5B)	1.401(13)
C(13)-C(14)	1.527(3)	C(5B)-C(6B)	1.395(13)
C(13)-C(15)	1.533(3)	C(6B)-C(7B)	1.376(13)

Symmetry transformations used to generate equivalent atoms:
#1 -x+1,y,-z+3/2

Table S9 Bond angles of **1c**.

N(4)-Li(1)-N(3)	72.08(16)	N(1)-Dy(1)-Dy(1)#1	137.30(5)	C(14)-C(13)-C(15)	108.0(2)
N(4)-Li(1)-O(2B)	119.4(2)	N(2)-Dy(1)-Dy(1)#1	137.76(5)	C(17A)-O(1A)-C(20A)	106.8(9)
N(3)-Li(1)-O(2B)	125.3(3)	Cl(3)-Dy(1)-Dy(1)#1	99.290(14)	C(17A)-O(1A)-Li(2)	124.1(11)
N(4)-Li(1)-O(2A)	119.4(2)	Cl(2)-Dy(1)-Dy(1)#1	45.280(15)	C(20A)-O(1A)-Li(2)	116.8(11)
N(3)-Li(1)-O(2A)	125.3(3)	Cl(1)#1-Dy(1)-Dy(1)#1	47.55(2)	O(1A)-C(17A)-C(18A)	105.5(8)
N(4)-Li(1)-O(3)	130.8(3)	Cl(1)-Dy(1)-Dy(1)#1	46.361(16)	C(19A)-C(18A)-C(17A)	106.2(7)
N(3)-Li(1)-O(3)	118.1(2)	S(1)-Dy(1)-Dy(1)#1	149.370(12)	C(18A)-C(19A)-C(20A)	105.3(7)
O(2B)-Li(1)-O(3)	94.40(19)	N(1)-C(1)-C(4)	115.76(19)	O(1A)-C(20A)-C(19A)	105.8(9)
O(2A)-Li(1)-O(3)	94.40(19)	N(1)-C(1)-C(3)	106.94(19)	C(17B)-O(1B)-C(20B)	107.0(8)
N(4)-Li(1)-S(1)	36.03(9)	C(4)-C(1)-C(3)	109.21(19)	C(17B)-O(1B)-Li(2)	127.7(12)
N(3)-Li(1)-S(1)	36.18(9)	N(1)-C(1)-C(2)	108.36(19)	C(20B)-O(1B)-Li(2)	119.9(10)
O(2B)-Li(1)-S(1)	133.6(2)	C(4)-C(1)-C(2)	108.0(2)	O(1B)-C(17B)-C(18B)	107.3(8)
O(2A)-Li(1)-S(1)	133.6(2)	C(3)-C(1)-C(2)	108.4(2)	C(19B)-C(18B)-C(17B)	105.5(7)
O(3)-Li(1)-S(1)	131.8(2)	Dy(1)#1-Cl(1)-Dy(1)	86.09(2)	C(18B)-C(19B)-C(20B)	102.1(7)
C(1)-N(1)-S(1)	125.51(15)	C(9)-N(3)-S(1)	129.18(16)	O(1B)-C(20B)-C(19B)	105.3(9)
C(1)-N(1)-Dy(1)	130.27(14)	C(9)-N(3)-Li(1)	134.90(19)	C(24A)-O(2A)-C(21A)	109.3(5)
S(1)-N(1)-Dy(1)	104.22(9)	S(1)-N(3)-Li(1)	95.21(16)	C(24A)-O(2A)-Li(1)	126.7(3)
N(4)-S(1)-N(3)	96.42(11)	Li(2)-Cl(3)-Dy(1)	99.14(14)	C(21A)-O(2A)-Li(1)	124.0(5)
N(4)-S(1)-N(1)	117.41(11)	C(5)-N(2)-S(1)	125.02(16)	O(2A)-C(21A)-C(22A)	106.5(6)
N(3)-S(1)-N(1)	118.00(11)	C(5)-N(2)-Dy(1)	132.06(15)	C(23A)-C(22A)-C(21A)	102.6(6)
N(4)-S(1)-N(2)	118.46(11)	S(1)-N(2)-Dy(1)	102.92(9)	C(24A)-C(23A)-C(22A)	102.7(6)
N(3)-S(1)-N(2)	117.25(11)	O(1A)#1-Li(2)-O(1A)	99.4(10)	O(2A)-C(24A)-C(23A)	105.3(5)
N(1)-S(1)-N(2)	91.24(10)	O(1A)#1-Li(2)-O(1B)#1	5.7(14)	C(24B)-O(2B)-C(21B)	106.5(10)
N(4)-S(1)-Li(1)	48.02(12)	O(1A)-Li(2)-O(1B)#1	104.1(4)	C(24B)-O(2B)-Li(1)	129.1(4)
N(3)-S(1)-Li(1)	48.60(12)	O(1B)-Li(2)-O(1B)#1	109.1(11)	C(21B)-O(2B)-Li(1)	119.8(8)
N(1)-S(1)-Li(1)	130.68(12)	O(1A)#1-Li(2)-Cl(3)#1	98.0(7)	O(2B)-C(21B)-C(22B)	104.9(11)
N(2)-S(1)-Li(1)	138.08(13)	O(1A)-Li(2)-Cl(3)#1	105.9(8)	C(23B)-C(22B)-C(21B)	105.0(10)
N(4)-S(1)-Dy(1)	132.37(8)	O(1A)#1-Li(2)-Cl(3)	105.9(8)	C(22B)-C(23B)-C(24B)	106.2(10)
N(3)-S(1)-Dy(1)	131.21(8)	O(1A)-Li(2)-Cl(3)	98.0(7)	O(2B)-C(24B)-C(23B)	102.7(8)
N(1)-S(1)-Dy(1)	45.14(7)	O(1B)-Li(2)-Cl(3)	99.4(7)	C(28)-O(3)-C(25)	108.9(2)
N(2)-S(1)-Dy(1)	46.11(7)	O(1B)#1-Li(2)-Cl(3)	102.0(8)	C(28)-O(3)-Li(1)	114.3(2)
Li(1)-S(1)-Dy(1)	175.80(10)	Cl(3)#1-Li(2)-Cl(3)	142.7(3)	C(25)-O(3)-Li(1)	136.6(2)
N(1)-Dy(1)-N(2)	61.61(7)	Dy(1)#1-Cl(2)-Dy(1)	89.44(3)	O(3)-C(25)-C(26)	106.9(2)
N(1)-Dy(1)-Cl(3)	108.56(5)	C(13)-N(4)-S(1)	130.30(16)	C(25)-C(26)-C(27)	102.6(2)
N(2)-Dy(1)-Cl(3)	108.19(5)	C(13)-N(4)-Li(1)	133.6(2)	C(28)-C(27)-C(26)	102.2(3)
N(1)-Dy(1)-Cl(2)	100.53(5)	S(1)-N(4)-Li(1)	95.95(16)	O(3)-C(28)-C(27)	105.3(2)
N(2)-Dy(1)-Cl(2)	103.35(5)	N(2)-C(5)-C(8)	108.6(2)	C(1A)-C(2A)-C(7A)	121.5(19)
Cl(3)-Dy(1)-Cl(2)	144.46(2)	N(2)-C(5)-C(6)	107.4(2)	C(1A)-C(2A)-C(3A)	120.4(19)
N(1)-Dy(1)-Cl(1)#1	163.99(5)	C(8)-C(5)-C(6)	108.3(2)	C(7A)-C(2A)-C(3A)	117.8(15)
N(2)-Dy(1)-Cl(1)#1	104.45(5)	N(2)-C(5)-C(7)	115.2(2)	C(2A)-C(3A)-C(4A)	122.5(17)
Cl(3)-Dy(1)-Cl(1)#1	82.54(2)	C(8)-C(5)-C(7)	108.4(2)	C(3A)-C(4A)-C(5A)	118.5(17)
Cl(2)-Dy(1)-Cl(1)#1	74.00(2)	C(6)-C(5)-C(7)	108.8(2)	C(4A)-C(5A)-C(6A)	119.0(16)
N(1)-Dy(1)-Cl(1)	108.05(5)	N(3)-C(9)-C(10)	116.83(19)	C(7A)-C(6A)-C(5A)	119.9(17)
N(2)-Dy(1)-Cl(1)	168.73(5)	N(3)-C(9)-C(11)	108.7(2)	C(2A)-C(7A)-C(6A)	119.9(17)
Cl(3)-Dy(1)-Cl(1)	78.68(2)	C(10)-C(9)-C(11)	108.4(2)	C(1B)-C(2B)-C(7B)	117.1(14)
Cl(2)-Dy(1)-Cl(1)	73.13(2)	N(3)-C(9)-C(12)	106.7(2)	C(1B)-C(2B)-C(3B)	119.9(15)
Cl(1)#1-Dy(1)-Cl(1)	85.07(2)	C(10)-C(9)-C(12)	108.5(2)	C(7B)-C(2B)-C(3B)	122.9(14)
N(1)-Dy(1)-S(1)	30.64(5)	C(11)-C(9)-C(12)	107.4(2)	C(4B)-C(3B)-C(2B)	116.7(14)
N(2)-Dy(1)-S(1)	30.97(5)	N(4)-C(13)-C(16)	117.0(2)	C(3B)-C(4B)-C(5B)	120.4(15)
Cl(3)-Dy(1)-S(1)	111.339(19)	N(4)-C(13)-C(14)	107.3(2)	C(6B)-C(5B)-C(4B)	120.3(14)
Cl(2)-Dy(1)-S(1)	104.11(2)	C(16)-C(13)-C(14)	108.8(2)	C(7B)-C(6B)-C(5B)	119.4(14)
Cl(1)#1-Dy(1)-S(1)	135.01(2)	N(4)-C(13)-C(15)	107.24(19)	C(6B)-C(7B)-C(2B)	119.0(14)
Cl(1)-Dy(1)-S(1)	138.559(18)	C(16)-C(13)-C(15)	108.2(2)		

Symmetry transformations used to generate equivalent atoms:
#1 -x+1,y,-z+3/2

XRD-Analysis of 1d

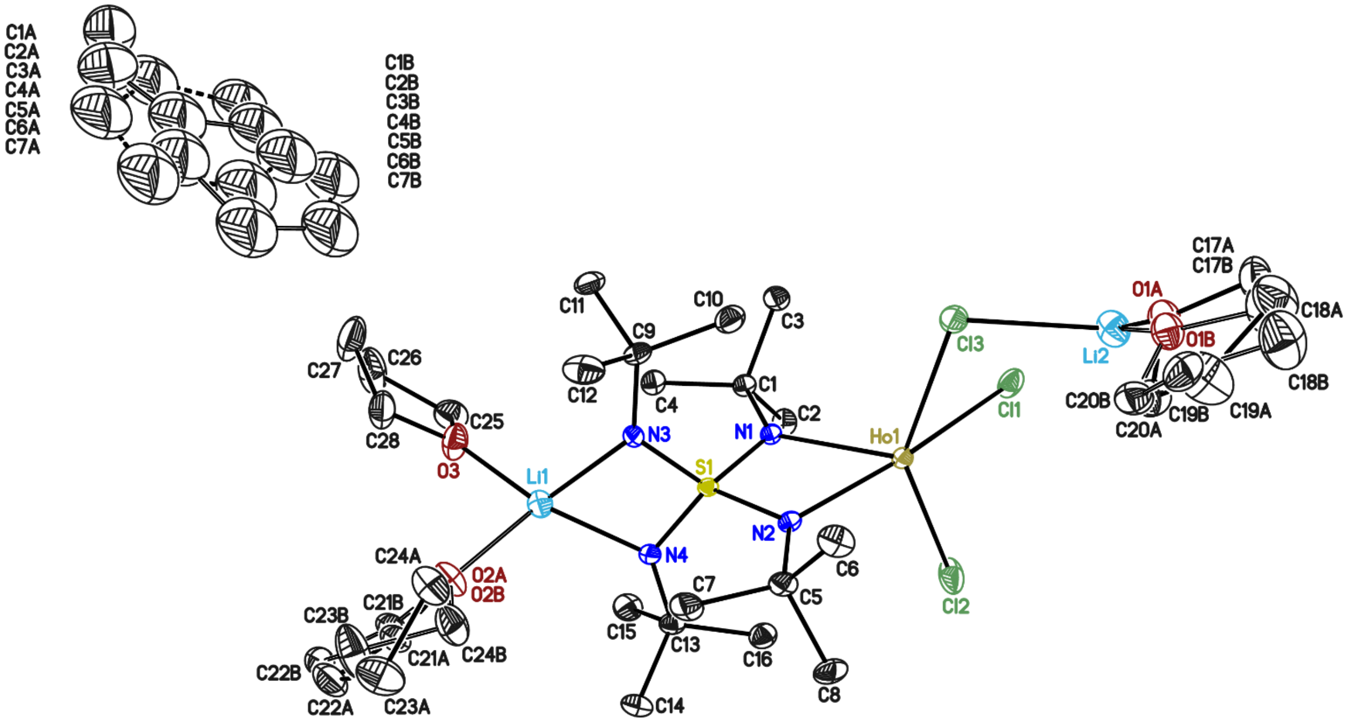

Figure S4 Asymmetric unit of **1d**. Anisotropic displacement parameters are depicted on a probability level of 50%. Hydrogen atoms are omitted for clarity. The disordered thf molecules were refined on two positions. The occupancy of the main positions for the thf molecule with O1A was refined to 0.471(19) and for the thf molecule with O2A to 0.640(16). The disordered toluene was refined on four positions, of which two each are related by a 2-fold axis. The occupancy of the main positions was refined to 0.331(6). For the refinement distance restraints and restraints for the anisotropic displacement parameters were used.

Table S10 Bond length of **1d** in Å.

**bond**	**length [Å]**	**bond**	**length [Å]**
Li(1)-N(4)	1.982(4)	O(1A)-C(20A)	1.435(11)
Li(1)-N(3)	1.989(4)	O(1A)-C(17A)	1.440(10)
Li(1)-O(2B)	2.009(4)	C(17A)-C(18A)	1.510(9)
Li(1)-O(2A)	2.009(4)	C(18A)-C(19A)	1.498(11)
Li(1)-O(3)	2.020(4)	C(19A)-C(20A)	1.494(10)
Li(1)-S(1)	2.646(4)	O(1B)-C(17B)	1.430(10)
N(1)-C(1)	1.490(3)	O(1B)-C(20B)	1.452(9)
N(1)-S(1)	1.6258(18)	C(17B)-C(18B)	1.503(8)
N(1)-Ho(1)	2.2524(17)	C(18B)-C(19B)	1.504(9)
S(1)-N(4)	1.5655(18)	C(19B)-C(20B)	1.499(9)
S(1)-N(3)	1.5688(18)	O(2A)-C(24A)	1.437(5)
S(1)-N(2)	1.6327(17)	O(2A)-C(21A)	1.446(7)
S(1)-Ho(1)	3.0759(6)	C(21A)-C(22A)	1.511(8)
Ho(1)-N(2)	2.2718(18)	C(22A)-C(23A)	1.502(8)
Ho(1)-Cl(3)	2.6321(6)	C(23A)-C(24A)	1.511(8)
Ho(1)-Cl(2)	2.6836(7)	O(2B)-C(24B)	1.440(8)
Ho(1)-Cl(1)#1	2.7465(6)	O(2B)-C(21B)	1.446(12)
Ho(1)-Cl(1)	2.7961(10)	C(21B)-C(22B)	1.504(12)
Ho(1)-Ho(1)#1	3.7665(5)	C(22B)-C(23B)	1.506(12)
C(1)-C(4)	1.527(3)	C(23B)-C(24B)	1.510(12)
C(1)-C(3)	1.527(3)	O(3)-C(28)	1.437(3)
C(1)-C(2)	1.535(3)	O(3)-C(25)	1.449(3)
N(3)-C(9)	1.483(3)	C(25)-C(26)	1.501(4)
Cl(3)-Li(2)	2.455(2)	C(26)-C(27)	1.519(5)
N(2)-C(5)	1.491(3)	C(27)-C(28)	1.515(4)
Li(2)-O(1A)	1.954(17)	C(1A)-C(2A)	1.34(3)
Li(2)-O(1A)#1	1.954(17)	C(2A)-C(7A)	1.387(14)
Li(2)-O(1B)#1	2.034(14)	C(2A)-C(3A)	1.398(14)
Li(2)-O(1B)	2.034(14)	C(3A)-C(4A)	1.395(14)
N(4)-C(13)	1.480(3)	C(4A)-C(5A)	1.407(14)
C(6)-C(5)	1.532(3)	C(5A)-C(6A)	1.409(14)
C(5)-C(7)	1.527(3)	C(6A)-C(7A)	1.407(14)
C(5)-C(8)	1.531(3)	C(1B)-C(2B)	1.32(2)
C(9)-C(10)	1.520(3)	C(2B)-C(7B)	1.401(12)
C(9)-C(12)	1.536(3)	C(2B)-C(3B)	1.406(13)
C(9)-C(11)	1.536(3)	C(3B)-C(4B)	1.392(13)
C(13)-C(16)	1.520(3)	C(4B)-C(5B)	1.411(12)
C(13)-C(14)	1.529(3)	C(5B)-C(6B)	1.395(12)
C(13)-C(15)	1.534(3)	C(6B)-C(7B)	1.374(12)

Symmetry transformations used to generate equivalent atoms:
#1 -x+1,y,-z+3/2

Table S11 Bond angles of **1d**.

N(4)-Li(1)-N(3)	72.11(15)	N(1)-Ho(1)-Ho(1)#1	137.08(5)	C(14)-C(13)-C(15)	107.83(19)
N(4)-Li(1)-O(2B)	119.0(2)	N(2)-Ho(1)-Ho(1)#1	137.91(4)	C(20A)-O(1A)-C(17A)	107.6(9)
N(3)-Li(1)-O(2B)	125.3(2)	Cl(3)-Ho(1)-Ho(1)#1	99.466(13)	C(20A)-O(1A)-Li(2)	115.7(11)
N(4)-Li(1)-O(2A)	119.0(2)	Cl(2)-Ho(1)-Ho(1)#1	45.431(14)	C(17A)-O(1A)-Li(2)	125.0(12)
N(3)-Li(1)-O(2A)	125.3(2)	Cl(1)#1-Ho(1)-Ho(1)#1	47.746(19)	O(1A)-C(17A)-C(18A)	105.0(8)
N(4)-Li(1)-O(3)	130.7(2)	Cl(1)-Ho(1)-Ho(1)#1	46.639(16)	C(19A)-C(18A)-C(17A)	106.1(7)
N(3)-Li(1)-O(3)	118.6(2)	S(1)-Ho(1)-Ho(1)#1	149.462(10)	C(20A)-C(19A)-C(18A)	105.6(7)
O(2B)-Li(1)-O(3)	94.30(17)	N(1)-C(1)-C(4)	115.52(17)	O(1A)-C(20A)-C(19A)	106.0(10)
O(2A)-Li(1)-O(3)	94.30(17)	N(1)-C(1)-C(3)	106.90(17)	C(17B)-O(1B)-C(20B)	106.2(7)
N(4)-Li(1)-S(1)	36.07(8)	C(4)-C(1)-C(3)	109.20(17)	C(17B)-O(1B)-Li(2)	126.9(11)
N(3)-Li(1)-S(1)	36.18(8)	N(1)-C(1)-C(2)	108.25(17)	C(20B)-O(1B)-Li(2)	120.7(9)
O(2B)-Li(1)-S(1)	133.49(19)	C(4)-C(1)-C(2)	108.14(17)	O(1B)-C(17B)-C(18B)	107.7(7)
O(2A)-Li(1)-S(1)	133.49(19)	C(3)-C(1)-C(2)	108.66(18)	C(17B)-C(18B)-C(19B)	105.5(7)
O(3)-Li(1)-S(1)	132.10(19)	Ho(1)#1-Cl(1)-Ho(1)	85.615(19)	C(20B)-C(19B)-C(18B)	101.7(6)
C(1)-N(1)-S(1)	125.63(14)	C(9)-N(3)-S(1)	129.30(14)	O(1B)-C(20B)-C(19B)	105.3(8)
C(1)-N(1)-Ho(1)	130.59(13)	C(9)-N(3)-Li(1)	134.64(18)	C(24A)-O(2A)-C(21A)	109.2(5)
S(1)-N(1)-Ho(1)	103.78(8)	S(1)-N(3)-Li(1)	95.36(14)	C(24A)-O(2A)-Li(1)	126.5(3)
N(4)-S(1)-N(3)	96.43(9)	Li(2)-Cl(3)-Ho(1)	99.41(14)	C(21A)-O(2A)-Li(1)	124.3(5)
N(4)-S(1)-N(1)	117.40(10)	C(5)-N(2)-S(1)	124.62(14)	O(2A)-C(21A)-C(22A)	106.8(6)
N(3)-S(1)-N(1)	117.90(9)	C(5)-N(2)-Ho(1)	132.64(13)	C(23A)-C(22A)-C(21A)	101.8(6)
N(4)-S(1)-N(2)	118.39(9)	S(1)-N(2)-Ho(1)	102.73(8)	C(22A)-C(23A)-C(24A)	103.2(6)
N(3)-S(1)-N(2)	117.22(10)	O(1A)-Li(2)-O(1A)#1	99.3(11)	O(2A)-C(24A)-C(23A)	104.6(5)
N(1)-S(1)-N(2)	91.42(9)	O(1A)-Li(2)-O(1B)#1	104.2(4)	C(24B)-O(2B)-C(21B)	106.6(10)
N(4)-S(1)-Li(1)	48.19(11)	O(1A)#1-Li(2)-O(1B)#1	5.5(13)	C(24B)-O(2B)-Li(1)	129.4(4)
N(3)-S(1)-Li(1)	48.45(11)	O(1B)#1-Li(2)-O(1B)	109.2(10)	C(21B)-O(2B)-Li(1)	119.4(8)
N(1)-S(1)-Li(1)	130.45(11)	O(1A)-Li(2)-Cl(3)	98.4(7)	O(2B)-C(21B)-C(22B)	104.6(11)
N(2)-S(1)-Li(1)	138.13(11)	O(1A)#1-Li(2)-Cl(3)	106.1(8)	C(21B)-C(22B)-C(23B)	106.2(10)
N(4)-S(1)-Ho(1)	132.24(7)	O(1B)#1-Li(2)-Cl(3)	102.6(7)	C(22B)-C(23B)-C(24B)	104.4(10)
N(3)-S(1)-Ho(1)	131.32(7)	O(1B)-Li(2)-Cl(3)	99.2(6)	O(2B)-C(24B)-C(23B)	103.3(9)
N(1)-S(1)-Ho(1)	45.33(6)	O(1A)-Li(2)-Cl(3)#1	106.1(8)	C(28)-O(3)-C(25)	108.76(18)
N(2)-S(1)-Ho(1)	46.09(6)	O(1A)#1-Li(2)-Cl(3)#1	98.4(7)	C(28)-O(3)-Li(1)	114.26(19)
Li(1)-S(1)-Ho(1)	175.76(9)	Cl(3)-Li(2)-Cl(3)#1	141.9(3)	C(25)-O(3)-Li(1)	136.71(19)
N(1)-Ho(1)-N(2)	62.07(6)	Ho(1)-Cl(2)-Ho(1)#1	89.14(3)	O(3)-C(25)-C(26)	107.2(2)
N(1)-Ho(1)-Cl(3)	108.29(5)	C(13)-N(4)-S(1)	130.34(15)	C(25)-C(26)-C(27)	102.5(2)
N(2)-Ho(1)-Cl(3)	107.77(5)	C(13)-N(4)-Li(1)	133.77(18)	C(28)-C(27)-C(26)	102.1(2)
N(1)-Ho(1)-Cl(2)	100.42(5)	S(1)-N(4)-Li(1)	95.74(14)	O(3)-C(28)-C(27)	105.4(2)
N(2)-Ho(1)-Cl(2)	103.43(5)	N(2)-C(5)-C(7)	115.68(18)	C(1A)-C(2A)-C(7A)	122.7(19)
Cl(3)-Ho(1)-Cl(2)	144.796(19)	N(2)-C(5)-C(8)	108.25(18)	C(1A)-C(2A)-C(3A)	120.3(19)
N(1)-Ho(1)-Cl(1)#1	164.17(5)	C(7)-C(5)-C(8)	108.62(19)	C(7A)-C(2A)-C(3A)	116.6(15)
N(2)-Ho(1)-Cl(1)#1	104.20(4)	N(2)-C(5)-C(6)	107.30(17)	C(4A)-C(3A)-C(2A)	122.5(16)
Cl(3)-Ho(1)-Cl(1)#1	82.706(19)	C(7)-C(5)-C(6)	108.66(19)	C(3A)-C(4A)-C(5A)	119.6(17)
Cl(2)-Ho(1)-Cl(1)#1	74.111(18)	C(8)-C(5)-C(6)	108.10(18)	C(4A)-C(5A)-C(6A)	117.6(16)
N(1)-Ho(1)-Cl(1)	107.27(5)	N(3)-C(9)-C(10)	116.50(18)	C(7A)-C(6A)-C(5A)	119.4(17)
N(2)-Ho(1)-Cl(1)	168.52(4)	N(3)-C(9)-C(12)	106.64(18)	C(2A)-C(7A)-C(6A)	120.0(17)
Cl(3)-Ho(1)-Cl(1)	78.94(2)	C(10)-C(9)-C(12)	108.84(19)	C(1B)-C(2B)-C(7B)	116.3(13)
Cl(2)-Ho(1)-Cl(1)	73.308(19)	N(3)-C(9)-C(11)	108.41(17)	C(1B)-C(2B)-C(3B)	121.3(14)
Cl(1)#1-Ho(1)-Cl(1)	85.67(2)	C(10)-C(9)-C(11)	108.44(19)	C(7B)-C(2B)-C(3B)	122.3(13)
N(1)-Ho(1)-S(1)	30.89(4)	C(12)-C(9)-C(11)	107.69(19)	C(4B)-C(3B)-C(2B)	118.2(13)
N(2)-Ho(1)-S(1)	31.18(4)	N(4)-C(13)-C(16)	117.10(18)	C(3B)-C(4B)-C(5B)	119.7(14)
Cl(3)-Ho(1)-S(1)	111.069(17)	N(4)-C(13)-C(14)	107.33(18)	C(6B)-C(5B)-C(4B)	119.1(14)
Cl(2)-Ho(1)-S(1)	104.048(18)	C(16)-C(13)-C(14)	109.02(19)	C(7B)-C(6B)-C(5B)	121.9(13)
Cl(1)#1-Ho(1)-S(1)	134.96(2)	N(4)-C(13)-C(15)	107.11(17)	C(6B)-C(7B)-C(2B)	117.7(13)
Cl(1)-Ho(1)-S(1)	138.034(17)	C(16)-C(13)-C(15)	108.12(19)		

Symmetry transformations used to generate equivalent atoms:
#1 -x+1,y,-z+3/2

XRD-Analysis of 1e

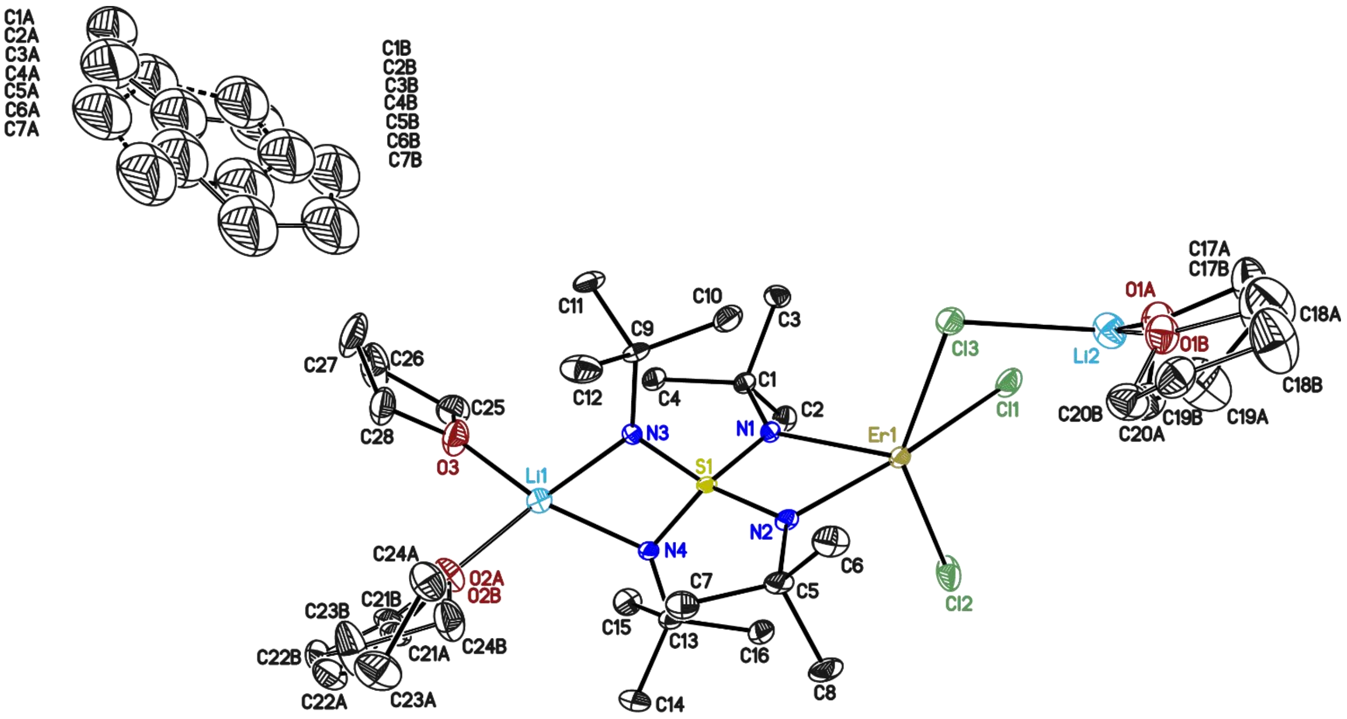

Figure S5 Asymmetric unit of **1e**. Anisotropic displacement parameters are depicted on a probability level of 50%. Hydrogen atoms are omitted for clarity. The disordered thf molecules were refined on two positions. The occupancy of the main positions for the thf molecule with O1A was refined to 0.41(2) and for the thf molecule with O2A to 0.63(2). The disordered toluene was refined on four positions, of which two each are related by a 2-fold axis. The occupancy of the main positions was refined to 0.312(7). For the refinement distance restraints and restraints for the anisotropic displacement parameters were used.

Table S12 Bond length of **1e** in Å.

**bond**	**length [Å]**	**bond**	**length [Å]**
Li(1)-N(4)	1.979(5)	O(1A)-C(20A)	1.431(12)
Li(1)-N(3)	1.989(5)	O(1A)-C(17A)	1.437(12)
Li(1)-O(2B)	2.008(5)	C(17A)-C(18A)	1.511(11)
Li(1)-O(2A)	2.008(5)	C(18A)-C(19A)	1.491(12)
Li(1)-O(3)	2.023(5)	C(19A)-C(20A)	1.504(11)
Li(1)-S(1)	2.648(5)	O(1B)-C(17B)	1.427(9)
N(1)-C(1)	1.490(3)	O(1B)-C(20B)	1.444(9)
N(1)-S(1)	1.625(2)	C(17B)-C(18B)	1.508(9)
N(1)-Er(1)	2.241(2)	C(18B)-C(19B)	1.495(9)
S(1)-N(4)	1.567(2)	C(19B)-C(20B)	1.500(9)
S(1)-N(3)	1.569(2)	O(2A)-C(24A)	1.439(6)
S(1)-N(2)	1.628(2)	O(2A)-C(21A)	1.447(8)
S(1)-Er(1)	3.0643(7)	C(21A)-C(22A)	1.508(8)
Er(1)-N(2)	2.264(2)	C(22A)-C(23A)	1.498(9)
Er(1)-Cl(3)	2.6171(8)	C(23A)-C(24A)	1.506(9)
Er(1)-Cl(2)	2.6729(8)	O(2B)-C(24B)	1.435(9)
Er(1)-Cl(1)#1	2.7365(7)	O(2B)-C(21B)	1.445(12)
Er(1)-Cl(1)	2.7835(11)	C(21B)-C(22B)	1.502(12)
Er(1)-Er(1)#1	3.7517(5)	C(22B)-C(23B)	1.493(12)
C(1)-C(4)	1.527(4)	C(23B)-C(24B)	1.506(12)
C(1)-C(2)	1.531(4)	O(3)-C(28)	1.437(4)
C(1)-C(3)	1.531(4)	O(3)-C(25)	1.447(4)
N(3)-C(9)	1.481(3)	C(25)-C(26)	1.502(5)
Cl(3)-Li(2)	2.454(3)	C(26)-C(27)	1.510(6)
N(2)-C(5)	1.495(3)	C(27)-C(28)	1.516(5)
Li(2)-O(1A)	1.93(2)	C(1A)-C(2A)	1.34(3)
Li(2)-O(1A)#1	1.93(2)	C(2A)-C(7A)	1.390(14)
Li(2)-O(1B)	2.040(14)	C(2A)-C(3A)	1.406(14)
Li(2)-O(1B)#1	2.040(14)	C(3A)-C(4A)	1.400(14)
N(4)-C(13)	1.475(3)	C(4A)-C(5A)	1.406(15)
C(6)-C(5)	1.531(4)	C(5A)-C(6A)	1.409(15)
C(5)-C(7)	1.526(4)	C(6A)-C(7A)	1.409(15)
C(5)-C(8)	1.531(4)	C(1B)-C(2B)	1.33(3)
C(9)-C(10)	1.521(4)	C(2B)-C(3B)	1.400(14)
C(9)-C(11)	1.533(4)	C(2B)-C(7B)	1.407(13)
C(9)-C(12)	1.542(4)	C(3B)-C(4B)	1.399(14)
C(13)-C(16)	1.526(4)	C(4B)-C(5B)	1.410(14)
C(13)-C(14)	1.532(4)	C(5B)-C(6B)	1.395(13)
C(13)-C(15)	1.535(4)	C(6B)-C(7B)	1.384(13)

Symmetry transformations used to generate equivalent atoms:
#1 -x+1,y,-z+3/2

Table S13 Bond angles of **1e**.

N(4)-Li(1)-N(3)	72.11(18)	N(1)-Er(1)-Er(1)#1	136.99(6)	C(14)-C(13)-C(15)	107.9(2)
N(4)-Li(1)-O(2B)	119.3(2)	N(2)-Er(1)-Er(1)#1	137.98(5)	C(20A)-O(1A)-C(17A)	107.2(11)
N(3)-Li(1)-O(2B)	125.3(3)	Cl(3)-Er(1)-Er(1)#1	99.606(16)	C(20A)-O(1A)-Li(2)	116.0(14)
N(4)-Li(1)-O(2A)	119.3(2)	Cl(2)-Er(1)-Er(1)#1	45.430(16)	C(17A)-O(1A)-Li(2)	124.0(14)
N(3)-Li(1)-O(2A)	125.3(3)	Cl(1)#1-Er(1)-Er(1)#1	47.71(2)	O(1A)-C(17A)-C(18A)	105.1(10)
N(4)-Li(1)-O(3)	130.7(3)	Cl(1)-Er(1)-Er(1)#1	46.659(17)	C(19A)-C(18A)-C(17A)	106.2(8)
N(3)-Li(1)-O(3)	118.6(2)	S(1)-Er(1)-Er(1)#1	149.511(12)	C(18A)-C(19A)-C(20A)	105.3(9)
O(2B)-Li(1)-O(3)	94.2(2)	N(1)-C(1)-C(4)	115.7(2)	O(1A)-C(20A)-C(19A)	106.5(11)
O(2A)-Li(1)-O(3)	94.2(2)	N(1)-C(1)-C(2)	108.5(2)	C(17B)-O(1B)-C(20B)	107.1(7)
N(4)-Li(1)-S(1)	36.08(10)	C(4)-C(1)-C(2)	108.1(2)	C(17B)-O(1B)-Li(2)	127.3(10)
N(3)-Li(1)-S(1)	36.16(10)	N(1)-C(1)-C(3)	106.9(2)	C(20B)-O(1B)-Li(2)	120.2(8)
O(2B)-Li(1)-S(1)	133.6(2)	C(4)-C(1)-C(3)	109.0(2)	O(1B)-C(17B)-C(18B)	107.8(7)
O(2A)-Li(1)-S(1)	133.6(2)	C(2)-C(1)-C(3)	108.4(2)	C(19B)-C(18B)-C(17B)	105.0(6)
O(3)-Li(1)-S(1)	132.1(2)	Er(1)#1-Cl(1)-Er(1)	85.63(2)	C(18B)-C(19B)-C(20B)	103.2(6)
C(1)-N(1)-S(1)	125.26(17)	C(9)-N(3)-S(1)	129.13(18)	O(1B)-C(20B)-C(19B)	105.1(7)
C(1)-N(1)-Er(1)	131.00(16)	C(9)-N(3)-Li(1)	134.7(2)	C(24A)-O(2A)-C(21A)	109.4(5)
S(1)-N(1)-Er(1)	103.74(10)	S(1)-N(3)-Li(1)	95.45(17)	C(24A)-O(2A)-Li(1)	126.7(3)
N(4)-S(1)-N(3)	96.24(11)	Li(2)-Cl(3)-Er(1)	99.49(17)	C(21A)-O(2A)-Li(1)	123.9(5)
N(4)-S(1)-N(1)	117.50(12)	C(5)-N(2)-S(1)	124.66(18)	O(2A)-C(21A)-C(22A)	106.1(7)
N(3)-S(1)-N(1)	118.02(11)	C(5)-N(2)-Er(1)	132.68(16)	C(23A)-C(22A)-C(21A)	102.4(7)
N(4)-S(1)-N(2)	118.40(12)	S(1)-N(2)-Er(1)	102.65(10)	C(22A)-C(23A)-C(24A)	102.9(7)
N(3)-S(1)-N(2)	117.28(12)	O(1A)-Li(2)-O(1A)#1	98.7(13)	O(2A)-C(24A)-C(23A)	104.6(6)
N(1)-S(1)-N(2)	91.37(11)	O(1A)-Li(2)-O(1B)#1	103.7(5)	C(24B)-O(2B)-C(21B)	106.7(10)
N(4)-S(1)-Li(1)	48.04(13)	O(1A)#1-Li(2)-O(1B)#1	5.6(13)	C(24B)-O(2B)-Li(1)	129.5(5)
N(3)-S(1)-Li(1)	48.39(13)	O(1B)-Li(2)-O(1B)#1	108.9(9)	C(21B)-O(2B)-Li(1)	119.7(8)
N(1)-S(1)-Li(1)	130.68(13)	O(1A)-Li(2)-Cl(3)#1	106.5(10)	O(2B)-C(21B)-C(22B)	105.5(11)
N(2)-S(1)-Li(1)	137.95(13)	O(1A)#1-Li(2)-Cl(3)#1	98.4(9)	C(23B)-C(22B)-C(21B)	105.6(10)
N(4)-S(1)-Er(1)	132.28(8)	O(1A)-Li(2)-Cl(3)	98.4(9)	C(22B)-C(23B)-C(24B)	105.6(10)
N(3)-S(1)-Er(1)	131.48(8)	O(1A)#1-Li(2)-Cl(3)	106.5(10)	O(2B)-C(24B)-C(23B)	103.9(9)
N(1)-S(1)-Er(1)	45.26(7)	O(1B)-Li(2)-Cl(3)	99.1(6)	C(28)-O(3)-C(25)	108.8(2)
N(2)-S(1)-Er(1)	46.11(8)	O(1B)#1-Li(2)-Cl(3)	103.0(7)	C(28)-O(3)-Li(1)	114.5(2)
Li(1)-S(1)-Er(1)	175.93(11)	Cl(3)#1-Li(2)-Cl(3)	141.4(3)	C(25)-O(3)-Li(1)	136.5(2)
N(1)-Er(1)-N(2)	62.24(7)	Er(1)-Cl(2)-Er(1)#1	89.14(3)	O(3)-C(25)-C(26)	107.2(3)
N(1)-Er(1)-Cl(3)	108.15(6)	C(13)-N(4)-S(1)	130.43(18)	C(25)-C(26)-C(27)	102.4(3)
N(2)-Er(1)-Cl(3)	107.53(6)	C(13)-N(4)-Li(1)	133.5(2)	C(26)-C(27)-C(28)	102.3(3)
N(1)-Er(1)-Cl(2)	100.42(6)	S(1)-N(4)-Li(1)	95.88(17)	O(3)-C(28)-C(27)	105.2(3)
N(2)-Er(1)-Cl(2)	103.51(6)	N(2)-C(5)-C(7)	115.5(2)	C(1A)-C(2A)-C(7A)	122.4(19)
Cl(3)-Er(1)-Cl(2)	144.94(2)	N(2)-C(5)-C(8)	108.3(2)	C(1A)-C(2A)-C(3A)	119.8(19)
N(1)-Er(1)-Cl(1)#1	164.28(6)	C(7)-C(5)-C(8)	108.6(2)	C(7A)-C(2A)-C(3A)	117.6(15)
N(2)-Er(1)-Cl(1)#1	104.21(6)	N(2)-C(5)-C(6)	107.3(2)	C(4A)-C(3A)-C(2A)	121.3(16)
Cl(3)-Er(1)-Cl(1)#1	82.83(2)	C(7)-C(5)-C(6)	108.9(2)	C(3A)-C(4A)-C(5A)	120.0(17)
Cl(2)-Er(1)-Cl(1)#1	74.04(2)	C(8)-C(5)-C(6)	108.0(2)	C(4A)-C(5A)-C(6A)	117.8(16)
N(1)-Er(1)-Cl(1)	107.03(6)	N(3)-C(9)-C(10)	116.9(2)	C(5A)-C(6A)-C(7A)	119.9(17)
N(2)-Er(1)-Cl(1)	168.47(6)	N(3)-C(9)-C(11)	108.6(2)	C(2A)-C(7A)-C(6A)	119.9(17)
Cl(3)-Er(1)-Cl(1)	79.15(2)	C(10)-C(9)-C(11)	108.5(2)	C(1B)-C(2B)-C(3B)	122.1(16)
Cl(2)-Er(1)-Cl(1)	73.27(2)	N(3)-C(9)-C(12)	106.5(2)	C(1B)-C(2B)-C(7B)	116.4(15)
Cl(1)#1-Er(1)-Cl(1)	85.73(3)	C(10)-C(9)-C(12)	108.6(2)	C(3B)-C(2B)-C(7B)	121.5(14)
N(1)-Er(1)-S(1)	31.00(5)	C(11)-C(9)-C(12)	107.4(2)	C(4B)-C(3B)-C(2B)	119.6(15)
N(2)-Er(1)-S(1)	31.23(5)	N(4)-C(13)-C(16)	117.1(2)	C(3B)-C(4B)-C(5B)	118.6(15)
Cl(3)-Er(1)-S(1)	110.88(2)	N(4)-C(13)-C(14)	107.4(2)	C(6B)-C(5B)-C(4B)	119.6(15)
Cl(2)-Er(1)-S(1)	104.10(2)	C(16)-C(13)-C(14)	108.6(2)	C(7B)-C(6B)-C(5B)	121.7(14)
Cl(1)#1-Er(1)-S(1)	135.01(2)	N(4)-C(13)-C(15)	107.3(2)	C(6B)-C(7B)-C(2B)	117.8(15)
Cl(1)-Er(1)-S(1)	137.91(2)	C(16)-C(13)-C(15)	108.1(2)		

Symmetry transformations used to generate equivalent atoms:
#1 -x+1,y,-z+3/2

XRD-Analysis of 2a

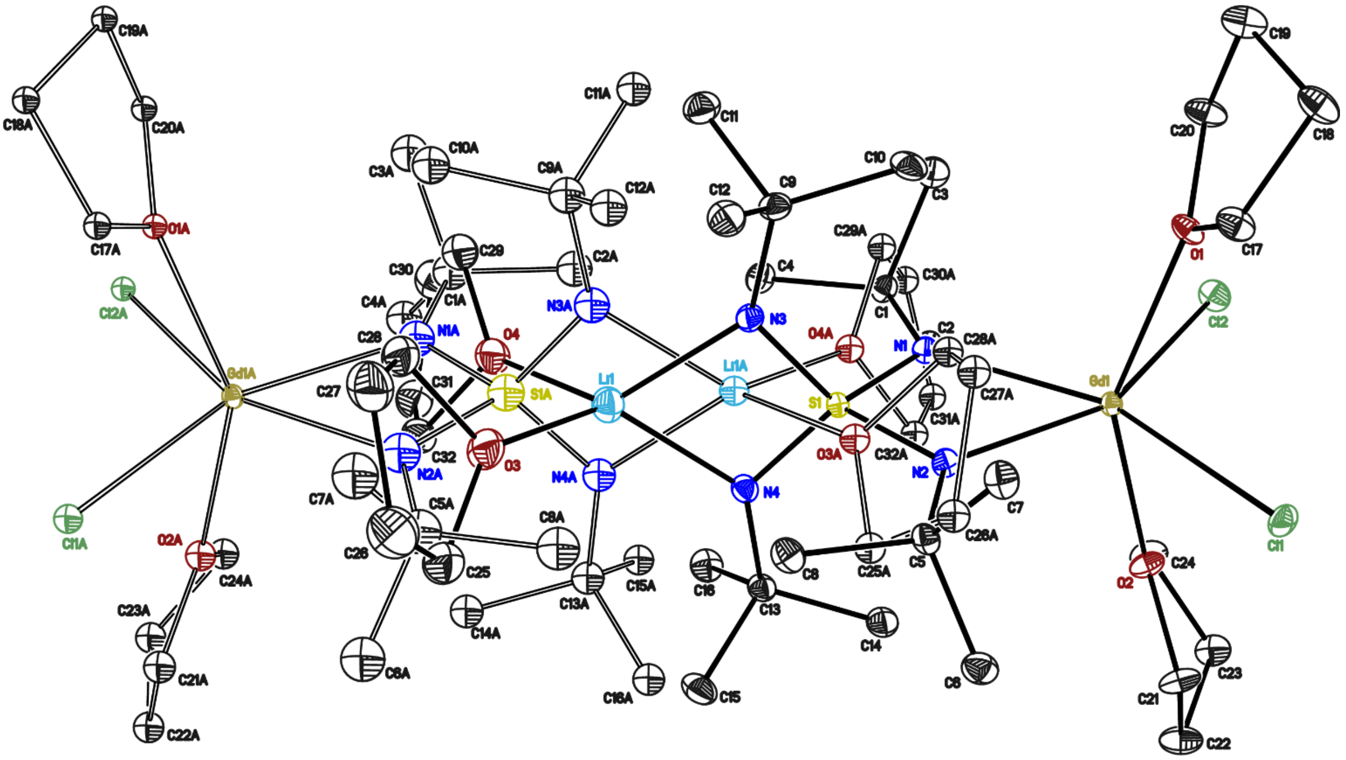

Figure S6 Asymmetric unit of **2a**. Anisotropic displacement parameters are depicted on a probability level of 50%. Hydrogen atoms are omitted for clarity. The whole molecule is disordered and was refined on two positions. The occupancy of the main positions was refined to 0.9628(5). For the refinement, distance restraints and restraints for the anisotropic displacement parameters were used. The structure was refined as an inversion twin.

Table S14 Bond length of **2a** in Å.

**bond**	**length [Å]**	**bond**	**length [Å]**
Gd(1)-N(2)	2.351(3)	Gd(1A)-N(1A)	2.356(10)
Gd(1)-N(1)	2.356(3)	Gd(1A)-N(2A)	2.361(10)
Gd(1)-O(1)	2.436(3)	Gd(1A)-O(2A)	2.431(9)
Gd(1)-O(2)	2.438(3)	Gd(1A)-O(1A)	2.441(9)
Gd(1)-Cl(2)	2.6645(17)	Gd(1A)-Cl(2A)	2.661(10)
Gd(1)-Cl(1)	2.6786(15)	Gd(1A)-Cl(1A)	2.676(10)
Gd(1)-S(1)	3.1571(9)	Gd(1A)-S(1A)	3.168(9)
Li(1)-O(3)	1.973(6)	Li(1A)-O(4A)	1.974(11)
Li(1)-O(4)	1.976(6)	Li(1A)-N(3A)	1.980(12)
Li(1)-N(3)	1.978(7)	Li(1A)-O(3A)	1.981(11)
Li(1)-N(4)	1.985(7)	Li(1A)-N(4A)	1.986(12)
Li(1)-S(1)	2.641(6)	Li(1A)-S(1A)	2.640(12)
S(1)-N(4)	1.562(4)	S(1A)-N(4A)	1.562(10)
S(1)-N(3)	1.567(4)	S(1A)-N(3A)	1.562(10)
S(1)-N(1)	1.626(3)	S(1A)-N(1A)	1.628(10)
S(1)-N(2)	1.633(3)	S(1A)-N(2A)	1.632(10)
N(1)-C(1)	1.498(5)	N(1A)-C(1A)	1.489(10)
C(1)-C(2)	1.532(5)	C(1A)-C(2A)	1.533(10)
C(1)-C(3)	1.538(5)	C(1A)-C(4A)	1.535(10)
C(1)-C(4)	1.540(5)	C(1A)-C(3A)	1.535(10)
N(2)-C(5)	1.494(5)	N(2A)-C(5A)	1.488(10)
C(5)-C(6)	1.531(5)	C(5A)-C(8A)	1.533(10)
C(5)-C(7)	1.537(5)	C(5A)-C(6A)	1.536(10)
C(5)-C(8)	1.538(5)	C(5A)-C(7A)	1.537(10)
N(3)-C(9)	1.478(5)	N(3A)-C(9A)	1.485(10)
C(9)-C(10)	1.530(5)	C(9A)-C(10A)	1.534(10)
C(9)-C(11)	1.534(5)	C(9A)-C(11A)	1.535(10)
C(9)-C(12)	1.538(5)	C(9A)-C(12A)	1.535(10)
N(4)-C(13)	1.478(5)	N(4A)-C(13A)	1.484(10)
C(13)-C(14)	1.531(5)	C(13A)-C(14A)	1.532(10)
C(13)-C(16)	1.535(5)	C(13A)-C(15A)	1.536(10)
C(13)-C(15)	1.535(5)	C(13A)-C(16A)	1.537(10)
O(1)-C(17)	1.460(5)	O(1A)-C(20A)	1.445(10)
O(1)-C(20)	1.464(5)	O(1A)-C(17A)	1.446(10)
C(17)-C(18)	1.508(5)	C(17A)-C(18A)	1.511(11)
C(18)-C(19)	1.519(6)	C(18A)-C(19A)	1.526(12)
C(19)-C(20)	1.517(6)	C(19A)-C(20A)	1.512(11)
O(2)-C(24)	1.458(4)	O(2A)-C(21A)	1.446(10)
O(2)-C(21)	1.467(5)	O(2A)-C(24A)	1.448(10)
C(21)-C(22)	1.512(6)	C(21A)-C(22A)	1.509(11)
C(22)-C(23)	1.526(6)	C(22A)-C(23A)	1.525(12)
C(23)-C(24)	1.508(5)	C(23A)-C(24A)	1.513(11)
O(3)-C(25)	1.434(5)	O(3A)-C(25A)	1.447(10)
O(3)-C(28)	1.442(5)	O(3A)-C(28A)	1.448(10)
C(25)-C(26)	1.516(6)	C(25A)-C(26A)	1.505(11)
C(26)-C(27)	1.528(6)	C(26A)-C(27A)	1.521(12)
C(27)-C(28)	1.512(6)	C(27A)-C(28A)	1.513(11)
O(4)-C(29)	1.436(5)	O(4A)-C(29A)	1.448(10)
O(4)-C(32)	1.437(5)	O(4A)-C(32A)	1.449(10)
C(29)-C(30)	1.514(6)	C(29A)-C(30A)	1.509(11)
C(30)-C(31)	1.531(6)	C(30A)-C(31A)	1.518(11)
C(31)-C(32)	1.526(6)	C(31A)-C(32A)	1.507(11)

Table S15 Bond angles of **2a**.

N(2)-Gd(1)-N(1)	60.27(10)	C(13)-N(4)-Li(1)	135.7(3)	N(3A)-S(1A)-Li(1A)	48.3(4)
N(2)-Gd(1)-O(1)	102.14(11)	S(1)-N(4)-Li(1)	95.5(2)	N(1A)-S(1A)-Li(1A)	134.7(8)
N(1)-Gd(1)-O(1)	103.66(11)	N(4)-C(13)-C(14)	116.3(3)	N(2A)-S(1A)-Li(1A)	132.6(8)
N(2)-Gd(1)-O(2)	104.09(11)	N(4)-C(13)-C(16)	109.5(3)	N(4A)-S(1A)-Gd(1A)	131.7(8)
N(1)-Gd(1)-O(2)	102.63(11)	C(14)-C(13)-C(16)	108.7(4)	N(3A)-S(1A)-Gd(1A)	131.5(8)
O(1)-Gd(1)-O(2)	149.55(8)	N(4)-C(13)-C(15)	106.9(3)	N(1A)-S(1A)-Gd(1A)	46.2(4)
N(2)-Gd(1)-Cl(2)	155.82(9)	C(14)-C(13)-C(15)	108.4(4)	N(2A)-S(1A)-Gd(1A)	46.4(4)
N(1)-Gd(1)-Cl(2)	95.74(8)	C(16)-C(13)-C(15)	106.6(4)	Li(1A)-S(1A)-Gd(1A)	179.1(8)
O(1)-Gd(1)-Cl(2)	79.38(9)	C(17)-O(1)-C(20)	108.7(3)	C(1A)-N(1A)-S(1A)	124.0(12)
O(2)-Gd(1)-Cl(2)	82.97(8)	C(17)-O(1)-Gd(1)	124.6(2)	C(1A)-N(1A)-Gd(1A)	132.1(12)
N(2)-Gd(1)-Cl(1)	96.03(8)	C(20)-O(1)-Gd(1)	126.6(2)	S(1A)-N(1A)-Gd(1A)	103.9(6)
N(1)-Gd(1)-Cl(1)	156.18(8)	O(1)-C(17)-C(18)	105.1(3)	N(1A)-C(1A)-C(2A)	111.9(14)
O(1)-Gd(1)-Cl(1)	82.50(8)	C(17)-C(18)-C(19)	102.0(4)	N(1A)-C(1A)-C(4A)	110.3(14)
O(2)-Gd(1)-Cl(1)	79.63(8)	C(20)-C(19)-C(18)	101.8(4)	C(2A)-C(1A)-C(4A)	107.9(14)
Cl(2)-Gd(1)-Cl(1)	108.05(4)	O(1)-C(20)-C(19)	105.5(3)	N(1A)-C(1A)-C(3A)	110.7(14)
N(2)-Gd(1)-S(1)	30.21(8)	C(24)-O(2)-C(21)	108.6(3)	C(2A)-C(1A)-C(3A)	108.1(14)
N(1)-Gd(1)-S(1)	30.06(8)	C(24)-O(2)-Gd(1)	124.8(2)	C(4A)-C(1A)-C(3A)	107.9(14)
O(1)-Gd(1)-S(1)	104.90(9)	C(21)-O(2)-Gd(1)	126.4(2)	C(5A)-N(2A)-S(1A)	124.0(13)
O(2)-Gd(1)-S(1)	105.55(9)	O(2)-C(21)-C(22)	105.6(4)	C(5A)-N(2A)-Gd(1A)	132.5(12)
Cl(2)-Gd(1)-S(1)	125.74(5)	C(21)-C(22)-C(23)	102.2(4)	S(1A)-N(2A)-Gd(1A)	103.5(6)
Cl(1)-Gd(1)-S(1)	126.21(5)	C(24)-C(23)-C(22)	101.5(4)	N(2A)-C(5A)-C(8A)	111.7(14)
O(3)-Li(1)-O(4)	93.0(3)	O(2)-C(24)-C(23)	105.5(3)	N(2A)-C(5A)-C(6A)	111.0(14)
O(3)-Li(1)-N(3)	117.1(4)	C(25)-O(3)-C(28)	105.5(3)	C(8A)-C(5A)-C(6A)	108.0(14)
O(4)-Li(1)-N(3)	128.7(3)	C(25)-O(3)-Li(1)	135.4(3)	N(2A)-C(5A)-C(7A)	111.0(14)
O(3)-Li(1)-N(4)	130.4(3)	C(28)-O(3)-Li(1)	118.9(3)	C(8A)-C(5A)-C(7A)	107.6(14)
O(4)-Li(1)-N(4)	119.5(4)	O(3)-C(25)-C(26)	105.1(4)	C(6A)-C(5A)-C(7A)	107.3(13)
N(3)-Li(1)-N(4)	72.3(2)	C(25)-C(26)-C(27)	105.2(4)	C(9A)-N(3A)-S(1A)	129.8(13)
O(3)-Li(1)-S(1)	133.2(3)	C(28)-C(27)-C(26)	103.8(4)	C(9A)-N(3A)-Li(1A)	134.4(13)
O(4)-Li(1)-S(1)	133.8(3)	O(3)-C(28)-C(27)	104.8(4)	S(1A)-N(3A)-Li(1A)	95.6(6)
N(3)-Li(1)-S(1)	36.19(14)	C(29)-O(4)-C(32)	104.8(3)	N(3A)-C(9A)-C(10A)	111.2(14)
N(4)-Li(1)-S(1)	36.07(14)	C(29)-O(4)-Li(1)	134.6(3)	N(3A)-C(9A)-C(11A)	110.9(14)
N(4)-S(1)-N(3)	96.61(16)	C(32)-O(4)-Li(1)	120.5(3)	C(10A)-C(9A)-C(11A)	108.0(14)
N(4)-S(1)-N(1)	117.6(2)	O(4)-C(29)-C(30)	105.8(3)	N(3A)-C(9A)-C(12A)	111.1(14)
N(3)-S(1)-N(1)	116.91(18)	C(29)-C(30)-C(31)	104.7(4)	C(10A)-C(9A)-C(12A)	107.8(13)
N(4)-S(1)-N(2)	117.00(18)	C(32)-C(31)-C(30)	103.5(4)	C(11A)-C(9A)-C(12A)	107.6(13)
N(3)-S(1)-N(2)	117.5(2)	O(4)-C(32)-C(31)	104.4(3)	C(13A)-N(4A)-S(1A)	131.1(13)
N(1)-S(1)-N(2)	92.98(14)	N(1A)-Gd(1A)-N(2A)	60.0(4)	C(13A)-N(4A)-Li(1A)	133.4(13)
N(4)-S(1)-Li(1)	48.42(18)	N(1A)-Gd(1A)-O(2A)	102.5(7)	S(1A)-N(4A)-Li(1A)	95.4(6)
N(3)-S(1)-Li(1)	48.19(17)	N(2A)-Gd(1A)-O(2A)	103.8(7)	N(4A)-C(13A)-C(14A)	112.3(14)
N(1)-S(1)-Li(1)	133.48(19)	N(1A)-Gd(1A)-O(1A)	103.7(7)	N(4A)-C(13A)-C(15A)	110.9(14)
N(2)-S(1)-Li(1)	133.55(19)	N(2A)-Gd(1A)-O(1A)	101.4(7)	C(14A)-C(13A)-C(15A)	107.8(13)
N(4)-S(1)-Gd(1)	131.85(15)	O(2A)-Gd(1A)-O(1A)	150.2(8)	N(4A)-C(13A)-C(16A)	110.7(14)
N(3)-S(1)-Gd(1)	131.54(15)	N(1A)-Gd(1A)-Cl(2A)	96.9(6)	C(14A)-C(13A)-C(16A)	107.6(14)
N(1)-S(1)-Gd(1)	46.55(11)	N(2A)-Gd(1A)-Cl(2A)	156.6(6)	C(15A)-C(13A)-C(16A)	107.2(13)
N(2)-S(1)-Gd(1)	46.43(11)	O(2A)-Gd(1A)-Cl(2A)	83.5(6)	C(20A)-O(1A)-C(17A)	109.0(10)
Li(1)-S(1)-Gd(1)	179.73(18)	O(1A)-Gd(1A)-Cl(2A)	79.7(6)	C(20A)-O(1A)-Gd(1A)	125.3(13)
C(1)-N(1)-S(1)	122.5(3)	N(1A)-Gd(1A)-Cl(1A)	155.3(6)	C(17A)-O(1A)-Gd(1A)	125.4(13)
C(1)-N(1)-Gd(1)	134.1(2)	N(2A)-Gd(1A)-Cl(1A)	95.4(5)	O(1A)-C(17A)-C(18A)	106.9(13)
S(1)-N(1)-Gd(1)	103.40(15)	O(2A)-Gd(1A)-Cl(1A)	79.5(6)	C(17A)-C(18A)-C(19A)	102.6(13)
N(1)-C(1)-C(2)	107.8(3)	O(1A)-Gd(1A)-Cl(1A)	82.5(6)	C(20A)-C(19A)-C(18A)	102.5(14)
N(1)-C(1)-C(3)	108.8(3)	Cl(2A)-Gd(1A)-Cl(1A)	107.8(7)	O(1A)-C(20A)-C(19A)	106.6(13)
C(2)-C(1)-C(3)	108.3(4)	N(1A)-Gd(1A)-S(1A)	29.9(3)	C(21A)-O(2A)-C(24A)	108.5(12)
N(1)-C(1)-C(4)	116.2(3)	N(2A)-Gd(1A)-S(1A)	30.1(3)	C(21A)-O(2A)-Gd(1A)	125.8(13)
C(2)-C(1)-C(4)	108.2(4)	O(2A)-Gd(1A)-S(1A)	105.3(6)	C(24A)-O(2A)-Gd(1A)	125.6(13)
C(3)-C(1)-C(4)	107.3(4)	O(1A)-Gd(1A)-S(1A)	104.5(6)	O(2A)-C(21A)-C(22A)	107.5(10)
C(5)-N(2)-S(1)	122.7(3)	Cl(2A)-Gd(1A)-S(1A)	126.7(5)	C(21A)-C(22A)-C(23A)	103.8(14)
C(5)-N(2)-Gd(1)	133.9(2)	Cl(1A)-Gd(1A)-S(1A)	125.4(5)	C(24A)-C(23A)-C(22A)	102.2(14)
S(1)-N(2)-Gd(1)	103.36(15)	O(4A)-Li(1A)-N(3A)	130.6(13)	O(2A)-C(24A)-C(23A)	104.9(14)
N(2)-C(5)-C(6)	109.3(3)	O(4A)-Li(1A)-O(3A)	93.1(10)	C(25A)-O(3A)-C(28A)	107.5(12)
N(2)-C(5)-C(7)	108.2(3)	N(3A)-Li(1A)-O(3A)	118.1(11)	C(25A)-O(3A)-Li(1A)	133.0(16)
C(6)-C(5)-C(7)	107.4(4)	O(4A)-Li(1A)-N(4A)	118.0(11)	C(28A)-O(3A)-Li(1A)	119.1(14)
N(2)-C(5)-C(8)	116.1(4)	N(3A)-Li(1A)-N(4A)	72.2(6)	O(3A)-C(25A)-C(26A)	107.9(11)
C(6)-C(5)-C(8)	107.6(4)	O(3A)-Li(1A)-N(4A)	129.2(13)	C(25A)-C(26A)-C(27A)	105.0(11)
C(7)-C(5)-C(8)	108.0(4)	O(4A)-Li(1A)-S(1A)	134.1(9)	C(28A)-C(27A)-C(26A)	103.3(14)
C(9)-N(3)-S(1)	129.1(3)	N(3A)-Li(1A)-S(1A)	36.1(3)	O(3A)-C(28A)-C(27A)	105.0(14)
C(9)-N(3)-Li(1)	135.2(3)	O(3A)-Li(1A)-S(1A)	132.8(9)	C(29A)-O(4A)-C(32A)	106.2(13)
S(1)-N(3)-Li(1)	95.6(2)	N(4A)-Li(1A)-S(1A)	36.1(3)	C(29A)-O(4A)-Li(1A)	134.6(16)
N(3)-C(9)-C(10)	116.5(4)	N(4A)-S(1A)-N(3A)	96.8(7)	C(32A)-O(4A)-Li(1A)	119.2(14)
N(3)-C(9)-C(11)	107.0(3)	N(4A)-S(1A)-N(1A)	118.5(12)	O(4A)-C(29A)-C(30A)	105.7(14)
C(10)-C(9)-C(11)	108.7(4)	N(3A)-S(1A)-N(1A)	116.9(12)	C(29A)-C(30A)-C(31A)	105.1(11)
N(3)-C(9)-C(12)	109.4(3)	N(4A)-S(1A)-N(2A)	116.2(12)	C(32A)-C(31A)-C(30A)	105.4(9)
C(10)-C(9)-C(12)	108.0(4)	N(3A)-S(1A)-N(2A)	117.6(12)	O(4A)-C(32A)-C(31A)	106.7(14)
C(11)-C(9)-C(12)	106.9(4)	N(1A)-S(1A)-N(2A)	92.7(7)		
C(13)-N(4)-S(1)	128.8(3)	N(4A)-S(1A)-Li(1A)	48.5(4)		

XRD-Analysis of 2b

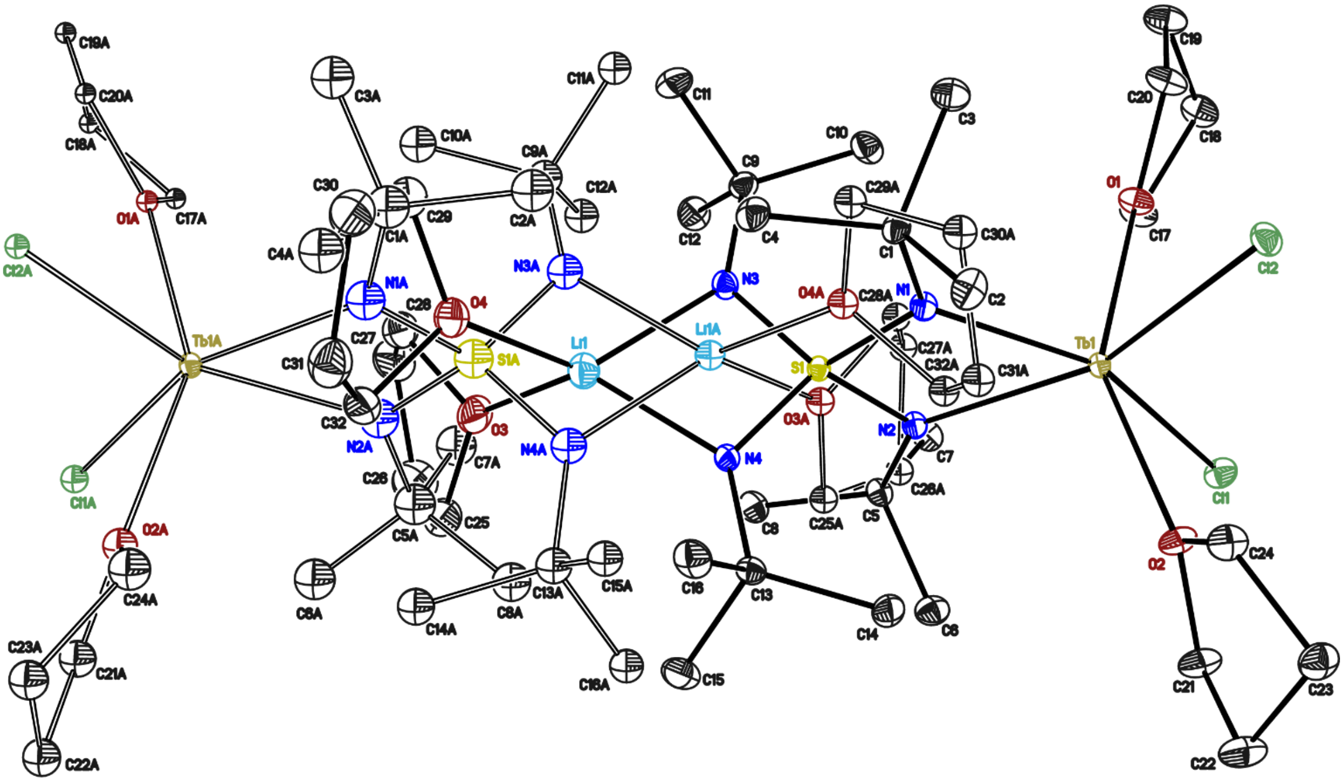

Figure S7 Asymmetric unit of **2b**. Anisotropic displacement parameters are depicted on a probability level of 50%. Hydrogen atoms are omitted for clarity. The whole molecule is disordered and was refined on two positions. The occupancy of the main positions was refined to 0.9877(4). For the refinement, distance restraints and restraints for the anisotropic displacement parameters were used. The structure was refined as an inversion twin.

Table S16 Bond length of **2b** in Å.

**bond**	**length [Å]**	**bond**	**length [Å]**
Tb(1)-N(2)	2.330(2)	Tb(1A)-N(1A)	2.339(10)
Tb(1)-N(1)	2.339(2)	Tb(1A)-N(2A)	2.340(10)
Tb(1)-O(2)	2.428(2)	Tb(1A)-O(2A)	2.428(9)
Tb(1)-O(1)	2.429(2)	Tb(1A)-O(1A)	2.429(9)
Tb(1)-Cl(2)	2.6520(10)	Tb(1A)-Cl(2A)	2.649(10)
Tb(1)-Cl(1)	2.6586(9)	Tb(1A)-Cl(1A)	2.652(10)
Tb(1)-S(1)	3.1378(7)	Tb(1A)-S(1A)	3.147(9)
Li(1)-O(3)	1.969(5)	Li(1A)-O(3A)	1.978(10)
Li(1)-O(4)	1.978(5)	Li(1A)-O(4A)	1.979(11)
Li(1)-N(3)	1.979(5)	Li(1A)-N(3A)	1.981(11)
Li(1)-N(4)	1.986(5)	Li(1A)-N(4A)	1.983(11)
Li(1)-S(1)	2.640(4)	Li(1A)-S(1A)	2.640(11)
S(1)-N(3)	1.565(3)	S(1A)-N(4A)	1.562(10)
S(1)-N(4)	1.565(3)	S(1A)-N(3A)	1.563(10)
S(1)-N(1)	1.626(2)	S(1A)-N(1A)	1.626(10)
S(1)-N(2)	1.630(2)	S(1A)-N(2A)	1.632(10)
N(1)-C(1)	1.499(4)	N(1A)-C(1A)	1.491(10)
C(1)-C(3)	1.535(4)	C(1A)-C(2A)	1.533(10)
C(1)-C(2)	1.536(4)	C(1A)-C(4A)	1.534(10)
C(1)-C(4)	1.538(4)	C(1A)-C(3A)	1.534(10)
N(2)-C(5)	1.499(4)	N(2A)-C(5A)	1.492(10)
C(5)-C(7)	1.532(4)	C(5A)-C(8A)	1.533(10)
C(5)-C(6)	1.534(4)	C(5A)-C(7A)	1.533(10)
C(5)-C(8)	1.534(4)	C(5A)-C(6A)	1.534(10)
N(3)-C(9)	1.479(4)	N(3A)-C(9A)	1.489(10)
C(9)-C(10)	1.532(4)	C(9A)-C(10A)	1.533(10)
C(9)-C(11)	1.533(4)	C(9A)-C(11A)	1.535(10)
C(9)-C(12)	1.537(4)	C(9A)-C(12A)	1.535(10)
N(4)-C(13)	1.479(4)	N(4A)-C(13A)	1.488(10)
C(13)-C(14)	1.529(4)	C(13A)-C(14A)	1.532(10)
C(13)-C(16)	1.534(4)	C(13A)-C(15A)	1.535(10)
C(13)-C(15)	1.538(4)	C(13A)-C(16A)	1.535(10)
O(1)-C(17)	1.458(3)	O(1A)-C(20A)	1.446(10)
O(1)-C(20)	1.464(3)	O(1A)-C(17A)	1.447(10)
C(17)-C(18)	1.513(4)	C(17A)-C(18A)	1.513(11)
C(18)-C(19)	1.512(5)	C(18A)-C(19A)	1.524(11)
C(19)-C(20)	1.516(4)	C(19A)-C(20A)	1.507(10)
O(2)-C(24)	1.454(3)	O(2A)-C(21A)	1.446(10)
O(2)-C(21)	1.475(3)	O(2A)-C(24A)	1.449(10)
C(21)-C(22)	1.514(4)	C(21A)-C(22A)	1.508(11)
C(22)-C(23)	1.526(5)	C(22A)-C(23A)	1.525(12)
C(23)-C(24)	1.505(4)	C(23A)-C(24A)	1.513(11)
O(3)-C(25)	1.432(4)	O(3A)-C(25A)	1.447(10)
O(3)-C(28)	1.441(4)	O(3A)-C(28A)	1.448(10)
C(25)-C(26)	1.517(5)	C(25A)-C(26A)	1.509(11)
C(26)-C(27)	1.524(5)	C(26A)-C(27A)	1.526(11)
C(27)-C(28)	1.517(5)	C(27A)-C(28A)	1.515(11)
O(4)-C(32)	1.434(4)	O(4A)-C(29A)	1.448(10)
O(4)-C(29)	1.436(4)	O(4A)-C(32A)	1.450(10)
C(29)-C(30)	1.516(4)	C(29A)-C(30A)	1.509(11)
C(30)-C(31)	1.544(5)	C(30A)-C(31A)	1.526(11)
C(31)-C(32)	1.514(5)	C(31A)-C(32A)	1.515(11)

Table S17 Bond angles of **2b**.

N(2)-Tb(1)-N(1)	60.67(7)	C(13)-N(4)-Li(1)	135.7(2)	N(3A)-S(1A)-Li(1A)	48.3(4)
N(2)-Tb(1)-O(2)	104.02(8)	S(1)-N(4)-Li(1)	95.31(18)	N(1A)-S(1A)-Li(1A)	134.6(9)
N(1)-Tb(1)-O(2)	102.51(8)	N(4)-C(13)-C(14)	116.4(2)	N(2A)-S(1A)-Li(1A)	132.9(9)
N(2)-Tb(1)-O(1)	101.99(8)	N(4)-C(13)-C(16)	109.7(2)	N(4A)-S(1A)-Tb(1A)	131.6(9)
N(1)-Tb(1)-O(1)	103.48(8)	C(14)-C(13)-C(16)	108.6(3)	N(3A)-S(1A)-Tb(1A)	131.6(9)
O(2)-Tb(1)-O(1)	149.79(6)	N(4)-C(13)-C(15)	106.6(2)	N(1A)-S(1A)-Tb(1A)	46.2(4)
N(2)-Tb(1)-Cl(2)	156.78(6)	C(14)-C(13)-C(15)	108.3(3)	N(2A)-S(1A)-Tb(1A)	46.3(4)
N(1)-Tb(1)-Cl(2)	96.30(6)	C(16)-C(13)-C(15)	106.7(2)	Li(1A)-S(1A)-Tb(1A)	179.2(9)
O(2)-Tb(1)-Cl(2)	82.72(6)	C(17)-O(1)-C(20)	108.9(2)	C(1A)-N(1A)-S(1A)	123.2(14)
O(1)-Tb(1)-Cl(2)	79.37(6)	C(17)-O(1)-Tb(1)	124.65(16)	C(1A)-N(1A)-Tb(1A)	132.6(14)
N(2)-Tb(1)-Cl(1)	96.89(6)	C(20)-O(1)-Tb(1)	126.33(16)	S(1A)-N(1A)-Tb(1A)	103.6(6)
N(1)-Tb(1)-Cl(1)	157.45(6)	O(1)-C(17)-C(18)	105.3(2)	N(1A)-C(1A)-C(2A)	111.1(14)
O(2)-Tb(1)-Cl(1)	79.61(5)	C(19)-C(18)-C(17)	102.3(3)	N(1A)-C(1A)-C(4A)	110.4(14)
O(1)-Tb(1)-Cl(1)	82.28(5)	C(18)-C(19)-C(20)	102.4(3)	C(2A)-C(1A)-C(4A)	108.3(13)
Cl(2)-Tb(1)-Cl(1)	106.22(2)	O(1)-C(20)-C(19)	105.5(2)	N(1A)-C(1A)-C(3A)	110.7(14)
N(2)-Tb(1)-S(1)	30.36(6)	C(24)-O(2)-C(21)	108.4(2)	C(2A)-C(1A)-C(3A)	108.2(13)
N(1)-Tb(1)-S(1)	30.31(6)	C(24)-O(2)-Tb(1)	125.21(16)	C(4A)-C(1A)-C(3A)	108.0(13)
O(2)-Tb(1)-S(1)	105.48(6)	C(21)-O(2)-Tb(1)	126.13(16)	C(5A)-N(2A)-S(1A)	122.4(15)
O(1)-Tb(1)-S(1)	104.74(6)	O(2)-C(21)-C(22)	105.0(2)	C(5A)-N(2A)-Tb(1A)	132.5(15)
Cl(2)-Tb(1)-S(1)	126.55(3)	C(21)-C(22)-C(23)	102.0(3)	S(1A)-N(2A)-Tb(1A)	103.4(6)
Cl(1)-Tb(1)-S(1)	127.22(3)	C(24)-C(23)-C(22)	101.1(3)	N(2A)-C(5A)-C(8A)	110.7(14)
O(3)-Li(1)-O(4)	93.4(2)	O(2)-C(24)-C(23)	105.7(2)	N(2A)-C(5A)-C(7A)	111.0(14)
O(3)-Li(1)-N(3)	116.7(3)	C(25)-O(3)-C(28)	105.2(2)	C(8A)-C(5A)-C(7A)	108.3(13)
O(4)-Li(1)-N(3)	128.9(3)	C(25)-O(3)-Li(1)	135.2(2)	N(2A)-C(5A)-C(6A)	110.6(14)
O(3)-Li(1)-N(4)	130.7(3)	C(28)-O(3)-Li(1)	119.1(2)	C(8A)-C(5A)-C(6A)	108.1(13)
O(4)-Li(1)-N(4)	118.9(3)	O(3)-C(25)-C(26)	105.2(3)	C(7A)-C(5A)-C(6A)	108.1(13)
N(3)-Li(1)-N(4)	72.32(17)	C(25)-C(26)-C(27)	105.3(3)	C(9A)-N(3A)-S(1A)	130.1(16)
O(3)-Li(1)-S(1)	133.0(2)	C(28)-C(27)-C(26)	103.5(3)	C(9A)-N(3A)-Li(1A)	133.7(16)
O(4)-Li(1)-S(1)	133.5(2)	O(3)-C(28)-C(27)	104.9(3)	S(1A)-N(3A)-Li(1A)	95.5(6)
N(3)-Li(1)-S(1)	36.14(10)	C(32)-O(4)-C(29)	104.8(2)	N(3A)-C(9A)-C(10A)	111.2(14)
N(4)-Li(1)-S(1)	36.18(10)	C(32)-O(4)-Li(1)	121.0(2)	N(3A)-C(9A)-C(11A)	110.7(14)
N(3)-S(1)-N(4)	96.74(11)	C(29)-O(4)-Li(1)	134.1(2)	C(10A)-C(9A)-C(11A)	108.1(13)
N(3)-S(1)-N(1)	116.96(13)	O(4)-C(29)-C(30)	105.9(3)	N(3A)-C(9A)-C(12A)	110.8(14)
N(4)-S(1)-N(1)	117.47(15)	C(29)-C(30)-C(31)	104.4(3)	C(10A)-C(9A)-C(12A)	108.1(13)
N(3)-S(1)-N(2)	117.63(15)	C(32)-C(31)-C(30)	103.2(3)	C(11A)-C(9A)-C(12A)	107.9(13)
N(4)-S(1)-N(2)	117.04(13)	O(4)-C(32)-C(31)	105.0(3)	C(13A)-N(4A)-S(1A)	131.1(16)
N(1)-S(1)-N(2)	92.77(10)	N(1A)-Tb(1A)-N(2A)	60.5(4)	C(13A)-N(4A)-Li(1A)	133.3(15)
N(3)-S(1)-Li(1)	48.23(14)	N(1A)-Tb(1A)-O(2A)	102.2(8)	S(1A)-N(4A)-Li(1A)	95.5(6)
N(4)-S(1)-Li(1)	48.51(14)	N(2A)-Tb(1A)-O(2A)	103.6(8)	N(4A)-C(13A)-C(14A)	111.7(14)
N(1)-S(1)-Li(1)	133.53(14)	N(1A)-Tb(1A)-O(1A)	103.6(8)	N(4A)-C(13A)-C(15A)	110.7(14)
N(2)-S(1)-Li(1)	133.70(14)	N(2A)-Tb(1A)-O(1A)	101.9(8)	C(14A)-C(13A)-C(15A)	108.0(13)
N(3)-S(1)-Tb(1)	131.56(10)	O(2A)-Tb(1A)-O(1A)	150.2(8)	N(4A)-C(13A)-C(16A)	110.7(14)
N(4)-S(1)-Tb(1)	131.71(10)	N(1A)-Tb(1A)-Cl(2A)	96.8(6)	C(14A)-C(13A)-C(16A)	108.0(13)
N(1)-S(1)-Tb(1)	46.53(8)	N(2A)-Tb(1A)-Cl(2A)	157.1(7)	C(15A)-C(13A)-C(16A)	107.6(13)
N(2)-S(1)-Tb(1)	46.24(8)	O(2A)-Tb(1A)-Cl(2A)	82.9(7)	C(20A)-O(1A)-C(17A)	108.4(12)
Li(1)-S(1)-Tb(1)	179.78(14)	O(1A)-Tb(1A)-Cl(2A)	79.6(6)	C(20A)-O(1A)-Tb(1A)	125.4(14)
C(1)-N(1)-S(1)	122.74(19)	N(1A)-Tb(1A)-Cl(1A)	156.8(6)	C(17A)-O(1A)-Tb(1A)	126.2(14)
C(1)-N(1)-Tb(1)	134.08(17)	N(2A)-Tb(1A)-Cl(1A)	96.5(6)	O(1A)-C(17A)-C(18A)	105.3(15)
S(1)-N(1)-Tb(1)	103.17(11)	O(2A)-Tb(1A)-Cl(1A)	79.4(6)	C(17A)-C(18A)-C(19A)	102.6(14)
N(1)-C(1)-C(3)	108.9(2)	O(1A)-Tb(1A)-Cl(1A)	82.6(6)	C(20A)-C(19A)-C(18A)	104.1(14)
N(1)-C(1)-C(2)	107.6(2)	Cl(2A)-Tb(1A)-Cl(1A)	106.4(7)	O(1A)-C(20A)-C(19A)	107.9(10)
C(3)-C(1)-C(2)	108.1(3)	N(1A)-Tb(1A)-S(1A)	30.2(3)	C(21A)-O(2A)-C(24A)	108.1(13)
N(1)-C(1)-C(4)	115.9(2)	N(2A)-Tb(1A)-S(1A)	30.3(3)	C(21A)-O(2A)-Tb(1A)	125.9(14)
C(3)-C(1)-C(4)	107.8(2)	O(2A)-Tb(1A)-S(1A)	104.9(6)	C(24A)-O(2A)-Tb(1A)	125.4(14)
C(2)-C(1)-C(4)	108.3(3)	O(1A)-Tb(1A)-S(1A)	104.9(6)	O(2A)-C(21A)-C(22A)	107.8(10)
C(5)-N(2)-S(1)	122.55(19)	Cl(2A)-Tb(1A)-S(1A)	126.9(6)	C(21A)-C(22A)-C(23A)	104.0(14)
C(5)-N(2)-Tb(1)	134.06(17)	Cl(1A)-Tb(1A)-S(1A)	126.7(6)	C(24A)-C(23A)-C(22A)	102.3(14)
S(1)-N(2)-Tb(1)	103.39(11)	O(3A)-Li(1A)-O(4A)	93.2(10)	O(2A)-C(24A)-C(23A)	105.2(15)
N(2)-C(5)-C(7)	108.1(2)	O(3A)-Li(1A)-N(3A)	118.3(12)	C(25A)-O(3A)-C(28A)	108.3(11)
N(2)-C(5)-C(6)	108.9(2)	O(4A)-Li(1A)-N(3A)	129.9(14)	C(25A)-O(3A)-Li(1A)	131.7(17)
C(7)-C(5)-C(6)	107.4(3)	O(3A)-Li(1A)-N(4A)	129.5(13)	C(28A)-O(3A)-Li(1A)	118.9(15)
N(2)-C(5)-C(8)	116.2(2)	O(4A)-Li(1A)-N(4A)	117.9(12)	O(3A)-C(25A)-C(26A)	107.7(11)
C(7)-C(5)-C(8)	108.6(2)	N(3A)-Li(1A)-N(4A)	72.2(6)	C(25A)-C(26A)-C(27A)	103.3(14)
C(6)-C(5)-C(8)	107.4(2)	O(3A)-Li(1A)-S(1A)	133.2(9)	C(28A)-C(27A)-C(26A)	101.8(14)
C(9)-N(3)-S(1)	129.5(2)	O(4A)-Li(1A)-S(1A)	133.6(9)	O(3A)-C(28A)-C(27A)	105.4(15)
C(9)-N(3)-Li(1)	134.9(2)	N(3A)-Li(1A)-S(1A)	36.1(3)	C(29A)-O(4A)-C(32A)	107.9(11)
S(1)-N(3)-Li(1)	95.63(18)	N(4A)-Li(1A)-S(1A)	36.1(3)	C(29A)-O(4A)-Li(1A)	130.9(18)
N(3)-C(9)-C(10)	116.2(3)	N(4A)-S(1A)-N(3A)	96.7(7)	C(32A)-O(4A)-Li(1A)	118.7(16)
N(3)-C(9)-C(11)	106.7(2)	N(4A)-S(1A)-N(1A)	117.9(13)	O(4A)-C(29A)-C(30A)	107.8(11)
C(10)-C(9)-C(11)	108.8(3)	N(3A)-S(1A)-N(1A)	117.3(13)	C(29A)-C(30A)-C(31A)	103.4(14)
N(3)-C(9)-C(12)	109.4(2)	N(4A)-S(1A)-N(2A)	116.8(13)	C(32A)-C(31A)-C(30A)	101.8(14)
C(10)-C(9)-C(12)	108.2(3)	N(3A)-S(1A)-N(2A)	117.3(13)	O(4A)-C(32A)-C(31A)	105.1(14)
C(11)-C(9)-C(12)	107.2(3)	N(1A)-S(1A)-N(2A)	92.6(7)		
C(13)-N(4)-S(1)	128.9(2)	N(4A)-S(1A)-Li(1A)	48.4(4)		

XRD-Analysis of 2c

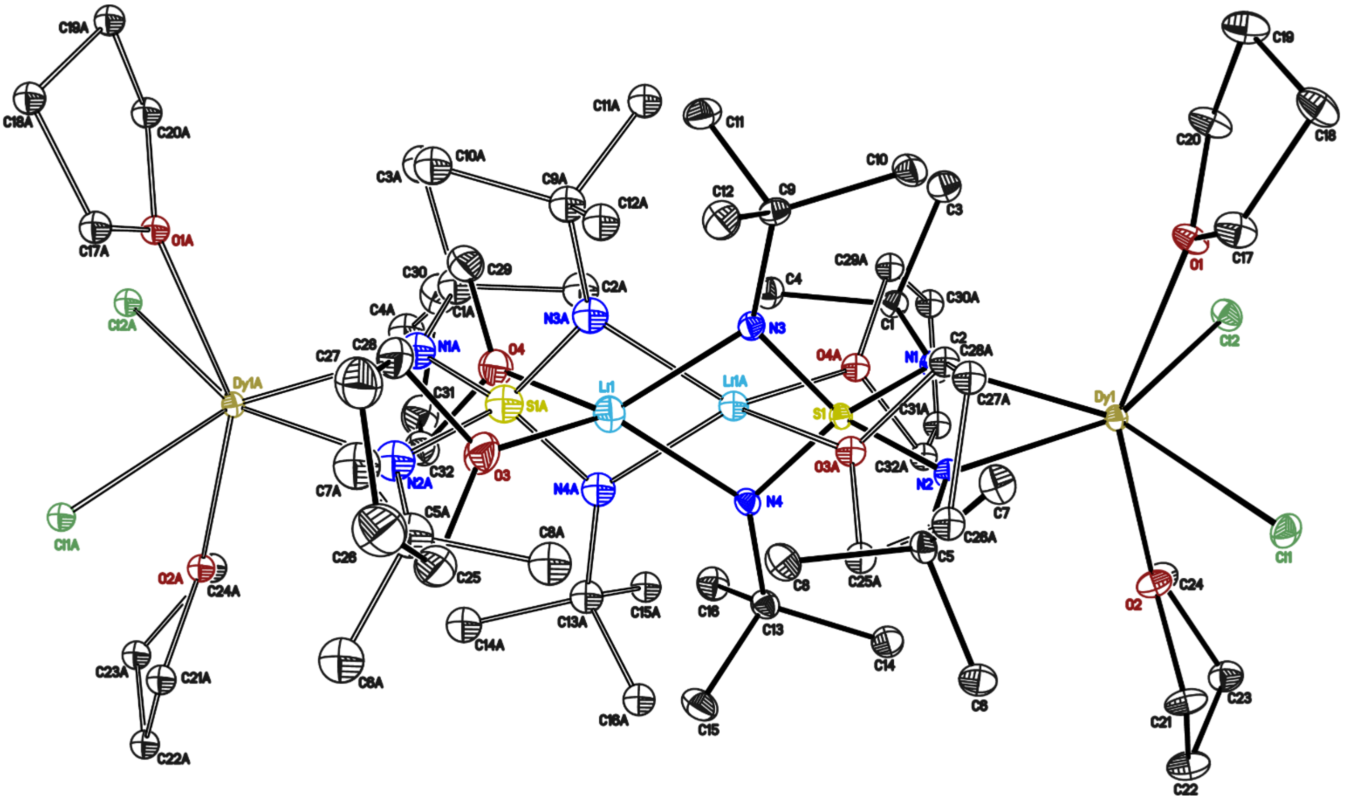

Figure S8 Asymmetric unit of **2c**. Anisotropic displacement parameters are depicted on a probability level of 50%. Hydrogen atoms are omitted for clarity. The whole molecule is disordered and was refined on two positions. The occupancy of the main positions was refined to 0.9730(4). For the refinement, distance restraints and restraints for the anisotropic displacement parameters were used. The structure was refined as an inversion twin.

Table S18 Bond length of **2c** in Å.

**bond**	**length [Å]**	**bond**	**length [Å]**
Dy(1)-N(2)	2.330(2)	Dy(1A)-N(1A)	2.331(10)
Dy(1)-N(1)	2.333(3)	Dy(1A)-N(2A)	2.337(10)
Dy(1)-O(2)	2.429(2)	Dy(1A)-O(2A)	2.424(9)
Dy(1)-O(1)	2.430(2)	Dy(1A)-O(1A)	2.435(9)
Dy(1)-Cl(2)	2.6427(13)	Dy(1A)-Cl(2A)	2.641(10)
Dy(1)-Cl(1)	2.6525(12)	Dy(1A)-Cl(1A)	2.655(10)
Dy(1)-S(1)	3.1360(16)	Dy(1A)-S(1A)	3.142(9)
Li(1)-N(3)	1.982(5)	Li(1A)-N(3A)	1.984(11)
Li(1)-O(3)	1.985(5)	Li(1A)-N(4A)	1.985(11)
Li(1)-N(4)	1.986(5)	Li(1A)-O(4A)	1.986(10)
Li(1)-O(4)	1.987(5)	Li(1A)-O(3A)	1.994(10)
Li(1)-S(1)	2.641(5)	Li(1A)-S(1A)	2.642(11)
S(1)-N(4)	1.569(3)	S(1A)-N(3A)	1.566(10)
S(1)-N(3)	1.571(3)	S(1A)-N(4A)	1.567(10)
S(1)-N(1)	1.633(3)	S(1A)-N(1A)	1.634(10)
S(1)-N(2)	1.640(3)	S(1A)-N(2A)	1.640(10)
N(1)-C(1)	1.509(4)	N(1A)-C(1A)	1.495(10)
C(1)-C(4)	1.541(4)	C(1A)-C(2A)	1.538(10)
C(1)-C(2)	1.544(4)	C(1A)-C(4A)	1.540(10)
C(1)-C(3)	1.545(4)	C(1A)-C(3A)	1.540(10)
N(2)-C(5)	1.500(4)	N(2A)-C(5A)	1.493(10)
C(5)-C(7)	1.540(4)	C(5A)-C(8A)	1.539(10)
C(5)-C(6)	1.540(4)	C(5A)-C(6A)	1.541(10)
C(5)-C(8)	1.540(4)	C(5A)-C(7A)	1.542(10)
N(3)-C(9)	1.479(4)	N(3A)-C(9A)	1.490(10)
C(9)-C(10)	1.536(4)	C(9A)-C(10A)	1.537(10)
C(9)-C(11)	1.539(4)	C(9A)-C(11A)	1.540(10)
C(9)-C(12)	1.548(4)	C(9A)-C(12A)	1.541(10)
N(4)-C(13)	1.483(4)	N(4A)-C(13A)	1.489(10)
C(13)-C(15)	1.536(4)	C(13A)-C(14A)	1.539(10)
C(13)-C(14)	1.537(4)	C(13A)-C(15A)	1.541(10)
C(13)-C(16)	1.540(4)	C(13A)-C(16A)	1.542(10)
O(1)-C(17)	1.465(4)	O(1A)-C(20A)	1.447(10)
O(1)-C(20)	1.466(4)	O(1A)-C(17A)	1.450(10)
C(17)-C(18)	1.515(4)	C(17A)-C(18A)	1.515(11)
C(18)-C(19)	1.520(5)	C(18A)-C(19A)	1.530(12)
C(19)-C(20)	1.521(4)	C(19A)-C(20A)	1.517(11)
O(2)-C(24)	1.463(3)	O(2A)-C(24A)	1.450(10)
O(2)-C(21)	1.477(3)	O(2A)-C(21A)	1.452(10)
C(21)-C(22)	1.521(5)	C(21A)-C(22A)	1.515(11)
C(22)-C(23)	1.533(5)	C(22A)-C(23A)	1.531(12)
C(23)-C(24)	1.514(4)	C(23A)-C(24A)	1.519(11)
O(3)-C(25)	1.437(4)	O(3A)-C(25A)	1.449(10)
O(3)-C(28)	1.446(4)	O(3A)-C(28A)	1.453(10)
C(25)-C(26)	1.523(5)	C(25A)-C(26A)	1.511(11)
C(26)-C(27)	1.531(5)	C(26A)-C(27A)	1.527(12)
C(27)-C(28)	1.520(5)	C(27A)-C(28A)	1.518(11)
O(4)-C(32)	1.436(4)	O(4A)-C(29A)	1.450(10)
O(4)-C(29)	1.441(4)	O(4A)-C(32A)	1.454(10)
C(29)-C(30)	1.523(5)	C(29A)-C(30A)	1.512(11)
C(30)-C(31)	1.543(5)	C(30A)-C(31A)	1.524(11)
C(31)-C(32)	1.523(5)	C(31A)-C(32A)	1.516(11)

Table S19 Bond angles of **2c**.

N(2)-Dy(1)-N(1)	61.10(8)	C(13)-N(4)-Li(1)	135.5(3)	N(4A)-S(1A)-Li(1A)	48.4(4)
N(2)-Dy(1)-O(2)	103.95(9)	S(1)-N(4)-Li(1)	95.25(19)	N(1A)-S(1A)-Li(1A)	135.2(8)
N(1)-Dy(1)-O(2)	102.40(9)	N(4)-C(13)-C(15)	106.8(3)	N(2A)-S(1A)-Li(1A)	132.2(8)
N(2)-Dy(1)-O(1)	101.91(9)	N(4)-C(13)-C(14)	116.3(3)	N(3A)-S(1A)-Dy(1A)	131.6(8)
N(1)-Dy(1)-O(1)	103.52(9)	C(15)-C(13)-C(14)	108.2(3)	N(4A)-S(1A)-Dy(1A)	131.6(8)
O(2)-Dy(1)-O(1)	149.85(7)	N(4)-C(13)-C(16)	109.6(3)	N(1A)-S(1A)-Dy(1A)	46.2(4)
N(2)-Dy(1)-Cl(2)	156.75(6)	C(15)-C(13)-C(16)	107.2(3)	N(2A)-S(1A)-Dy(1A)	46.4(4)
N(1)-Dy(1)-Cl(2)	95.83(7)	C(14)-C(13)-C(16)	108.4(3)	Li(1A)-S(1A)-Dy(1A)	178.6(8)
O(2)-Dy(1)-Cl(2)	82.77(7)	C(17)-O(1)-C(20)	108.8(2)	C(1A)-N(1A)-S(1A)	123.5(13)
O(1)-Dy(1)-Cl(2)	79.53(7)	C(17)-O(1)-Dy(1)	124.80(17)	C(1A)-N(1A)-Dy(1A)	133.0(12)
N(2)-Dy(1)-Cl(1)	96.28(7)	C(20)-O(1)-Dy(1)	126.25(17)	S(1A)-N(1A)-Dy(1A)	103.4(6)
N(1)-Dy(1)-Cl(1)	157.27(6)	O(1)-C(17)-C(18)	105.3(3)	N(1A)-C(1A)-C(2A)	111.6(14)
O(2)-Dy(1)-Cl(1)	79.75(6)	C(17)-C(18)-C(19)	102.2(3)	N(1A)-C(1A)-C(4A)	109.9(14)
O(1)-Dy(1)-Cl(1)	82.27(6)	C(18)-C(19)-C(20)	102.1(3)	C(2A)-C(1A)-C(4A)	108.3(13)
Cl(2)-Dy(1)-Cl(1)	106.86(4)	O(1)-C(20)-C(19)	105.6(3)	N(1A)-C(1A)-C(3A)	110.7(14)
N(2)-Dy(1)-S(1)	30.63(6)	C(24)-O(2)-C(21)	108.5(2)	C(2A)-C(1A)-C(3A)	108.3(13)
N(1)-Dy(1)-S(1)	30.47(6)	C(24)-O(2)-Dy(1)	125.11(17)	C(4A)-C(1A)-C(3A)	107.9(13)
O(2)-Dy(1)-S(1)	105.37(6)	C(21)-O(2)-Dy(1)	126.13(18)	C(5A)-N(2A)-S(1A)	123.4(13)
O(1)-Dy(1)-S(1)	104.78(6)	O(2)-C(21)-C(22)	105.2(3)	C(5A)-N(2A)-Dy(1A)	133.7(13)
Cl(2)-Dy(1)-S(1)	126.25(4)	C(21)-C(22)-C(23)	102.0(3)	S(1A)-N(2A)-Dy(1A)	103.0(6)
Cl(1)-Dy(1)-S(1)	126.88(4)	C(24)-C(23)-C(22)	101.1(3)	N(2A)-C(5A)-C(8A)	111.3(14)
N(3)-Li(1)-O(3)	116.6(3)	O(2)-C(24)-C(23)	105.5(2)	N(2A)-C(5A)-C(6A)	110.8(14)
N(3)-Li(1)-N(4)	72.59(18)	C(25)-O(3)-C(28)	105.1(3)	C(8A)-C(5A)-C(6A)	108.0(13)
O(3)-Li(1)-N(4)	130.7(3)	C(25)-O(3)-Li(1)	135.3(3)	N(2A)-C(5A)-C(7A)	110.9(14)
N(3)-Li(1)-O(4)	129.1(3)	C(28)-O(3)-Li(1)	119.3(2)	C(8A)-C(5A)-C(7A)	107.9(13)
O(3)-Li(1)-O(4)	92.9(2)	O(3)-C(25)-C(26)	105.2(3)	C(6A)-C(5A)-C(7A)	107.8(13)
N(4)-Li(1)-O(4)	119.3(3)	C(25)-C(26)-C(27)	105.3(3)	C(9A)-N(3A)-S(1A)	131.0(14)
N(3)-Li(1)-S(1)	36.31(11)	C(28)-C(27)-C(26)	103.4(3)	C(9A)-N(3A)-Li(1A)	133.1(13)
O(3)-Li(1)-S(1)	133.2(2)	O(3)-C(28)-C(27)	105.1(3)	S(1A)-N(3A)-Li(1A)	95.4(6)
N(4)-Li(1)-S(1)	36.27(11)	C(32)-O(4)-C(29)	104.9(2)	N(3A)-C(9A)-C(10A)	111.4(14)
O(4)-Li(1)-S(1)	133.9(2)	C(32)-O(4)-Li(1)	120.8(2)	N(3A)-C(9A)-C(11A)	110.3(14)
N(4)-S(1)-N(3)	96.81(12)	C(29)-O(4)-Li(1)	134.2(2)	C(10A)-C(9A)-C(11A)	108.3(13)
N(4)-S(1)-N(1)	117.64(16)	O(4)-C(29)-C(30)	105.9(3)	N(3A)-C(9A)-C(12A)	110.7(14)
N(3)-S(1)-N(1)	117.06(14)	C(29)-C(30)-C(31)	104.4(3)	C(10A)-C(9A)-C(12A)	108.2(13)
N(4)-S(1)-N(2)	117.01(14)	C(32)-C(31)-C(30)	103.4(3)	C(11A)-C(9A)-C(12A)	107.8(13)
N(3)-S(1)-N(2)	117.23(16)	O(4)-C(32)-C(31)	104.9(3)	C(13A)-N(4A)-S(1A)	130.9(13)
N(1)-S(1)-N(2)	92.83(11)	N(1A)-Dy(1A)-N(2A)	61.0(4)	C(13A)-N(4A)-Li(1A)	133.5(13)
N(4)-S(1)-Li(1)	48.47(14)	N(1A)-Dy(1A)-O(2A)	102.8(7)	S(1A)-N(4A)-Li(1A)	95.4(6)
N(3)-S(1)-Li(1)	48.34(14)	N(2A)-Dy(1A)-O(2A)	103.5(7)	N(4A)-C(13A)-C(14A)	111.9(14)
N(1)-S(1)-Li(1)	133.64(15)	N(1A)-Dy(1A)-O(1A)	103.6(7)	N(4A)-C(13A)-C(15A)	110.9(14)
N(2)-S(1)-Li(1)	133.53(15)	N(2A)-Dy(1A)-O(1A)	101.4(7)	C(14A)-C(13A)-C(15A)	107.7(13)
N(4)-S(1)-Dy(1)	131.79(11)	O(2A)-Dy(1A)-O(1A)	150.1(8)	N(4A)-C(13A)-C(16A)	110.7(14)
N(3)-S(1)-Dy(1)	131.39(11)	N(1A)-Dy(1A)-Cl(2A)	97.1(6)	C(14A)-C(13A)-C(16A)	107.9(13)
N(1)-S(1)-Dy(1)	46.44(9)	N(2A)-Dy(1A)-Cl(2A)	157.9(6)	C(15A)-C(13A)-C(16A)	107.5(13)
N(2)-S(1)-Dy(1)	46.40(8)	O(2A)-Dy(1A)-Cl(2A)	83.2(6)	C(20A)-O(1A)-C(17A)	109.1(10)
Li(1)-S(1)-Dy(1)	179.72(14)	O(1A)-Dy(1A)-Cl(2A)	79.9(6)	C(20A)-O(1A)-Dy(1A)	126.4(12)
C(1)-N(1)-S(1)	122.2(2)	N(1A)-Dy(1A)-Cl(1A)	156.6(6)	C(17A)-O(1A)-Dy(1A)	124.3(12)
C(1)-N(1)-Dy(1)	134.71(18)	N(2A)-Dy(1A)-Cl(1A)	95.8(5)	O(1A)-C(17A)-C(18A)	107.1(12)
S(1)-N(1)-Dy(1)	103.09(12)	O(2A)-Dy(1A)-Cl(1A)	79.4(6)	C(17A)-C(18A)-C(19A)	103.0(13)
N(1)-C(1)-C(4)	116.4(3)	O(1A)-Dy(1A)-Cl(1A)	81.9(6)	C(20A)-C(19A)-C(18A)	102.6(14)
N(1)-C(1)-C(2)	107.5(3)	Cl(2A)-Dy(1A)-Cl(1A)	106.2(7)	O(1A)-C(20A)-C(19A)	106.6(13)
C(4)-C(1)-C(2)	108.2(3)	N(1A)-Dy(1A)-S(1A)	30.4(3)	C(24A)-O(2A)-C(21A)	108.6(11)
N(1)-C(1)-C(3)	108.6(3)	N(2A)-Dy(1A)-S(1A)	30.6(3)	C(24A)-O(2A)-Dy(1A)	126.4(13)
C(4)-C(1)-C(3)	107.5(3)	O(2A)-Dy(1A)-S(1A)	105.2(6)	C(21A)-O(2A)-Dy(1A)	125.0(13)
C(2)-C(1)-C(3)	108.3(3)	O(1A)-Dy(1A)-S(1A)	104.6(6)	O(2A)-C(21A)-C(22A)	107.4(11)
C(5)-N(2)-S(1)	122.6(2)	Cl(2A)-Dy(1A)-S(1A)	127.4(6)	C(21A)-C(22A)-C(23A)	103.3(14)
C(5)-N(2)-Dy(1)	134.44(18)	Cl(1A)-Dy(1A)-S(1A)	126.3(5)	C(24A)-C(23A)-C(22A)	102.0(14)
S(1)-N(2)-Dy(1)	102.97(12)	N(3A)-Li(1A)-N(4A)	72.4(6)	O(2A)-C(24A)-C(23A)	105.2(14)
N(2)-C(5)-C(7)	108.3(3)	N(3A)-Li(1A)-O(4A)	130.5(13)	C(25A)-O(3A)-C(28A)	107.9(12)
N(2)-C(5)-C(6)	109.0(3)	N(4A)-Li(1A)-O(4A)	117.9(11)	C(25A)-O(3A)-Li(1A)	132.9(16)
C(7)-C(5)-C(6)	107.4(3)	N(3A)-Li(1A)-O(3A)	117.4(11)	C(28A)-O(3A)-Li(1A)	118.7(14)
N(2)-C(5)-C(8)	116.2(3)	N(4A)-Li(1A)-O(3A)	130.1(13)	O(3A)-C(25A)-C(26A)	108.1(10)
C(7)-C(5)-C(8)	108.4(3)	O(4A)-Li(1A)-O(3A)	93.0(10)	C(25A)-C(26A)-C(27A)	104.9(12)
C(6)-C(5)-C(8)	107.3(3)	N(3A)-Li(1A)-S(1A)	36.2(3)	C(28A)-C(27A)-C(26A)	102.9(13)
C(9)-N(3)-S(1)	129.6(2)	N(4A)-Li(1A)-S(1A)	36.2(3)	O(3A)-C(28A)-C(27A)	105.4(14)
C(9)-N(3)-Li(1)	135.0(3)	O(4A)-Li(1A)-S(1A)	134.3(9)	C(29A)-O(4A)-C(32A)	106.2(13)
S(1)-N(3)-Li(1)	95.35(19)	O(3A)-Li(1A)-S(1A)	132.7(9)	C(29A)-O(4A)-Li(1A)	134.7(16)
N(3)-C(9)-C(10)	116.4(3)	N(3A)-S(1A)-N(4A)	96.8(7)	C(32A)-O(4A)-Li(1A)	119.0(14)
N(3)-C(9)-C(11)	107.2(3)	N(3A)-S(1A)-N(1A)	117.1(12)	O(4A)-C(29A)-C(30A)	107.1(13)
C(10)-C(9)-C(11)	108.8(3)	N(4A)-S(1A)-N(1A)	118.2(12)	C(29A)-C(30A)-C(31A)	105.6(9)
N(3)-C(9)-C(12)	109.2(3)	N(3A)-S(1A)-N(2A)	117.3(12)	C(32A)-C(31A)-C(30A)	104.4(12)
C(10)-C(9)-C(12)	108.0(3)	N(4A)-S(1A)-N(2A)	116.5(12)	O(4A)-C(32A)-C(31A)	105.4(14)
C(11)-C(9)-C(12)	106.9(3)	N(1A)-S(1A)-N(2A)	92.6(7)		
C(13)-N(4)-S(1)	129.2(2)	N(3A)-S(1A)-Li(1A)	48.4(4)		

XRD-Analysis of 2d

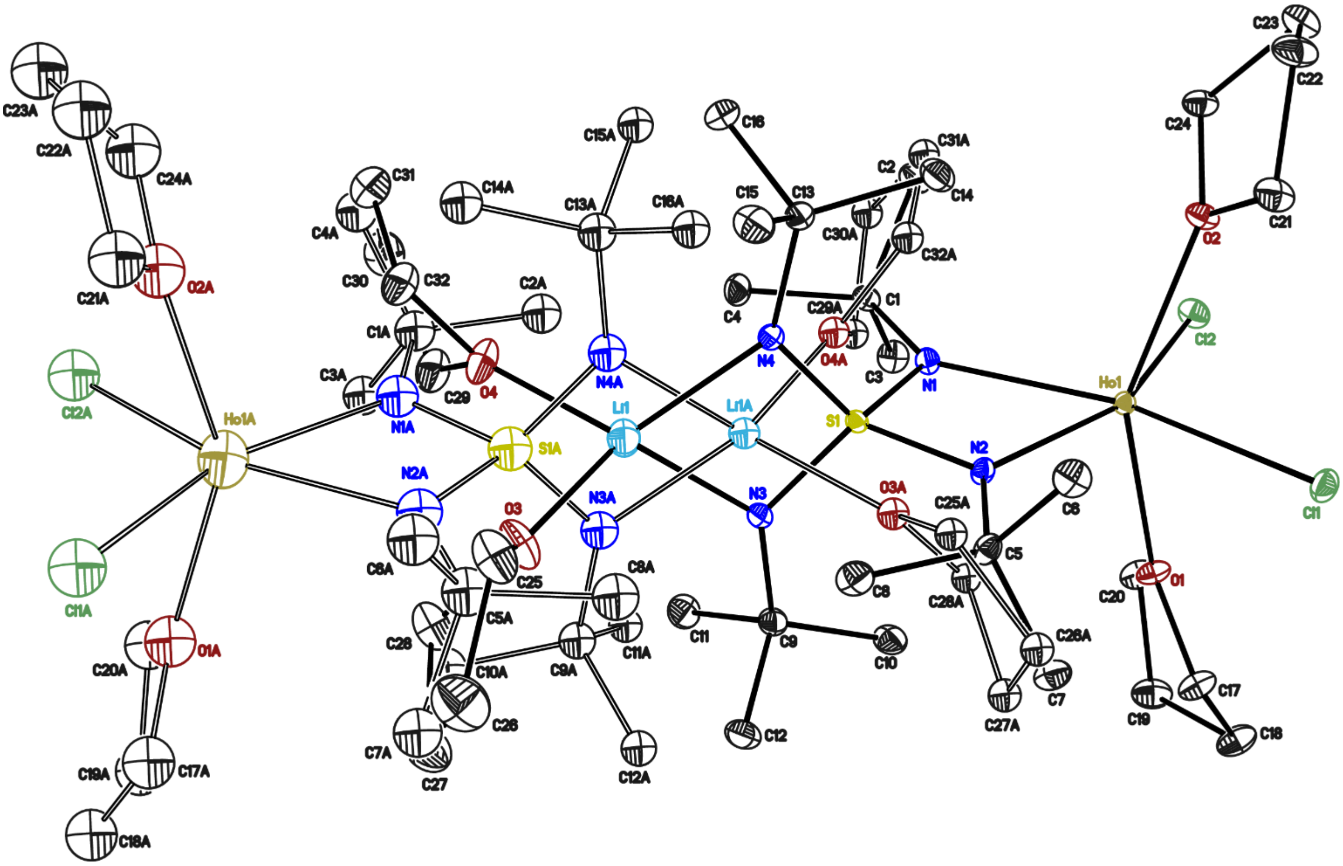

Figure S9 Asymmetric unit of **2d**. Anisotropic displacement parameters are depicted on a probability level of 50%. Hydrogen atoms are omitted for clarity. The whole molecule is disordered and was refined on two positions. The occupancy of the main positions was refined to 0.9267(7). For the refinement, distance restraints and restraints for the anisotropic displacement parameters were used. The structure was refined as an inversion twin.

Table S20 Bond length of **2d** in Å.

**bond**	**length [Å]**	**bond**	**length [Å]**
Ho(1)-N(2)	2.312(2)	Ho(1A)-N(1A)	2.316(9)
Ho(1)-N(1)	2.317(2)	Ho(1A)-N(2A)	2.321(9)
Ho(1)-O(2)	2.404(2)	Ho(1A)-O(2A)	2.398(9)
Ho(1)-O(1)	2.407(2)	Ho(1A)-O(1A)	2.407(9)
Ho(1)-Cl(2)	2.6222(10)	Ho(1A)-Cl(2A)	2.621(9)
Ho(1)-Cl(1)	2.6327(9)	Ho(1A)-Cl(1A)	2.624(8)
Ho(1)-S(1)	3.1148(7)	Ho(1A)-S(1A)	3.112(7)
Li(1)-N(3)	1.972(5)	Li(1A)-N(3A)	1.972(10)
Li(1)-N(4)	1.975(5)	Li(1A)-O(3A)	1.979(10)
Li(1)-O(3)	1.977(5)	Li(1A)-O(4A)	1.982(10)
Li(1)-O(4)	1.979(5)	Li(1A)-N(4A)	1.983(11)
Li(1)-S(1)	2.632(5)	Li(1A)-S(1A)	2.632(10)
S(1)-N(4)	1.564(2)	S(1A)-N(3A)	1.556(9)
S(1)-N(3)	1.565(2)	S(1A)-N(4A)	1.564(9)
S(1)-N(1)	1.630(2)	S(1A)-N(2A)	1.626(9)
S(1)-N(2)	1.631(2)	S(1A)-N(1A)	1.627(9)
N(1)-C(1)	1.503(4)	N(1A)-C(1A)	1.495(10)
C(1)-C(2)	1.529(4)	C(1A)-C(2A)	1.530(10)
C(1)-C(4)	1.530(4)	C(1A)-C(4A)	1.531(10)
C(1)-C(3)	1.539(4)	C(1A)-C(3A)	1.532(10)
N(2)-C(5)	1.504(4)	N(2A)-C(5A)	1.494(10)
C(5)-C(6)	1.532(4)	C(5A)-C(8A)	1.532(10)
C(5)-C(7)	1.532(4)	C(5A)-C(6A)	1.533(10)
C(5)-C(8)	1.537(4)	C(5A)-C(7A)	1.535(10)
N(3)-C(9)	1.479(4)	N(3A)-C(9A)	1.490(9)
C(9)-C(10)	1.529(4)	C(9A)-C(12A)	1.530(10)
C(9)-C(11)	1.532(4)	C(9A)-C(10A)	1.530(10)
C(9)-C(12)	1.538(4)	C(9A)-C(11A)	1.534(10)
N(4)-C(13)	1.481(4)	N(4A)-C(13A)	1.484(9)
C(13)-C(14)	1.524(4)	C(13A)-C(14A)	1.529(10)
C(13)-C(15)	1.534(4)	C(13A)-C(15A)	1.534(10)
C(13)-C(16)	1.536(4)	C(13A)-C(16A)	1.537(10)
O(1)-C(17)	1.458(3)	O(1A)-C(20A)	1.440(10)
O(1)-C(20)	1.465(3)	O(1A)-C(17A)	1.445(10)
C(17)-C(18)	1.515(4)	C(17A)-C(18A)	1.510(10)
C(18)-C(19)	1.520(4)	C(18A)-C(19A)	1.525(11)
C(19)-C(20)	1.517(4)	C(19A)-C(20A)	1.512(10)
O(2)-C(24)	1.461(3)	O(2A)-C(21A)	1.444(10)
O(2)-C(21)	1.468(3)	O(2A)-C(24A)	1.447(10)
C(21)-C(22)	1.513(4)	C(21A)-C(22A)	1.513(10)
C(22)-C(23)	1.527(4)	C(22A)-C(23A)	1.529(11)
C(23)-C(24)	1.509(4)	C(23A)-C(24A)	1.511(11)
O(3)-C(25)	1.432(4)	O(3A)-C(25A)	1.449(10)
O(3)-C(28)	1.434(4)	O(3A)-C(28A)	1.451(10)
C(25)-C(26)	1.523(5)	C(25A)-C(26A)	1.506(10)
C(26)-C(27)	1.532(5)	C(26A)-C(27A)	1.525(11)
C(27)-C(28)	1.520(5)	C(27A)-C(28A)	1.526(10)
O(4)-C(32)	1.430(4)	O(4A)-C(29A)	1.443(10)
O(4)-C(29)	1.439(4)	O(4A)-C(32A)	1.449(10)
C(29)-C(30)	1.513(5)	C(29A)-C(30A)	1.511(10)
C(30)-C(31)	1.541(5)	C(30A)-C(31A)	1.521(11)
C(31)-C(32)	1.520(5)	C(31A)-C(32A)	1.508(10)

Table S21 Bond angles of **2d**.

N(2)-Ho(1)-N(1)	61.36(7)	C(13)-N(4)-Li(1)	135.5(2)	N(4A)-S(1A)-Li(1A)	48.6(4)
N(2)-Ho(1)-O(2)	103.93(8)	S(1)-N(4)-Li(1)	95.44(19)	N(2A)-S(1A)-Li(1A)	134.8(6)
N(1)-Ho(1)-O(2)	102.48(8)	N(4)-C(13)-C(14)	116.5(2)	N(1A)-S(1A)-Li(1A)	132.0(6)
N(2)-Ho(1)-O(1)	101.83(8)	N(4)-C(13)-C(15)	106.4(2)	N(3A)-S(1A)-Ho(1A)	132.8(5)
N(1)-Ho(1)-O(1)	103.33(8)	C(14)-C(13)-C(15)	108.3(3)	N(4A)-S(1A)-Ho(1A)	130.4(5)
O(2)-Ho(1)-O(1)	149.93(6)	N(4)-C(13)-C(16)	109.5(2)	N(2A)-S(1A)-Ho(1A)	46.7(3)
N(2)-Ho(1)-Cl(2)	156.91(6)	C(14)-C(13)-C(16)	108.7(3)	N(1A)-S(1A)-Ho(1A)	46.5(3)
N(1)-Ho(1)-Cl(2)	95.73(6)	C(15)-C(13)-C(16)	107.0(2)	Li(1A)-S(1A)-Ho(1A)	178.2(5)
O(2)-Ho(1)-Cl(2)	82.75(6)	C(17)-O(1)-C(20)	109.0(2)	C(1A)-N(1A)-S(1A)	123.2(9)
O(1)-Ho(1)-Cl(2)	79.58(6)	C(17)-O(1)-Ho(1)	124.76(16)	C(1A)-N(1A)-Ho(1A)	133.9(9)
N(2)-Ho(1)-Cl(1)	96.35(6)	C(20)-O(1)-Ho(1)	126.09(16)	S(1A)-N(1A)-Ho(1A)	102.8(5)
N(1)-Ho(1)-Cl(1)	157.61(6)	O(1)-C(17)-C(18)	105.1(2)	N(1A)-C(1A)-C(2A)	112.2(12)
O(2)-Ho(1)-Cl(1)	79.67(6)	C(17)-C(18)-C(19)	102.0(3)	N(1A)-C(1A)-C(4A)	109.8(12)
O(1)-Ho(1)-Cl(1)	82.33(5)	C(20)-C(19)-C(18)	102.0(3)	C(2A)-C(1A)-C(4A)	108.3(12)
Cl(2)-Ho(1)-Cl(1)	106.63(3)	O(1)-C(20)-C(19)	105.3(2)	N(1A)-C(1A)-C(3A)	111.1(12)
N(2)-Ho(1)-S(1)	30.68(6)	C(24)-O(2)-C(21)	108.4(2)	C(2A)-C(1A)-C(3A)	107.3(12)
N(1)-Ho(1)-S(1)	30.68(6)	C(24)-O(2)-Ho(1)	125.13(16)	C(4A)-C(1A)-C(3A)	108.1(12)
O(2)-Ho(1)-S(1)	105.45(6)	C(21)-O(2)-Ho(1)	126.25(17)	C(5A)-N(2A)-S(1A)	124.0(9)
O(1)-Ho(1)-S(1)	104.62(6)	O(2)-C(21)-C(22)	105.5(2)	C(5A)-N(2A)-Ho(1A)	133.3(9)
Cl(2)-Ho(1)-S(1)	126.36(3)	C(21)-C(22)-C(23)	101.9(3)	S(1A)-N(2A)-Ho(1A)	102.7(5)
Cl(1)-Ho(1)-S(1)	127.01(3)	C(24)-C(23)-C(22)	101.3(3)	N(2A)-C(5A)-C(8A)	113.0(12)
N(3)-Li(1)-N(4)	72.53(18)	O(2)-C(24)-C(23)	105.4(2)	N(2A)-C(5A)-C(6A)	111.4(12)
N(3)-Li(1)-O(3)	116.8(3)	C(25)-O(3)-C(28)	105.4(3)	C(8A)-C(5A)-C(6A)	107.6(12)
N(4)-Li(1)-O(3)	130.9(3)	C(25)-O(3)-Li(1)	135.0(3)	N(2A)-C(5A)-C(7A)	109.6(12)
N(3)-Li(1)-O(4)	129.1(3)	C(28)-O(3)-Li(1)	119.3(2)	C(8A)-C(5A)-C(7A)	107.0(12)
N(4)-Li(1)-O(4)	119.2(3)	O(3)-C(25)-C(26)	105.2(3)	C(6A)-C(5A)-C(7A)	108.1(12)
O(3)-Li(1)-O(4)	92.8(2)	C(25)-C(26)-C(27)	105.2(3)	C(9A)-N(3A)-S(1A)	129.9(9)
N(3)-Li(1)-S(1)	36.28(11)	C(28)-C(27)-C(26)	103.1(3)	C(9A)-N(3A)-Li(1A)	134.3(9)
N(4)-Li(1)-S(1)	36.25(11)	O(3)-C(28)-C(27)	105.2(3)	S(1A)-N(3A)-Li(1A)	95.8(6)
O(3)-Li(1)-S(1)	133.3(3)	C(32)-O(4)-C(29)	104.7(2)	N(3A)-C(9A)-C(12A)	110.5(12)
O(4)-Li(1)-S(1)	133.9(3)	C(32)-O(4)-Li(1)	120.9(2)	N(3A)-C(9A)-C(10A)	112.2(12)
N(4)-S(1)-N(3)	96.52(11)	C(29)-O(4)-Li(1)	134.2(2)	C(12A)-C(9A)-C(10A)	108.8(12)
N(4)-S(1)-N(1)	117.65(15)	O(4)-C(29)-C(30)	105.8(3)	N(3A)-C(9A)-C(11A)	109.7(12)
N(3)-S(1)-N(1)	116.99(13)	C(29)-C(30)-C(31)	104.6(3)	C(12A)-C(9A)-C(11A)	107.8(12)
N(4)-S(1)-N(2)	117.05(13)	C(32)-C(31)-C(30)	103.0(3)	C(10A)-C(9A)-C(11A)	107.7(12)
N(3)-S(1)-N(2)	117.60(15)	O(4)-C(32)-C(31)	104.9(3)	C(13A)-N(4A)-S(1A)	132.5(9)
N(1)-S(1)-N(2)	92.83(10)	N(1A)-Ho(1A)-N(2A)	61.3(4)	C(13A)-N(4A)-Li(1A)	132.2(9)
N(4)-S(1)-Li(1)	48.31(14)	N(1A)-Ho(1A)-O(2A)	102.7(6)	S(1A)-N(4A)-Li(1A)	95.1(6)
N(3)-S(1)-Li(1)	48.21(14)	N(2A)-Ho(1A)-O(2A)	104.1(6)	N(4A)-C(13A)-C(14A)	113.6(12)
N(1)-S(1)-Li(1)	133.47(14)	N(1A)-Ho(1A)-O(1A)	103.5(6)	N(4A)-C(13A)-C(15A)	110.9(12)
N(2)-S(1)-Li(1)	133.69(14)	N(2A)-Ho(1A)-O(1A)	102.0(6)	C(14A)-C(13A)-C(15A)	107.5(12)
N(4)-S(1)-Ho(1)	131.86(10)	O(2A)-Ho(1A)-O(1A)	149.5(7)	N(4A)-C(13A)-C(16A)	110.0(12)
N(3)-S(1)-Ho(1)	131.62(10)	N(1A)-Ho(1A)-Cl(2A)	96.0(4)	C(14A)-C(13A)-C(16A)	107.9(12)
N(1)-S(1)-Ho(1)	46.50(8)	N(2A)-Ho(1A)-Cl(2A)	157.1(5)	C(15A)-C(13A)-C(16A)	106.5(12)
N(2)-S(1)-Ho(1)	46.34(8)	O(2A)-Ho(1A)-Cl(2A)	82.5(5)	C(20A)-O(1A)-C(17A)	108.9(9)
Li(1)-S(1)-Ho(1)	179.83(14)	O(1A)-Ho(1A)-Cl(2A)	79.4(5)	C(20A)-O(1A)-Ho(1A)	126.2(11)
C(1)-N(1)-S(1)	122.26(18)	N(1A)-Ho(1A)-Cl(1A)	157.5(4)	C(17A)-O(1A)-Ho(1A)	124.8(11)
C(1)-N(1)-Ho(1)	134.90(17)	N(2A)-Ho(1A)-Cl(1A)	96.3(4)	O(1A)-C(17A)-C(18A)	107.3(11)
S(1)-N(1)-Ho(1)	102.82(11)	O(2A)-Ho(1A)-Cl(1A)	79.7(5)	C(17A)-C(18A)-C(19A)	102.7(12)
N(1)-C(1)-C(2)	107.8(2)	O(1A)-Ho(1A)-Cl(1A)	82.0(5)	C(20A)-C(19A)-C(18A)	102.8(12)
N(1)-C(1)-C(4)	116.3(2)	Cl(2A)-Ho(1A)-Cl(1A)	106.5(5)	O(1A)-C(20A)-C(19A)	107.0(11)
C(2)-C(1)-C(4)	108.4(3)	N(1A)-Ho(1A)-S(1A)	30.6(2)	C(21A)-O(2A)-C(24A)	109.0(9)
N(1)-C(1)-C(3)	108.5(2)	N(2A)-Ho(1A)-S(1A)	30.6(2)	C(21A)-O(2A)-Ho(1A)	125.4(11)
C(2)-C(1)-C(3)	107.9(2)	O(2A)-Ho(1A)-S(1A)	105.7(5)	C(24A)-O(2A)-Ho(1A)	125.1(11)
C(4)-C(1)-C(3)	107.7(2)	O(1A)-Ho(1A)-S(1A)	104.8(5)	O(2A)-C(21A)-C(22A)	106.6(11)
C(5)-N(2)-S(1)	122.77(18)	Cl(2A)-Ho(1A)-S(1A)	126.6(4)	C(21A)-C(22A)-C(23A)	102.3(12)
C(5)-N(2)-Ho(1)	134.24(17)	Cl(1A)-Ho(1A)-S(1A)	126.9(4)	C(24A)-C(23A)-C(22A)	101.7(12)
S(1)-N(2)-Ho(1)	102.98(11)	N(3A)-Li(1A)-O(3A)	118.7(9)	O(2A)-C(24A)-C(23A)	106.0(12)
N(2)-C(5)-C(6)	108.7(2)	N(3A)-Li(1A)-O(4A)	131.6(10)	C(25A)-O(3A)-C(28A)	106.8(10)
N(2)-C(5)-C(7)	108.2(2)	O(3A)-Li(1A)-O(4A)	93.4(8)	C(25A)-O(3A)-Li(1A)	134.3(11)
C(6)-C(5)-C(7)	108.3(3)	N(3A)-Li(1A)-N(4A)	72.3(5)	C(28A)-O(3A)-Li(1A)	118.8(11)
N(2)-C(5)-C(8)	115.4(2)	O(3A)-Li(1A)-N(4A)	128.6(9)	O(3A)-C(25A)-C(26A)	107.7(9)
C(6)-C(5)-C(8)	107.4(2)	O(4A)-Li(1A)-N(4A)	116.4(8)	C(25A)-C(26A)-C(27A)	104.1(10)
C(7)-C(5)-C(8)	108.6(3)	N(3A)-Li(1A)-S(1A)	36.0(3)	C(26A)-C(27A)-C(28A)	101.9(11)
C(9)-N(3)-S(1)	129.6(2)	O(3A)-Li(1A)-S(1A)	133.9(7)	O(3A)-C(28A)-C(27A)	102.8(11)
C(9)-N(3)-Li(1)	134.9(2)	O(4A)-Li(1A)-S(1A)	132.6(7)	C(29A)-O(4A)-C(32A)	105.4(10)
S(1)-N(3)-Li(1)	95.51(19)	N(4A)-Li(1A)-S(1A)	36.3(3)	C(29A)-O(4A)-Li(1A)	136.1(11)
N(3)-C(9)-C(10)	116.4(3)	N(3A)-S(1A)-N(4A)	96.8(6)	C(32A)-O(4A)-Li(1A)	118.2(10)
N(3)-C(9)-C(11)	107.0(2)	N(3A)-S(1A)-N(2A)	118.6(10)	O(4A)-C(29A)-C(30A)	105.2(11)
C(10)-C(9)-C(11)	108.7(3)	N(4A)-S(1A)-N(2A)	115.8(10)	C(29A)-C(30A)-C(31A)	103.7(10)
N(3)-C(9)-C(12)	109.2(2)	N(3A)-S(1A)-N(1A)	117.2(10)	C(32A)-C(31A)-C(30A)	104.8(9)
C(10)-C(9)-C(12)	108.0(3)	N(4A)-S(1A)-N(1A)	116.9(10)	O(4A)-C(32A)-C(31A)	107.9(10)
C(11)-C(9)-C(12)	107.1(3)	N(2A)-S(1A)-N(1A)	93.2(6)		
C(13)-N(4)-S(1)	129.1(2)	N(3A)-S(1A)-Li(1A)	48.2(4)		

XRD-Analysis of 2e

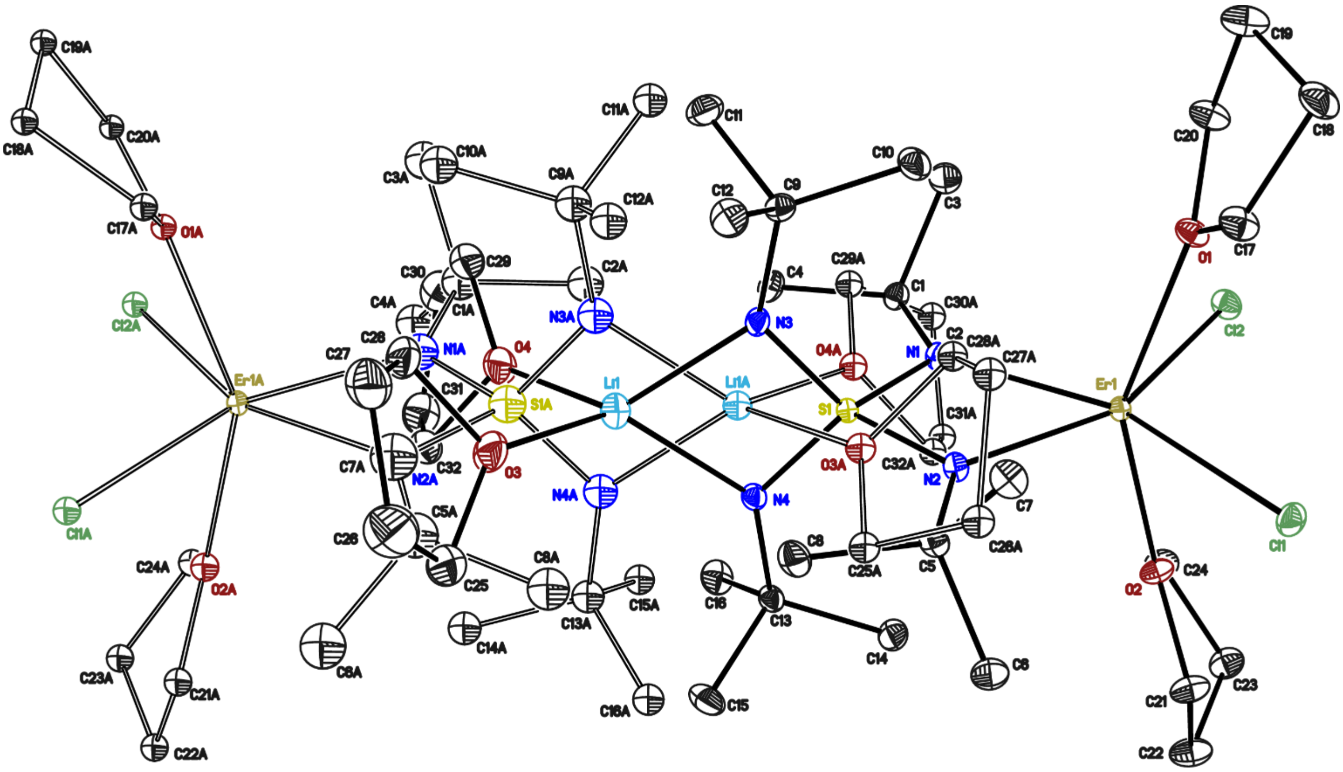

Figure S10 Asymmetric unit of **2e**. Anisotropic displacement parameters are depicted on a probability level of 50%. Hydrogen atoms are omitted for clarity. The whole molecule is disordered and was refined on two positions. The occupancy of the main positions was refined to 0.9879(3). For the refinement distance restraints and restraints for the anisotropic displacement parameters were used. The structure was refined as an inversion twin.

Table S22 Bond length of **2e** in Å.

**bond**	**length [Å]**	**bond**	**length [Å]**
Er(1)-N(2)	2.3016(17)	Er(1A)-N(1A)	2.307(10)
Er(1)-N(1)	2.3070(17)	Er(1A)-N(2A)	2.309(10)
Er(1)-O(1)	2.3904(15)	Er(1A)-O(2A)	2.391(9)
Er(1)-O(2)	2.3914(15)	Er(1A)-O(1A)	2.393(9)
Er(1)-Cl(2)	2.6118(7)	Er(1A)-Cl(2A)	2.611(10)
Er(1)-Cl(1)	2.6190(7)	Er(1A)-Cl(1A)	2.616(10)
Er(1)-S(1)	3.1039(6)	Er(1A)-S(1A)	3.108(9)
Li(1)-N(3)	1.974(4)	Li(1A)-N(3A)	1.977(11)
Li(1)-O(3)	1.978(4)	Li(1A)-N(4A)	1.977(11)
Li(1)-O(4)	1.979(4)	Li(1A)-O(3A)	1.982(10)
Li(1)-N(4)	1.982(4)	Li(1A)-O(4A)	1.984(10)
Li(1)-S(1)	2.638(3)	Li(1A)-S(1A)	2.635(10)
S(1)-N(4)	1.5667(18)	S(1A)-N(3A)	1.562(10)
S(1)-N(3)	1.5673(18)	S(1A)-N(4A)	1.564(10)
S(1)-N(1)	1.6276(17)	S(1A)-N(1A)	1.631(10)
S(1)-N(2)	1.6327(18)	S(1A)-N(2A)	1.632(10)
N(1)-C(1)	1.503(3)	N(1A)-C(1A)	1.492(10)
C(1)-C(2)	1.536(3)	C(1A)-C(2A)	1.534(10)
C(1)-C(4)	1.538(3)	C(1A)-C(4A)	1.535(10)
C(1)-C(3)	1.540(3)	C(1A)-C(3A)	1.535(10)
N(2)-C(5)	1.500(3)	N(2A)-C(5A)	1.491(10)
C(5)-C(7)	1.535(3)	C(5A)-C(8A)	1.534(10)
C(5)-C(8)	1.536(3)	C(5A)-C(7A)	1.535(10)
C(5)-C(6)	1.537(3)	C(5A)-C(6A)	1.536(10)
N(3)-C(9)	1.475(3)	N(3A)-C(9A)	1.487(10)
C(9)-C(10)	1.529(3)	C(9A)-C(10A)	1.534(10)
C(9)-C(11)	1.538(3)	C(9A)-C(12A)	1.536(10)
C(9)-C(12)	1.539(3)	C(9A)-C(11A)	1.536(10)
N(4)-C(13)	1.478(3)	N(4A)-C(13A)	1.486(10)
C(13)-C(14)	1.526(3)	C(13A)-C(14A)	1.533(10)
C(13)-C(15)	1.533(3)	C(13A)-C(16A)	1.537(10)
C(13)-C(16)	1.537(3)	C(13A)-C(15A)	1.537(10)
O(1)-C(17)	1.458(3)	O(1A)-C(20A)	1.446(10)
O(1)-C(20)	1.467(2)	O(1A)-C(17A)	1.449(10)
C(17)-C(18)	1.508(3)	C(17A)-C(18A)	1.514(11)
C(18)-C(19)	1.517(3)	C(18A)-C(19A)	1.526(11)
C(19)-C(20)	1.518(3)	C(19A)-C(20A)	1.509(10)
O(2)-C(24)	1.461(2)	O(2A)-C(24A)	1.448(10)
O(2)-C(21)	1.474(2)	O(2A)-C(21A)	1.448(10)
C(21)-C(22)	1.517(3)	C(21A)-C(22A)	1.511(11)
C(22)-C(23)	1.522(3)	C(22A)-C(23A)	1.527(11)
C(23)-C(24)	1.511(3)	C(23A)-C(24A)	1.514(11)
O(3)-C(25)	1.429(3)	O(3A)-C(25A)	1.448(10)
O(3)-C(28)	1.437(3)	O(3A)-C(28A)	1.449(10)
C(25)-C(26)	1.525(4)	C(25A)-C(26A)	1.511(11)
C(26)-C(27)	1.528(4)	C(26A)-C(27A)	1.528(11)
C(27)-C(28)	1.517(4)	C(27A)-C(28A)	1.516(11)
O(4)-C(32)	1.432(3)	O(4A)-C(29A)	1.448(10)
O(4)-C(29)	1.436(3)	O(4A)-C(32A)	1.448(10)
C(29)-C(30)	1.512(3)	C(29A)-C(30A)	1.511(11)
C(30)-C(31)	1.542(4)	C(30A)-C(31A)	1.527(11)
C(31)-C(32)	1.518(4)	C(31A)-C(32A)	1.515(11)

Table S23 Bond angles of **2e**.

N(2)-Er(1)-N(1)	61.61(5)	C(13)-N(4)-Li(1)	135.31(18)	N(4A)-S(1A)-Li(1A)	48.3(4)
N(2)-Er(1)-O(1)	101.64(6)	S(1)-N(4)-Li(1)	95.34(14)	N(1A)-S(1A)-Li(1A)	133.5(9)
N(1)-Er(1)-O(1)	103.28(6)	N(4)-C(13)-C(14)	116.33(18)	N(2A)-S(1A)-Li(1A)	133.7(9)
N(2)-Er(1)-O(2)	103.82(6)	N(4)-C(13)-C(15)	106.64(17)	N(3A)-S(1A)-Er(1A)	131.9(9)
N(1)-Er(1)-O(2)	102.15(6)	C(14)-C(13)-C(15)	108.24(19)	N(4A)-S(1A)-Er(1A)	131.5(9)
O(1)-Er(1)-O(2)	150.29(5)	N(4)-C(13)-C(16)	109.58(18)	N(1A)-S(1A)-Er(1A)	46.3(4)
N(2)-Er(1)-Cl(2)	157.04(4)	C(14)-C(13)-C(16)	108.63(18)	N(2A)-S(1A)-Er(1A)	46.4(4)
N(1)-Er(1)-Cl(2)	95.62(4)	C(15)-C(13)-C(16)	107.04(18)	Li(1A)-S(1A)-Er(1A)	179.8(9)
O(1)-Er(1)-Cl(2)	79.66(4)	C(17)-O(1)-C(20)	108.59(15)	C(1A)-N(1A)-S(1A)	122.6(14)
O(2)-Er(1)-Cl(2)	82.82(4)	C(17)-O(1)-Er(1)	125.19(12)	C(1A)-N(1A)-Er(1A)	134.0(14)
N(2)-Er(1)-Cl(1)	96.21(4)	C(20)-O(1)-Er(1)	126.06(12)	S(1A)-N(1A)-Er(1A)	102.9(6)
N(1)-Er(1)-Cl(1)	157.71(4)	O(1)-C(17)-C(18)	105.75(18)	N(1A)-C(1A)-C(2A)	111.3(14)
O(1)-Er(1)-Cl(1)	82.38(4)	C(17)-C(18)-C(19)	102.1(2)	N(1A)-C(1A)-C(4A)	110.7(14)
O(2)-Er(1)-Cl(1)	79.90(4)	C(18)-C(19)-C(20)	102.25(19)	C(2A)-C(1A)-C(4A)	108.0(13)
Cl(2)-Er(1)-Cl(1)	106.636(19)	O(1)-C(20)-C(19)	105.43(18)	N(1A)-C(1A)-C(3A)	110.8(14)
N(2)-Er(1)-S(1)	30.85(4)	C(24)-O(2)-C(21)	108.46(15)	C(2A)-C(1A)-C(3A)	108.0(13)
N(1)-Er(1)-S(1)	30.75(4)	C(24)-O(2)-Er(1)	125.30(12)	C(4A)-C(1A)-C(3A)	107.9(13)
O(1)-Er(1)-S(1)	104.51(4)	C(21)-O(2)-Er(1)	125.95(12)	C(5A)-N(2A)-S(1A)	122.9(14)
O(2)-Er(1)-S(1)	105.20(4)	O(2)-C(21)-C(22)	105.12(18)	C(5A)-N(2A)-Er(1A)	134.3(14)
Cl(2)-Er(1)-S(1)	126.32(2)	C(21)-C(22)-C(23)	101.92(19)	S(1A)-N(2A)-Er(1A)	102.8(6)
Cl(1)-Er(1)-S(1)	127.04(2)	C(24)-C(23)-C(22)	101.50(19)	N(2A)-C(5A)-C(8A)	111.1(14)
N(3)-Li(1)-O(3)	116.5(2)	O(2)-C(24)-C(23)	105.30(17)	N(2A)-C(5A)-C(7A)	111.0(14)
N(3)-Li(1)-O(4)	129.13(19)	C(25)-O(3)-C(28)	105.37(18)	C(8A)-C(5A)-C(7A)	108.1(13)
O(3)-Li(1)-O(4)	93.07(16)	C(25)-O(3)-Li(1)	134.95(19)	N(2A)-C(5A)-C(6A)	110.9(14)
N(3)-Li(1)-N(4)	72.49(13)	C(28)-O(3)-Li(1)	119.23(18)	C(8A)-C(5A)-C(6A)	107.8(13)
O(3)-Li(1)-N(4)	130.92(19)	O(3)-C(25)-C(26)	105.3(2)	C(7A)-C(5A)-C(6A)	107.8(13)
O(4)-Li(1)-N(4)	119.0(2)	C(25)-C(26)-C(27)	104.9(2)	C(9A)-N(3A)-S(1A)	130.5(15)
N(3)-Li(1)-S(1)	36.24(8)	C(28)-C(27)-C(26)	103.5(2)	C(9A)-N(3A)-Li(1A)	133.6(14)
O(3)-Li(1)-S(1)	133.13(19)	O(3)-C(28)-C(27)	105.1(2)	S(1A)-N(3A)-Li(1A)	95.5(6)
O(4)-Li(1)-S(1)	133.71(19)	C(32)-O(4)-C(29)	104.83(17)	N(3A)-C(9A)-C(10A)	111.5(14)
N(4)-Li(1)-S(1)	36.25(8)	C(32)-O(4)-Li(1)	120.86(18)	N(3A)-C(9A)-C(12A)	110.8(14)
N(4)-S(1)-N(3)	96.55(8)	C(29)-O(4)-Li(1)	134.15(18)	C(10A)-C(9A)-C(12A)	107.9(13)
N(4)-S(1)-N(1)	117.65(11)	O(4)-C(29)-C(30)	105.9(2)	N(3A)-C(9A)-C(11A)	110.6(14)
N(3)-S(1)-N(1)	117.14(9)	C(29)-C(30)-C(31)	104.5(2)	C(10A)-C(9A)-C(11A)	108.1(13)
N(4)-S(1)-N(2)	117.08(9)	C(32)-C(31)-C(30)	103.1(2)	C(12A)-C(9A)-C(11A)	107.7(13)
N(3)-S(1)-N(2)	117.47(11)	O(4)-C(32)-C(31)	104.83(19)	C(13A)-N(4A)-S(1A)	131.1(15)
N(1)-S(1)-N(2)	92.75(7)	N(1A)-Er(1A)-N(2A)	61.6(4)	C(13A)-N(4A)-Li(1A)	133.4(14)
N(4)-S(1)-Li(1)	48.41(11)	N(1A)-Er(1A)-O(2A)	102.2(8)	S(1A)-N(4A)-Li(1A)	95.5(6)
N(3)-S(1)-Li(1)	48.14(11)	N(2A)-Er(1A)-O(2A)	103.3(8)	N(4A)-C(13A)-C(14A)	111.9(14)
N(1)-S(1)-Li(1)	133.61(11)	N(1A)-Er(1A)-O(1A)	103.3(8)	N(4A)-C(13A)-C(16A)	110.6(14)
N(2)-S(1)-Li(1)	133.64(11)	N(2A)-Er(1A)-O(1A)	101.7(8)	C(14A)-C(13A)-C(16A)	108.0(13)
N(4)-S(1)-Er(1)	131.85(7)	O(2A)-Er(1A)-O(1A)	150.6(8)	N(4A)-C(13A)-C(15A)	110.8(14)
N(3)-S(1)-Er(1)	131.60(7)	N(1A)-Er(1A)-Cl(2A)	96.1(6)	C(14A)-C(13A)-C(15A)	107.7(13)
N(1)-S(1)-Er(1)	46.45(6)	N(2A)-Er(1A)-Cl(2A)	157.5(6)	C(16A)-C(13A)-C(15A)	107.5(13)
N(2)-S(1)-Er(1)	46.30(6)	O(2A)-Er(1A)-Cl(2A)	82.9(6)	C(20A)-O(1A)-C(17A)	108.5(11)
Li(1)-S(1)-Er(1)	179.74(11)	O(1A)-Er(1A)-Cl(2A)	79.9(6)	C(20A)-O(1A)-Er(1A)	125.6(14)
C(1)-N(1)-S(1)	122.15(14)	N(1A)-Er(1A)-Cl(1A)	157.2(6)	C(17A)-O(1A)-Er(1A)	125.3(14)
C(1)-N(1)-Er(1)	135.04(13)	N(2A)-Er(1A)-Cl(1A)	95.8(6)	O(1A)-C(17A)-C(18A)	105.1(14)
S(1)-N(1)-Er(1)	102.79(8)	O(2A)-Er(1A)-Cl(1A)	79.7(6)	C(17A)-C(18A)-C(19A)	102.3(14)
N(1)-C(1)-C(2)	107.58(17)	O(1A)-Er(1A)-Cl(1A)	82.5(6)	C(20A)-C(19A)-C(18A)	103.9(14)
N(1)-C(1)-C(4)	116.14(17)	Cl(2A)-Er(1A)-Cl(1A)	106.6(7)	O(1A)-C(20A)-C(19A)	107.7(10)
C(2)-C(1)-C(4)	108.43(19)	N(1A)-Er(1A)-S(1A)	30.8(3)	C(24A)-O(2A)-C(21A)	108.7(10)
N(1)-C(1)-C(3)	108.71(17)	N(2A)-Er(1A)-S(1A)	30.8(3)	C(24A)-O(2A)-Er(1A)	125.7(14)
C(2)-C(1)-C(3)	108.02(18)	O(2A)-Er(1A)-S(1A)	104.8(6)	C(21A)-O(2A)-Er(1A)	125.0(14)
C(4)-C(1)-C(3)	107.71(18)	O(1A)-Er(1A)-S(1A)	104.6(6)	O(2A)-C(21A)-C(22A)	107.3(12)
C(5)-N(2)-S(1)	122.34(14)	Cl(2A)-Er(1A)-S(1A)	126.9(6)	C(21A)-C(22A)-C(23A)	102.7(14)
C(5)-N(2)-Er(1)	134.81(13)	Cl(1A)-Er(1A)-S(1A)	126.5(6)	C(24A)-C(23A)-C(22A)	101.9(14)
S(1)-N(2)-Er(1)	102.85(8)	N(3A)-Li(1A)-N(4A)	72.4(5)	O(2A)-C(24A)-C(23A)	105.7(14)
N(2)-C(5)-C(7)	108.25(17)	N(3A)-Li(1A)-O(3A)	117.9(11)	C(25A)-O(3A)-C(28A)	108.7(10)
N(2)-C(5)-C(8)	116.15(18)	N(4A)-Li(1A)-O(3A)	130.0(13)	C(25A)-O(3A)-Li(1A)	130.7(17)
C(7)-C(5)-C(8)	108.23(18)	N(3A)-Li(1A)-O(4A)	129.9(13)	C(28A)-O(3A)-Li(1A)	118.2(15)
N(2)-C(5)-C(6)	109.18(17)	N(4A)-Li(1A)-O(4A)	117.8(11)	O(3A)-C(25A)-C(26A)	106.9(12)
C(7)-C(5)-C(6)	107.44(18)	O(3A)-Li(1A)-O(4A)	93.2(10)	C(25A)-C(26A)-C(27A)	102.5(14)
C(8)-C(5)-C(6)	107.28(18)	N(3A)-Li(1A)-S(1A)	36.2(3)	C(28A)-C(27A)-C(26A)	101.5(13)
C(9)-N(3)-S(1)	129.66(15)	N(4A)-Li(1A)-S(1A)	36.2(3)	O(3A)-C(28A)-C(27A)	105.8(14)
C(9)-N(3)-Li(1)	134.69(19)	O(3A)-Li(1A)-S(1A)	133.7(9)	C(29A)-O(4A)-C(32A)	108.4(10)
S(1)-N(3)-Li(1)	95.61(14)	O(4A)-Li(1A)-S(1A)	133.1(9)	C(29A)-O(4A)-Li(1A)	130.1(17)
N(3)-C(9)-C(10)	116.51(19)	N(3A)-S(1A)-N(4A)	96.6(7)	C(32A)-O(4A)-Li(1A)	118.1(15)
N(3)-C(9)-C(11)	106.91(18)	N(3A)-S(1A)-N(1A)	117.2(13)	O(4A)-C(29A)-C(30A)	107.3(12)
C(10)-C(9)-C(11)	108.6(2)	N(4A)-S(1A)-N(1A)	117.8(13)	C(29A)-C(30A)-C(31A)	102.7(14)
N(3)-C(9)-C(12)	109.33(19)	N(3A)-S(1A)-N(2A)	117.6(13)	C(32A)-C(31A)-C(30A)	101.7(13)
C(10)-C(9)-C(12)	108.20(19)	N(4A)-S(1A)-N(2A)	116.7(13)	O(4A)-C(32A)-C(31A)	105.9(14)
C(11)-C(9)-C(12)	106.83(19)	N(1A)-S(1A)-N(2A)	92.8(7)		
C(13)-N(4)-S(1)	129.33(15)	N(3A)-S(1A)-Li(1A)	48.3(4)		

XRD-Analysis of 3b

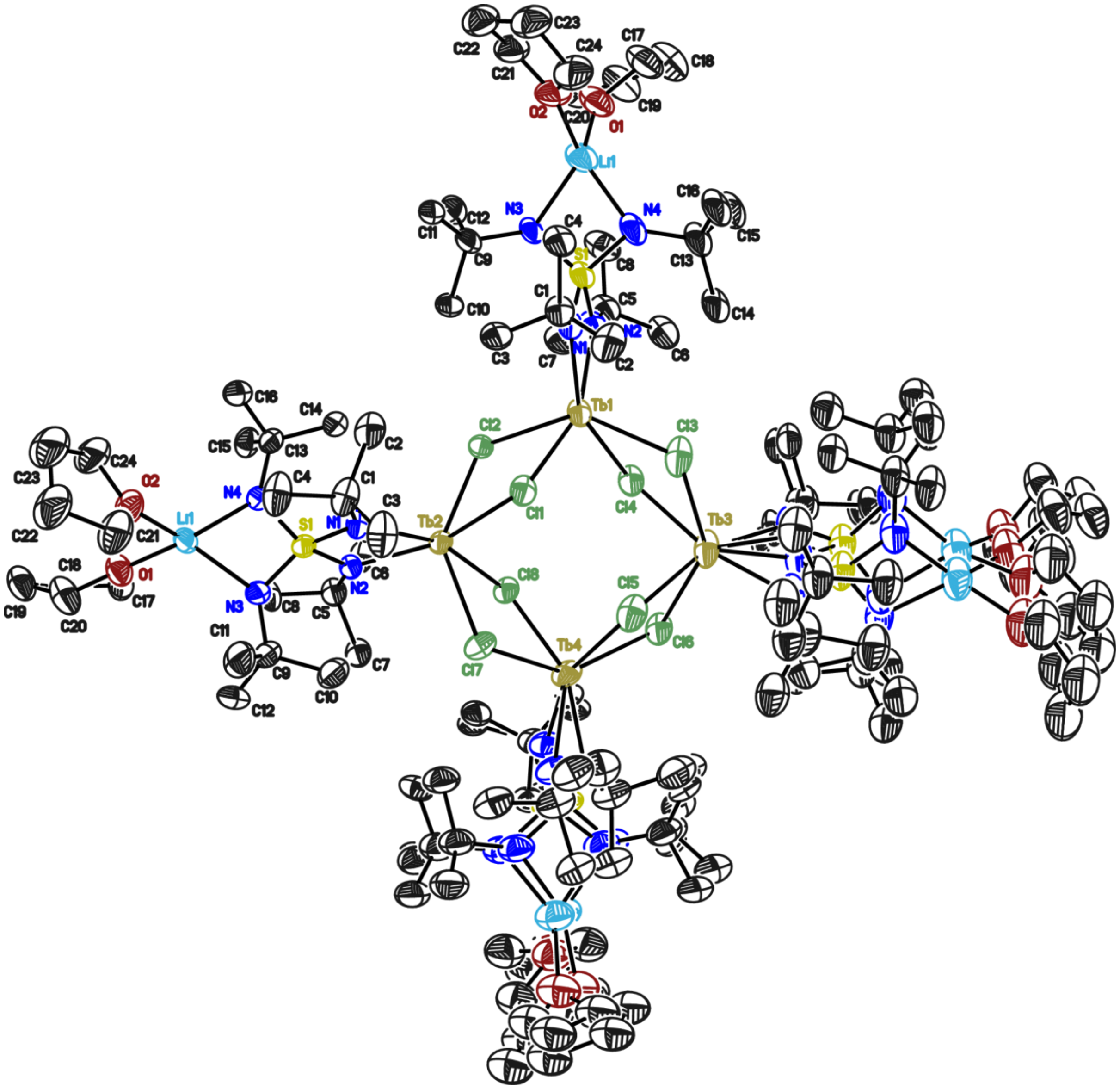

Figure S11 Asymmetric unit of **3b**. Anisotropic displacement parameters are depicted on a probability level of 50%. Hydrogen atoms are omitted for clarity. The ligands coordinating Tb3 and Tb4, including the coordinated lithium atom and the thf molecules, are disordered and were refined on two positions each. The occupancy of the main position for the ligand coordinating to Tb3 was refined to 0.584(5) and the one coordinating to Tb4 with 0.512(6), respectively. For the refinement, distance restraints and restraints for the anisotropic displacement parameters were used. The crystal contained solvent channels filled with disordered toluene molecules, which were treated using SQUEEZE from the PLATON software package.[1]

Table S24 Bond length of **3b** in Å.

bond	length [Å]	bond	length [Å]
Tb(2)-N22	2.294(7)	N43-C133	1.479(14)
Tb(2)-N12	2.308(7)	N43-Li13	1.999(16)
Tb(2)-Cl(7)	2.688(3)	C133-C143	1.515(14)
Tb(2)-Cl(2)	2.740(3)	C133-C163	1.522(14)
Tb(2)-Cl(1)	2.747(3)	C133-C153	1.523(14)
Tb(2)-Cl(8)	2.770(3)	Li13-O13	1.980(15)
Tb(2)-S12	3.106(3)	Li13-O23	1.984(15)
Cl(1)-Tb(1)	2.730(3)	O13-C173	1.405(14)
Cl(2)-Tb(1)	2.746(3)	O13-C203	1.408(14)
Tb(1)-N21	2.315(8)	C173-C183	1.487(15)
Tb(1)-N11	2.319(7)	C183-C193	1.514(17)
Tb(1)-Cl(3)	2.717(4)	C193-C203	1.472(15)
Tb(1)-Cl(4)	2.752(3)	O23-C213	1.391(14)
Tb(1)-S11	3.113(3)	O23-C243	1.407(14)
Cl(3)-Tb(3)	2.723(3)	C213-C223	1.472(15)
Cl(4)-Tb(3)	2.779(4)	C223-C233	1.508(17)
Tb(3)-N13	2.288(12)	C233-C243	1.469(15)
Tb(3)-N24	2.293(11)	S14-N44	1.558(11)
Tb(3)-N23	2.343(12)	S14-N34	1.561(11)
Tb(3)-N14	2.355(11)	S14-N24	1.614(11)
Tb(3)-Cl(5)	2.713(4)	S14-N14	1.614(11)
Tb(3)-Cl(6)	2.741(3)	N14-C14	1.510(14)
Tb(3)-S13	3.116(10)	C14-C44	1.532(13)
Tb(3)-S14	3.117(7)	C14-C24	1.536(13)
Cl(5)-Tb(4)	2.769(3)	C14-C34	1.540(13)
Cl(6)-Tb(4)	2.737(3)	N24-C54	1.475(14)
Tb(4)-N25	2.276(12)	C54-C64	1.528(13)
Tb(4)-N15	2.325(12)	C54-C84	1.530(13)
Tb(4)-N26	2.327(12)	C54-C74	1.534(13)
Tb(4)-N16	2.330(12)	N34-C94	1.485(13)
Tb(4)-Cl(8)	2.696(3)	N34-Li14	1.998(15)
Tb(4)-Cl(7)	2.735(3)	C94-C124	1.519(13)
Tb(4)-S15	3.085(10)	C94-C114	1.523(13)
Tb(4)-S16	3.117(10)	C94-C104	1.527(13)
S11-N31	1.548(8)	N44-C134	1.489(13)
S11-N41	1.558(8)	N44-Li14	2.001(15)
S11-N11	1.617(8)	C134-C154	1.519(13)
S11-N21	1.623(7)	C134-C144	1.526(13)
N11-C11	1.498(10)	C134-C164	1.532(13)
C11-C31	1.524(11)	Li14-O14	1.987(14)
C11-C21	1.524(11)	Li14-O24	1.990(15)
C11-C41	1.538(11)	O14-C174	1.410(13)
N21-C51	1.498(11)	O14-C204	1.412(13)
C51-C81	1.528(11)	C174-C184	1.501(15)
C51-C61	1.529(11)	C184-C194	1.533(16)
C51-C71	1.536(11)	C194-C204	1.477(14)
N31-C91	1.473(11)	O24-C214	1.382(13)
N31-Li11	2.004(14)	O24-C244	1.415(13)
C91-C121	1.520(11)	C214-C224	1.476(15)
C91-C111	1.525(11)	C224-C234	1.528(16)
C91-C101	1.532(10)	C234-C244	1.486(15)
N41-C131	1.490(11)	S15-N45	1.551(11)
N41-Li11	1.993(14)	S15-N35	1.558(12)
C131-C151	1.519(12)	S15-N15	1.617(12)
C131-C141	1.520(11)	S15-N25	1.625(11)
C131-C161	1.531(11)	N15-C15	1.501(13)
Li11-O11	1.976(13)	C15-C25	1.526(13)
Li11-O21	1.995(13)	C15-C35	1.527(13)
O11-C201	1.399(11)	C15-C45	1.531(13)
O11-C171	1.415(11)	N25-C55	1.490(14)
C171-C181	1.462(13)	C55-C85	1.527(13)
C181-C191	1.506(14)	C55-C65	1.534(14)
C191-C201	1.478(13)	C55-C75	1.538(13)
O21-C241	1.389(11)	N35-C95	1.481(13)
O21-C211	1.424(11)	N35-Li15	1.994(15)
C211-C221	1.482(12)	C95-C125	1.523(13)
C221-C231	1.517(14)	C95-C115	1.525(14)
C231-C241	1.482(12)	C95-C105	1.526(13)
S12-N32	1.552(7)	N45-C135	1.481(14)
S12-N42	1.555(7)	N45-Li15	1.993(16)
S12-N22	1.624(7)	C135-C155	1.517(14)
S12-N12	1.626(7)	C135-C145	1.525(13)
N12-C12	1.505(10)	C135-C165	1.532(14)
C12-C32	1.524(11)	Li15-O15	1.990(15)
C12-C42	1.525(11)	Li15-O25	1.994(14)

C12-C22	1.539(11)	O15-C175	1.380(13)
N22-C52	1.501(10)	O15-C205	1.393(14)
C52-C62	1.524(11)	C175-C185	1.473(15)
C52-C82	1.533(11)	C185-C195	1.514(17)
C52-C72	1.535(11)	C195-C205	1.473(15)
N32-C92	1.483(11)	O25-C215	1.393(14)
N32-Li12	2.003(13)	O25-C245	1.416(14)
C92-C102	1.511(11)	C215-C225	1.475(15)
C92-C122	1.519(11)	C225-C235	1.520(16)
C92-C112	1.533(11)	C235-C245	1.465(15)
N42-C132	1.483(10)	S16-N36	1.552(12)
N42-Li12	2.014(13)	S16-N46	1.562(12)
C132-C142	1.519(10)	S16-N16	1.615(12)
C132-C162	1.530(11)	S16-N26	1.634(12)
C132-C152	1.531(11)	N16-C16	1.501(13)
Li12-O12	1.992(12)	C16-C26	1.529(13)
Li12-O22	2.014(12)	C16-C36	1.529(14)
O12-C202	1.410(10)	C16-C46	1.530(13)
O12-C172	1.414(10)	N26-C56	1.507(14)
C172-C182	1.492(11)	C56-C76	1.528(14)
C182-C192	1.511(13)	C56-C86	1.528(13)
C192-C202	1.483(12)	C56-C66	1.535(14)
O22-C242	1.399(11)	N36-C96	1.480(14)
O22-C212	1.406(11)	N36-Li16	2.008(16)
C212-C222	1.478(12)	C96-C106	1.512(13)
C222-C232	1.474(14)	C96-C116	1.521(14)
C232-C242	1.473(13)	C96-C126	1.525(14)
S13-N43	1.556(12)	N46-C136	1.486(14)
S13-N33	1.556(12)	N46-Li16	1.990(15)
S13-N13	1.612(12)	C136-C166	1.517(14)
S13-N23	1.621(12)	C136-C146	1.520(13)
N13-C13	1.486(14)	C136-C156	1.523(13)
C13-C23	1.527(14)	Li16-O16	1.967(14)
C13-C43	1.530(14)	Li16-O26	1.993(15)
C13-C33	1.539(14)	O16-C206	1.400(14)
N23-C53	1.504(14)	O16-C176	1.421(14)
C53-C73	1.530(14)	C176-C186	1.496(15)
C53-C83	1.531(14)	C186-C196	1.535(17)
C53-C63	1.532(14)	C196-C206	1.492(15)
N33-C93	1.483(14)	O26-C246	1.390(14)
N33-Li13	1.999(16)	O26-C216	1.393(14)
C93-C113	1.524(14)	C216-C226	1.471(15)
C93-C103	1.527(14)	C226-C236	1.559(16)
C93-C123	1.528(14)	C236-C246	1.460(15)

Table S25 Bond angles of **3b**.

N22-Tb(2)-N12	61.2(2)	O21-Li11-N31	117.4(7)	C64-C54-C74	107.0(13)
N22-Tb(2)-Cl(7)	102.9(2)	C201-O11-C171	103.9(9)	C84-C54-C74	109.2(14)
N12-Tb(2)-Cl(7)	100.9(2)	C201-O11-Li11	135.4(8)	C94-N34-S14	131.2(10)
N22-Tb(2)-Cl(2)	109.4(2)	C171-O11-Li11	120.6(8)	C94-N34-Li14	132.8(10)
N12-Tb(2)-Cl(2)	114.0(2)	O11-C171-C181	106.7(9)	S14-N34-Li14	95.6(7)
Cl(7)-Tb(2)-Cl(2)	140.60(9)	C171-C181-C191	104.8(10)	N34-C94-C124	108.8(13)
N22-Tb(2)-Cl(1)	157.30(18)	C201-C191-C181	103.1(9)	N34-C94-C114	107.5(13)
N12-Tb(2)-Cl(1)	96.42(18)	O11-C201-C191	106.9(10)	C124-C94-C114	109.3(14)
Cl(7)-Tb(2)-Cl(1)	84.12(10)	C241-O21-C211	107.4(8)	N34-C94-C104	114.0(12)
Cl(2)-Tb(2)-Cl(1)	74.78(9)	C241-O21-Li11	132.6(8)	C124-C94-C104	110.1(14)
N22-Tb(2)-Cl(8)	97.95(18)	C211-O21-Li11	118.8(7)	C114-C94-C104	106.9(13)
N12-Tb(2)-Cl(8)	158.06(19)	O21-C211-C221	105.7(9)	C134-N44-S14	130.4(10)
Cl(7)-Tb(2)-Cl(8)	75.88(9)	C211-C221-C231	102.7(9)	C134-N44-Li14	134.0(10)
Cl(2)-Tb(2)-Cl(8)	77.84(9)	C241-C231-C221	103.8(9)	S14-N44-Li14	95.6(7)
Cl(1)-Tb(2)-Cl(8)	104.72(9)	O21-C241-C231	109.5(9)	N44-C134-C154	108.8(14)
N22-Tb(2)-S12	30.55(17)	N32-S12-N42	97.7(4)	N44-C134-C144	113.9(13)
N12-Tb(2)-S12	30.67(17)	N32-S12-N22	118.2(4)	C154-C134-C144	108.1(14)
Cl(7)-Tb(2)-S12	104.90(9)	N42-S12-N22	116.1(5)	N44-C134-C164	108.5(14)
Cl(2)-Tb(2)-S12	114.40(8)	N32-S12-N12	117.5(5)	C154-C134-C164	108.5(15)
Cl(1)-Tb(2)-S12	126.89(8)	N42-S12-N12	116.7(4)	C144-C134-C164	108.9(14)
Cl(8)-Tb(2)-S12	128.35(8)	N22-S12-N12	92.2(4)	O14-Li14-O24	93.1(9)
Tb(1)-Cl(1)-Tb(2)	103.70(10)	N32-S12-Tb(2)	134.2(3)	O14-Li14-N34	127.5(11)
Tb(2)-Cl(2)-Tb(1)	103.45(10)	N42-S12-Tb(2)	128.0(3)	O24-Li14-N34	123.5(10)
N21-Tb(1)-N11	60.9(3)	N22-S12-Tb(2)	45.9(3)	O14-Li14-N44	122.9(10)
N21-Tb(1)-Cl(3)	114.3(3)	N12-S12-Tb(2)	46.4(3)	O24-Li14-N44	121.5(11)
N11-Tb(1)-Cl(3)	97.5(2)	C12-N12-S12	122.8(6)	N34-Li14-N44	71.5(7)
N21-Tb(1)-Cl(1)	154.5(2)	C12-N12-Tb(2)	134.2(5)	C174-O14-C204	106.2(11)
N11-Tb(1)-Cl(1)	98.9(2)	S12-N12-Tb(2)	102.9(3)	C174-O14-Li14	129.3(12)
Cl(3)-Tb(1)-Cl(1)	81.86(11)	N12-C12-C32	107.6(8)	C204-O14-Li14	123.3(12)
N21-Tb(1)-Cl(2)	98.5(2)	N12-C12-C42	115.7(8)	O14-C174-C184	104.4(13)
N11-Tb(1)-Cl(2)	114.9(2)	C32-C12-C42	109.1(9)	C174-C184-C194	96.3(14)
Cl(3)-Tb(1)-Cl(2)	142.35(10)	N12-C12-C22	108.3(8)	C204-C194-C184	98.3(13)
Cl(1)-Tb(1)-Cl(2)	74.96(9)	C32-C12-C22	108.6(9)	O14-C204-C194	107.8(13)
N21-Tb(1)-Cl(4)	97.7(2)	C42-C12-C22	107.3(9)	C214-O24-C244	107.7(13)
N11-Tb(1)-Cl(4)	153.1(2)	C52-N22-S12	124.4(6)	C214-O24-Li14	131.0(13)
Cl(3)-Tb(1)-Cl(4)	75.55(9)	C52-N22-Tb(2)	131.9(5)	C244-O24-Li14	120.9(12)
Cl(1)-Tb(1)-Cl(4)	105.67(10)	S12-N22-Tb(2)	103.6(3)	O24-C214-C224	108.4(13)
Cl(2)-Tb(1)-Cl(4)	82.59(9)	N22-C52-C62	108.5(8)	C214-C224-C234	101.9(13)
N21-Tb(1)-S11	30.52(19)	N22-C52-C82	114.3(8)	C244-C234-C224	99.2(14)
N11-Tb(1)-S11	30.4(2)	C62-C52-C82	109.2(9)	O24-C244-C234	102.9(14)
Cl(3)-Tb(1)-S11	108.79(10)	N22-C52-C72	108.0(8)	N45-S15-N35	98.4(8)
Cl(1)-Tb(1)-S11	127.58(9)	C62-C52-C72	108.1(8)	N45-S15-N15	118.9(12)
Cl(2)-Tb(1)-S11	108.86(9)	C82-C52-C72	108.6(8)	N35-S15-N15	115.9(12)
Cl(4)-Tb(1)-S11	126.74(10)	C92-N32-S12	130.3(6)	N45-S15-N25	115.6(10)
Tb(1)-Cl(3)-Tb(3)	103.20(10)	C92-N32-Li12	134.1(7)	N35-S15-N25	116.0(11)
Tb(1)-Cl(4)-Tb(3)	100.88(11)	S12-N32-Li12	95.5(5)	N15-S15-N25	93.5(8)
N13-Tb(3)-N23	60.4(5)	N32-C92-C102	114.5(9)	N45-S15-Tb(4)	133.0(7)
N24-Tb(3)-N14	60.5(4)	N32-C92-C122	108.4(9)	N35-S15-Tb(4)	128.5(6)
N13-Tb(3)-Cl(5)	105.3(4)	C102-C92-C122	109.7(10)	N15-S15-Tb(4)	47.6(5)
N24-Tb(3)-Cl(5)	150.1(4)	N32-C92-C112	108.9(9)	N25-S15-Tb(4)	45.9(5)
N23-Tb(3)-Cl(5)	159.7(6)	C102-C92-C112	107.5(10)	C15-N15-S15	123.6(11)
N14-Tb(3)-Cl(5)	91.2(3)	C122-C92-C112	107.6(10)	C15-N15-Tb(4)	134.8(11)
N13-Tb(3)-Cl(3)	91.4(5)	C132-N42-S12	128.0(6)	S15-N15-Tb(4)	101.5(7)
N24-Tb(3)-Cl(3)	110.9(5)	C132-N42-Li12	136.6(7)	N15-C15-C25	109.6(15)
N23-Tb(3)-Cl(3)	109.0(7)	S12-N42-Li12	95.0(5)	N15-C15-C35	110.3(15)
N14-Tb(3)-Cl(3)	105.1(4)	N42-C132-C142	116.1(8)	C25-C15-C35	107.1(15)
Cl(5)-Tb(3)-Cl(3)	84.23(11)	N42-C132-C162	106.8(8)	N15-C15-C45	113.9(15)
N13-Tb(3)-Cl(6)	123.6(6)	C142-C132-C162	109.1(8)	C25-C15-C45	107.7(15)
N24-Tb(3)-Cl(6)	101.9(5)	N42-C132-C152	108.6(8)	C35-C15-C45	108.0(15)
N23-Tb(3)-Cl(6)	100.1(6)	C142-C132-C152	107.4(9)	C55-N25-S15	128.8(10)
N14-Tb(3)-Cl(6)	106.0(4)	C162-C132-C152	108.6(9)	C55-N25-Tb(4)	128.0(9)
Cl(5)-Tb(3)-Cl(6)	75.24(10)	O12-Li12-N32	119.2(6)	S15-N25-Tb(4)	103.3(6)
Cl(3)-Tb(3)-Cl(6)	142.84(10)	O12-Li12-O22	94.0(6)	N25-C55-C85	112.7(14)
N13-Tb(3)-Cl(4)	147.3(6)	N32-Li12-O22	125.9(7)	N25-C55-C65	111.6(15)
N24-Tb(3)-Cl(4)	105.9(3)	O12-Li12-N42	125.3(7)	C85-C55-C65	108.0(15)
N23-Tb(3)-Cl(4)	95.6(4)	N32-Li12-N42	71.3(5)	N25-C55-C75	108.4(14)
N14-Tb(3)-Cl(4)	165.9(3)	O22-Li12-N42	124.2(6)	C85-C55-C75	107.7(15)
Cl(5)-Tb(3)-Cl(4)	102.80(10)	C202-O12-C172	104.2(8)	C65-C55-C75	108.3(15)
Cl(3)-Tb(3)-Cl(4)	75.03(10)	C202-O12-Li12	124.7(7)	C95-N35-S15	129.6(11)
Cl(6)-Tb(3)-Cl(4)	79.71(10)	C172-O12-Li12	129.0(7)	C95-N35-Li15	135.8(11)
N13-Tb(3)-S13	30.0(3)	O12-C172-C182	107.3(8)	S15-N35-Li15	94.5(8)
N23-Tb(3)-S13	30.6(3)	C172-C182-C192	104.1(8)	N35-C95-C125	109.0(14)
Cl(5)-Tb(3)-S13	132.8(2)	C202-C192-C182	102.7(9)	N35-C95-C115	107.4(14)
Cl(3)-Tb(3)-S13	104.1(2)	O12-C202-C192	105.1(9)	C125-C95-C115	108.1(15)
Cl(6)-Tb(3)-S13	112.6(2)	C242-O22-C212	108.7(8)	N35-C95-C105	114.7(14)
Cl(4)-Tb(3)-S13	124.4(2)	C242-O22-Li12	123.2(8)	C125-C95-C105	108.5(14)

N24-Tb(3)-S14	30.1(3)	C212-O22-Li12	128.0(8)	C115-C95-C105	108.9(15)
N14-Tb(3)-S14	30.5(3)	O22-C212-C222	107.8(10)	C135-N45-S15	131.1(11)
Cl(5)-Tb(3)-S14	121.29(18)	C232-C222-C212	102.8(10)	C135-N45-Li15	134.0(11)
Cl(3)-Tb(3)-S14	110.19(15)	C242-C232-C222	105.8(10)	S15-N45-Li15	94.8(8)
Cl(6)-Tb(3)-S14	106.93(15)	O22-C242-C232	107.9(10)	N45-C135-C155	109.5(15)
Cl(4)-Tb(3)-S14	135.80(17)	N43-S13-N33	98.3(9)	N45-C135-C145	114.5(14)
Tb(3)-Cl(5)-Tb(4)	103.51(12)	N43-S13-N13	115.5(12)	C155-C135-C145	108.3(15)
Tb(4)-Cl(6)-Tb(3)	103.62(10)	N33-S13-N13	117.3(11)	N45-C135-C165	109.8(14)
N25-Tb(4)-N15	61.7(5)	N43-S13-N23	117.2(12)	C155-C135-C165	107.8(15)
N26-Tb(4)-N16	60.7(5)	N33-S13-N23	117.8(12)	C145-C135-C165	106.7(15)
N25-Tb(4)-Cl(8)	153.7(4)	N13-S13-N23	92.2(8)	O15-Li15-N35	122.4(11)
N15-Tb(4)-Cl(8)	96.5(5)	N43-S13-Tb(3)	125.1(7)	O15-Li15-N45	126.1(11)
N26-Tb(4)-Cl(8)	139.1(5)	N33-S13-Tb(3)	136.5(7)	N35-Li15-N45	72.3(7)
N16-Tb(4)-Cl(8)	96.2(5)	N13-S13-Tb(3)	45.2(5)	O15-Li15-O25	91.9(9)
N25-Tb(4)-Cl(7)	100.6(5)	N23-S13-Tb(3)	47.3(5)	N35-Li15-O25	129.8(11)
N15-Tb(4)-Cl(7)	119.3(9)	C13-N13-S13	125.7(11)	N45-Li15-O25	118.4(11)
N26-Tb(4)-Cl(7)	85.2(5)	C13-N13-Tb(3)	129.5(10)	C175-O15-C205	107.8(13)
N16-Tb(4)-Cl(7)	117.5(9)	S13-N13-Tb(3)	104.8(7)	C175-O15-Li15	129.6(14)
Cl(8)-Tb(4)-Cl(7)	76.34(9)	N13-C13-C23	109.8(16)	C205-O15-Li15	120.0(14)
N25-Tb(4)-Cl(6)	109.6(5)	N13-C13-C43	113.6(15)	O15-C175-C185	107.6(15)
N15-Tb(4)-Cl(6)	95.0(8)	C23-C13-C43	109.1(16)	C175-C185-C195	101.8(13)
N26-Tb(4)-Cl(6)	127.5(5)	N13-C13-C33	109.2(15)	C205-C195-C185	103.9(12)
N16-Tb(4)-Cl(6)	96.8(8)	C23-C13-C33	108.3(16)	O15-C205-C195	109.1(13)
Cl(8)-Tb(4)-Cl(6)	85.57(10)	C43-C13-C33	106.7(15)	C215-O25-C245	109.8(13)
Cl(7)-Tb(4)-Cl(6)	142.40(9)	C53-N23-S13	122.2(11)	C215-O25-Li15	128.1(14)
N25-Tb(4)-Cl(5)	101.2(4)	C53-N23-Tb(3)	135.1(11)	C245-O25-Li15	120.8(13)
N15-Tb(4)-Cl(5)	156.2(7)	S13-N23-Tb(3)	102.1(7)	O25-C215-C225	104.4(14)
N26-Tb(4)-Cl(5)	107.8(4)	N23-C53-C73	110.2(16)	C215-C225-C235	103.0(14)
N16-Tb(4)-Cl(5)	157.4(8)	N23-C53-C83	113.5(15)	C245-C235-C225	103.5(13)
Cl(8)-Tb(4)-Cl(5)	103.68(10)	C73-C53-C83	107.4(16)	O25-C245-C235	107.5(13)
Cl(7)-Tb(4)-Cl(5)	78.22(10)	N23-C53-C63	110.0(16)	N36-S16-N46	97.8(8)
Cl(6)-Tb(4)-Cl(5)	74.41(10)	C73-C53-C63	107.2(16)	N36-S16-N16	119.8(12)
N25-Tb(4)-S15	30.8(3)	C83-C53-C63	108.3(16)	N46-S16-N16	116.7(13)
N15-Tb(4)-S15	30.9(3)	C93-N33-S13	131.6(12)	N36-S16-N26	116.8(11)
Cl(8)-Tb(4)-S15	125.9(2)	C93-N33-Li13	133.3(11)	N46-S16-N26	114.6(11)
Cl(7)-Tb(4)-S15	112.5(2)	S13-N33-Li13	94.7(8)	N16-S16-N26	92.8(8)
Cl(6)-Tb(4)-S15	104.9(2)	N33-C93-C113	109.0(15)	N36-S16-Tb(4)	140.2(7)
Cl(5)-Tb(4)-S15	130.4(2)	N33-C93-C103	113.5(15)	N46-S16-Tb(4)	121.9(7)
N26-Tb(4)-S16	30.8(3)	C113-C93-C103	107.8(15)	N16-S16-Tb(4)	46.8(5)
N16-Tb(4)-S16	30.3(3)	N33-C93-C123	109.6(15)	N26-S16-Tb(4)	46.8(5)
Cl(8)-Tb(4)-S16	122.5(2)	C113-C93-C123	107.6(16)	C16-N16-S16	124.9(11)
Cl(7)-Tb(4)-S16	106.0(2)	C103-C93-C123	109.2(16)	C16-N16-Tb(4)	132.2(11)
Cl(6)-Tb(4)-S16	111.5(2)	C133-N43-S13	131.4(12)	S16-N16-Tb(4)	102.9(7)
Cl(5)-Tb(4)-S16	133.6(3)	C133-N43-Li13	133.2(12)	N16-C16-C26	110.1(15)
Tb(2)-Cl(7)-Tb(4)	102.50(11)	S13-N43-Li13	94.7(8)	N16-C16-C36	109.7(16)
Tb(4)-Cl(8)-Tb(2)	101.39(10)	N43-C133-C143	113.9(15)	C26-C16-C36	106.5(15)
N31-S11-N41	98.4(5)	N43-C133-C163	109.7(16)	N16-C16-C46	114.5(15)
N31-S11-N11	116.3(5)	C143-C133-C163	108.3(16)	C26-C16-C46	107.8(15)
N41-S11-N11	117.2(5)	N43-C133-C153	108.5(16)	C36-C16-C46	107.9(15)
N31-S11-N21	116.4(5)	C143-C133-C153	107.8(16)	C56-N26-S16	122.8(10)
N41-S11-N21	117.0(5)	C163-C133-C153	108.4(17)	C56-N26-Tb(4)	134.3(10)
N11-S11-N21	93.0(4)	O13-Li13-O23	94.0(10)	S16-N26-Tb(4)	102.4(7)
N31-S11-Tb(1)	129.3(3)	O13-Li13-N43	122.9(11)	N26-C56-C76	111.2(15)
N41-S11-Tb(1)	132.3(4)	O23-Li13-N43	122.6(12)	N26-C56-C86	114.5(14)
N11-S11-Tb(1)	46.5(3)	O13-Li13-N33	122.0(12)	C76-C56-C86	106.7(15)
N21-S11-Tb(1)	46.4(3)	O23-Li13-N33	126.3(11)	N26-C56-C66	107.6(15)
C11-N11-S11	123.7(6)	N43-Li13-N33	72.2(8)	C76-C56-C66	107.2(15)
C11-N11-Tb(1)	133.1(6)	C173-O13-C203	102.8(15)	C86-C56-C66	109.4(16)
S11-N11-Tb(1)	103.1(4)	C173-O13-Li13	131.4(14)	C96-N36-S16	133.3(11)
N11-C11-C31	108.1(8)	C203-O13-Li13	124.5(14)	C96-N36-Li16	131.8(11)
N11-C11-C21	110.1(8)	O13-C173-C183	102.7(16)	S16-N36-Li16	94.9(8)
C31-C11-C21	108.5(10)	C173-C183-C193	100.4(15)	N36-C96-C106	111.6(14)
N11-C11-C41	114.6(9)	C203-C193-C183	103.9(13)	N36-C96-C116	110.5(14)
C31-C11-C41	107.0(8)	O13-C203-C193	107.0(14)	C106-C96-C116	108.8(14)
C21-C11-C41	108.4(9)	C213-O23-C243	105.7(15)	N36-C96-C126	109.9(15)
C51-N21-S11	123.8(6)	C213-O23-Li13	130.4(14)	C106-C96-C126	108.0(14)
C51-N21-Tb(1)	133.1(6)	C243-O23-Li13	122.7(14)	C116-C96-C126	108.0(15)
S11-N21-Tb(1)	103.0(4)	O23-C213-C223	108.6(14)	C136-N46-S16	130.2(11)
N21-C51-C81	115.1(9)	C213-C223-C233	103.8(13)	C136-N46-Li16	134.3(11)
N21-C51-C61	109.3(9)	C243-C233-C223	103.4(14)	S16-N46-Li16	95.3(8)
C81-C51-C61	109.2(10)	O23-C243-C233	105.3(16)	N46-C136-C166	110.7(15)
N21-C51-C71	107.1(9)	N44-S14-N34	97.1(7)	N46-C136-C146	113.2(14)
C81-C51-C71	107.7(10)	N44-S14-N24	115.5(9)	C166-C136-C146	106.3(15)
C61-C51-C71	108.2(9)	N34-S14-N24	114.8(10)	N46-C136-C156	106.4(14)
C91-N31-S11	130.7(7)	N44-S14-N14	118.5(9)	C166-C136-C156	111.8(16)
C91-N31-Li11	134.4(7)	N34-S14-N14	119.4(9)	C146-C136-C156	108.5(15)
S11-N31-Li11	94.7(5)	N24-S14-N14	93.1(7)	O16-Li16-N46	129.0(11)
N31-C91-C121	108.0(9)	N44-S14-Tb(3)	132.6(6)	O16-Li16-O26	92.6(10)
N31-C91-C111	108.3(8)	N34-S14-Tb(3)	130.0(6)	N46-Li16-O26	120.4(11)

C121-C91-C111	108.3(9)	N24-S14-Tb(3)	45.4(4)	O16-Li16-N36	125.5(12)
N31-C91-C101	116.3(8)	N14-S14-Tb(3)	47.7(4)	N46-Li16-N36	71.9(7)
C121-C91-C101	108.0(9)	C14-N14-S14	121.3(9)	O26-Li16-N36	120.8(12)
C111-C91-C101	107.8(9)	C14-N14-Tb(3)	136.7(9)	C206-O16-C176	102.1(14)
C131-N41-S11	129.7(7)	S14-N14-Tb(3)	101.9(6)	C206-O16-Li16	126.0(14)
C131-N41-Li11	135.4(7)	N14-C14-C44	115.7(13)	C176-O16-Li16	131.8(14)
S11-N41-Li11	94.8(5)	N14-C14-C24	110.8(14)	O16-C176-C186	100.4(15)
N41-C131-C151	108.2(10)	C44-C14-C24	107.7(14)	C176-C186-C196	98.5(13)
N41-C131-C141	114.4(9)	N14-C14-C34	108.7(14)	C206-C196-C186	104.8(12)
C151-C131-C141	108.9(10)	C44-C14-C34	106.9(14)	O16-C206-C196	103.7(14)
N41-C131-C161	107.3(9)	C24-C14-C34	106.7(14)	C246-O26-C216	108.5(14)
C151-C131-C161	108.7(9)	C54-N24-S14	128.1(9)	C246-O26-Li16	125.1(14)
C141-C131-C161	109.3(10)	C54-N24-Tb(3)	127.4(9)	C216-O26-Li16	126.2(14)
O11-Li11-N41	122.0(7)	S14-N24-Tb(3)	104.5(6)	O26-C216-C226	101.8(15)
O11-Li11-O21	94.1(7)	N24-C54-C64	109.4(13)	C216-C226-C236	102.2(14)
N41-Li11-O21	130.5(8)	N24-C54-C84	113.4(13)	C246-C236-C226	101.5(12)
O11-Li11-N31	123.5(7)	C64-C54-C84	108.0(13)	O26-C246-C236	108.5(13)
N41-Li11-N31	72.1(6)	N24-C54-C74	109.6(14)		

Magnetic Measurements

The magnetic samples of [{(thf)$_2$Li(N*t*Bu)$_2$S(*t*BuN)$_2$LnCl$_2$}$_2$•ClLi(thf)$_2$] **1a-e**, where M = Gd, Tb, Dy, Ho, and Er, and species [{(thf)$_2$Li(N*t*Bu)$_2$S(*t*BuN)$_2$LnCl$_2$(thf)$_2$] **2a-e** (**a**: Ln = Gd, **b**: Tb, **c**: Dy, **d**: Ho, **e**: Er) were prepared by loading crushed crystalline samples into tubes. Sufficient liquid eicosane (at 60 °C) was added to saturate and cover the samples to prevent crystallite torqueing and provide good thermal contact between the sample and the bath. Tubes were sealed air-tight before transferred to the magnetometer. Magnetic susceptibility measurements were collected using a Quantum Design MPMSXL SQUID magnetometer and Quantum Design MPMS3 SQUID magnetometer, respectively. DC susceptibility data measurements were performed at temperatures ranging from 2 to 300 K for **1a-1e** and **2b-2e**, using applied fields of 0.1, 0.5 and 1 T. Ac magnetic susceptibility data measurements were performed using a 3.6 (MPMSXL) and 4 Oe (MPMS3) switching field, respectively. All data were corrected for diamagnetic contributions from the eicosane and core diamagnetism estimated using Pascal's constants.[2] Cole-Cole plots were fitted using formulae describing χ' and χ'' in terms of frequency, constant temperature susceptibility (χ_T), adiabatic susceptibility (χ_S), relaxation time (τ), and a variable representing the distribution of relaxation times (α).[3]

The sweep rate at zero field can have a rather substantial impact on whether or not a drop in magnetization due to fast magnetic relaxation processes is observed. Therefore, the average sweep rate reported for the hysteresis loops is determined from the data points collected in the +1 to -1 T and -1 to +1 T regions of each loop. Note that this sweep rate is only approximate and may have a large error, since the MPMS-XL does not provide rigorous control over field sweep rate.

Arrhenius Plot Fitting Details.

The temperature dependence of the magnetic relaxation times recorded on sample of **1c** and **2b**, were analyzed in terms of the contributions of different relaxation processes to the observed relaxation rates. Herein we give the details related to the fitting and interpretation of the results, though the reader is referred to the main text for a broader discussion. The curvature of the plots suggested the occurrence of multiple relaxation processes, thus many fits were tried with different incorporated relaxation pathways depending on the sample measurement conditions.

The typical magnetic relaxation pathways discussed in the literature, along with their dependence on temperature, are the following: a temperature independent quantum tunneling pathway, Direct relaxation ($\propto T$ or T^2), the Raman relaxation process ($\propto T^n$, n = 4, 5, 7, or 9 typically), and the Orbach process ($\propto \exp(U_{eff}/k_BT)$).[4,5] Note that successful modeling of the Arrhenius plots for **1c** did not require each term. For example, the data collected on all compounds, at zero dc field were not modeled with the Direct process since the corresponding contribution is nullified in the absence of a dc field. The inclusion of a Direct process did not improve the quality of the fit for **1c**.

Fitting details for compound 1c

The Arrhenius data for **1c** composed from relaxation time data obtained from ac measurements at zero dc field, was modeled using equation (1) and the resulting best-fit parameters are given in Table S26.

$$\frac{1}{\tau_{obs}} = \frac{1}{\tau_{QTM}} + CT^n + \tau_0^{-1} exp\left({}^{-U_{eff}}\big/_{k_B T}\right) \qquad (1)$$

Here, the first term is from the tunneling pathway, the second is for the Raman process, and the third term models Orbach relaxation pathway (see Figure 6 (top) and Figure S53).

The Arrhenius data for **1c** composed from relaxation time data obtained from ac measurements at zero dc field, was modeled using equation (2) and the resulting best-fit parameters are given in Table S27.

$$\frac{1}{\tau_{obs}} = \frac{1}{\tau_{QTM}} + CT^n \qquad (2)$$

Here, the first term is from the tunneling pathway and the second is for the Raman process (see Figures S54-55).

The Arrhenius data for **1c** composed from relaxation time data obtained from ac measurements at 500 Oe dc field, was modeled using equation (3) and the resulting best-fit parameters are given in Table S29.

$$\frac{1}{\tau_{obs}} = CT^n + \tau_0^{-1} exp\left({}^{-U_{eff}}\big/_{\mathrm{k_B}T} \right) \qquad (3)$$

Here, the first term is for the Raman process and the second term models Orbach relaxation pathway (see Figures S56-57). The inclusion of a tunneling term did not improve the quality of the fit for **1c**.

Fitting detail for compound 2b

The Arrhenius data for **2b** composed from relaxation time data obtained from ac measurements, was modeled using equation (4) and the resulting best-fit parameters are given in Table S31.

$$\frac{1}{\tau_{obs}} = AT + CT^n + \tau_0^{-1} exp\left({}^{-U_{eff}}\big/_{\mathrm{k_B}T} \right) \qquad (4)$$

Here, the first term is from the direct process, the second is for the Raman process, and the third term models the Orbach relaxation pathways (Figure 6 (bottom) and Figure S75). The inclusion of a Direct process improved the quality of the fit for **2b**.

Table S26. Best-fit parameters for the Arrhenius plot of **1c** from 2 to 13 K. Equation 1 was used.

	τ_{QTM} (s)	C (s^{-1}K^{-n})	n	τ_0 (s)	U_{eff} (cm^{-1})
**1c** (H_{dc} = 0 Oe)	1.63(1)	0.0017(1)	5.89(1)	5.7(1) × 10^{-5}	28.0(2)

Table S27. Best-fit parameters for the Arrhenius plot of **1c** from 2 to 13 K. Equation 2 was used

	τ_{QTM} (s)	C (s^{-1}K^{-n})	n	τ_0 (s)	U_{eff} (cm^{-1})
**1c** (H_{dc} = 0 Oe)	1.81(5)	0.002(1)	5.91(2)	-	-

Table S28. Best-fit parameters for the Arrhenius plot of **1c** from 7 to 13 and 15 K, respectively. Equation 3 was used.

	τ_{QTM} (s)	C (s^{-1}K^{-n})	n	τ_0 (s)	U_{eff} (cm^{-1})
**1c** (H_{dc} = 0 Oe)	-	11.4(1)	1.251(7)	1.1(1) × 10^{-6}	44.8(1)
**1c** (H_{dc} = 250 Oe)	-	1.66(1)	2.474(1)	4.7(1) × 10^{-7}	54.4(1)

Table S29. Best-fit parameters for the Arrhenius plot of **1c** from 2 to 12 K. Equation 3 was used.

	τ_{QTM} (s)	C (s^{-1}K^{-n})	n	τ_0 (s)	U_{eff} (cm^{-1})
**1c** (H_{dc} = 500 Oe)	-	0.001(1)	6.272(1)	5.2(8) × 10^{-7}	64.9(1)

Table S30. Best-fit parameters for the Arrhenius plot of **1c** from 2 to 12 K. Data was fit to only a Raman Process, CT^n.

	τ_{QTM} (s)	C (s^{-1}K^{-n})	n	τ_0 (s)	U_{eff} (cm^{-1})
**1c** (H_{dc} = 500 Oe)	-	0.00087(8)	6.364(1)	-	-

Table S31. Best-fit parameters for the Arrhenius plot of **2b** from 2 to 9 K. Equation 4 was used.

	A (K^{-1}s^{-1})	C (s^{-1}K^{-n})	n	τ_0 (s)	U_{eff} (cm^{-1})
**2b** (H_{dc} = 2000 Oe)	31.6(7)	68.21(2)	1.686(7)	2.2(4) × 10^{-6}	42.8(6)

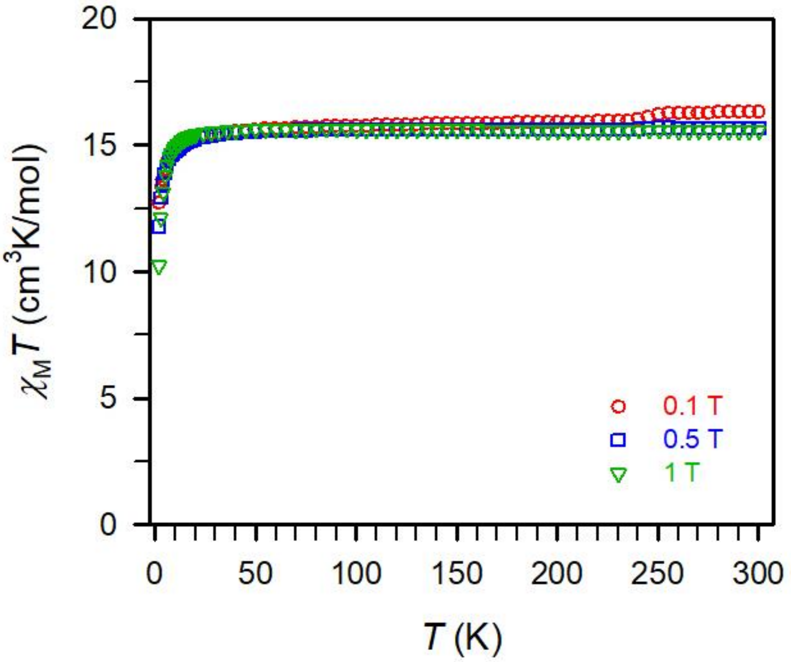

Figure S12. Variable-temperature dc magnetic susceptibility data for a restrained polycrystalline sample of **1a** collected under a 0.1 T (red circles), 0.5 T (blue squares), 1 T (green triangles) applied dc field.

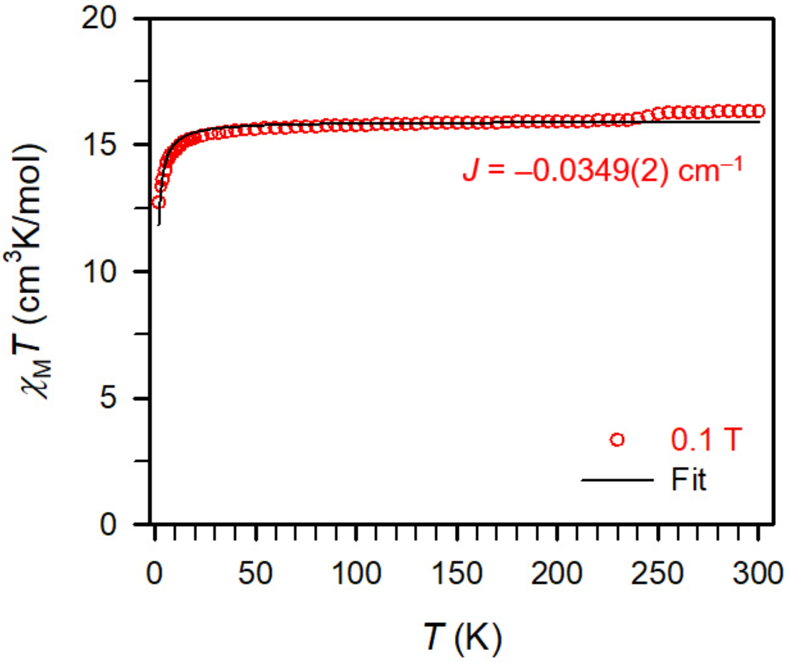

Figure S13. Variable-temperature dc magnetic susceptibility data for a restrained polycrystalline sample of **1a** collected under a 0.1 T applied dc field. The black lines represents a fit to the data for **1a**, as discussed in the main text.

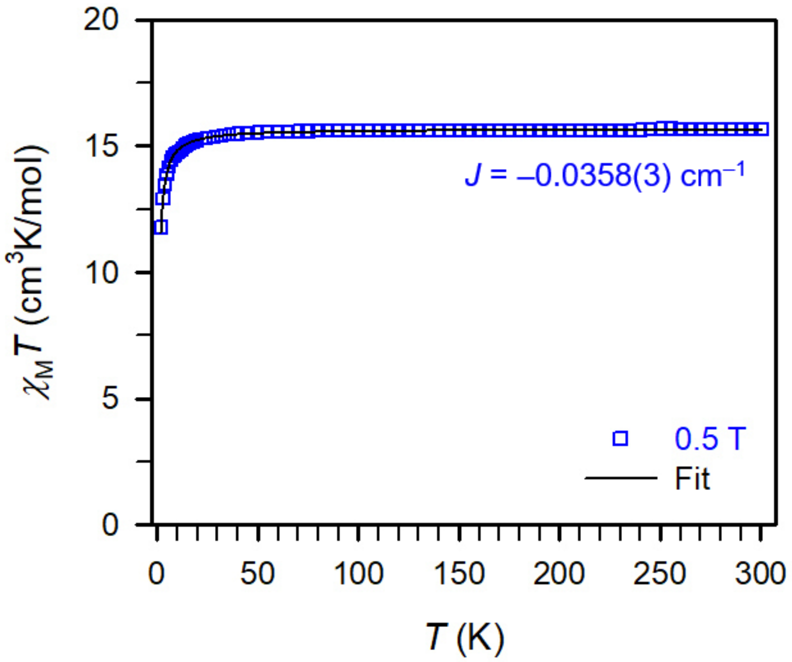

Figure S14. Variable-temperature dc magnetic susceptibility data for a restrained polycrystalline sample of **1a** collected under a 0.5 T applied dc field. The black lines represents a fit to the data for **1a**, as discussed in the main text.

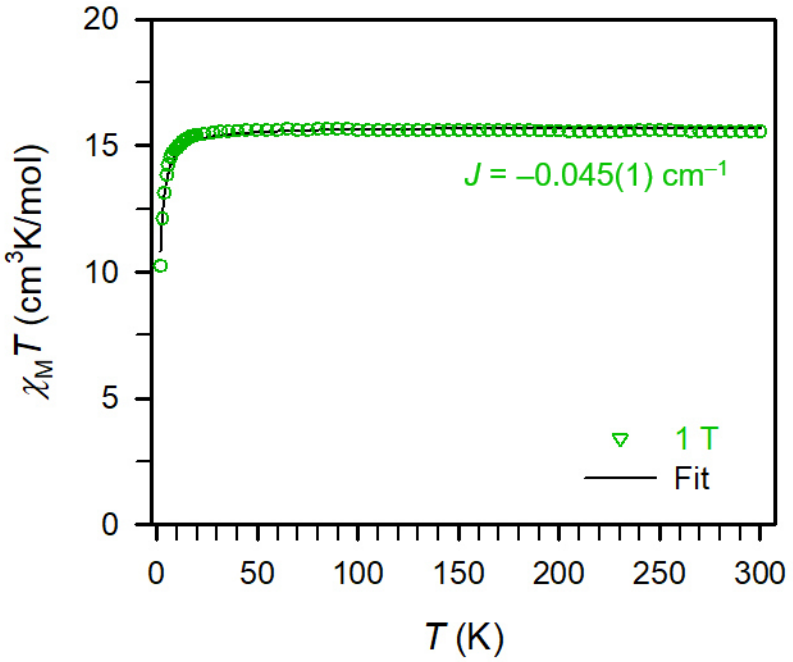

Figure S15. Variable-temperature dc magnetic susceptibility data for a restrained polycrystalline sample of **1a** collected under a 1 T applied dc field. The black lines represents a fit to the data for **1a**, as discussed in the main text.

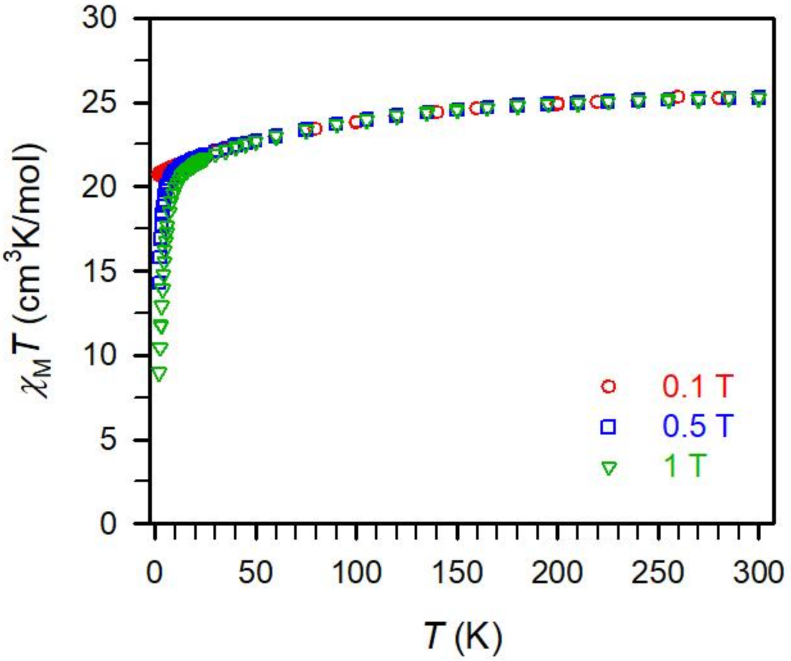

Figure S16. Variable-temperature dc magnetic susceptibility data for restrained polycrystalline samples of **1b** collected under a 0.1 T (red), 0.5 T (blue), and 1 T (green) applied dc field.

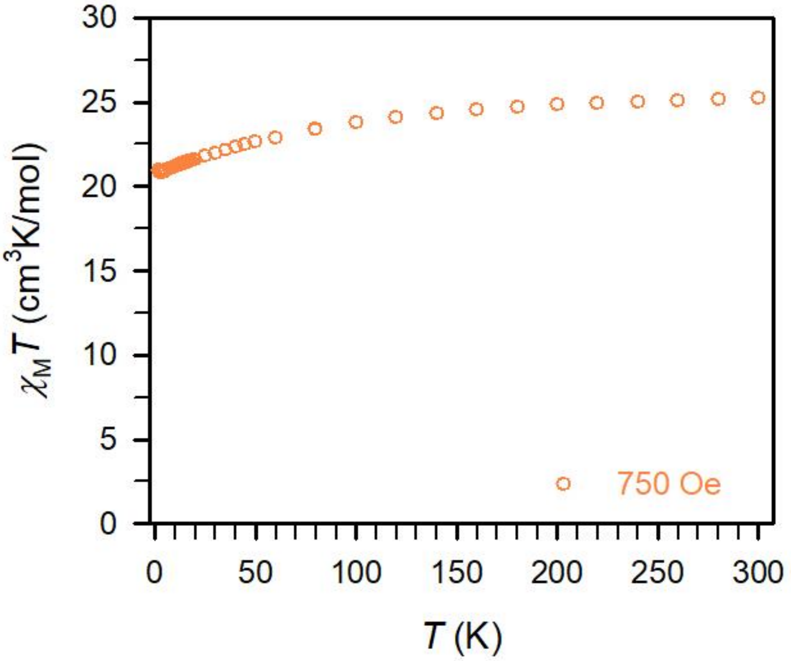

Figure S17. Variable-temperature dc magnetic susceptibility data for a restrained polycrystalline sample of **1b** collected under a 750 Oe applied dc field.

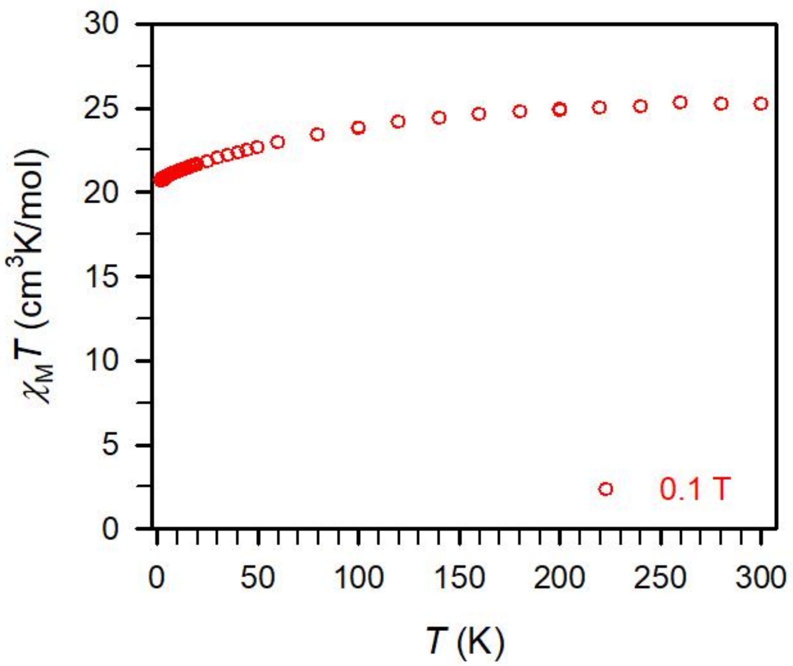

Figure S18. Variable-temperature dc magnetic susceptibility data for a restrained polycrystalline sample of **1b** collected under a 0.1 T applied dc field.

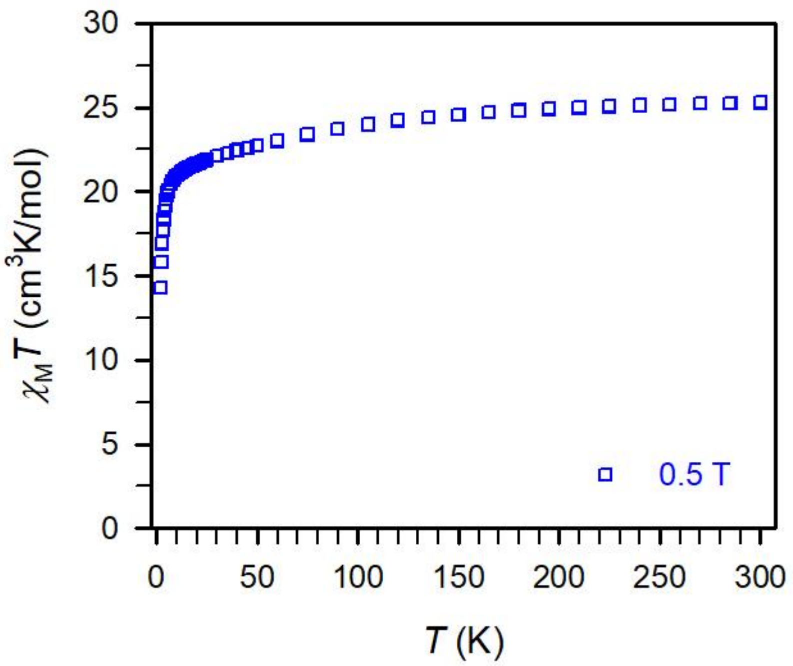

Figure S19. Variable-temperature dc magnetic susceptibility data for a restrained polycrystalline sample of **1b** collected under a 0.5 T applied dc field.

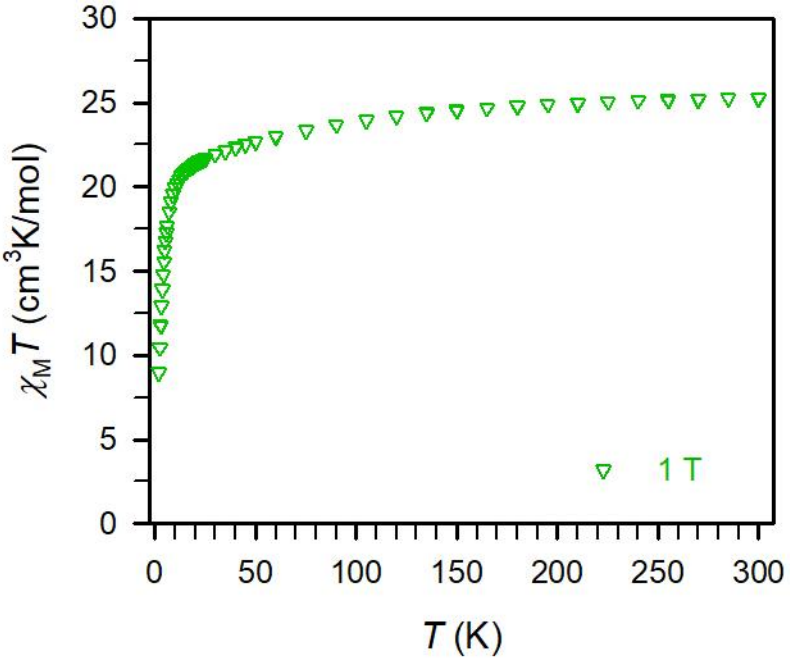

Figure S20. Variable-temperature dc magnetic susceptibility data for a restrained polycrystalline sample of **1b** collected under a 1 T applied dc field.

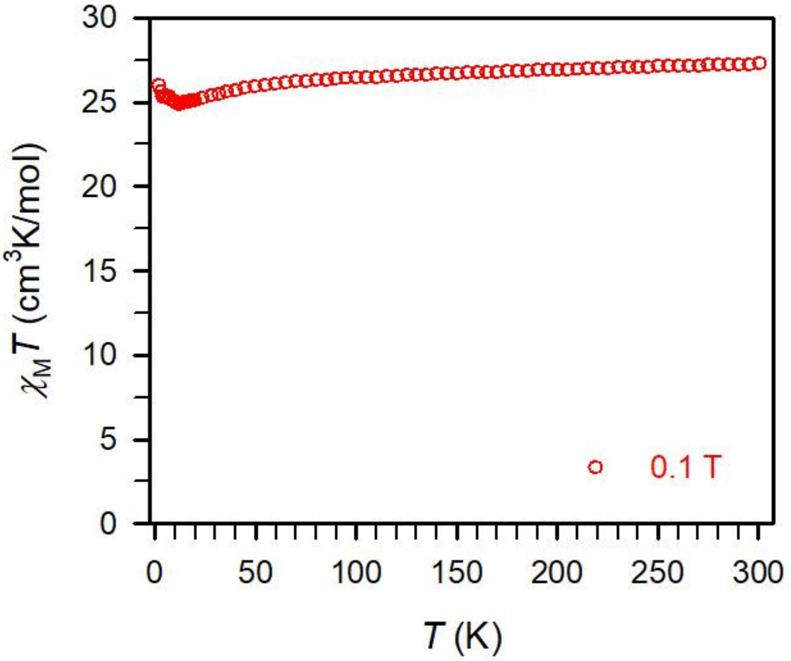

Figure S21. Variable-temperature dc magnetic susceptibility data for restrained polycrystalline samples of **1c** collected under a 0.1 T applied dc field.

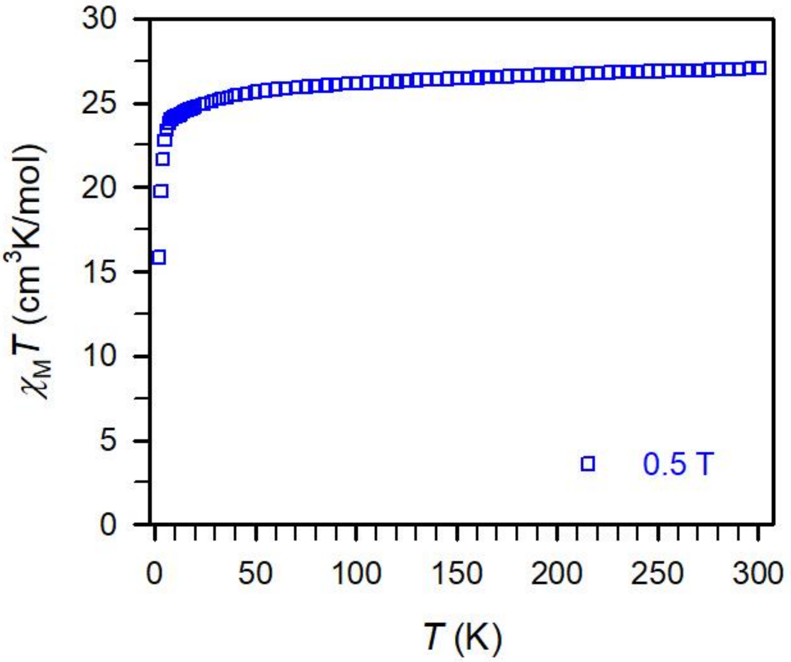

Figure S22. Variable-temperature dc magnetic susceptibility data for restrained polycrystalline samples of **1c** collected under a 0.5 T applied dc field.

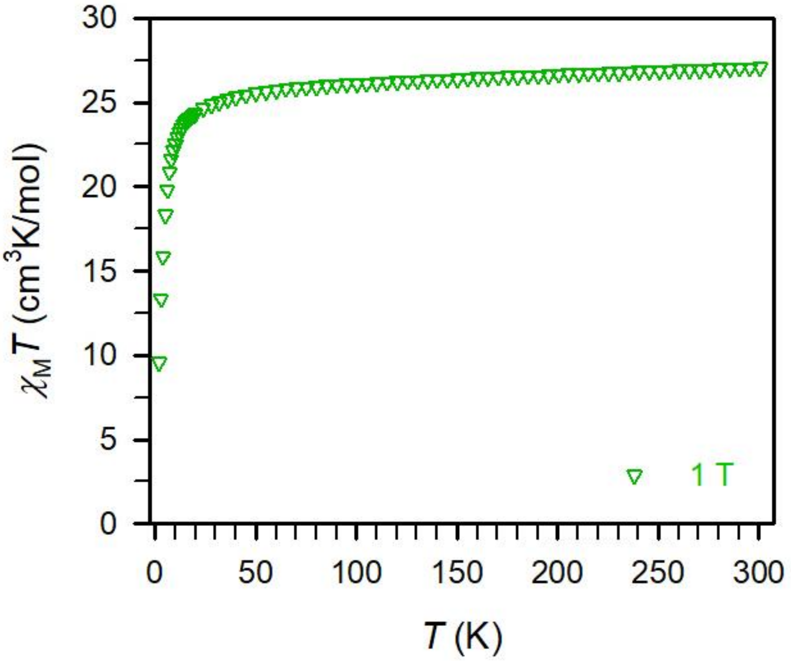

Figure S23. Variable-temperature dc magnetic susceptibility data for restrained polycrystalline samples of **1c** collected under a 1 T applied dc field.

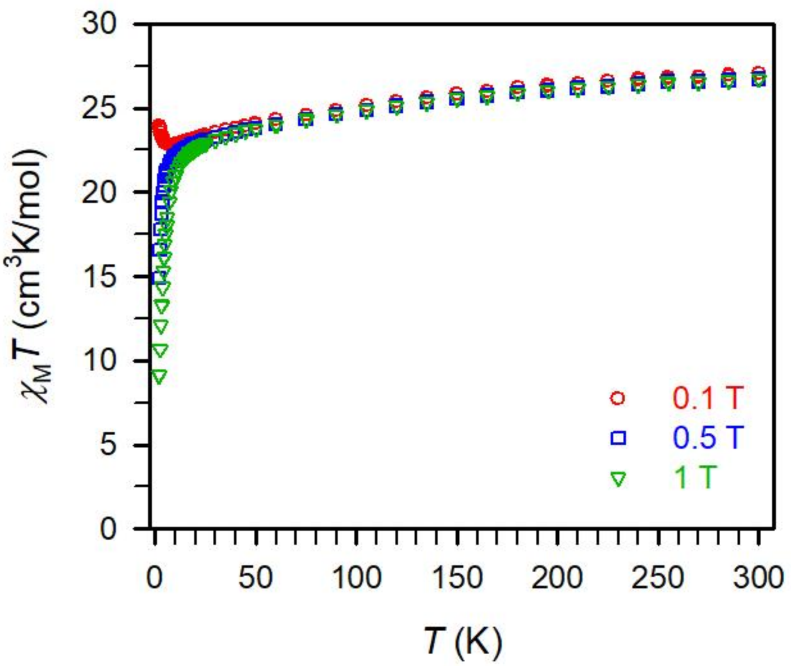

Figure S24. Variable-temperature dc magnetic susceptibility data for restrained polycrystalline samples of **1d** collected under a 0.1 T (red), 0.5 T (blue), and 1 T (green) applied dc field.

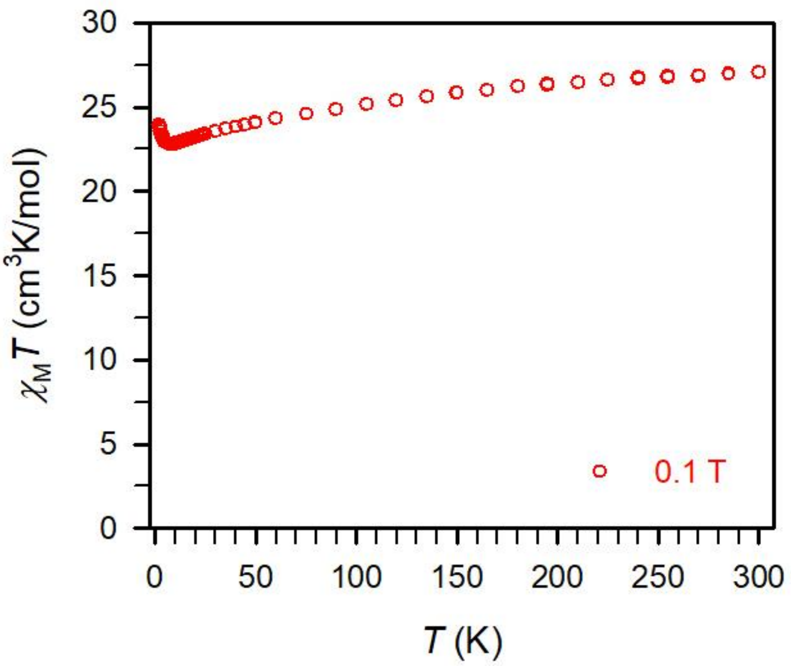

Figure S25. Variable-temperature dc magnetic susceptibility data for restrained polycrystalline samples of **1d** collected under a 0.1 T applied dc field.

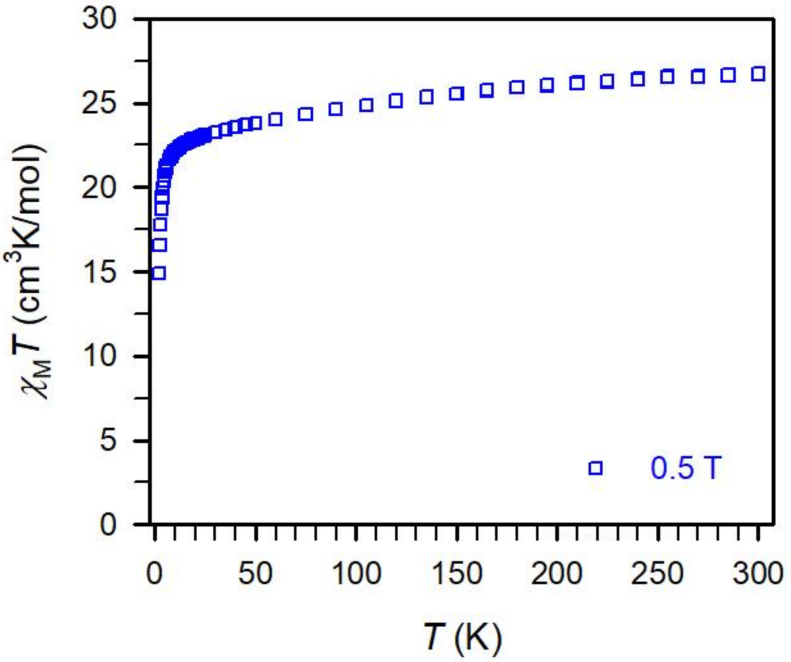

Figure S26. Variable-temperature dc magnetic susceptibility data for restrained polycrystalline samples of **1d** collected under a 0.5 T applied dc field.

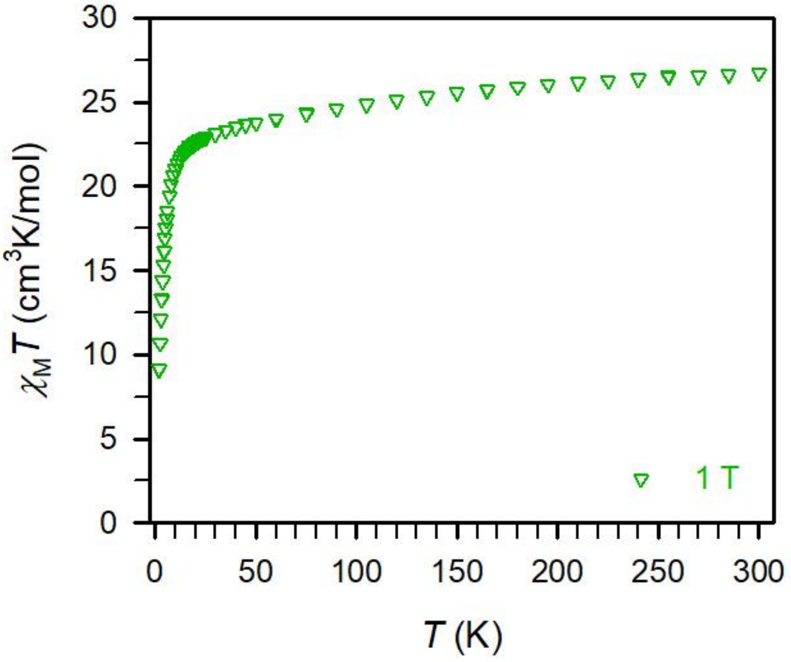

Figure S27. Variable-temperature dc magnetic susceptibility data for restrained polycrystalline samples of **1d** collected under a 1 T applied dc field.

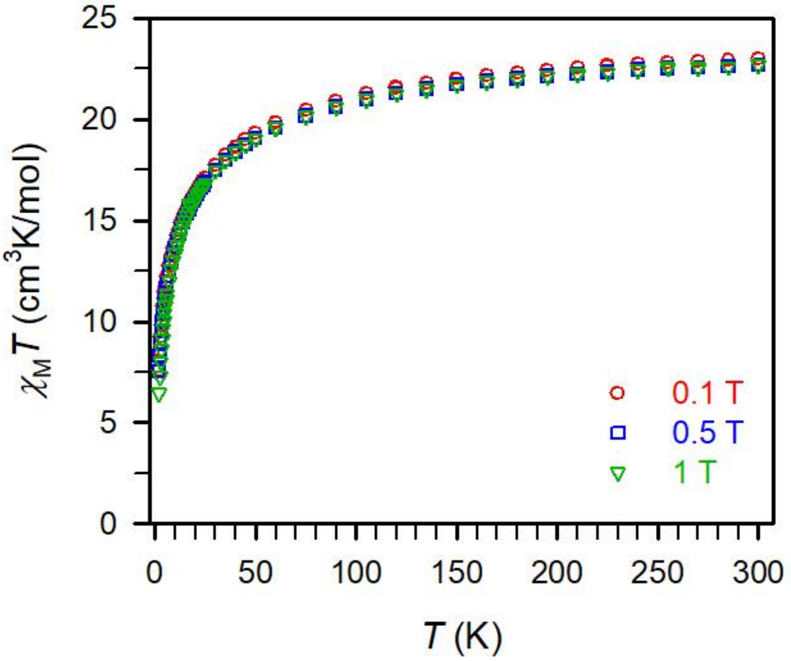

Figure S28. Variable-temperature dc magnetic susceptibility data for restrained polycrystalline samples of **1e** collected under a 0.1 T (red), 0.5 T (blue), and 1 T (green) applied dc field.

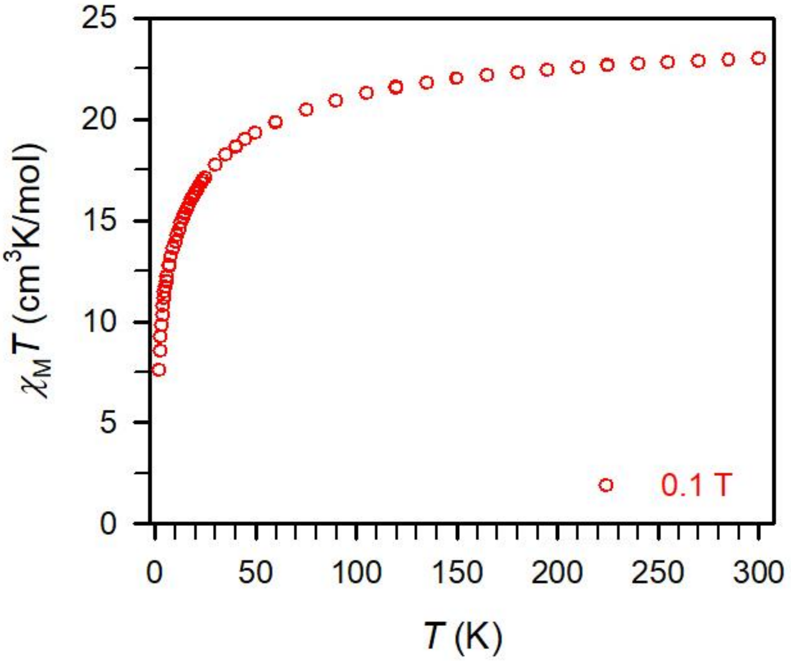

Figure S29. Variable-temperature dc magnetic susceptibility data for restrained polycrystalline samples of **1e** collected under a 0.1 T applied dc field.

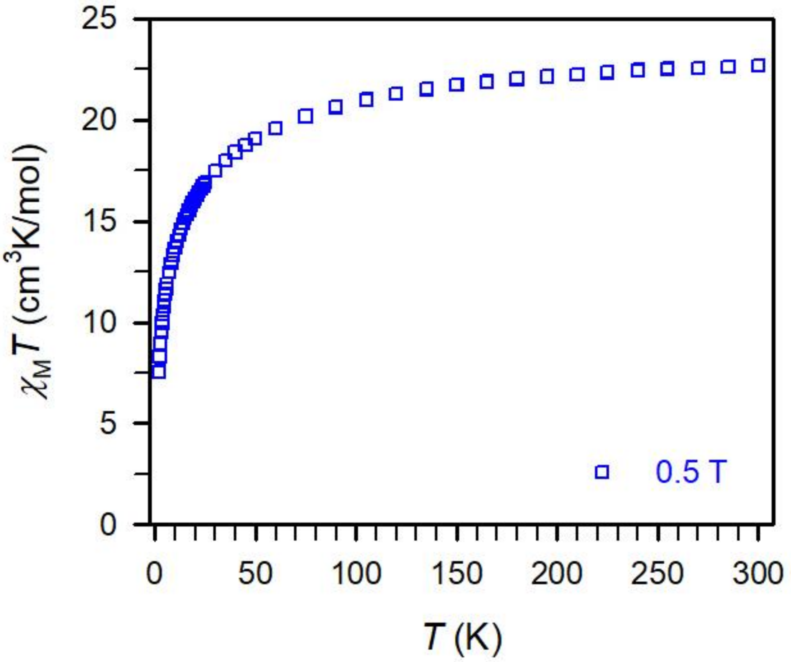

Figure S30. Variable-temperature dc magnetic susceptibility data for restrained polycrystalline samples of **1e** collected under a 0.5 T applied dc field.

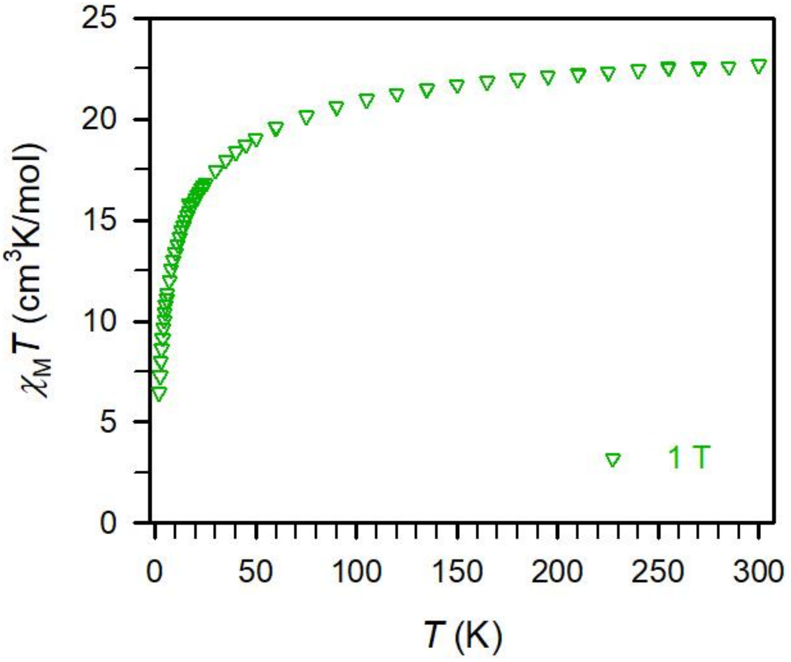

Figure S31. Variable-temperature dc magnetic susceptibility data for restrained polycrystalline samples of **1e** collected under a 1 T applied dc field.

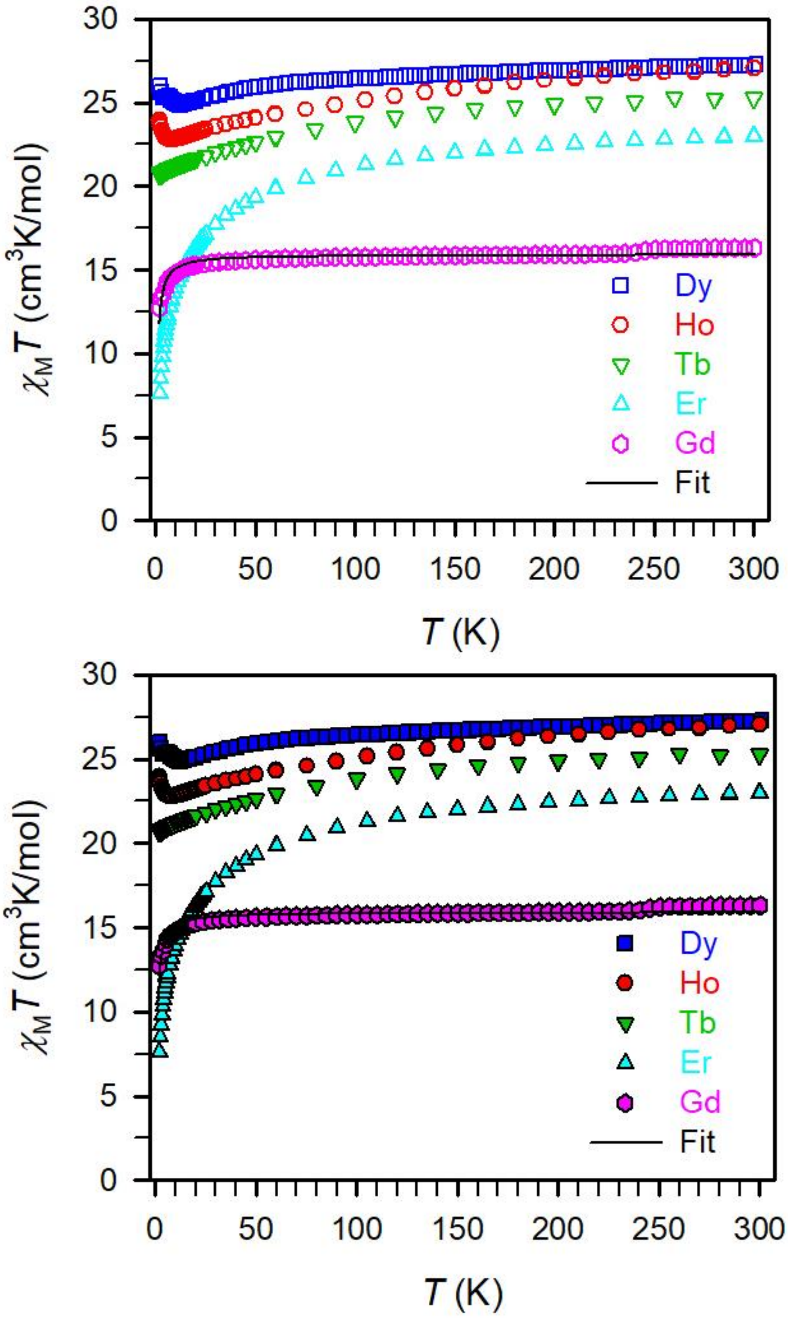

Figure S32. Variable-temperature dc magnetic susceptibility data for restrained polycrystalline samples of **1a** (pink, Gd), **1b** (cyan, Er), **1c** (green, Tb), **1d** (red, Ho), and **1e** (blue, Dy) collected under a 0.1 T applied dc field. The black lines represents a fit to the data for **1a**, as discussed in the main text.

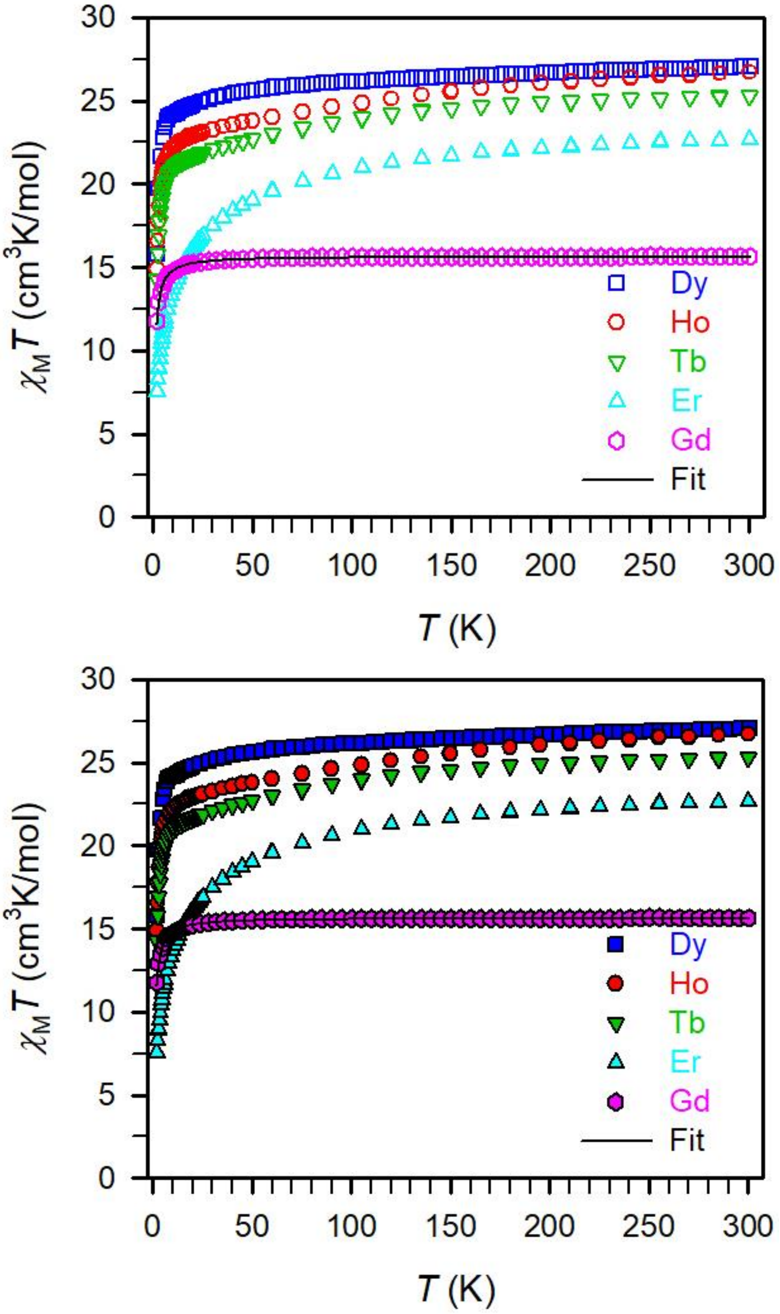

Figure S33. Variable-temperature dc magnetic susceptibility data for restrained polycrystalline samples of **1a** (pink, Gd), **1b** (cyan, Er), **1c** (green, Tb), **1d** (red, Ho), and **1e** (blue, Dy) collected under a 0.5 T applied dc field. The black lines represents a fit to the data for **1a**, as discussed in the main text.

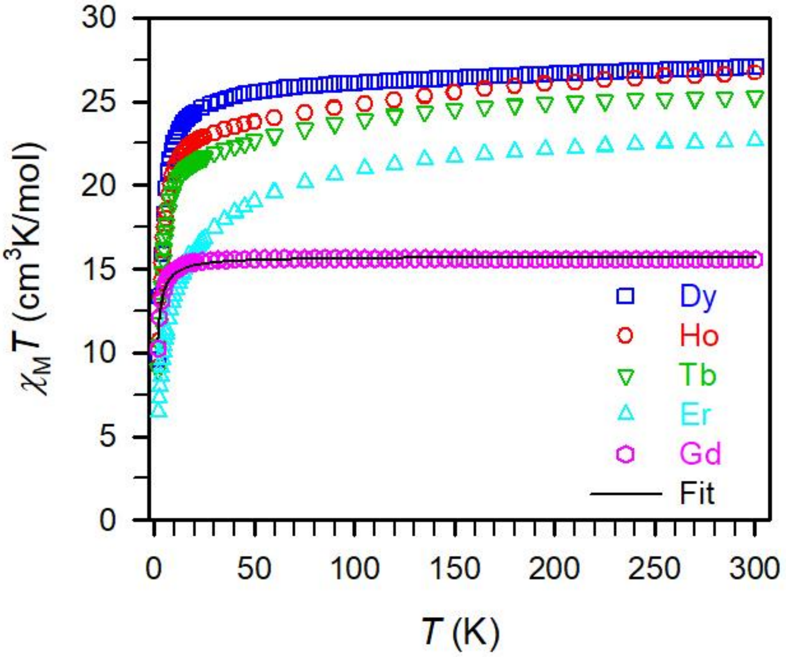

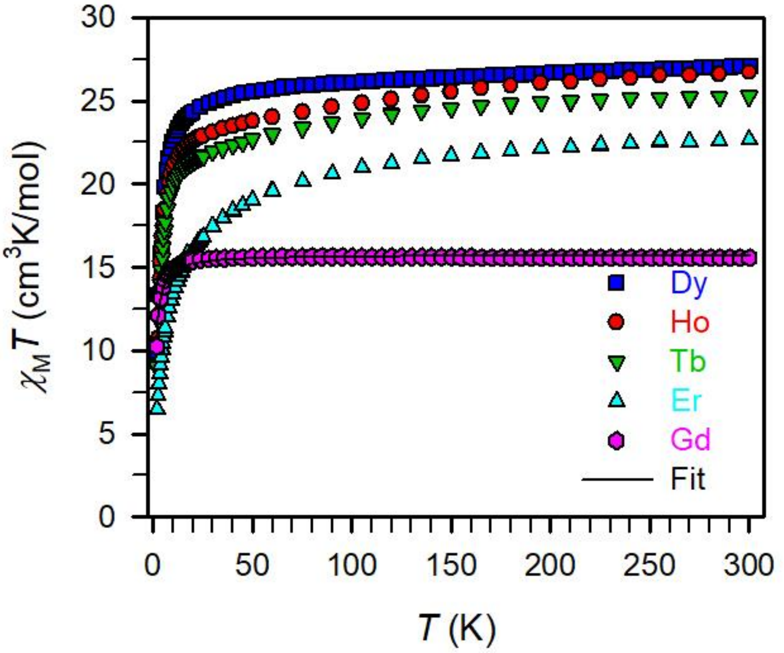

Figure S34. Variable-temperature dc magnetic susceptibility data for restrained polycrystalline samples of **1a** (pink, Gd), **1b** (cyan, Er), **1c** (green, Tb), **1d** (red, Ho), and **1e** (blue, Dy) collected under a 1 T applied dc field. The black lines represents a fit to the data for **1a**, as discussed in the main text.

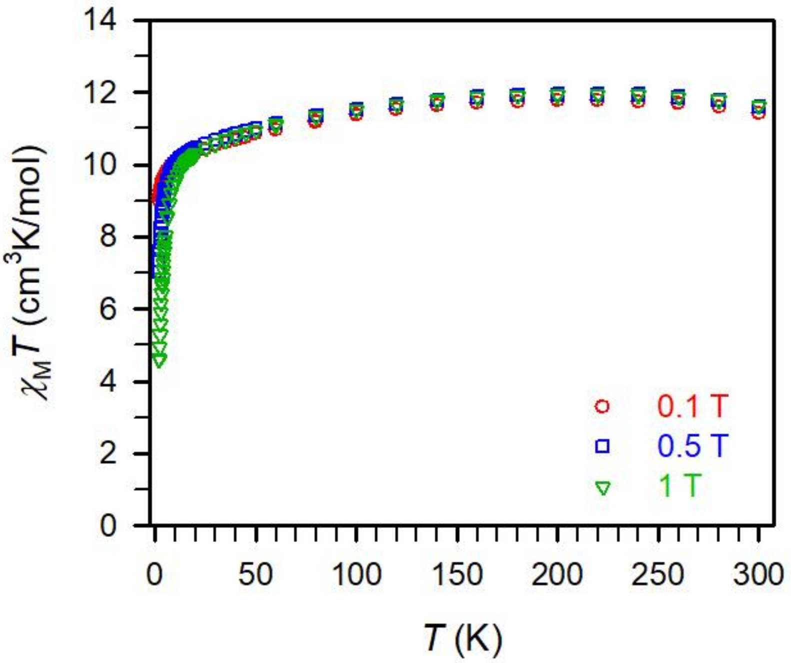

Figure S35. Variable-temperature dc magnetic susceptibility data for restrained polycrystalline samples of **2b** collected under a 0.1 T (red), 0.5 T (blue), and 1 T (green) applied dc field.

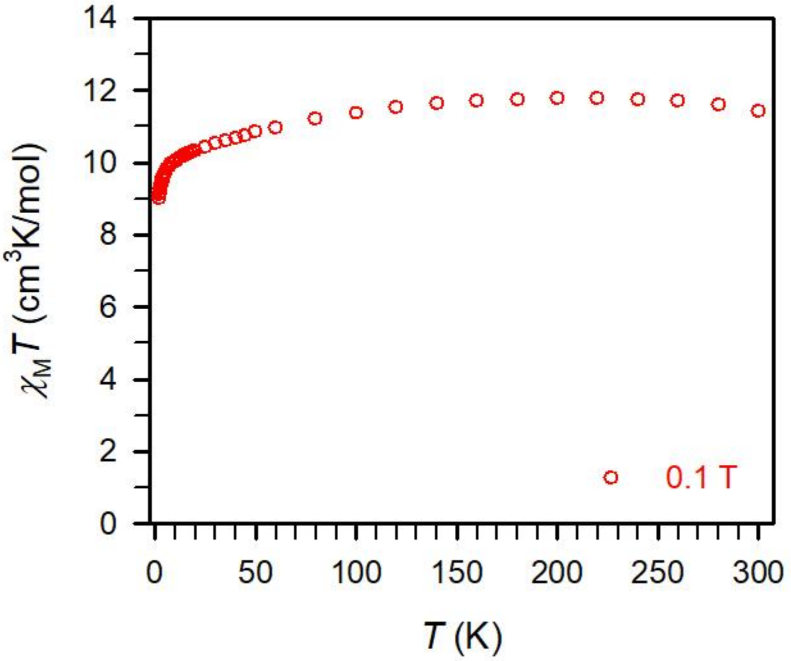

Figure S36. Variable-temperature dc magnetic susceptibility data for restrained polycrystalline samples of **2b** collected under a 0.1 T applied dc field.

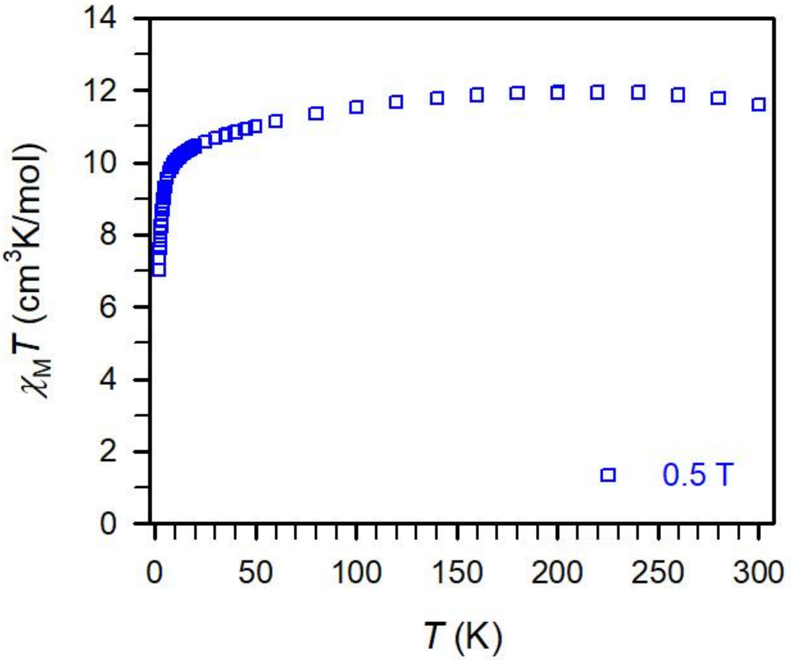

Figure S37. Variable-temperature dc magnetic susceptibility data for restrained polycrystalline samples of **2b** collected under a 0.5 T applied dc field.

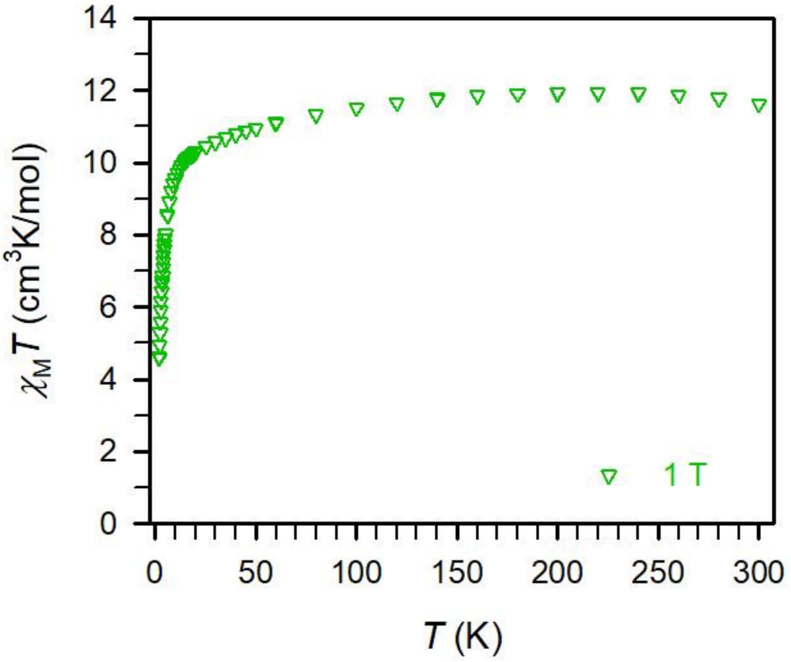

Figure S38. Variable-temperature dc magnetic susceptibility data for restrained polycrystalline samples of **2b** collected under a 1 T applied dc field.

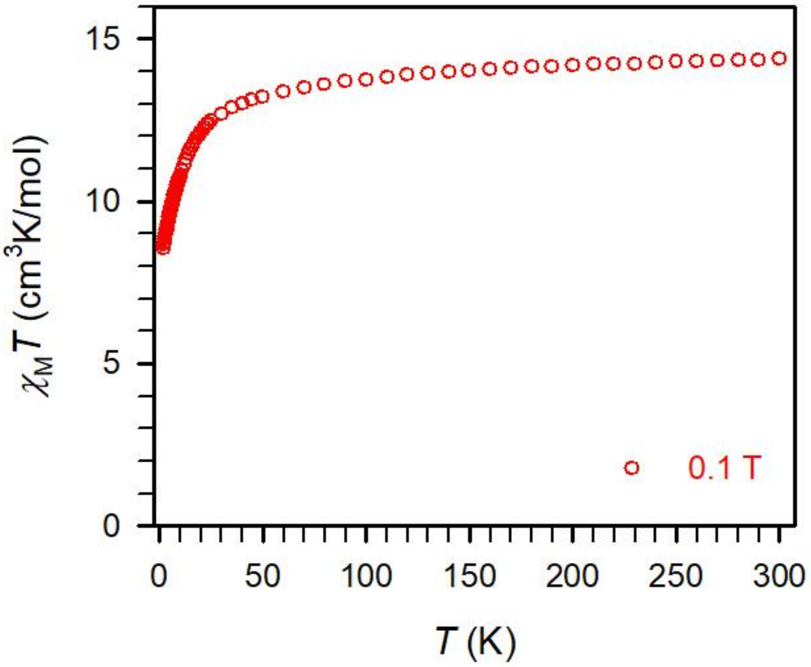

Figure S39. Variable-temperature dc magnetic susceptibility data for restrained polycrystalline samples of **2c** collected under a 0.1 T applied dc field.

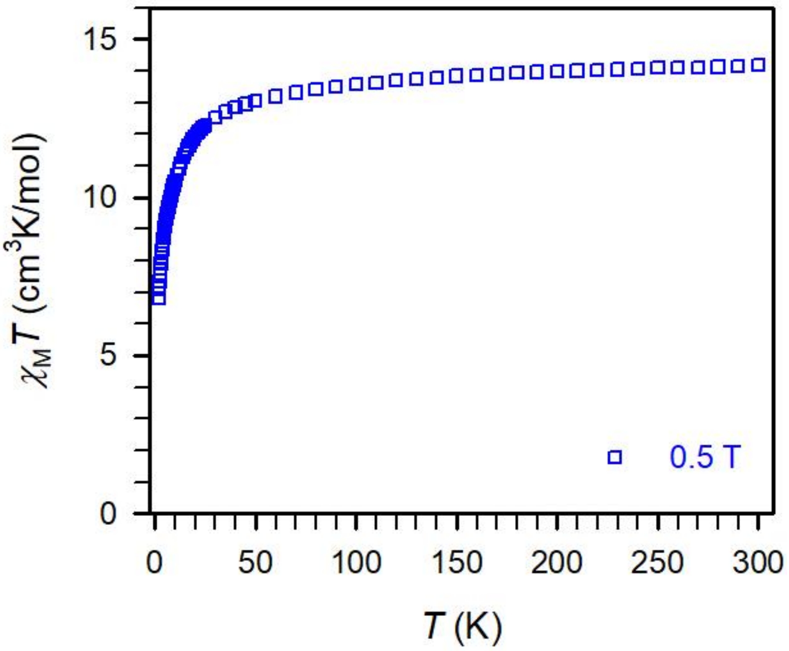

Figure S40. Variable-temperature dc magnetic susceptibility data for restrained polycrystalline samples of **2c** collected under a 0.5 T applied dc field.

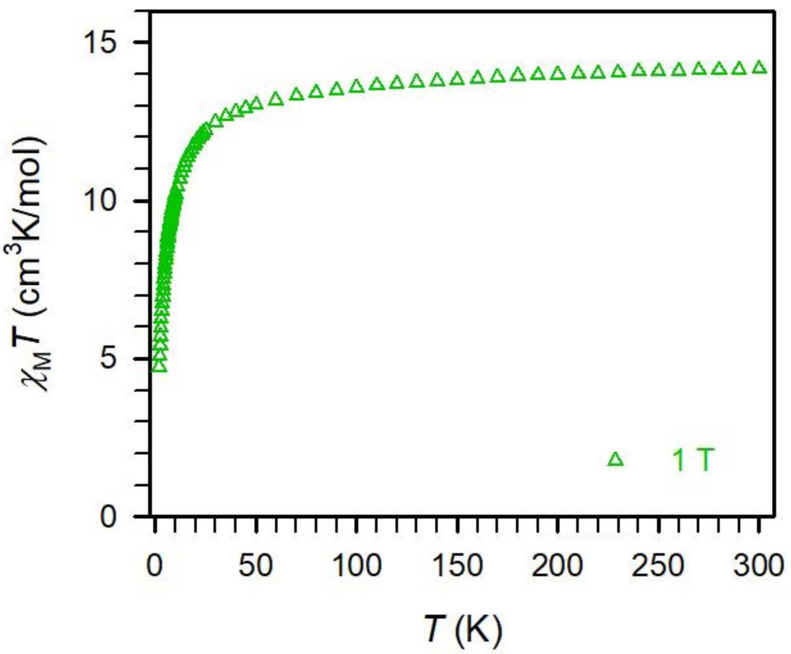

Figure S41. Variable-temperature dc magnetic susceptibility data for restrained polycrystalline samples of **2c** collected under a 1 T applied dc field.

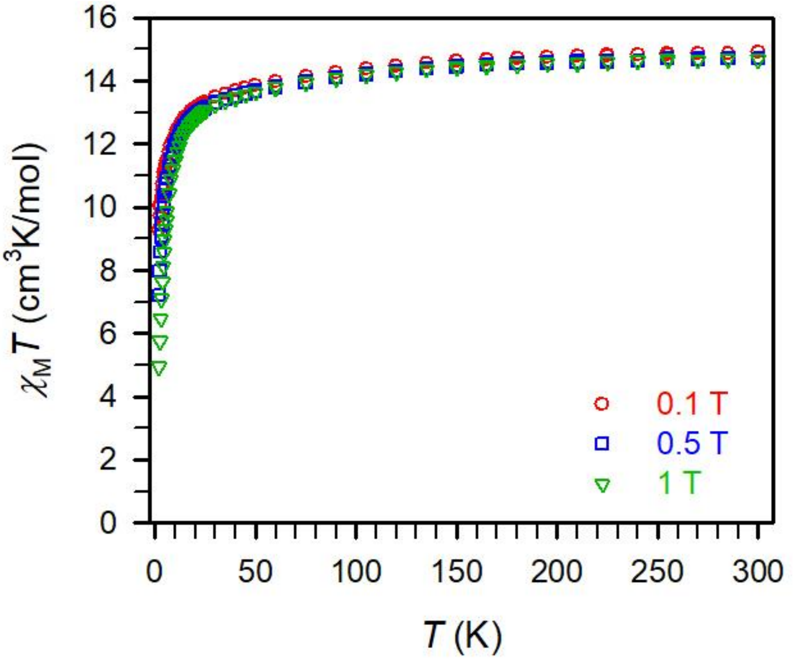

Figure S42. Variable-temperature dc magnetic susceptibility data for restrained polycrystalline samples of **2d** collected under a 0.1 T (red), 0.5 T (blue), and 1 T (green) applied dc field.

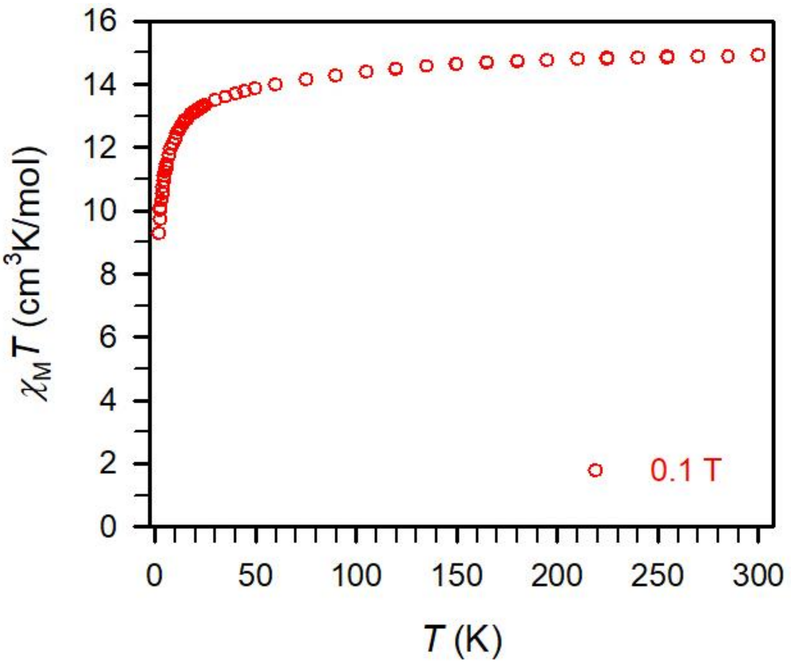

Figure S43. Variable-temperature dc magnetic susceptibility data for restrained polycrystalline samples of **2d** collected under a 0.1 T applied dc field.

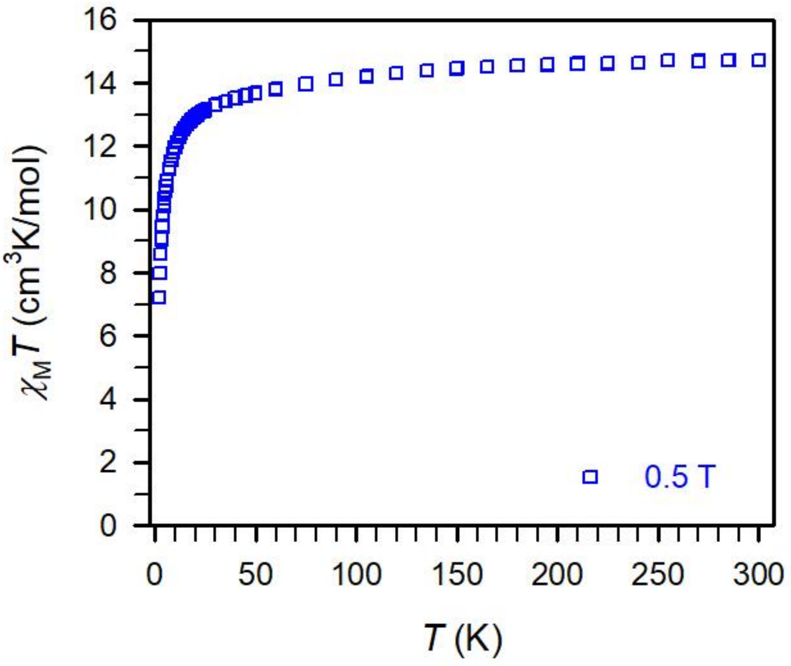

Figure S44. Variable-temperature dc magnetic susceptibility data for restrained polycrystalline samples of **2d** collected under a 0.5 T applied dc field.

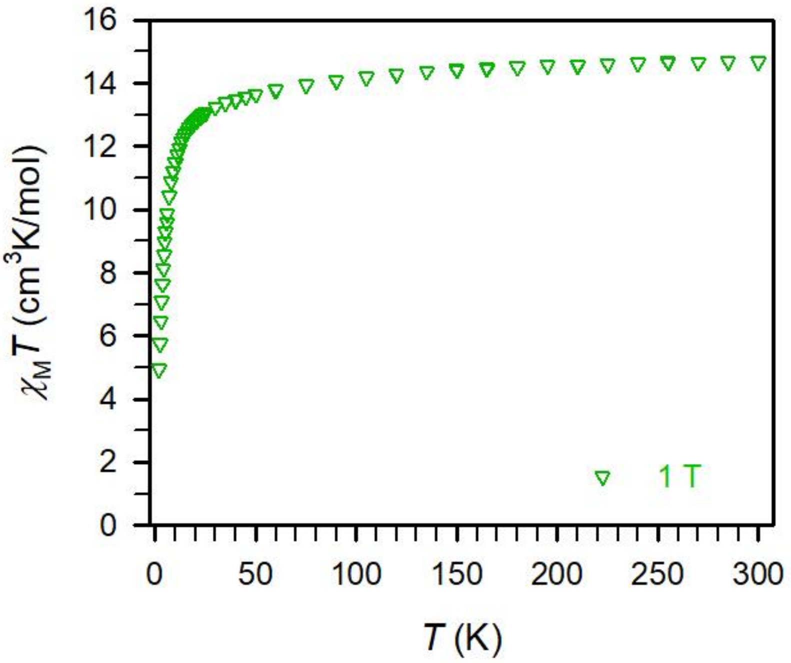

Figure S45. Variable-temperature dc magnetic susceptibility data for restrained polycrystalline samples of **2d** collected under a 1 T applied dc field.

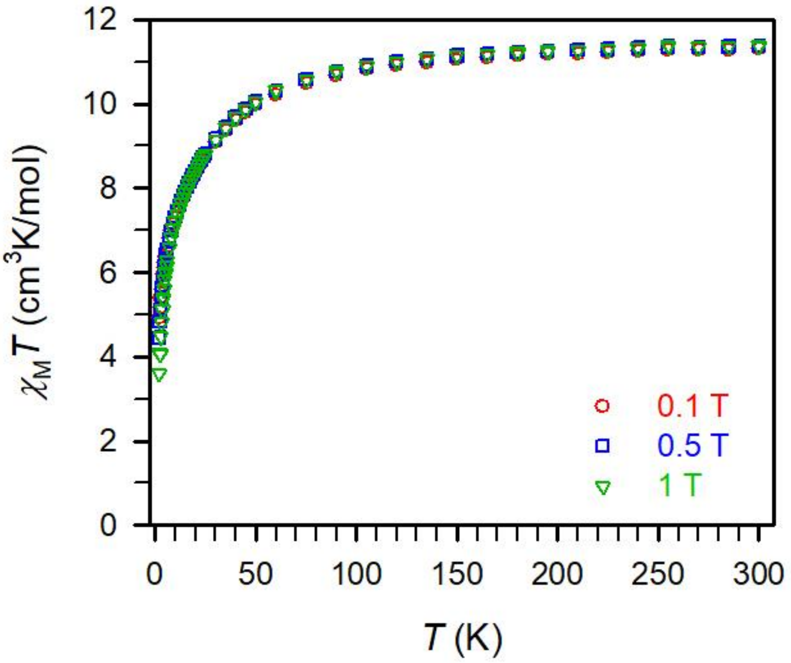

Figure S46. Variable-temperature dc magnetic susceptibility data for restrained polycrystalline samples of **2e** collected under a 0.1 T (red), 0.5 T (blue), and 1 T (green) applied dc field.

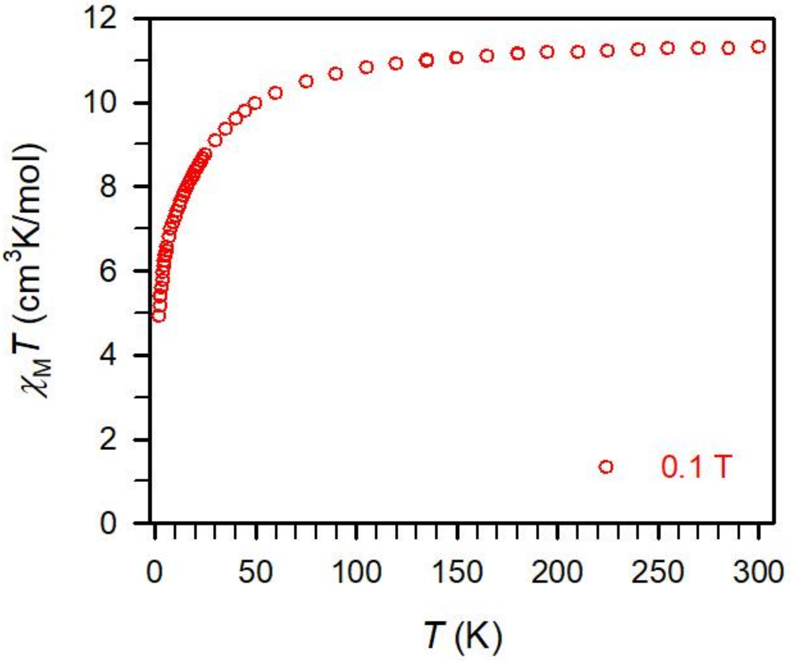

Figure S47. Variable-temperature dc magnetic susceptibility data for restrained polycrystalline samples of **2e** collected under a 0.5 T applied dc field.

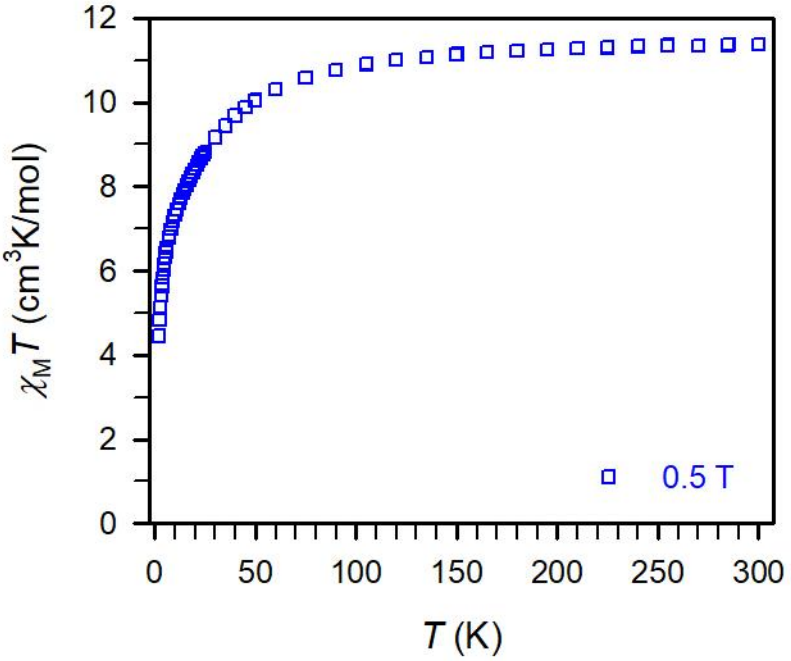

Figure S48. Variable-temperature dc magnetic susceptibility data for restrained polycrystalline samples of **2e** collected under a 0.5 T applied dc field.

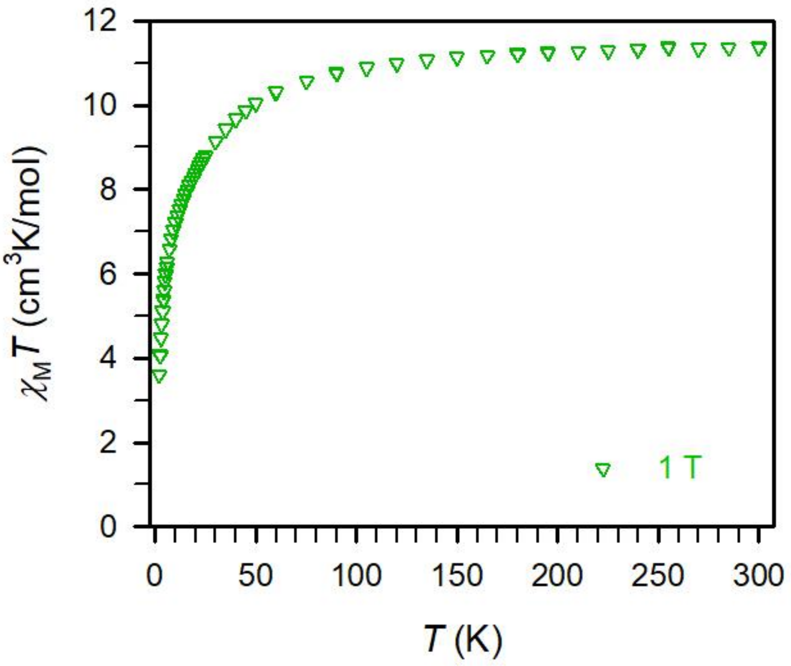

Figure S49. Variable-temperature dc magnetic susceptibility data for restrained polycrystalline samples of **2e** collected under a 1 T applied dc field.

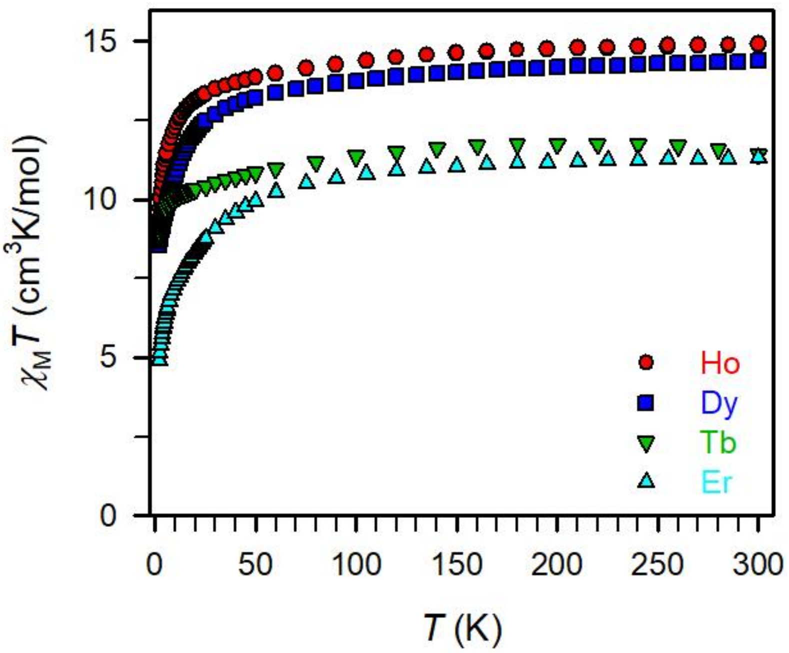

Figure S50. Variable-temperature dc magnetic susceptibility data for restrained polycrystalline samples of **2b** (green, Tb), **2c** (blue, Dy), **2d** (red, Ho), and **2e** (cyan, Er) collected under a 0.1 T applied dc field.

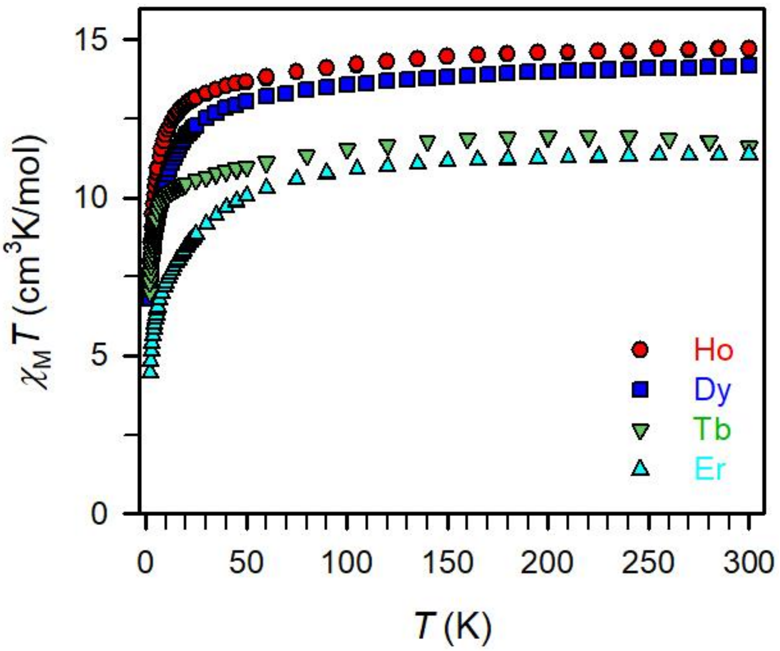

Figure S51. Variable-temperature dc magnetic susceptibility data for restrained polycrystalline samples of **2b** (green, Tb), **2c** (blue, Dy), **2d** (red, Ho), and **2e** (cyan, Er) collected under a 0.5 T applied dc field.

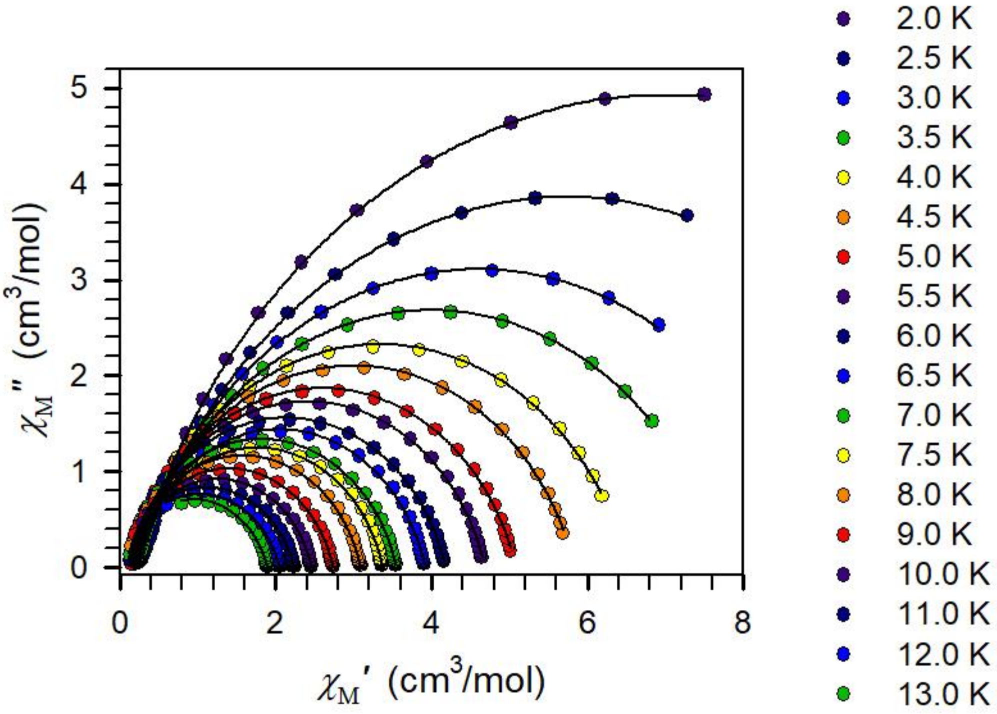

Figure S52. Cole-Cole (Argand) plots for ac susceptibility collected from 2 to 13 K under 0 Oe applied dc field for **1c**. Symbols represent the experimental data points and the points representing the fits are connected by solid black lines.

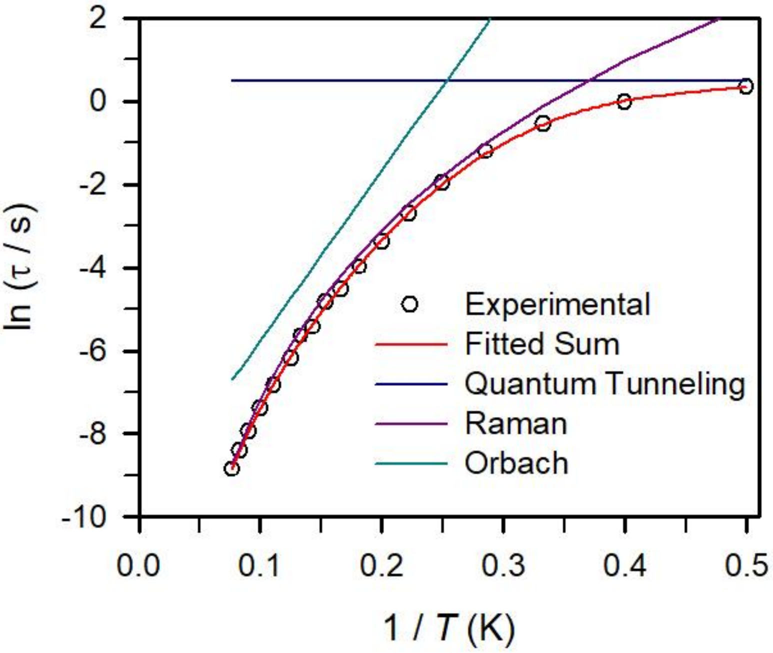

Figure S53. Individual contributions of the multiple magnetic relaxation pathways to the Arrhenius plots of **1c** at 0 Oe. Individual parameters used to calculate the contributions are given in Table S26.

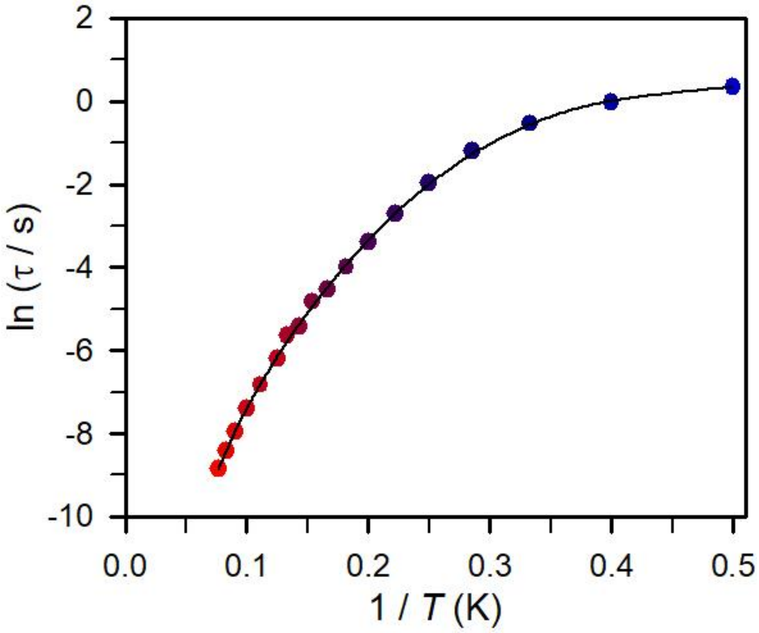

Figure S54. Arrhenius plot of relaxation time data for **1c** at zero dc field from 2 K (blue circles) to 13 K (red circles). Solid line represents a fit to the data (see Figure 55).

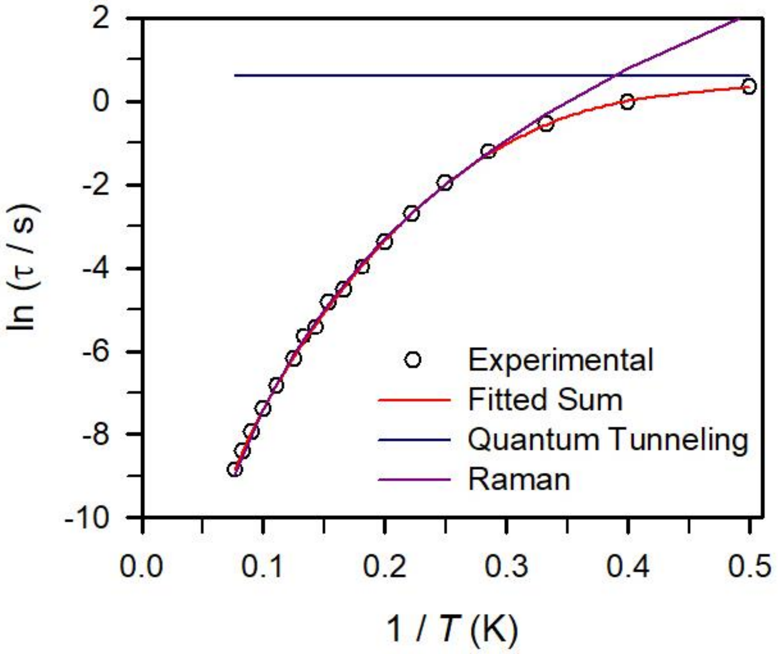

Figure S55. Individual contributions of the multiple magnetic relaxation pathways to the Arrhenius plots of **1c** at 0 Oe. Individual parameters used to calculate the contributions are given in Table S27 (see Figure S54).

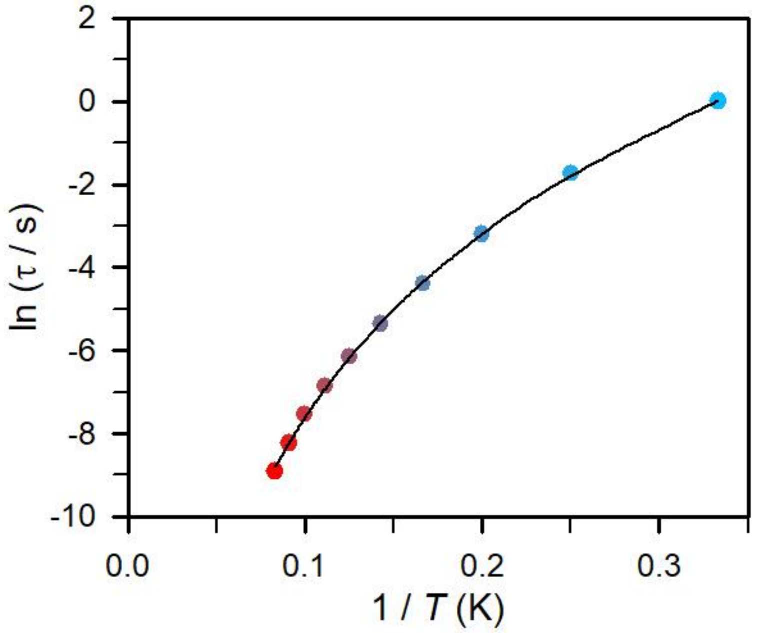

Figure S56. Arrhenius plot of relaxation time data for **1c** at 500 Oe dc field from 3 K (pale blue circles) to 12 K (red circles). Solid line represents a fit to the data (see Figure S47 and Table S29).

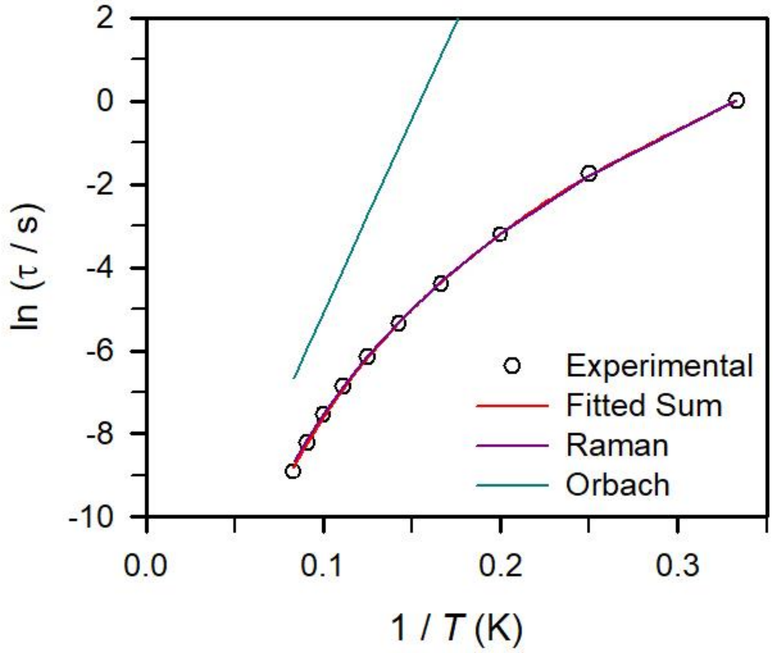

Figure S57. Individual contributions of the multiple magnetic relaxation pathways to the Arrhenius plots of **1c** at 500 Oe. Individual parameters used to calculate the contributions are given in Table S4 (see Figure S56).

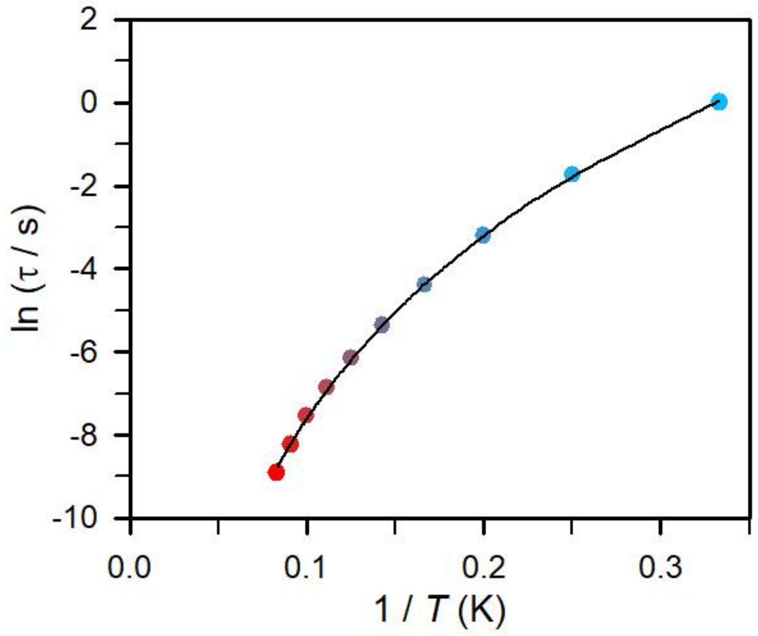

Figure S58. Arrhenius plot of relaxation time data for **1c** at 500 Oe dc field from 3 K (pale blue circles) to 12 K (red circles). Solid lines represent a fit to the data (see Figure S59 and Table S30).

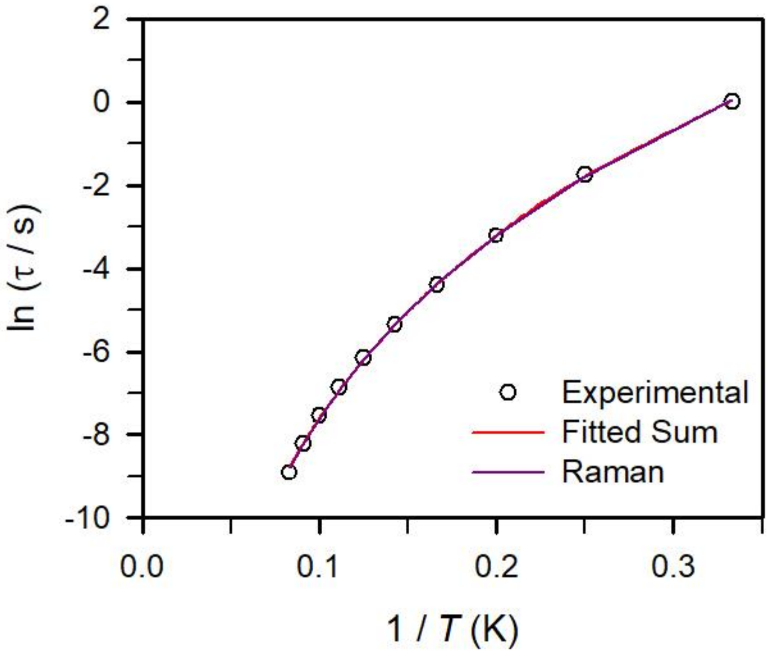

Figure S59. Individual contributions of the multiple magnetic relaxation pathways to the Arrhenius plots of **1c** at 500 Oe. Individual parameters used to calculate the contributions are given in Table S5 (see Figure 58).

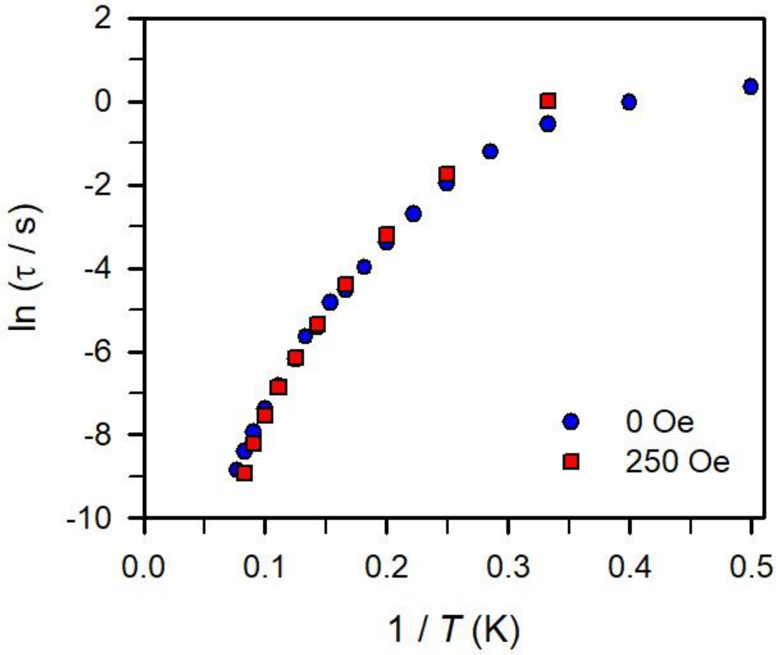

Figure S60. Arrhenius plot of relaxation time data for **1c** at 0 Oe (blue circles) and 500 Oe dc fields (red squares).

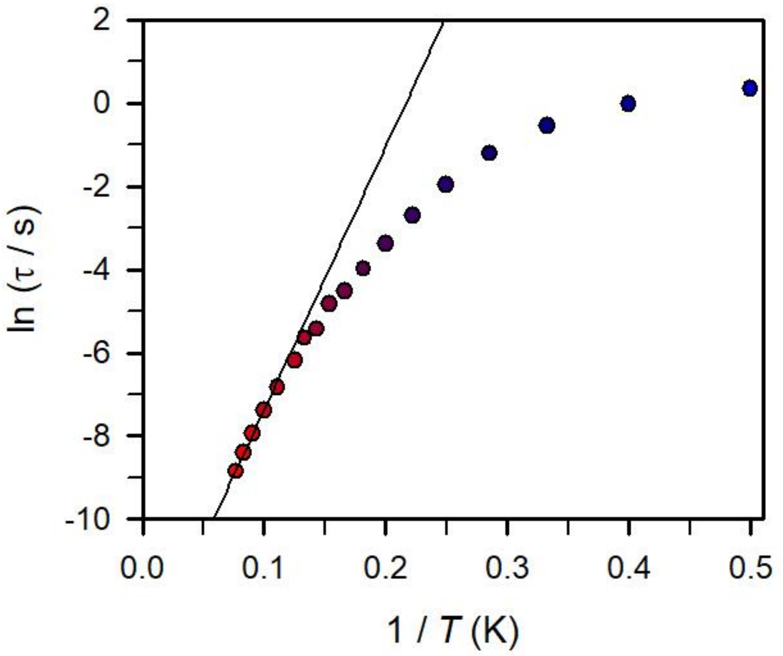

Figure S61. Arrhenius plot of relaxation time data for **1c** at 0 Oe dc field from 2 K (blue circles) to 13 K (red circles). Solid line represents a fit to the highest temperature data collected from 10 to 13 K yielding $U_{\mathrm{eff}} = 43.9(8)\ \mathrm{cm}^{-1}$ and $\tau_0 = 1.1(1)\cdot 10^{-6}$ s.

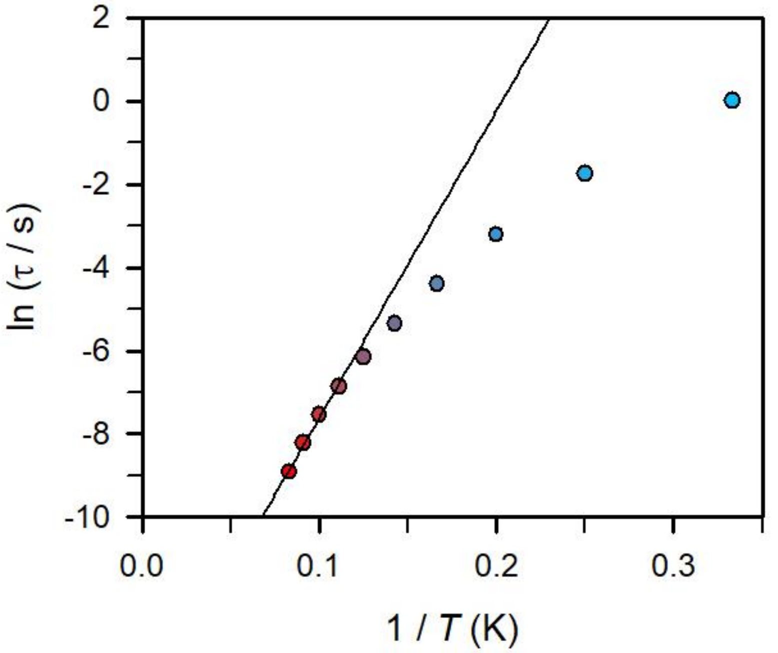

Figure S62. Arrhenius plot of relaxation time data for **1c** at 500 Oe dc field from 3 K (pale blue circles) to 12 K (red circles). Solid line represents a fit to the highest temperature data collected from 9 to 12 K yielding $U_{\mathrm{eff}} = 45.7(3)\ \mathrm{cm}^{-1}$ and $\tau_0 = 6.4(4)\cdot 10^{-7}$ s.

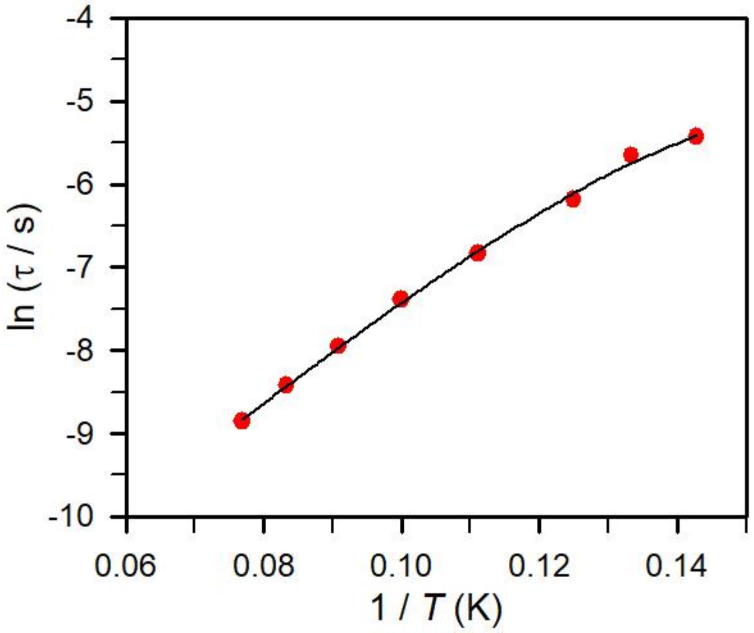

Figure S63. Arrhenius plot of relaxation time data for **1c** at zero dc field from 7 K to 13 K (red circles). Solid line represents a fit to the data (see Figure S64).

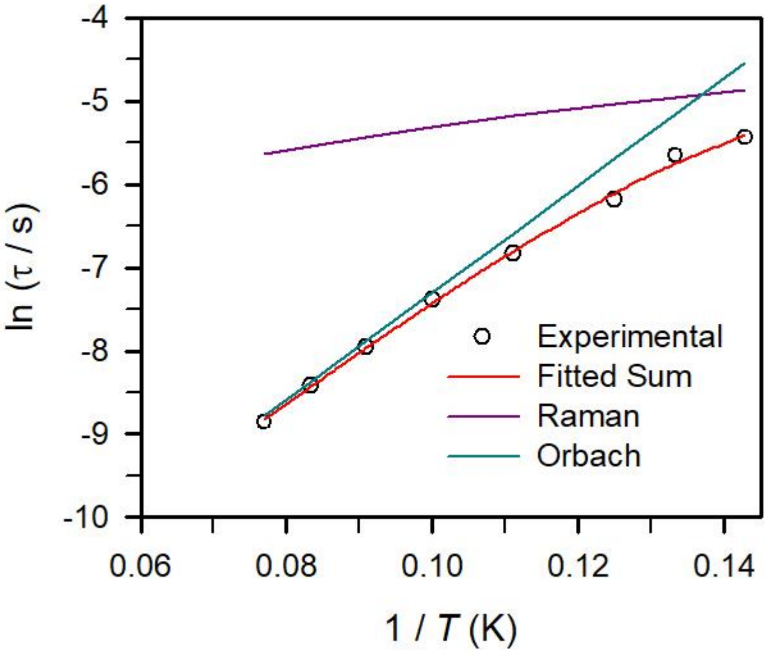

Figure S64. Individual contributions of the multiple magnetic relaxation pathways to the Arrhenius plots of **1c** at 0 Oe from 7 K to 13 K. Individual parameters used to calculate the contributions are given in Table S28 (see Figure S63).

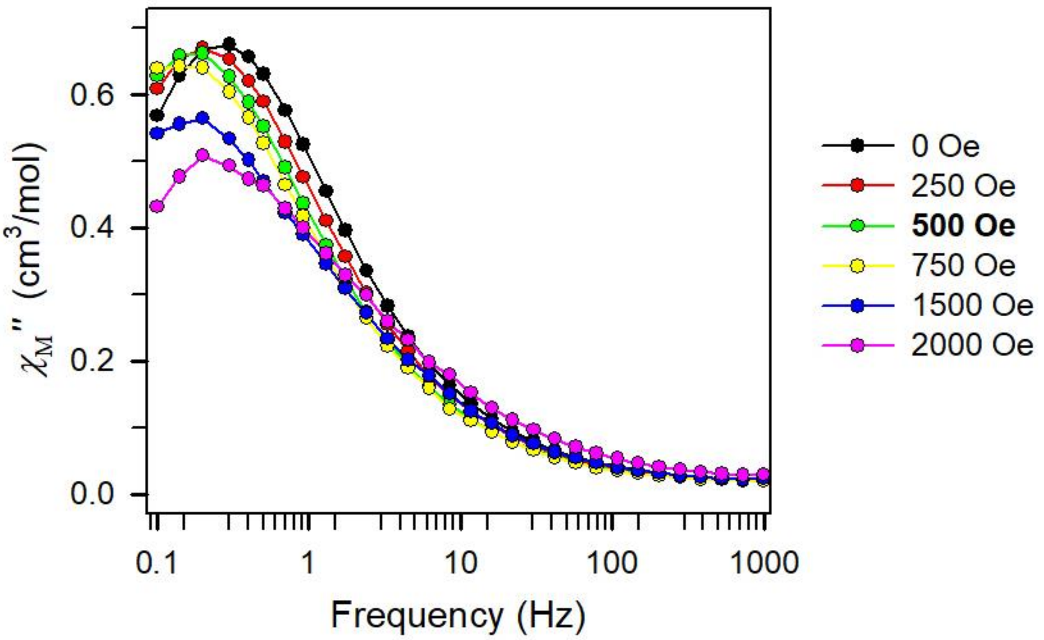

Figure S65. Out-of-phase ac susceptibility (χ_M'') collected on pure **1c** at 3 K under dc fields ranging from 0 Oe to 2000 Oe. Solid lines are guides for the eye.

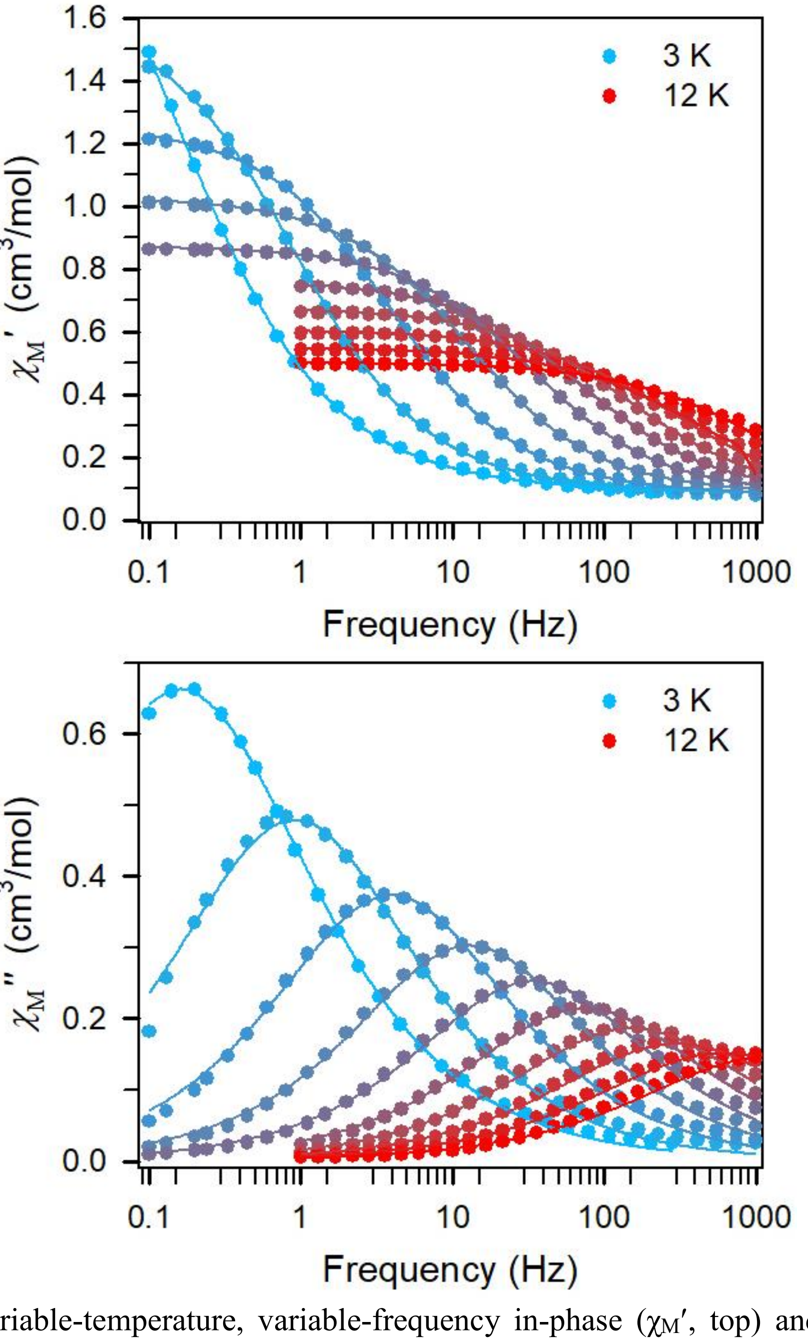

Figure S66. Variable-temperature, variable-frequency in-phase (χ_M', top) and out-of-phase (χ_M'', bottom) ac magnetic susceptibility data collected for **1c** under 500 Oe applied dc field from 2 K (pale blue circles) to 12 K (red circles). Solid lines represent a fit to the data.

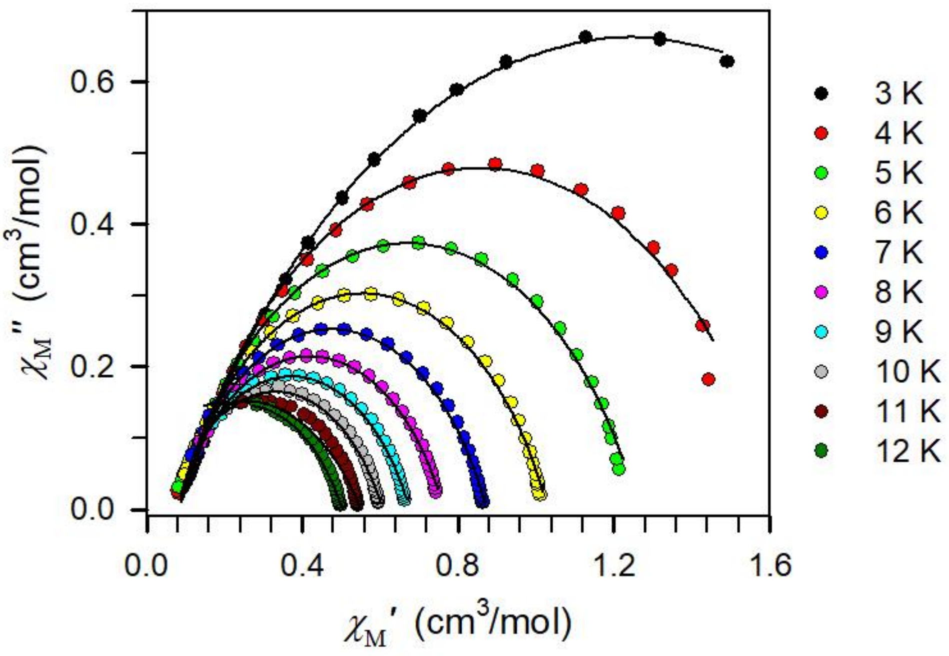

Figure S67. Cole-Cole (Argand) plots for ac susceptibility collected from 3 to 12 K under 500 Oe applied dc field for **1c**. Symbols represent the experimental data points and the points representing the fits are connected by solid black lines.

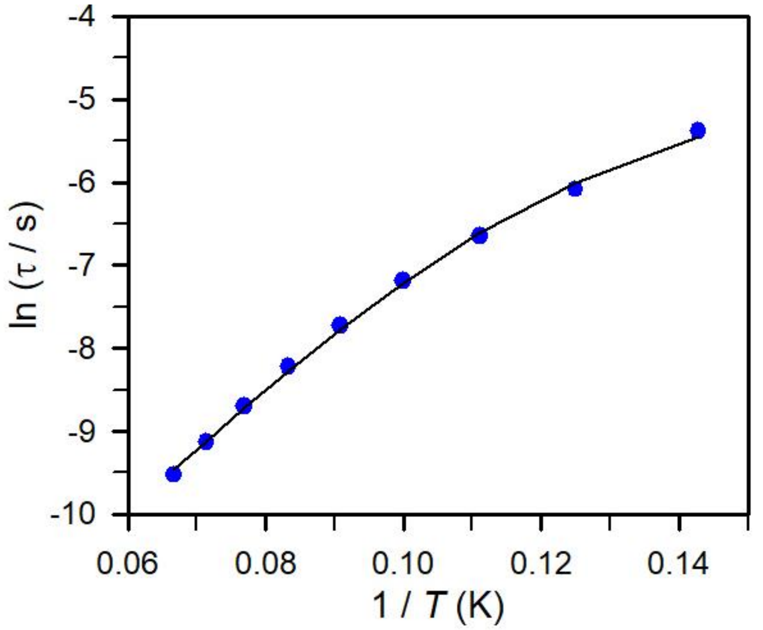

Figure S68. Arrhenius plot of relaxation time data for **1c** at 250 Oe dc field from 7 K to 15 K (blue circles). Solid line represents a fit to the data (see Figure S69).

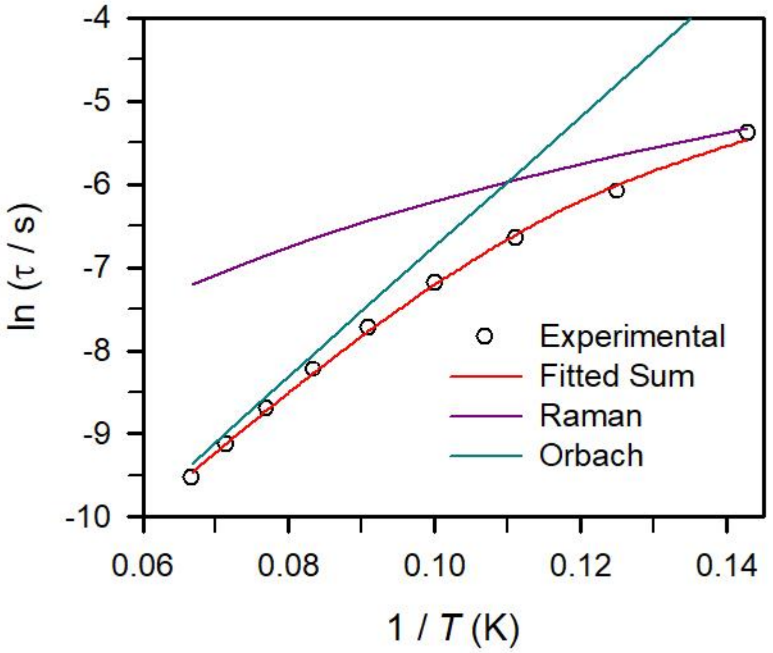

Figure S69. Individual contributions of the multiple magnetic relaxation pathways to the Arrhenius plots of **1c** at 250 Oe from 7 K to 15 K. Individual parameters used to calculate the contributions are given in Table S28 (see Figure S68).

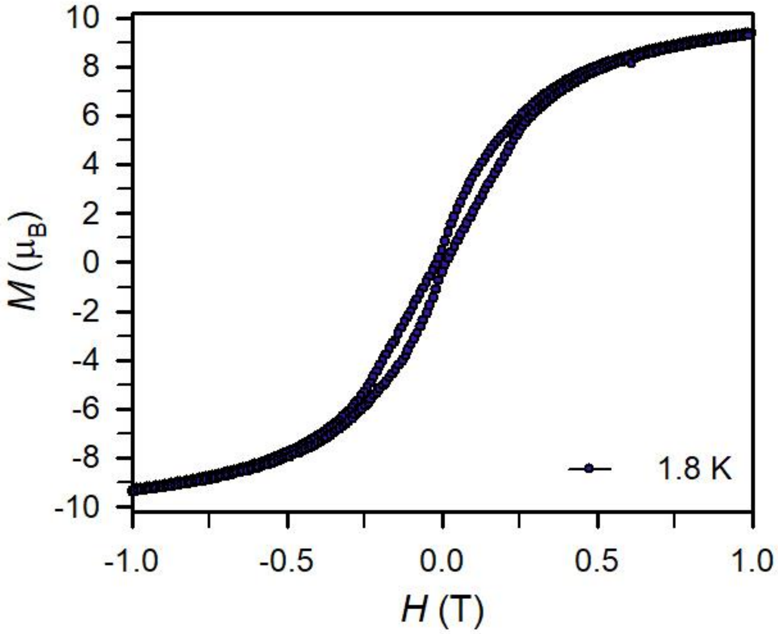

Figure S70. Variable field magnetization (*M*) data for **1c** collected at 1.8 K at an average sweep rate of 0.01 Ts^{-1}.

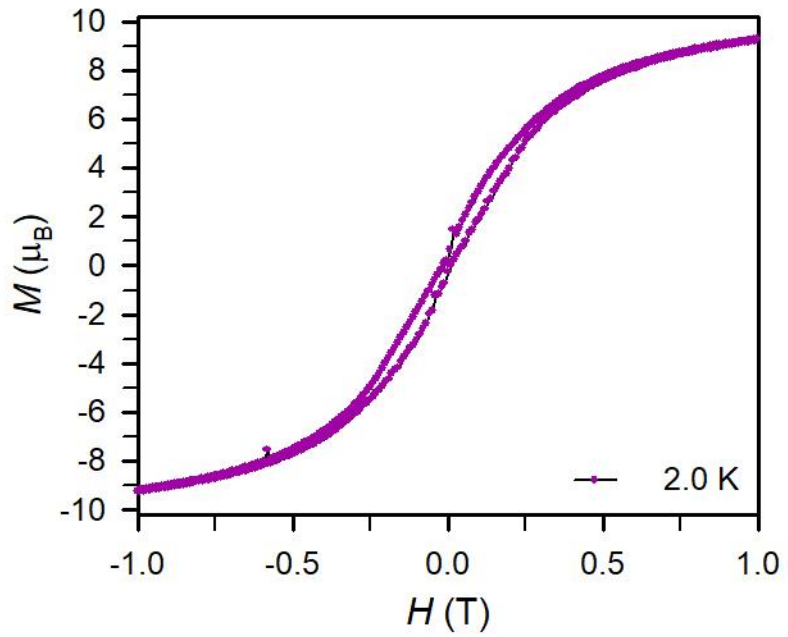

Figure S71. Variable field magnetization (*M*) data for **1c** collected at 2.0 K at an average sweep rate of 0.01 Ts^{-1}.

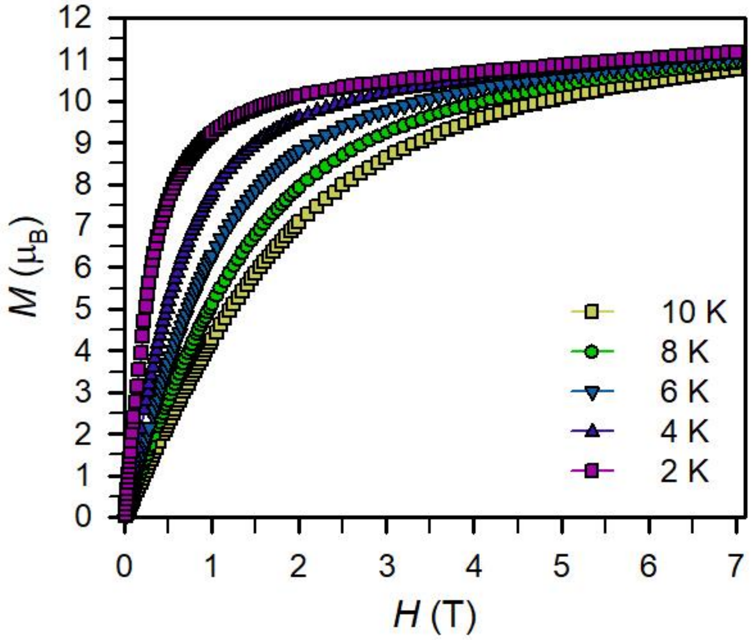

Figure S72. Variable temperature *M*(*H*) curves for **1c** collected from 0 to 7 T.

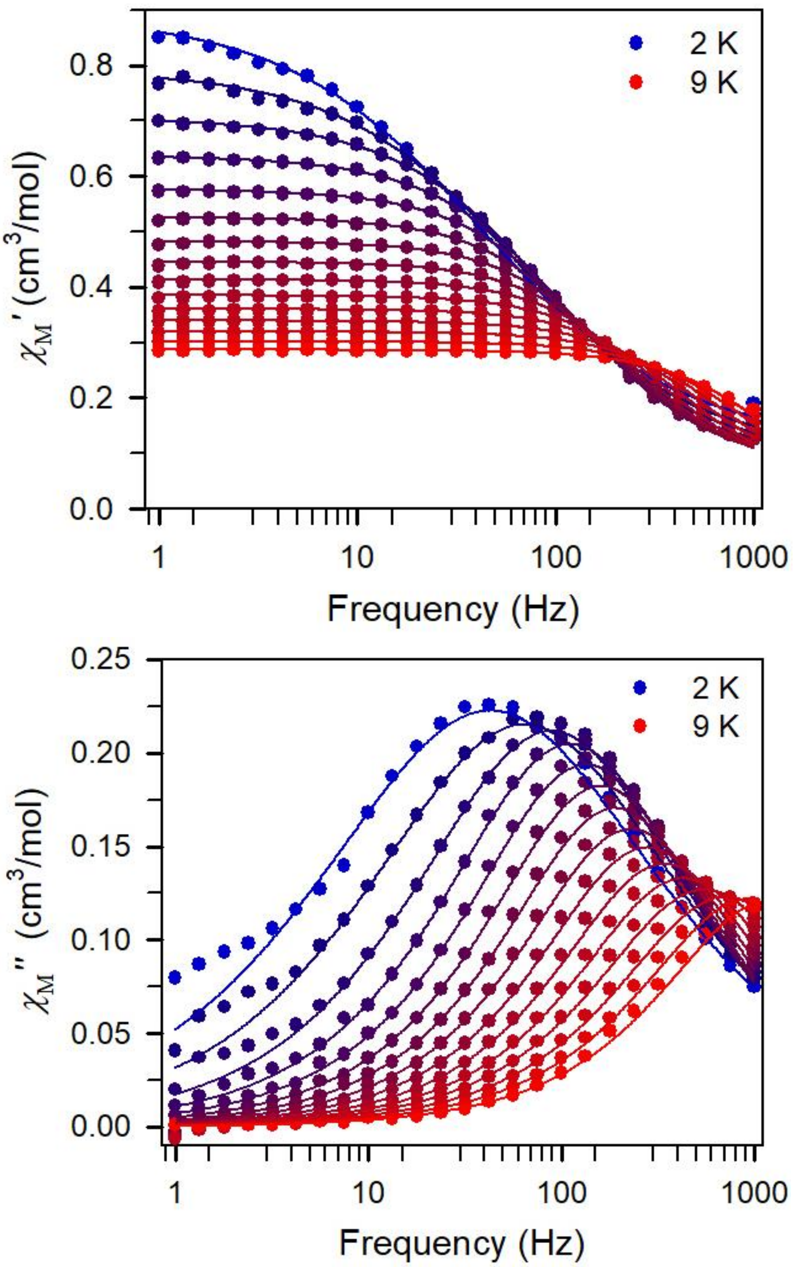

Figure S73. Variable-temperature, variable-frequency in-phase (χ_M', top) and out-of-phase (χ_M'', bottom) ac magnetic susceptibility data collected for **2b** under 2000 Oe applied dc field from 2 K (dark blue circles) to 9 K (red circles). Solid lines represent a fit to the data.

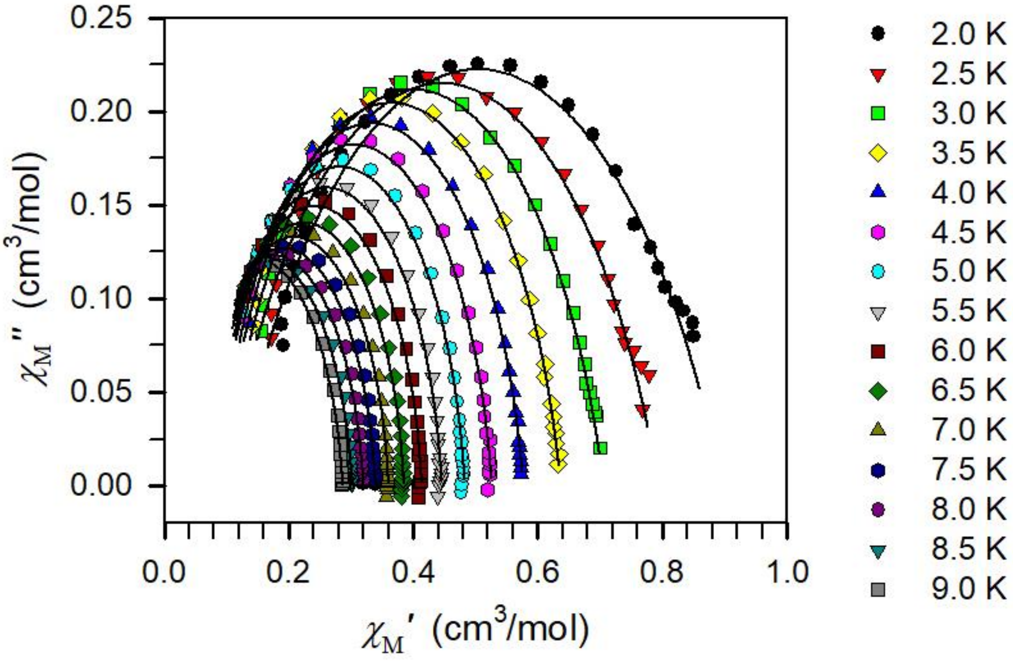

Figure S74. Cole-Cole (Argand) plots for ac susceptibility collected from 2 to 9 K under 2000 Oe applied dc field for **2b**. Symbols represent the experimental data points and the points representing the fits are connected by solid black lines.

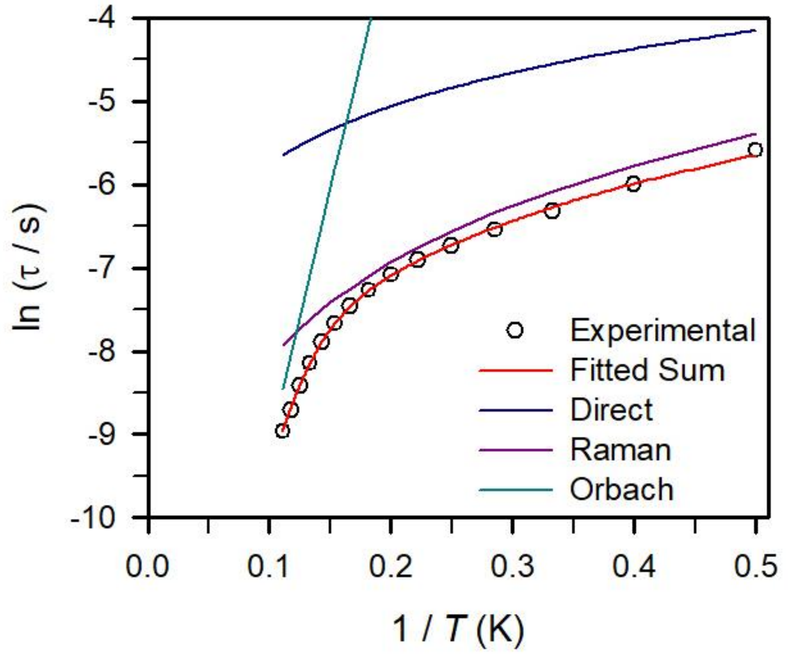

Figure S75. Individual contributions of the multiple magnetic relaxation pathways to the Arrhenius plots of **2b** at 2000 Oe. Individual parameters used to calculate the contributions are given in Table S31.

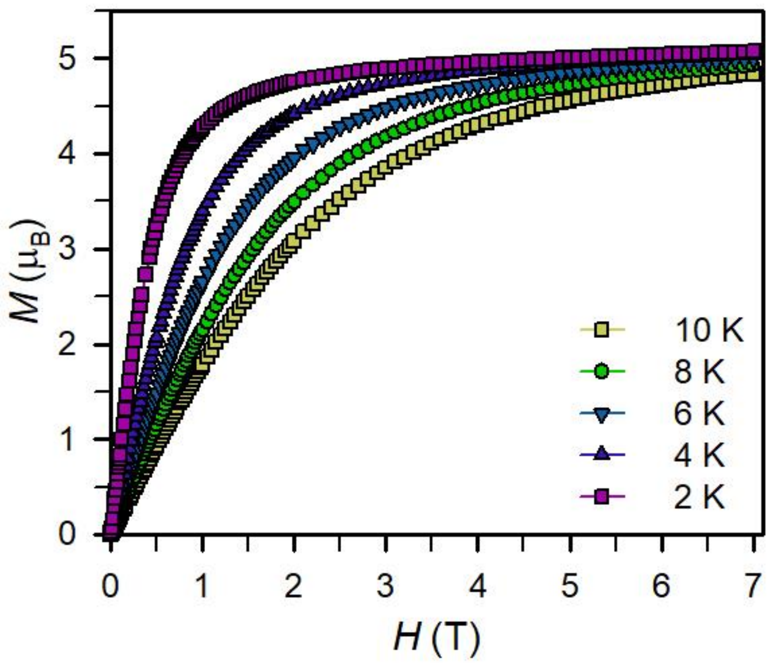

Figure S76. Variable temperature *M*(*H*) curves for **2b** collected from 0 to 7 T.

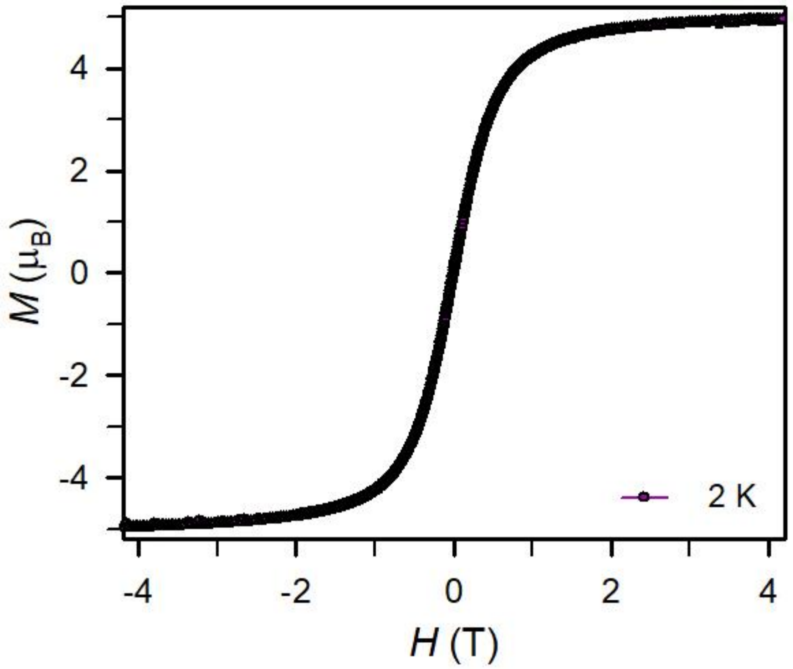

Figure S77. Variable field magnetization (*M*) data for **2b** collected at 2 K at an average sweep rate of 0.01 Ts^{-1}.

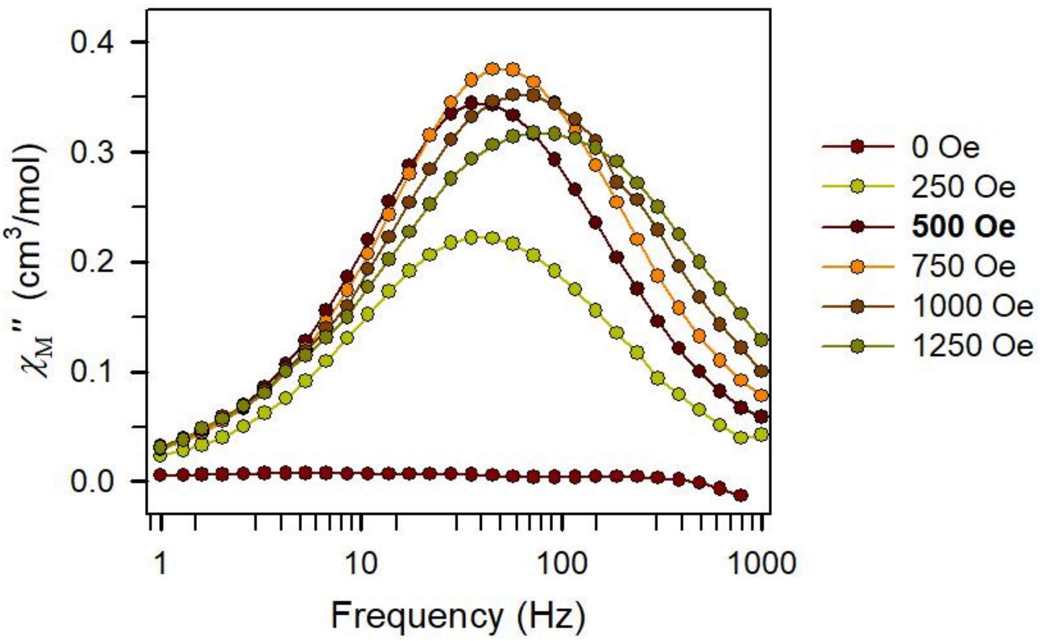

Figure S78. Out-of-phase ac susceptibility (χ_M'') collected on pure **2c** at 1.8 K under dc fields ranging from 0 Oe to 1250 Oe. Solid lines are guides for the eye.

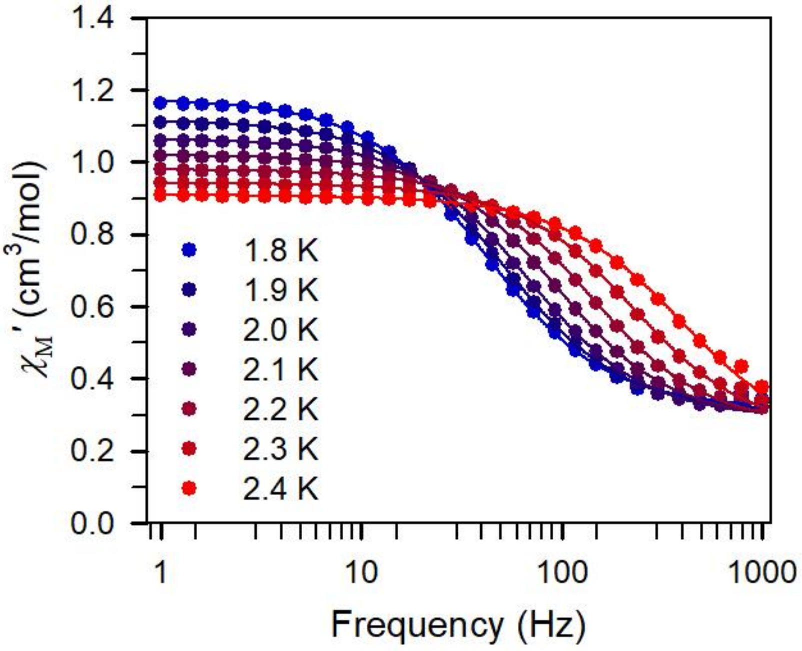

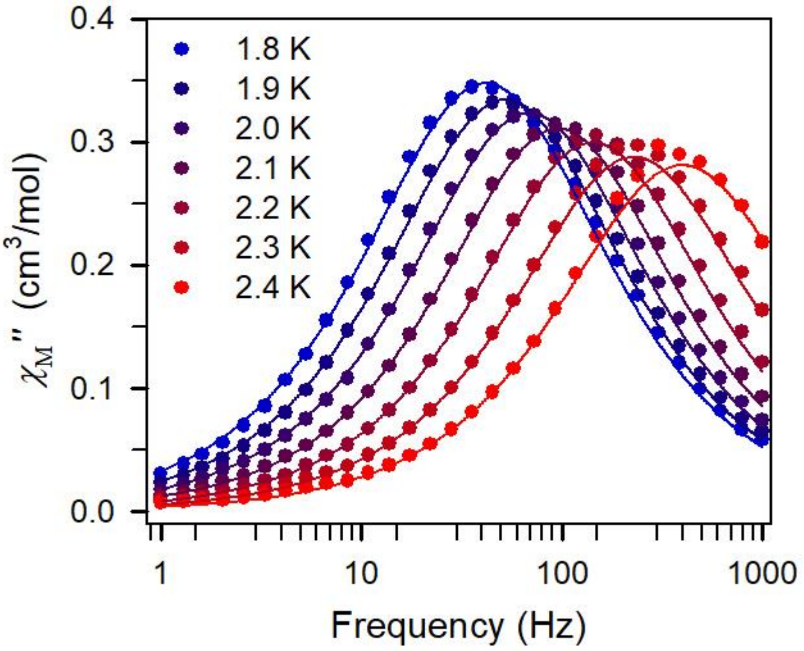

Figure S79. Variable-temperature, variable-frequency in-phase (χ_M', top) and out-of-phase (χ_M'', bottom) ac magnetic susceptibility data collected for **2c** under 500 Oe applied dc field from 1.8 K (dark blue circles) to 2.4 K (red circles). Solid lines represent a fit to the data.

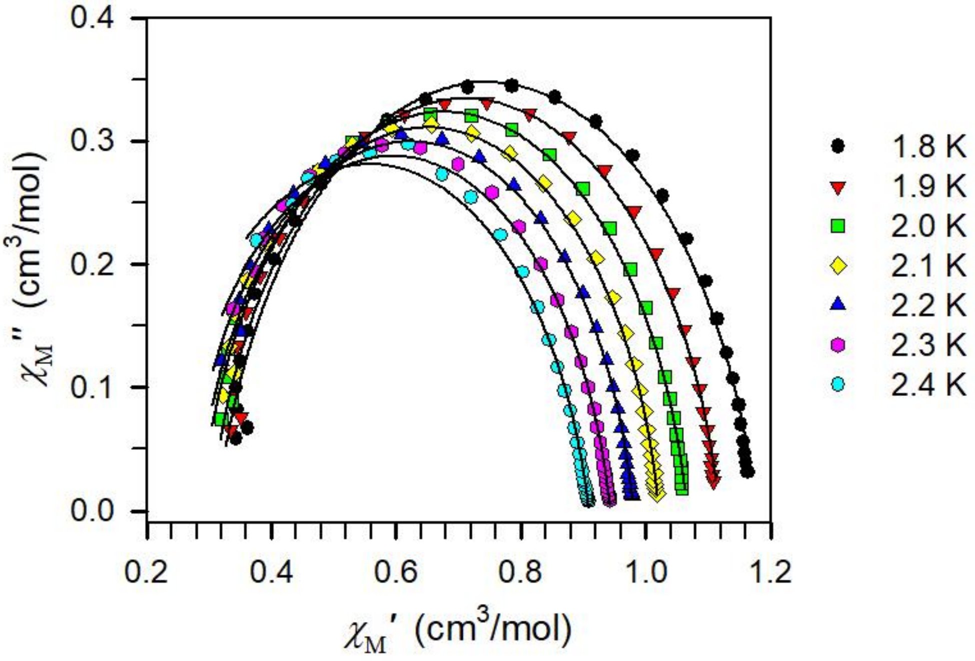

Figure S80. Cole-Cole (Argand) plots for ac susceptibility collected from 1.8 to 2.4 K under 500 Oe applied dc field for **2c**. Symbols represent the experimental data points and the points representing the fits are connected by solid black lines.

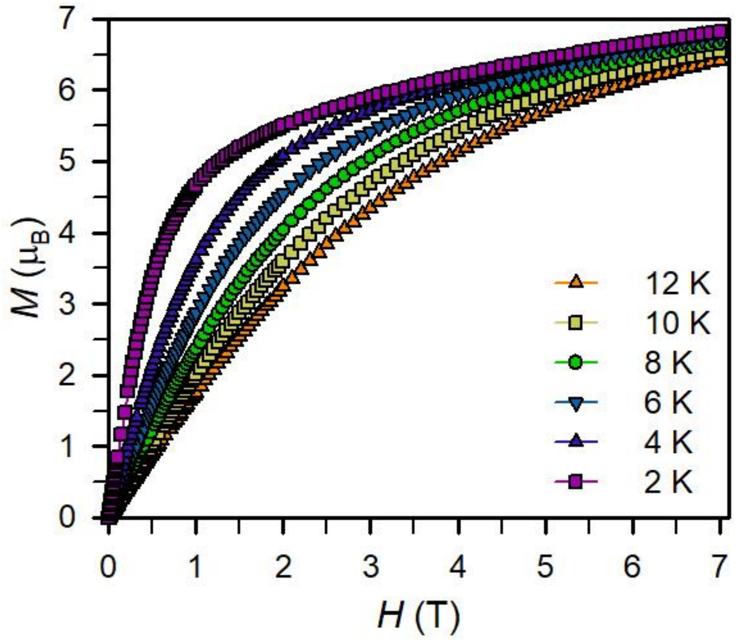

Figure S81. Variable temperature $M(H)$ curves for **2c** collected from 0 to 5 T.

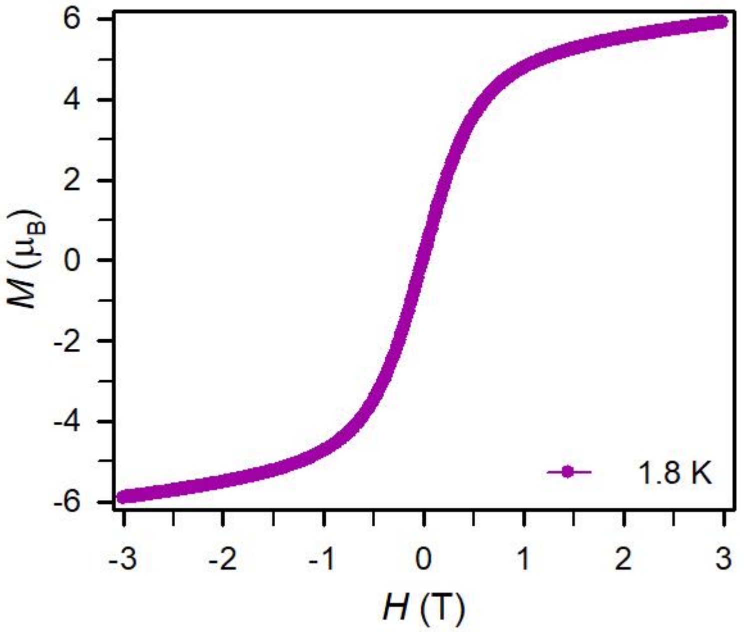

Figure S82. Variable field magnetization (*M*) data for **2c** collected at 1.8 K at an average sweep rate of 0.01 Ts^{-1}.

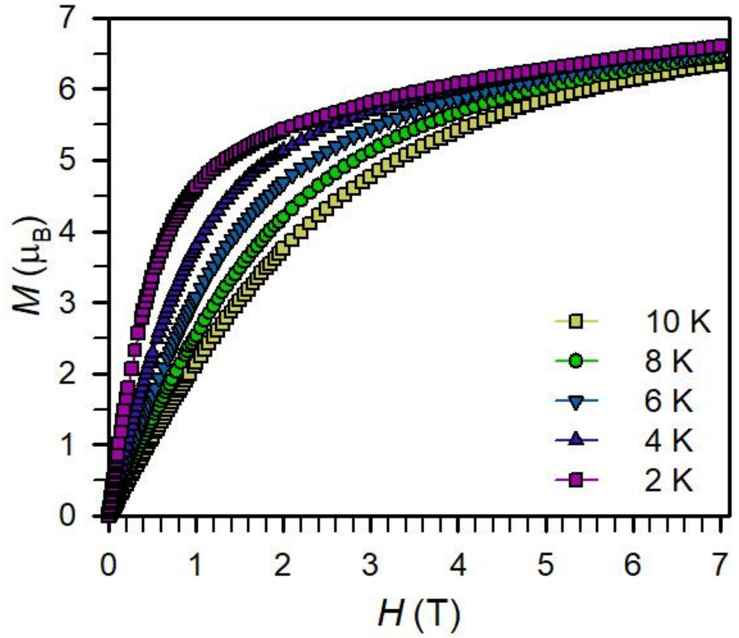

Figure S83. Variable temperature $M(H)$ curves for **2d** collected from 0 to 7 T.

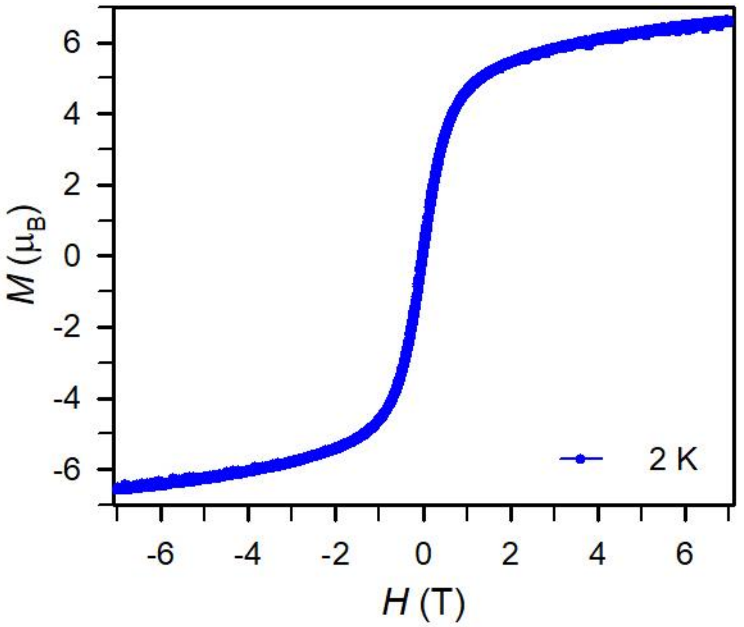

Figure S84. Variable field magnetization (*M*) data for **2d** collected at 2 K at an average sweep rate of 0.002 Ts^{-1}.

References

[1] (a) A. L. Spek, *Acta Cryst. D* **2009**, *65*, 148; (b) A. L. Spek, *Acta Cryst. C* **2015**, *71*, 9.

[2] Though these constants are well established in a variety of references, the following has an exceptional collection of almost all useful values for the necessary calculations: Bain, G. A.; Berry, J. F. *J. Chem. Educ.* **2008**, *85*, 532.

[3] Gatteschi, D., Sessoli, R., Villain, J. *Molecular Nanomagnets*, Oxford University Press, Oxford, **2006**.

[4] Eaton, G. R.; Eaton, S. S. In *Distance Measurements in Biological Systems by EPR*, Berliner, L. J., Ed.; Biological Magnetic Resonance, Vol. 19; Kluwer Academic/Plenum Publishers: New York, 2000.

[5] (a) Orbach, R. *Proc. R. Soc. London, Ser. A*, **1961**, *264*, 458; (b) Orbach, R. *Proc. R. Soc. London, Ser. A,* **1961**, *264*, 485; (c) Walker, M. B. *Can. J. Phys.*, **1968**, *46*, 1347.
